# Radical Control in O‑Atom Transfer from an Unusually Oxidized Oxorhenium Complex

**DOI:** 10.1021/acs.inorgchem.5c05970

**Published:** 2026-09-01

**Authors:** Jennifer A. Hill, Andrew G. Hill, Sulaiman Somani, Ashley M. McDaniel, Cameron A. Lippert, Grant R. Wilkinson, Kenneth I. Hardcastle, John Bacsa, Jake D. Soper

**Affiliations:** † School of Chemistry and Biochemistry, 1372 Georgia Institute of Technology, Atlanta, Georgia 30332-0400, United States; ‡ Department of Chemistry, 1371 Emory University, 1515 Dickey Drive, Atlanta, Georgia 30322, United States

## Abstract

A new S = 1/2 d^0^ dioxorhenium complex, [Re­(O)_2_(ap)­(isq^•^)] ([ap]^2–^ = 2,4-di-*tert*-butyl-6-(phenylamido)­phenolate, [isq^•^]^−^ = 2,4-di-*tert*-butyl-6-(phenylimino)­semiquinonate), was prepared by oxidation of the Re­(VII) species [Re­(O)_2_(ap)_2_]^−^. Solid-state structural and spectroscopic data for [Re­(O)_2_(ap)­(isq^•^)] suggest a strengthening of the Re=O bonding upon oxidation of [Re­(O)_2_(ap)_2_]^−^. [Re­(O)_2_(ap)­(isq^•^)] is a weak O-atom donor, H^•^ acceptor, and modest outer-sphere 1e^–^ oxidant, but it cleanly oxidizes the stable triphenylmethyl (Ph_3_C^•^) radical, affording Ph_3_COH and deoxygenated bimetallic μ-oxo dimers. Data support a mechanism of initial C–O radical coupling (RC) at a terminal Re=O bond, followed by net H^•^ transfer from Gomberg’s dimer, reversing the steps for classic rebound-type C–H hydroxylation. The closed-shell structural homologue [Re­(O)_2_(ap)_2_]^−^ has comparable O-atom transfer thermodynamics but is inert to Ph_3_C^•^. Computational data show the [isq^•^]^−^ radical in [Re­(O)_2_(ap)­(isq^•^)] is partially delocalized into the closed-shell metal–oxo group in the ground state, which might permit the net 2e^–^ oxo transfer to Ph_3_C^•^ to occur via kinetically facile ligand-centered radical steps. Accordingly, a strategy is presented for the preparation of stable oxo–metal complexes that exhibit oxidizing oxyl radical-type reactivity via delocalization of a low-lying, redox-active ligand-centered hole into the terminal M–O_oxo_ π-bonding manifold. This “masked oxyl” approach establishes design principles for generation of thermodynamically stable oxidants that are kinetically activated for selective odd-electron bond-making and -breaking redox reactions, with broad implications for selective oxidations and energy conversion and storage.

## Graphical abstract

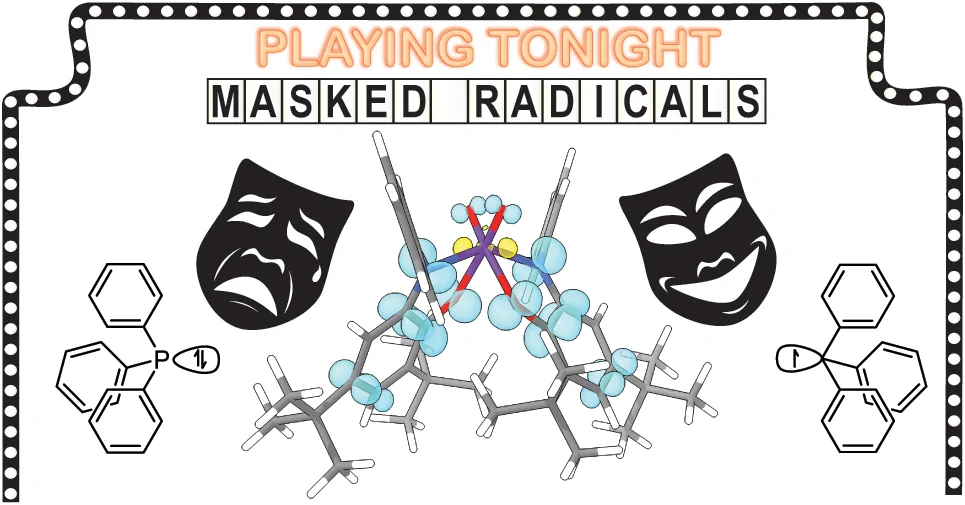

## Introduction

High valent metal–oxo species are the oxidants in processes ranging from industrial petroleum processing to biological detoxification, energy conversion and storage, environmental remediation, and benchtop organic synthesis.^1–17^ Accordingly, the electronic structures and reactivity of complexes containing metal–oxido bonds have been a subject of ongoing study for over 60 years.^6,18^ Most isolable metal–oxo complexes are closed shell species, but theoretical literature suggests metal–oxyl radials (M=O^•^) with unpaired spin density at the oxo ligand are the reactive intermediates in numerous low-barrier small molecule bond-making and -breaking redox reactions. These include, but are not limited to, O–O bond formation in natural and artificial photosynthetic systems,^19–24^ epoxidation,^25^ and C–H activation.^26–28^ Femtosecond spectroscopy experiments have observed Ti^III^–O^•^ transients upon photoexcitation of closed-shell titanosilicate Ti^IV^=O photocatalysts that are active for CO_2_ reduction and water splitting.,^29,30^ and gas-phase metal–oxide cations of the formula [MO]^•+^ (M = Mg, Mn, Fe) have been extensively investigated for their capacity to undergo thermal H atom abstraction from hydrocarbons including methane.^31–35^ A 2017 review attributed kinetically facile CH_4_ activation in the latter to “the presence of unpaired, high spin density at the abstracting, preferentially terminal oxygen atom”.^35^

Given the well-established reactivity of organic and small molecule free radicals, it seems intuitive that M=O^•^ species should be similarly biased for radical-type bond making and breaking. Accordingly, several synthetic water oxidation catalysts have been suggested to operate via a so-called radical coupling (RC) mechanism, whereby radical character at oxygen lowers the kinetic barrier to O–O bond formation.^22,24,36^ Although there is theoretical and computational support for this proposal in some systems, the general utility of oxygen radical character for bond-making reactivity at oxo ligands remains an open question.

The kinetic effects of unpaired spin on metal–oxo reactivity have been examined most extensively in C–H hydroxylations.^26,27,37^ Unpaired spin at the oxo ligand was historically considered a prerequisite for radical H atom abstraction at a terminal oxo ligand. This rationalized, for instance, the capacity of so-called Compound I (por^•^)­Fe^IV^=O species to abstract an H atom in the first step of the canonical hydroxyl rebound mechanism for alkane hydroxylation.^1^ But beginning in the mid-1980s, Mayer began compiling evidence that suggested radical character at oxygen was not a prerequisite for H atom abstraction by a terminal metal–oxo.^38,39^ In this model, C–H activation rates by M=O species are a consequence of the thermodynamic oxidizing power and basicity of the complex.

Despite their apparent importance, efforts to generate, stabilize, or rationally incorporate M=O^•^ species into functional catalysts have been mostly stymied. Isolated metal–oxyl complexes are exceedingly rare.^27,40–43^ Authentic terminal metal–oxyls, with unusually low M–O π bond orders and spin on oxygen, should be both thermodynamically unstable and kinetically reactive; the properties that make them desirable also make them elusive. Moreover, metal–oxyl radicals are unlike organic oxyls, particularly in their ability to delocalize the unpaired spin onto the metal center via the M–O π bonds. Accordingly, the electronic structure of a metal oxyl is described best by MO theory. The classic Ballhausen and Gray MO diagram for a terminal oxo complex in a tetragonal ligand field serves as a convenient starting point (Figure 1a).^18^ Generation of a metal–oxyl requires introduction of an unpaired spin into the metal-oxo π-bonding manifold. This can be accomplished in two ways as illustrated in Figure 1b: [1] addition of 1e^–^ to an antibonding M–O π* orbital; [2] removal of one electron from a doubly occupied π-bonding MO. Both lower the M–O bond order by 0.5. The former approach is more experimentally accessible. Reduction of a d^2^ oxo complex places a single electron into an e­(d_*xz*/yz_) π* MO, but if oxidizing metal complexes are desired, this is potentially problematic. First, because reduction of a d^2^ oxo complex results in occupation of a high energy orbital, the resulting metal complexes are outer-sphere reductants. Second, in cases where the metal center is more electropositive than oxygen—the typical situation for most Werner complexes—the primary contributors to the π* orbitals are the metal d_*xz*_ and d_*yz*_ atomic orbitals, so the unpaired spin is primarily localized on the metal. In contrast, oxidation of a d^0^ oxo complex will generate a strong oxidant. But because the e­(p_x/y_) π-bonding orbitals are typically low in energy and among other ligand-centered MOs, generation of this type of species is much more synthetically challenging and has not been conclusively demonstrated in condensed phases.

**Figure 1:**
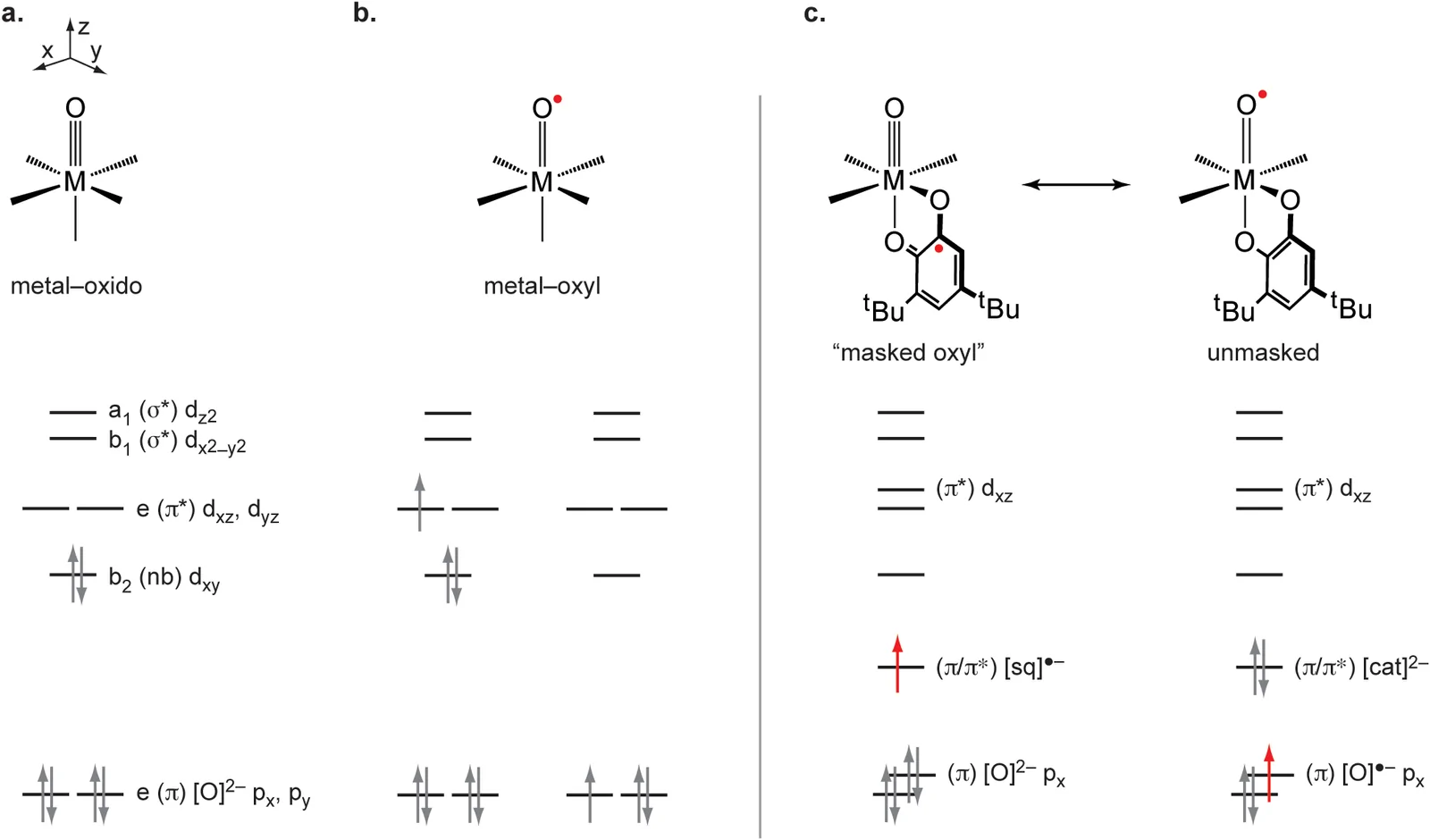
Qualitative MO diagrams illustrating strategies for generating metal–oxyl π radicals in a tetragonal ligand field. (a) A closed shell terminal oxido complex can accommodate up to 2 electrons in the nonbonding b_2_(d*_xy_*) orbital without perturbing the M≡O triple bond. (b) Addition of 1e^–^ to a M–O π* orbital (left) or removal of 1e^–^ from a π-bonding MO (right) introduces unpaired spin into the M–O π manifold and lowers the M–O bond order from 3.0 to 2.5. Both are appropriately formulated as metal–oxyl radicals. (c) Radical character is unmasked via delocalization of the semiquinonate [sq]^•–^ spin into the *trans*-disposed oxido [O]^2–^ ligand, conferring M=O^•^ oxyl radical character to the complex.

We proposed that redox-active ligands might confer oxidizing oxyl radical-type reactivity at closed-shell metal–oxo complexes.^44^ The key feature of this strategy is the introduction of a low-energy, ligand-centered hole that is appropriately positioned to delocalize into the M–O_oxo_ π-bonding manifold. For example, the semiquinonate [sq]^•–^ ligand *trans* to the oxo in Figure 1c has its unpaired electron in a π-symmetry MO that overlaps with d_*xy*_ as well as one of the e­(d_*xz*/yz_) orbitals that are used to form the M≡O π bonds. In the limit of full intraligand [O]^2–^ → [sq]^•–^ charge transfer (LLCT), the M–O_oxo_ bond order decreases from 3.0 to 2.5, making the complex an oxyl radical. Such excited state reactivity is a not a prerequisite, however, if symmetry allowed mixing of the [sq]^•–^ ligand with a M≡O π bond delocalizes the [sq]^•–^ SOMO across the M–O_oxo_ π system.

Presented below is a demonstration of this “masked oxyl” strategy. We describe the preparation and characterization of a new S = 1/2 d^0^ dioxorhenium complex with an iminosemiquinonate ligand-centered radical, one oxidation state above Re­(VII). Its capacity to oxidize the stable triphenylmethyl (Ph_3_C^•^) radical is reported. Computational data show that an iminosemiquinonate radical ligand imparts radical character to the closed-shell metal–oxo group, and mechanistic data are presented to suggest the radical reactivity of these complexes correlates with ligand spin density in the electronic ground state. These design principles form a basis for generation of a new class of stabilized complexes that behave like metal–oxyls.

## Results

### Oxidation of [Re­(O)_2_(ap)_2_]^−^

Our quest for a monomeric S = 1/2 oxorhenium complex that satisfied the “masked oxyl” design criteria in Figure 1c began with the previously reported [Re­(O)_2_(ap)_2_]^−^ anion ([ap]^2–^ = 2,4-di-*tert*-butyl-6-(phenylamido)­phenolate).^45^ The cyclic voltammogram (CV) of [Re­(O)_2_(ap)_2_]^−^ in CH_2_Cl_2_ solutions containing 0.1 M tetra-*n*-butylammonium hexfluorophosphate ([^n^Bu_4_N]­[PF_6_]) shows a single quasi-reversible oxidation at E_pa_ = −0.228 V vs Fc^+/0^ (Figure 2, blue). Accordingly, treatment of the navy blue MeCN solutions of [Re­(O)_2_(ap)_2_]^−^ with 1 equiv of a suitably strong oxidant ([Ag]­[PF_6_], [NO]­[BF_4_], or [Cp_2_Fe]­[PF_6_]) affords an immediate color change to dark green followed by precipitation of a green powder over 5 to 10 min at ambient temperature, which was isolated by vacuum filtration under N_2_. As discussed below, the choice of oxidant impacts the purity and stability of the inorganic product; the highest yields (87%) and cleanest product were obtained using [Cp_2_Fe]­[PF_6_] (eq 1). The green species is paramagnetic, as evidenced by a solution magnetic moment μ_eff_ = 1.99 μ_B_ (Evans’ method,^46^ CD_2_Cl_2_), which is close to the expected spin-only value for an S = 1/2 system.
1

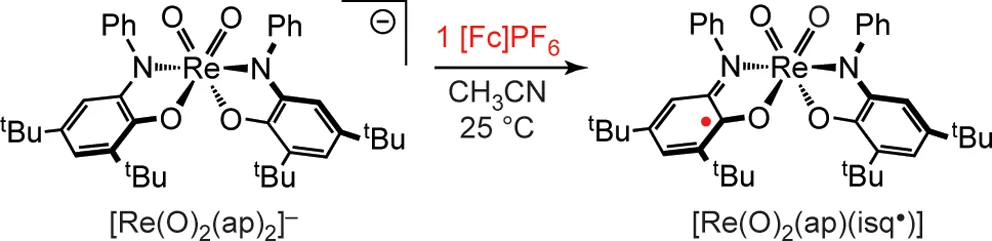




**Figure 2:**
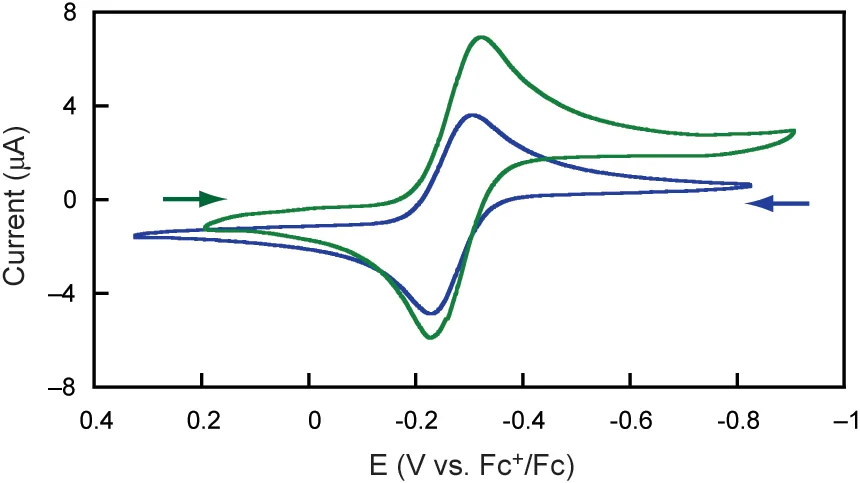
Cyclic voltammograms of [Re­(O)_2_(ap)_2_]^−^ (blue) and [Re­(O)_2_(ap)­(isq^•^)] (green) in CH_2_Cl_2_ containing 0.1 M [nBu_4_N]­[PF_6_], 10 mm Pt electrode, 100 mV s^–1^ scan rate, 25 °C.

The cyclic voltammogram of the green oxidation product (Figure 2, green) is essentially identical to that of [Re­(O)_2_(ap)_2_]^−^, except the open circuit potential is shifted to positive values such that the first observable redox event is a cathodic wave at E_pc_ = −0.323 V vs Fc^+/0^. The similarity of the voltammograms in Figure 2 imply that the isolated green product from chemical oxidation is the same one observed upon electrochemical 1e^–^ oxidation of [Re­(O)_2_(ap)_2_]^−^.

### Structure of [Re­(O)_2_(ap)­(isq^•^)]: 1. Solid State Structure

Slow diffusion of pentane into a saturated toluene solution of the green oxidation product at 25 °C in the absence of light affords single crystals suitable for analysis by X-ray diffraction. A thermal ellipsoid plot is shown in Figure 3. It contains a charge neutral rhenium center with pseudo-octahedral geometry. The gross coordination geometry is unchanged from [Re­(O)_2_(ap)_2_]^−^. Two terminal oxo ligands occupy *cis* coordination sites, as expected for a d^0^ metal center, with an O–Re–O bond angle of 101.2(2)°. The remaining four sites are filled by two bidentate aminophenol-derived ligands. The Re–N bonds are approximately *trans* disposed, so each oxo ligand is *trans* to a Re–O_ap_ bond. Selected bond distances for the [Re­(O)_2_(ap)_2_]^−^ anion and its 1e^–^ oxidation product are collected in Figure 4. Charge balance confirms the complex is one redox level above [Re­(O)_2_(ap)_2_]^−^, suggesting the solid-state structure is the same as the S = 1/2 species in solution (*vide supra*). The average Re–O_oxo_ bond lengths in the oxidized complex are slightly contracted, but statistically within error of those in [Re­(O)_2_(ap)_2_]^−^, consistent with modest enhancement of electrostatics and/or covalency in the Re–O bonding but inconsistent with oxidation of the d^0^ Re­(VII) center or an oxido ligand [O]^2–^. The observed *E*_1/2_ value is well within the range expected for generation of an iminosemiquinonate radical, however.

**Figure 3:**
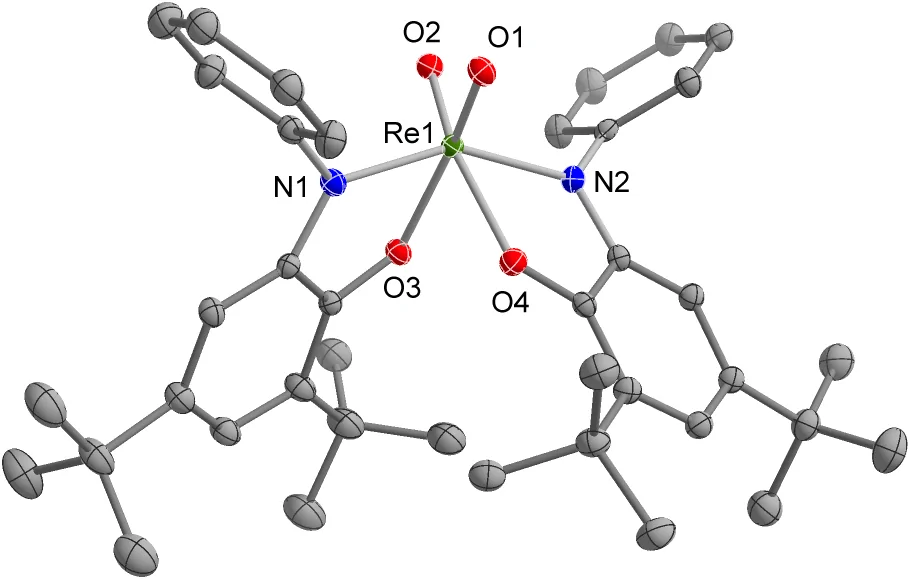
Solid-state structure of [Re­(O)_2_(ap)­(isq^•^)] shown with 50% probability ellipsoids. Hydrogen atoms omitted for clarity.

**Figure 4:**
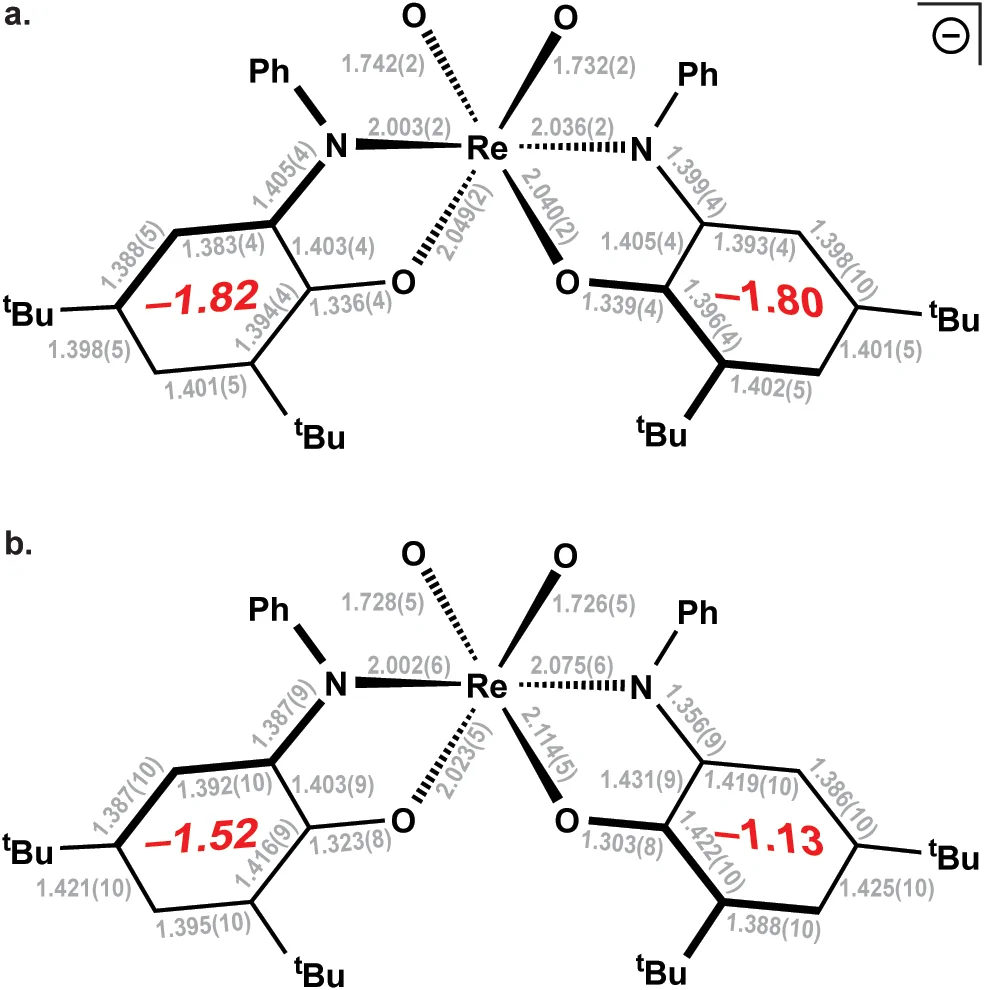
Schematic of selected bond lengths (Å) for complexes (a) [Re­(O)_2_(ap)_2_]^−^ and (b) its 1e^–^ oxidation product [Re­(O)_2_(ap)­(isq^•^)]. Red text are the computed metrical oxidation states (MOS) for each ligand.^47^

The aminophenol ligands in the oxidized complex are crystallographically inequivalent, and the bond distances suggest they are also electronically inequivalent (Figure 4b). Both show a quinoid-type pattern of four long and two short C–C bonds in the amidophenoxide moieties that typically accompanies ligand oxidation. The metrical oxidation state (MOS) values of −1.52 ± 0.11 and −1.13 ± 0.07 (Figure 4b) fall between the idealized closed-shell [ap]^2–^ ligand (−2.0) and an [isq^•^]^−^ radical (−1.0),^47^ and the sum of the two MOS values is −2.63 ± 0.13, seemingly suggesting oxidation of the ligands by more than 1e^–^. However, the total MOS values in diamagnetic [Re­(O)_2_(ap)_2_]^−^ (Figure 4a) are −3.62 ± 0.09. Brown has suggested that noninteger MOS values often reflect metal–ligand π-bonding interactions.^47^ In this case, the Re­(VII) centers in both [Re­(O)_2_(ap)_2_]^−^ and its oxidized congener should be excellent π-acceptors and the [ap]^2–^ ligands have filled π-symmetry MOs. Accordingly, the ca. +0.4 increase in the combined MOS values in both [Re­(O)_2_(ap)_2_]^−^ and the oxidized complex vs their idealized values can be ascribed to [ap]^2–^→ Re π donation. In sum, the metrical data are consistent with 1e^–^ oxidation of [Re­(O)_2_(ap)_2_]^−^, producing a product in which a total of 1.0 unit of charge is removed from the aminophenol ligands. For simplicity, the oxidized species is hereafter denoted [Re­(O)_2_(ap)­(isq^•^)] to emphasize the aminophenol ligand-centered hole. However, the solid-state data are insufficient to distinguish a localized vs delocalized charge distribution, and it is possible that the observed asymmetry in the ligands represents a crystal packing effect that is not representative of the solution structure, as discussed below.

To assess changes in Re=O bonding upon oxidation, solid-state infrared spectroscopy (ATR–FTIR) was performed on [Re­(O)_2_(ap)_2_]^−^ and [Re­(O)_2_(ap)­(isq^•^)] (Figure 5). Two bands at 874 and 847 cm^–1^ in the spectrum of [Re­(O)_2_(ap)_2_]^−^ are within the range typical of Re=O bonds and consistent with the ν_s_(ReO_2_) and ν_as_(ReO_2_) pair expected for a *cis*-ReO_2_ unit.^48^ These assignments were confirmed using ^18^O-labeled materials, which were prepared by aerobic oxidation of [Re­(O)­(ap)_2_]^−^ in MeCN solutions containing an excess of ^18^OH_2_. All three [Re­(^18/16^O)_2_(ap)_2_]^−^ isotopologues were observed by electrospray ionization mass spectrometry (ESI–MS); the major product in all preparations was [Re­(^18^O)­(^16^O)­(ap)_2_]^−^ as evidenced by a molecular ion at 811 *m*/*z*. Comparison of the FTIR spectrum of the ^18^O-enriched material to unlabeled [Re­(O)_2_(ap)_2_]^−^ shows a shift of the ReO_2_ bands to 828 and 800 cm^–1^ (Figure 5a), which matches closely the 43 cm^–1^ reduction in frequency computed using Hooke’s law. The ReO_2_ stretches in [Re­(O)_2_(ap)­(isq^•^)] appear at 910 and 875 cm^–1^ (Figure 5b). The shift to higher frequencies upon oxidation of [Re­(O)_2_(ap)_2_]^−^ is consistent with enhanced Re–O_oxo_ bonding in [Re­(O)_2_(ap)­(isq^•^)], mirroring the contraction of the Re–O_oxo_ bonds observed in the X-ray structural data described above. DFT computation (*vide infra*) of the normal modes of [Re­(O)_2_(ap)­(isq^•^)] predicts that the asymmetric and symmetric stretches occur at 935 and 906 cm^–1^, respectively. Three additional intense bands at 775, 738, and 581 cm^–1^ in the FTIR spectrum of [Re­(O)_2_(ap)­(isq^•^)] are assigned by DFT computation (793, 758, 597 cm^–1^) as in-plane breathing modes of the [isq^•^]^−^ ligand.

**Figure 5:**
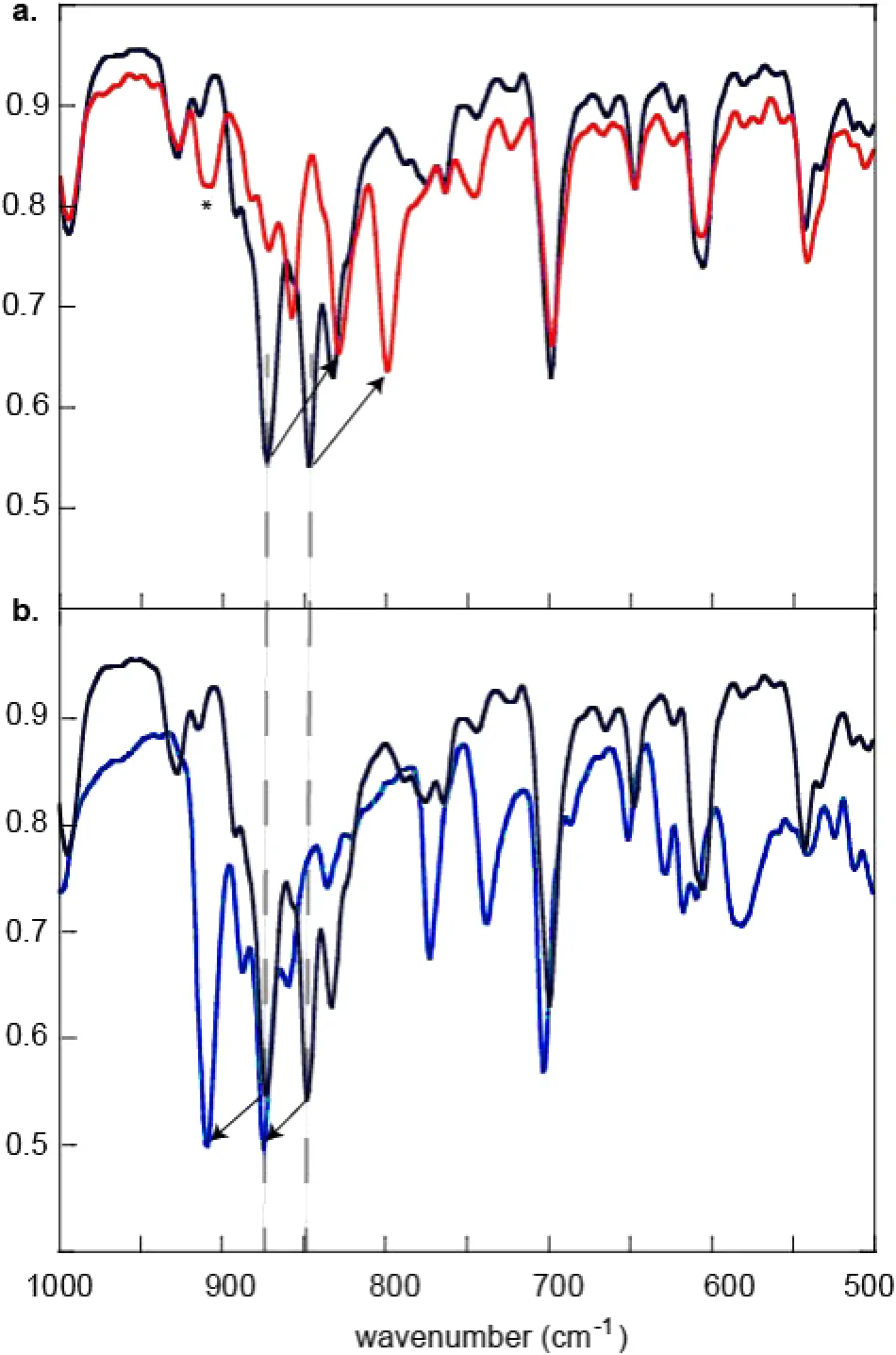
ATR–FTIR spectra of (a) [Re­(O)_2_(ap)_2_]^−^ (black) and [Re­(^18^O)­(^16^O)­(ap)_2_]^−^ (red), with arrows indicating shifts of the ReO_2_ stretching bands upon labeling, and (b) [Re­(O)_2_(ap)_2_]^−^ (black) and [Re­(O)_2_(ap)­(isq^•^)] (blue), with arrows indicating shifts the ReO_2_ bands upon oxidation. Bands at 775, 738, and 581 cm^–1^ in the blue spectrum are assigned to the [isq^•^]^−^ radical ligand. The peak labeled with an asterisk (*) is the [ReO_4_]^−^ byproduct from the aerobic synthesis of [Re­(^18^O)­(^16^O)­(ap)_2_]^−^.

### Structure of [Re­(O)_2_(ap)­(isq^•^)]: 2. EPR Spectroscopy

The X-band EPR spectrum of [Re­(O)_2_(ap)­(isq^•^)] was obtained in toluene glass at 77 K (Figure 6). ORCA simulations (*vide infra*) of the *g* values and hyperfine coupling constants (Table S2) were used as a guide to interpreting the spectrum. Hyperfine coupling to the amido nitrogen and two protons on each redox-active ring were considered in addition to the Re metal center. By inspection, the calculated values are consistent with axial *g* and hyperfine tensors, and the magnitude of all nitrogen and proton hyperfine coupling constants are smaller than 1 mT and thus not resolvable in the experimental spectrum. Considering this, the spectrum was fit in *EasySpin* considering only an axial *g* tensor and Re hyperfine coupling and the best fit was afforded for *g*_⊥_ = 2.008, *g*_∥_ = 2.005, *A*_⊥_(Re) = 37 MHz, and *A*_∥_(Re) = 139 MHz (see Experimental for full details). The observed *g* anisotropy is smaller than that predicted by the ORCA calculations, though it affords an identical *g*_avg_ value of 2.007 (2.007 predicted), and is close to the expected value for a free electron, consistent with a ligand-centered radical gaining minimal anisotropy through interaction with the Re­(VII) center and its significant spin–orbit coupling. The Re hyperfine coupling values obtained from the fit afford a slightly smaller *A*_avg_(Re) value of 71 MHz than the predicted 84.27 MHz magnitude from the ORCA calculation, and are intermediate between those previously reported for ligand-centered radicals coupled to Re­(I)^49,50^ (22–549 MHz) and metal-centered Re­(VI) species^51–53^ (130–2302 MHz). This is consistent with the expected higher metal–ligand bond covalency of Re­(VII) over Re­(I) and further validates the assignment of this complex as a ligand-centered radical coupled to a diamagnetic Re­(VII) metal center, rather than a Re­(VI) complex. Due to the large number of spin-active nuclei in this complex, spectral line-broadening was applied via H-strain (σ_H_) to account for the unresolvable nitrogen and proton interactions predicted by calculation (*vide supra*).

**Figure 6:**
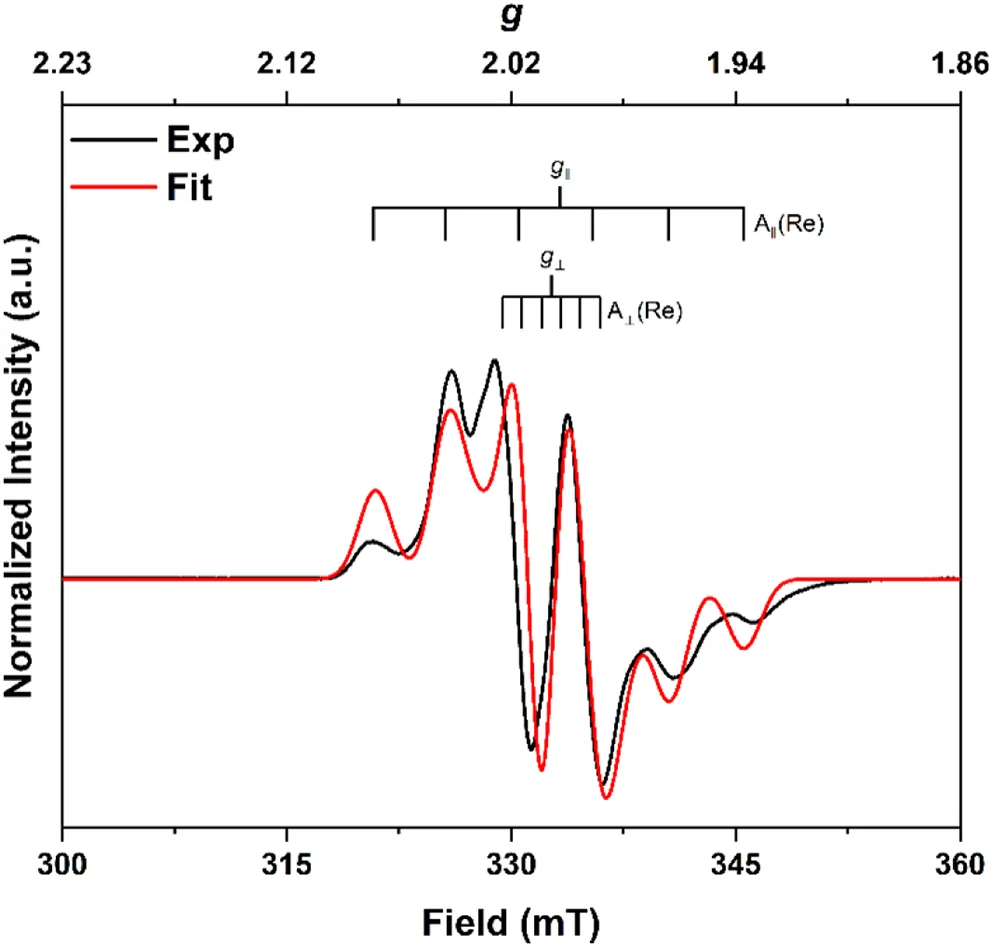
Experimental (black) and fit (red) EPR spectrum of [Re­(O)_2_(ap)­(isq^•^)] recorded at 9.35 GHz and 77 K on a 4 mM frozen toluene solution.

### Structure of [Re­(O)_2_(ap)­(isq^•^)]: 3. Computed Electronic Structure

The electronic structure of [Re­(O)_2_(ap)­(isq^•^)] was computed with unrestricted DFT calculations in ORCA 6.0.1 ^54–59^ on the full model (including ^t^Bu groups) using the M06-L functional,^60^ D3 dispersion correction,^61^ and zeroth-order regular approximation^62–64^ combined with Ahlrich’s def2-TZVPP basis set^65,66^ (ZORA-def2-TZVPP). The segmented all-electron relativistically contracted version of this basis set^67^ was used for Re. The data converged well as a doublet (⟨s^2^⟩ = 0.759). In contrast to the solid-state structure, the computed minimum is effectively symmetrical about the Re center, as manifested by the singly occupied molecular orbital (SOMO; Figure 7), which is distributed equally across both aminophenol ligands with the shape characteristic of an [isq^•^]^−^ radical. The use of implicit solvation (CPCM)^68^ did not result in an asymmetric geometric minimum in the low (toluene) or high (water) dielectric limit. The solution geometry may very well be different from the solid-state structure, which is influenced by packing effects. Consistent with its formulation as [Re­(O)_2_(ap)­(isq^•^)], the vast majority of the spin density is localized on the aminophenol-derived ligands (Figure S1), but the observation of 0.02 unpaired spins on each of the terminal oxo ligands is notable because it is consistent with oxyl character in the ground state, as proposed above. A small amount of spin-down density is localized on Re.

**Figure 7:**
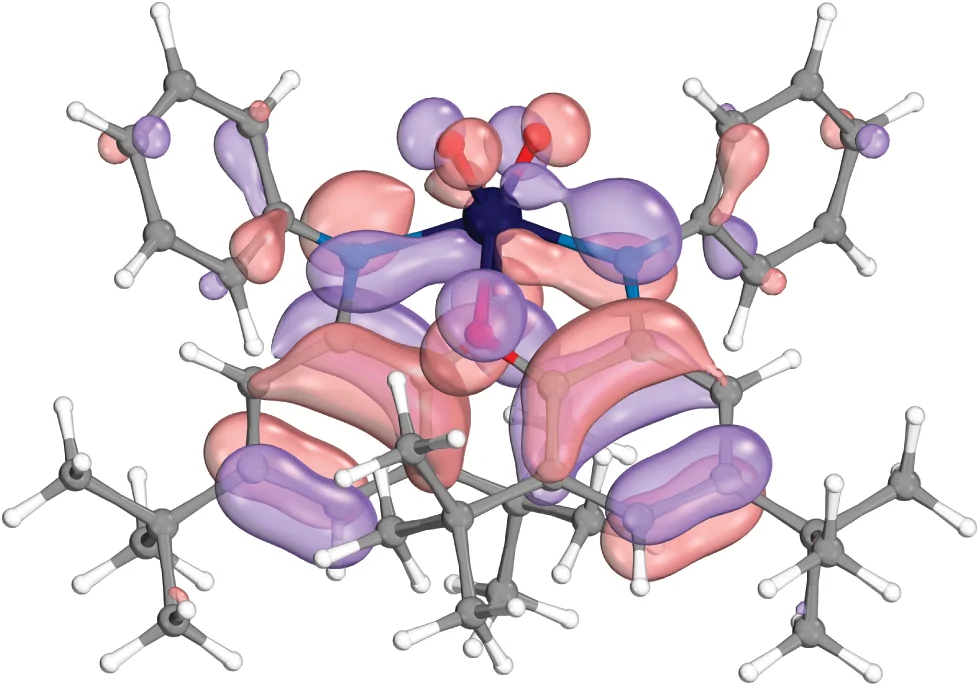
SOMO plot of [Re­(O)_2_(ap)­(isq^•^)] generated with IBOview (isosurface value 0.3).

### [Re­(O)_2_(ap)­(isq^•^)] O-Atom Transfer: 1. Via H-Atom Abstraction

Initial attempts to grow X-ray quality single crystals of [Re­(O)_2_(ap)­(isq^•^)] were challenged by a degradation reaction. This was first observed in acetone solutions of green [Re­(O)_2_(ap)­(isq^•^)], which become an intense purple color after 1 day at ambient temperature under N_2_ (Figure 8a). Removal of the solvent under vacuum afforded a purple powder, and MALDI–MS analysis of the isolated solids showed a molecular ion peak at 1602 *m*/*z*, corresponding to the molecular weight of two equivalents of [Re­(O)_2_(ap)­(isq^•^)] less one O atom. The rate of this conversion varies with solvent. Whereas UV–vis spectra of [Re­(O)_2_(ap)­(isq^•^)] in THF or CH_2_Cl_2_ show features characteristic of the degradation product in hours under N_2_ and ambient light (Figure 8b), the UV–vis spectrum [Re­(O)_2_(ap)­(isq^•^)] in C_6_H_6_ is essentially unchanged over 24 h, and 14 d were required to observe a comparable (15%) yield of the purple product (Figure 8c). Formation of the purple material is accelerated slightly by exposure to air, but light significantly increases the rate of the degradation reaction. Exposure of a C_6_H_6_ solution of [Re­(O)_2_(ap)­(isq^•^)] to a 16 W 2700 K (soft white) compact fluorescent light (CFL) gave ca. 15% conversion to the purple product over 24 h. Conversely, the UV–vis spectrum of [Re­(O)_2_(ap)­(isq^•^)] in CH_2_Cl_2_ was unchanged over 4 d in the dark at ambient temperature.

**Figure 8:**
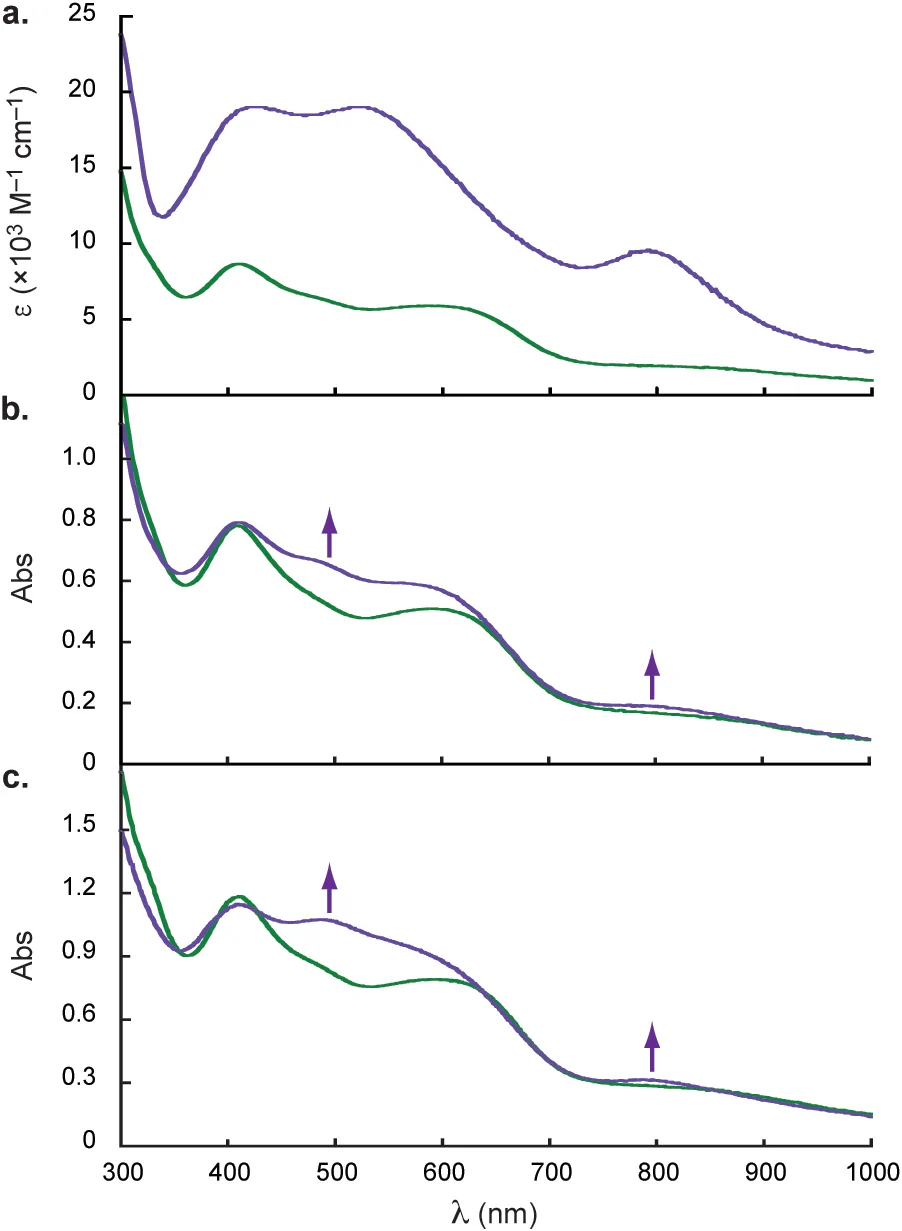
(a) UV–vis absorption spectra of [Re­(O)_2_(ap)­(isq^•^)] (green line) and [Re_2_(μ-O)­(O)_2_(ap)_2_(isq^•^)_2_] (purple line) in C_6_H_6_. (b) THF solution of 9.3 × 10^–5^ M [Re­(O)_2_(ap)­(isq^•^)] *t* = 0 (green line) and *t* = 24 h (purple line) under N_2_ at 25 °C showing 13% conversion to [Re_2_(μ-O)­(O)_2_(ap)_2_(isq^•^)_2_]. (c) C_6_H_6_ solution of 1.6 × 10^–4^ M [Re­(O)_2_(ap)­(isq^•^)] at *t* = 0 (green line) and *t* = 14 d (purple line) under N_2_ at 25 °C showing 15% conversion to [Re_2_(μ-O)­(O)_2_(ap)_2_(isq^•^)_2_]. % conversion determined by iterative fits of the UV–vis data to linear combinations of the isolated spectra for [Re­(O)_2_(ap)­(isq^•^)] and [Re_2_(μ-O)­(O)_2_(ap)_2_(isq^•^)_2_].

X-ray quality crystals of the purple degradation product were obtained by slow evaporation of a saturated MeCN solution at −20 °C. The solid-state structure (Figure 9a) contains a charge-neutral oxo-bridged dirhenium complex with approximate *C*_2_ symmetry. Each Re center has pseudo-octahedral geometry. Two terminal oxo ligands are *cis* to the μ-oxo linkage, and the Re–O_oxo_ bonds are approximately eclipsed (γ = 6.6° and 10.6° in the two crystallographically independent dimer molecules). Two *cis*-oriented bidentate aminophenol-derived ligands take up the remaining four sites about each Re with Re–N bonds *trans* to the μ-oxo ligand and a Re–O *trans* to each of the terminal oxos. Examination of the aminophenol ligand bond distances suggests the symmetry-inequivalent ligands are also electronically inequivalent (Figure 9b). In the ligand *trans* to the μ-oxo bridge, the C–C bond distances within the aminophenol ring are equidistant within 3σ (1.40 ± 0.02 Å) and the C–N and C–O bond distances are consistent with single bonds, suggesting the ligands are fully reduced, closed-shell [ap]^2–^ dianions. In contrast, the aminophenol rings *trans* to the terminal oxo groups have contracted C–N and C–O bond distances and show quinoid-type deviations from aromaticity, typical of the radical [isq^•^]^−^ semiquinonate monoanion. These assignments are reflected in the mean computed MOS values of −1.86 ± 0.23 and −1.24 ± 0.22 for the ligands *trans* to Re–O_μ‑oxo_ and Re–O_oxo_, respectively (Figure 9b).^47^ The sum of these data suggest that the dimeric complex is best formulated as [Re_2_(μ-O)­(O)_2_(ap)_2_(isq^•^)_2_], with the radicals primarily localized on the ligands *trans* to the terminal oxos.

**Figure 9:**
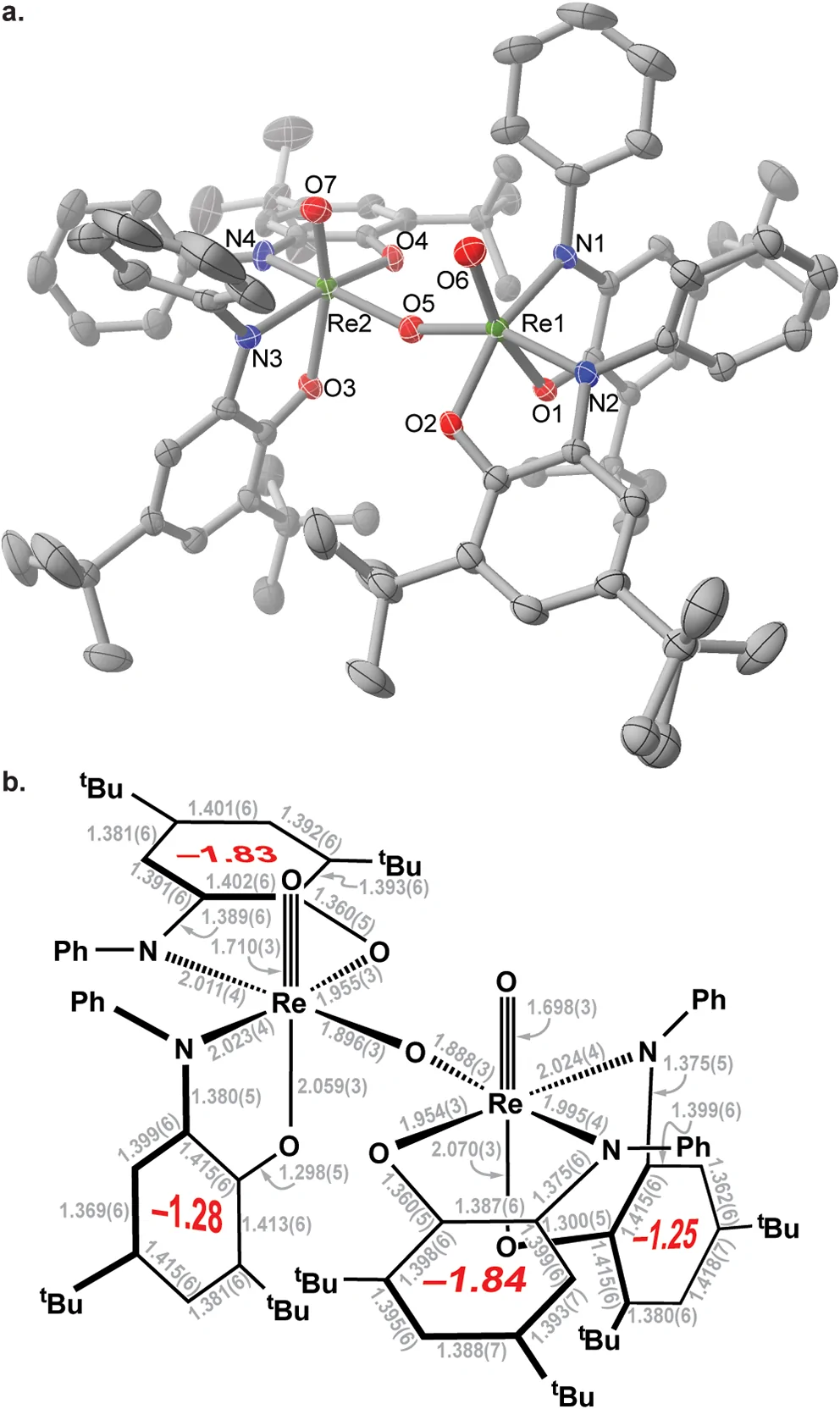
(a) Solid-state structure of [Re_2_(μ-O)­(O)_2_(ap)_2_(isq^•^)_2_] shown with 50% probability ellipsoids. Hydrogen atoms omitted for clarity. A second, crystallographically unique dimer is not shown. (b) Schematic of selected bond lengths (Å) and computed MOS values (red) for each ligand in one dimer molecule.^47^

Formation of [Re_2_(μ-O)­(O)_2_(ap)_2_(isq^•^)_2_] from 2 equiv [Re­(O)_2_(ap)­(isq^•^)] is a net 2e^–^ process, wherein each Re center is reduced from Re­(VII) to Re­(VI). The mean terminal Re–O_oxo_ bond distance of 1.708(8) Å in [Re_2_(μ-O)­(O)_2_(ap)_2_(isq^•^)_2_] is within the range expected for monooxo Re≡O triple bonds and matches the Re–O_oxo_ bonds in previously reported monomeric [Re­(O)­(ap)­(isq^•^)­X] complexes.^44^ These data are consistent with the added electron occupying a π-nonbonding orbital (d_*xy*_ when the terminal Re–O_oxo_ bond is coincident with the *z* axis). The long Re–Re distances of 3.705(6) and 3.673(6) Å preclude metal–metal bonding. Formulation as [Re_2_(μ-O)­(O)_2_(ap)_2_(isq^•^)_2_] implies four total unpaired spins, with two d^1^ ions each coordinated to a free radical [isq^•^]^−^ ligand, but the complex is diamagnetic, as evidenced by the observation of sharp ^1^H NMR resonances (δ = +0.00 to 8.00 ppm) in CDCl_3_ at 25 °C (Figure S2), suggesting the spins are antiferromagnetically coupled, as discussed below.

Conversion of two equivalents of [Re­(O)_2_(ap)­(isq^•^)] to one [Re_2_(μ-O)­(O)_2_(ap)_2_(isq^•^)_2_] is balanced by loss of one O atom. This is sometimes termed “incomplete” oxo transfer.^69^ The fate of the O atom here is unknown. Kinetics of the degradation in CH_2_Cl_2_ were monitored by UV–vis spectroscopy at varying initial Re concentrations. The data exhibited a first-order dependence on [Re­(O)_2_(ap)­(isq^•^)] (Table S1), which suggests that bimetallic RC-type coupling to produce O_2_ is unlikely. As described above, the rate of the dimerization reaction is qualitatively faster in solvents with weaker C–H bonds, suggesting that the reduced μ-oxo product might be generated by transfer of H^•^ equivalents to [Re­(O)_2_(ap)­(isq^•^)]. The anticipated water or solvent-derived oxidation products of this reaction were not observed by mass spectrometry or NMR spectroscopy, but addition of 1 equiv 9,10-dihydroanthracene (DHA) to [Re­(O)_2_(ap)­(isq^•^)] in C_6_H_6_ produced water and anthracene (16%) over 24 h at ambient temperature, as evidenced by ^1^H NMR (Figure S3), as well as quantitative conversion to [Re­(μ-O)_2_(ap)­(isq^•^)] by UV–vis spectroscopy (eq 2). We have previously reported the formation of [Re_2_(μ-O)_2_(ap)_2_(isq^•^)_2_] from rapid dimerization of [Re­(O)­(ap)­(isq^•^)], and data below show [Re_2_(μ-O)_2_(ap)_2_(isq^•^)_2_] is also generated by O-atom transfer from [Re_2_(μ-O)­(O)_2_(ap)_2_(isq^•^)_2_] (Scheme 1). Accordingly, the apparent intermediacy of [Re­(O)­(ap)­(isq^•^)] or [Re_2_(μ-O)­(O)_2_(ap)_2_(isq^•^)_2_] in the reaction with DHA suggests facile deoxygenation upon net transfer of two H^•^ to [Re­(O)_2_(ap)­(isq^•^)].
2

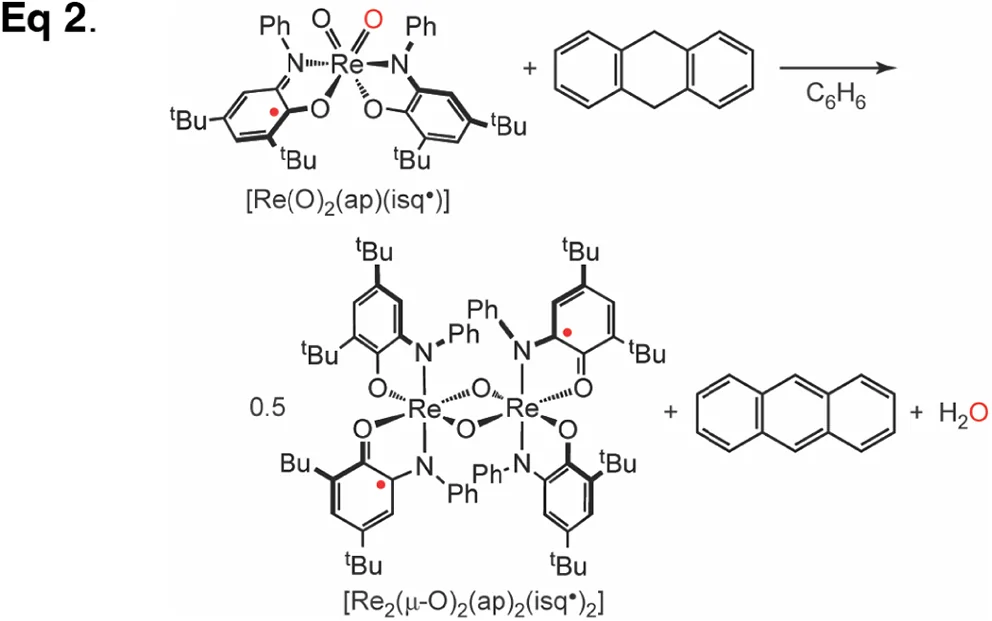




**Scheme 1:**
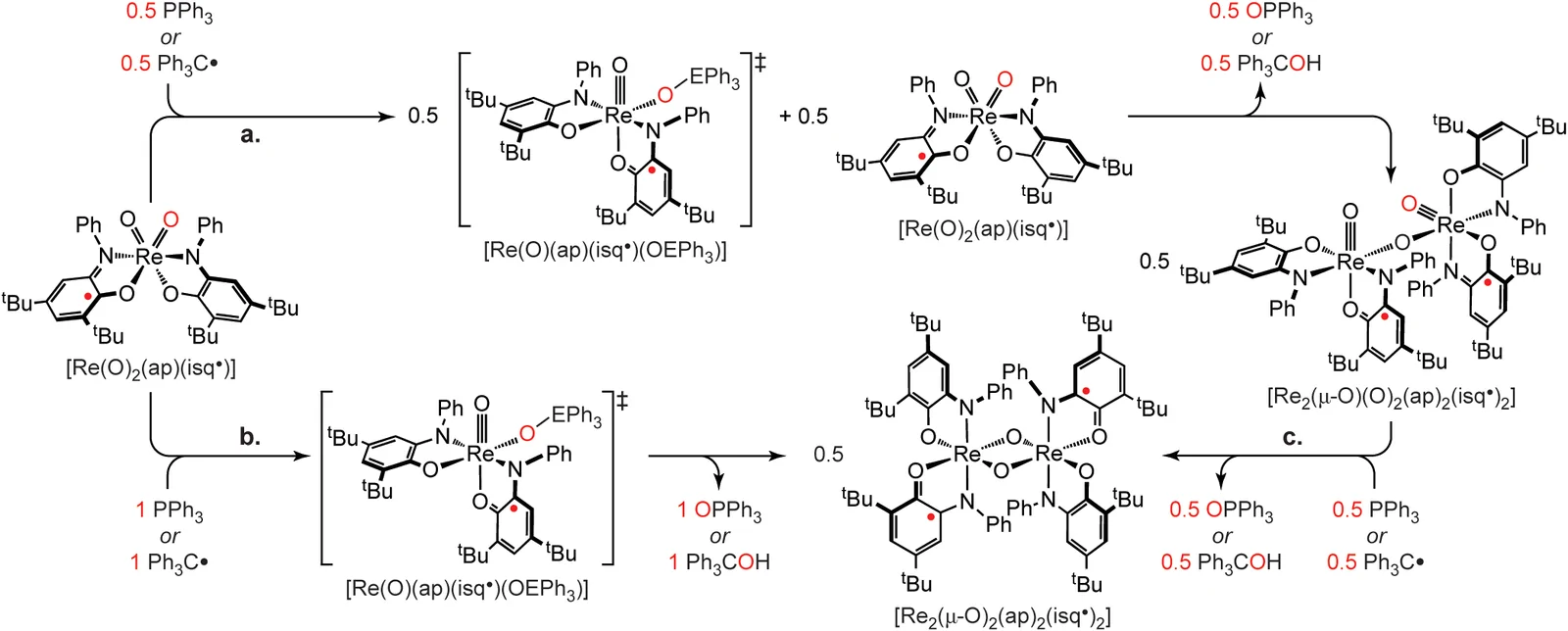
Routes to μ-Oxo-Bridged Dimers from the Reactions of [Re­(O)_2_(ap)­(isq^•^)] with PPh_3_ or Ph_3_C^•^

### [Re­(O)_2_(ap)­(isq^•^)] O-Atom Transfer: 2. PPh_3_ Oxidation

Treating a green CH_2_Cl_2_ solution of [Re­(O)_2_(ap)­(isq^•^)] with 0.5 equiv PPh_3_ affords an immediate color change to dark purple with a UV–vis spectrum that is identical to isolated [Re_2_(μ-O)­(O)_2_(ap)_2_(isq^•^)_2_]. Concomitant quantitative formation of OPPh_3_ was confirmed by ^1^H NMR spectroscopy (Figure S2) and GC-MS (Figure S4). Accordingly, the conversion of 2 [Re­(O)_2_(ap)­(isq^•^)] + PPh_3_ → [Re_2_(μ-O)­(O)_2_(ap)_2_(isq^•^)_2_] + OPPh_3_ is a balanced reaction that can be envisioned to occur by direct PPh_3_ attack at an oxido ligand in [Re­(O)_2_(ap)­(isq^•^)] followed by displacement of the resulting OPPh_3_ by a second equivalent of [Re­(O)_2_(ap)­(isq^•^)], as shown in Scheme 1a.

Addition of 10 equiv PPh_3_ to CH_2_Cl_2_ solutions of [Re­(O)_2_(ap)­(isq^•^)] produces a different purple solution, with a UV–vis absorption spectrum that matches exactly the bis­(μ-oxo) dirhenium complex [Re_2_(μ-O)_2_(ap)_2_(isq^•^)_2_]. As noted above, formation of [Re_2_(μ-O)_2_(ap)_2_(isq^•^)_2_] was previously postulated to occur by rapid dimerization of the putative monooxo species *cis*-[Re­(O)­(ap)­(isq^•^)].^44^ An unobserved solvent or OPPh_3_ adduct is proposed in Scheme 1, but the mechanism of substitution (associative vs dissociative) cannot be inferred from the experimental data herein.^79^ In this context, the reaction with excess PPh_3_ might completely consume [Re­(O)_2_(ap)­(isq^•^)], driving the reaction toward the bis­(μ-oxo) via the [Re­(O)­(ap)­(isq^•^)­(OPPh_3_)] transient (Scheme 1b). However, the same [Re_2_(μ-O)_2_(ap)_2_(isq^•^)_2_] dimer is quantitatively obtained upon treating [Re_2_(μ-O)­(O)_2_(ap)_2_(isq^•^)_2_] with 1 equiv PPh_3_ (Scheme 1c). The net reaction stoichiometry is the same following either the **a** + **c** or **b** paths in Scheme 1: 2 [Re­(O)_2_(ap)­(isq^•^)] + 2 PPh_3_ → [Re_2_(μ-O)_2_(ap)_2_(isq^•^)_2_] + 2 OPPh_3_. Accordingly, the potential intermediacy of [Re_2_(μ-O)­(O)_2_(ap)_2_(isq^•^)_2_] in the reaction with excess PPh_3_ cannot be inferred from the observed product distribution.

### [Re­(O)_2_(ap)­(isq^•^)] O-Atom Transfer: 3. Ph_3_C^•^ Trapping

Treating solutions of [Re­(O)_2_(ap)­(isq^•^)] with triphenylmethyl radical Ph_3_C^•^ affords a color change from green to purple over hours at ambient temperature in C_6_H_6_ or over ∼1 min in CH_3_CN. The inorganic products of this reaction parallel exactly those described above for O-atom transfer to PPh_3_ (Scheme 1). Accordingly, reaction with 0.5 equiv Ph_3_C^•^ followed by column chromatography on silica gel gave [Re_2_(μ-O)­(O)_2_(ap)_2_(isq^•^)_2_] as the major Re-containing product, as evidenced by its characteristic UV–vis and MALDI–MS spectra. Reaction of [Re­(O)_2_(ap)­(isq^•^)] with excess Ph_3_C^•^, or addition of 1 equiv Ph_3_C^•^ to isolated [Re_2_(μ-O)­(O)_2_(ap)_2_(isq^•^)_2_], in C_6_H_6_ gave quantitative conversion to [Re_2_(μ-O)_2_(ap)_2_(isq^•^)_2_]. Because the triphenylmethyl radical monomer–dimer equilibrium in C_6_H_6_ strongly favors the Gomberg dimer (ca. 2% Ph_3_C^•^), the majority of the stoichiometric reactions occur with a large excess of dissolved [Re] relative to [Ph_3_C^•^]. Gas chromatography–mass spectrometry (GC–MS) analysis of the reaction mixtures revealed that triphenylmethanol Ph_3_COH was the major organic product under all conditions. Minor products included benzophenone and triphenylmethane (Figures S5–S7). The Ph_3_COH yields vary with different preparations of [Re­(O)_2_(ap)­(isq^•^)]. For reactions of [Re­(O)_2_(ap)­(isq^•^)] + 0.5 Ph_3_C^•^, quantification by GC–FID gave Ph_3_COH yields as high as 69% against a control reaction containing no Re; average Ph_3_COH yields were 45% after 24 h. The substoichiometric organic yields reflect competing side reactions, including consumption of the Gomberg dimer by the product of C–O bond formation, as detailed below. Exclusion of ambient light had no measurable impact on the Ph_3_COH yield. Reaction of Ph_3_C^•^ + O_2_ without Re gives no Ph_3_COH under conditions analogous to those employed in Scheme 1, suggesting autoxidation is not the primary path to Ph_3_COH.

The energetics of electron transfer were evaluated in the context of the observed solvent dependence on the reaction rates. The independently measured *E*_1/2_ values for [CPh_3_]^+/0^ = −0.11 V and [Re­(O)_2_(ap)­(isq^•^)]/[Re­(O)_2_(ap)_2_]^−^ = −0.125 V vs Fc^+/0^ imply that outer-sphere ET from Ph_3_C^•^ to [Re­(O)_2_(ap)­(isq^•^)] in CH_3_CN is uphill by approximately +0.3 kcal mol^–1^. Hess’s law DFT calculations (M06-L, def2-TZVPP, ZORA, CPCM) in simulated CH_3_CN give good qualitative agreement, with ΔG_ET_ = +1.8 kcal mol^–1^ for formation of the [CPh_3_]­[Re­(O)_2_(ap)_2_] ion pair. Electrochemical measurements in C_6_H_6_ were not viable, but analogous calculations in C_6_H_6_ pseudosolvent reveal that the ion pair is significantly destabilized in C_6_H_6_, with ΔG_ET_ = +31 kcal mol^–1^ vs the neutral radicals. Implications for the mechanism of C–O bond formation are discussed below.

In total, the observed Re and organic products are consistent with the oxo-transfer reaction stoichiometries shown in Scheme 2, where X = PPh_3_ or Ph_3_C^•^. However, the reactions with Ph_3_C^•^ are not balanced because each equivalent of Ph_3_COH generated requires one additional H^•^. The immediate product of O atom addition to Ph_3_C^•^ is a triphenylmethoxyl Ph_3_CO^•^ radical, which undergoes a rapid 1,2-phenyl shift to afford the α-phenoxyldiphenylmethyl radical.^70^ Accordingly, high yields of Ph_3_COH are inconsistent with generation of a free Ph_3_CO^•^ radical intermediate, suggesting the “adventitious” H^•^ is delivered to an intact Re–OCPh_3_ bond.

**Scheme 2:**
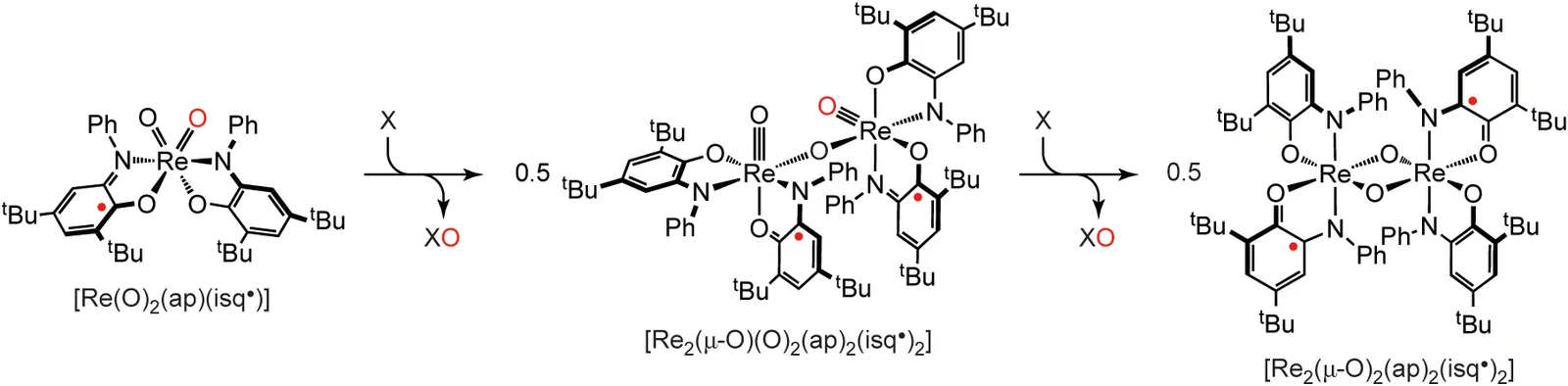
Distribution of μ-Oxo-Bridged Dimer Products Follows O-Atom Acceptor Stoichiometry

### [Re­(O)_2_(ap)­(isq^•^)] O-Atom Transfer: 4. Alkoxide Intermediates and “Adventitious” H^•^

To probe the potential intermediacy of 1e^–^ transfer products in the net O-atom transfer to Ph_3_C^•^, a reaction of the putative products of initial outer-sphere electron transfer (ET) was independently performed. Adding 1 equiv [Ph_3_C]­[BF_4_] to [Re­(O)_2_(ap)_2_]^−^ in C_6_H_6_ affords an immediate color change from navy blue to purple with a UV–vis spectrum that does not match either of the μ-oxo dimers but closely resembles the previously reported [Re­(O)­(ap)­(isq^•^)­(OC_6_Cl_5_)] alkoxide complex (Figure S9).^44^ Analysis of the solution by ESI–MS showed a molecular ion peak at 1052 *m/*z (Figure S8) consistent with formulation of the purple species as the triphenylmethoxide complex [Re­(O)­(ap)­(isq^•^)­(OCPh_3_)] (eq 3). Electrochemical measurements of *in situ* generated [Re­(O)­(ap)­(isq^•^)­(OCPh_3_)] show an irreversible reduction at E_PC_ = −0.58 V vs Fc^+/0^ in CH_3_CN (Figure S14). C_6_H_6_ solutions containing [Re­(O)­(ap)­(isq^•^)­(OCPh_3_)] are stable for days; monitoring by UV–vis showed no conversion to [Re_2_(μ-O)­(O)_2_(ap)_2_(isq^•^)_2_] over 2 days at ambient temperature. Observation of a long-lived C–O coupling product in the [Ph_3_C]^+^ + [Re­(O)_2_(ap)_2_]^−^ reaction suggested that the H^•^ required for Ph_3_COH formation from Ph_3_C^•^ + [Re­(O)_2_(ap)­(isq^•^)] did not derive from solvent, glass, or a persistent impurity.
3

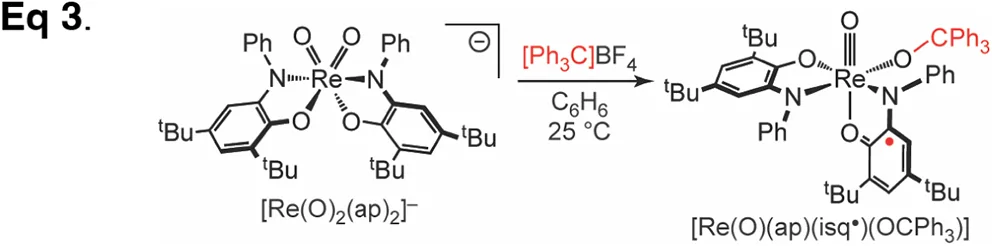




Addition of an H atom donor, in the form of 0.5 equiv DHA or 1,4-cyclohexadiene (CHD), to *in situ* generated [Re­(O)­(ap)­(isq^•^)­(OCPh_3_)] in C_6_H_6_ resulted in quantitative conversion to [Re_2_(μ-O)_2_(ap)_2_(isq^•^)_2_] by UV–vis spectroscopy (Figure S10). The observed products and reaction stoichiometry suggest [Re­(O)­(ap)­(isq^•^)­(OCPh_3_)] is a viable intermediate in the Ph_3_C^•^ oxidations by [Re­(O)_2_(ap)­(isq^•^)], but the comparatively long lifetime of [Re­(O)­(ap)­(isq^•^)­(OCPh_3_)] in only the [Ph_3_C]^+^ + [Re­(O)_2_(ap)_2_]^−^ reaction prompted us to reexamine the role of the Ph_3_C^•^ in the O-atom transfer reaction. In particular, structural similarities in the quinoid Gomberg dimer to DHA and CHD led us to postulate that it might also supply the necessary H^•^ for generation of Ph_3_COH, and Gomberg dimer is known to transfer H^•^ to peroxy radicals during radical chain autoxidation.^71^ Consistent with this hypothesis, treating [Re­(O)­(ap)­(isq^•^)­(OCPh_3_)] with 1 equiv Gomberg dimer gave clean conversion to [Re_2_(μ-O)_2_(ap)_2_(isq^•^)_2_] (eq 4), suggesting the Gomberg dimer generates Ph_3_COH by net H^•^ transfer. Accordingly, the dual roles of Gomberg dimer in its reaction with [Re­(O)_2_(ap)­(isq^•^)]—a source of Ph_3_C^•^ and a reductant to supply H^•^ to cleave the Re–OCPh_3_ bond—provides a rationale for the observed substoichiometric yields of Ph_3_COH (*vide supra*). Formation of [Re­(O)­(ap)­(isq^•^)­(OCAr_3_)] was also observed from reaction with *para*-*tert*-butyl trityl, which is incapable of supplying an H atom, demonstrating that initial C–O bond formation is viable for generation of observed Ph_3_COH (Figure S3).
4

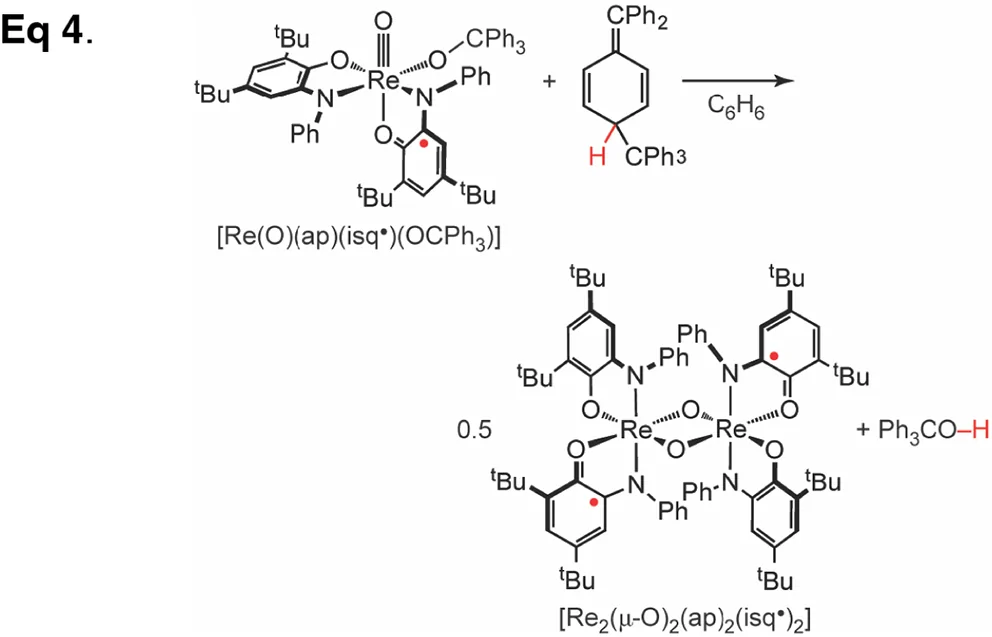




### [Re­(O)_2_(ap)­(isq^•^)] O-Atom Transfer: 5. Thermodynamics

DFT calculations (M06-L, ZORA-def2-TZVPP, D3) give a Re–O_oxo_ bond dissociation enthalpy (BDE) of 106 kcal mol^–1^ for both [Re­(O)_2_(ap)_2_]^−^ and [Re­(O)_2_(ap)­(isq^•^)]. This agrees with the primary oxo transfer reactivity collected in Figure 10a, which was previously used to bracket the BDE in [Re­(O)_2_(ap)_2_]^−^ at 118 ± 15 kcal mol^–1^.^45^ As shown in Figure 10b, [Re­(O)_2_(ap)­(isq^•^)] is cleanly reduced by O-atom acceptors with X–O bond strengths greater than 87 kcal mol^–1^ (X = Me_2_S) (Figures S11–S13), and reactions proceed in the reverse direction when the X–O bond strength is less than 72 kcal mol^–1^ (X = C_5_H_5_N).^72^ However, formation of a μ-oxo bridge provides additional driving force in incomplete oxo transfer reactions. Accordingly, the thermodynamics of O atom removal from [Re­(O)_2_(ap)­(isq^•^)] must account for the strength of the μ-oxo Re–ORe bond in [Re_2_(μ-O)­(O)_2_(ap)_2_(isq^•^)_2_]. No spectroscopic evidence for dissociation of [Re_2_(μ-O)­(O)_2_(ap)_2_(isq^•^)_2_] was seen at temperatures up to 40 °C in CH_2_Cl_2_ or C_6_H_6_ solutions, suggesting this bond is relatively robust. Hess’s law calculations of the DFT free energies of [Re_2_(μ-O)­(O)_2_(ap)_2_(isq^•^)_2_], [Re­(O)_2_(ap)­(isq^•^)], and putative [Re­(O)­(ap)_2_] give a Re–ORe bond strength of 27 kcal mol^–1^. These calculations were repeated with other standard functionals, returning an estimated range of 18–30 kcal mol^–1^ (Table S3), providing a “pseudo-error-bar” for the computed ΔG_Re–ORe_. Combined with the experimental data collected in Figure 10b, this gives a range of 107 ± 8 kcal mol^–1^ for dissociation of the Re–O_oxo_ bond in [Re­(O)_2_(ap)­(isq^•^)], consistent with, at most, modest weakening of the Re–O_oxo_ bonds in [Re­(O)_2_(ap)_2_]^−^ upon 1e^–^ oxidation to [Re­(O)_2_(ap)­(isq^•^)]. Alongside the contracted Re–O_oxo_ bond distance and increased ν­(Re≡O) frequency in the solid-state data (*vide supra*), it seems safest to assume that D­(Re–O_oxo_) in [Re­(O)_2_(ap)­(isq^•^)] is similar in bond strength to [Re­(O)_2_(ap)_2_]^−^.

**Figure 10:**
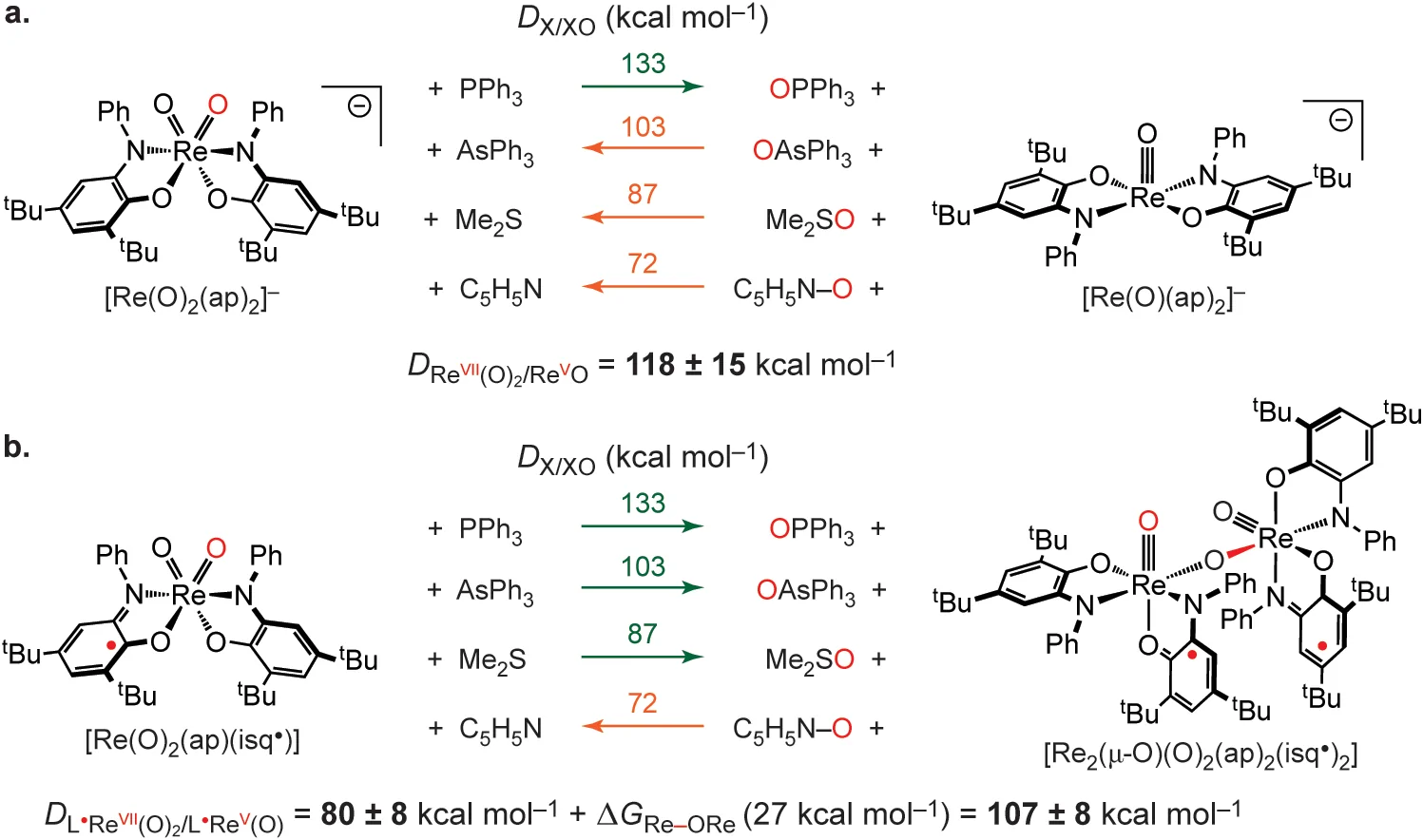
Estimated Re–O_oxo_ bond strengths in (a) [Re­(O)_2_(ap)_2_]^−^ and (b) [Re­(O)_2_(ap)­(isq^•^)] from thermodynamic oxo transfer reactivity with substrates containing known X/XO bond dissociation energies (D_X/XO_).

## Discussion

### Are [Re­(O)_2_(ap)­(isq^•^)] and [Re_2_(μ-O)­(O)_2_(ap)_2_(isq^•^)_2_] Oxyl Radicals?

Strictly, no. As outlined in the Introduction, an authentic terminal metal–oxyl complex must have an unusually low M–O π bond order and spin on oxygen. Neither of the two new Re complexes reported herein satisfy the former criterion. 1e^–^ oxidation of [Re­(O)_2_(ap)_2_]^−^ affords an S = 1/2 monomer. Because oxidation of an [O]^2–^ ligand to an [O^•^]^−^ radical formally reduces the net Re–O bond order by 0.5, significant weakening or lengthening of the Re–O_oxo_ bonds in [Re­(O)_2_(ap)­(isq^•^)] could be evidence of oxyl character. This is not observed. The Re–O_oxo_ bond distances are contracted by an average of 0.01 Å upon oxidation of [Re­(O)_2_(ap)_2_]^−^ to [Re­(O)_2_(ap)­(isq^•^)], and FTIR spectroscopy data suggest enhanced Re–O bonding in the oxidized congener. Formulation as [Re­(O)_2_(ap)­(isq^•^)] correctly emphasizes that the locus of oxidation is primarily aminophenol ligand centered. Indeed, there is no structural evidence to support formulation of the monomer as [Re­(O^•^)­(O)­(ap)_2_], and given the redox potentials of the ligands, this would be an electronically excited state.

However, the [Re­(O)_2_(ap)­(isq^•^)] formulation is an oversimplification that belies essential complexities in the electronic structure. First, in *C*_2_ point symmetry, the aminophenol-derived ligands are symmetry equivalent, and strong through-bond coupling via the Re π orbitals should mediate ligand-to-ligand charge transfer (LLCT) that makes the aminophenol ligands electronically equivalent. Indeed, the computed structure suggests the majority of the spin is distributed equally across both aminophenol ligands, equivalent to a Robin-Day Class III mixed-valence compound.^73^ Second, approximately 4% of the spin is divided across the oxo ligands in [Re­(O)_2_(ap)­(isq^•^)], consistent with delocalization of the semiquinonte [sq]^•–^ hole into the *trans*-disposed oxido [O]^2–^ ligand, as proposed for the “masked oxyl” strategy in Figure 1c. In this respect, a more straightforward, and perhaps more relevant, description invokes significant covalency in the π bonding, wherein delocalization of the unpaired spin in the ground state is a natural consequence of partial occupancy of a single MO with significant contributions from the [ap]^2–^/[isq^•^]^−^, [O]^2–^, and Re π-symmetry orbitals. Indeed, this is supported by the Re hyperfine coupling observed in the EPR spectrum despite the [Re­(O)_2_(ap)­(isq^•^)] d^0^ electron configuration. Accordingly, the nonzero spin on the oxo ligand in the ground state, along with its steric accessibility, suggests that radical coupling at oxo might be viable, as demonstrated by its reactivity with Ph_3_C^•^.

The quasi-octahedral [Re­(O)­(ap)­(isq^•^)­(μ-O)] fragments that make up the [Re_2_(μ-O)­(O)_2_(ap)_2_(isq^•^)_2_] dimer are isoelectronic and isostructural with previously reported [Re­(O)­(ap)­(isq^•^)­(X)] species (X = Cl^–^, OAr^–^).^44^ In all, the *C*_1_ symmetry of the monomeric units makes the oxo and aminophenol ligands inequivalent, and the structural data suggest the unpaired spins localize on the ligands *trans* to the terminal oxo groups. This preference likely reflects the strong *trans* influence of the oxo and the weaker Lewis basicity of the monoanionic [isq^•^]^−^ as compared to [ap]^2–^. This geometric preference also makes the [Re­(O)­(ap)­(isq^•^)­(X)] species diamagnetic. The [isq^•^]^−^ SOMO contains an in-phase combination of O and N p orbitals that are capable of mixing with π-symmetry metal d orbitals.^74^ Accordingly, a π interaction facilitates antiferromagnetic coupling between the [isq^•^]^−^ radical and the singly occupied d_*xy*_ orbital on Re, yielding an S = 0 ground state. Formulation of the μ-oxo dimer as [Re_2_(μ-O)­(O)_2_(ap)_2_(isq^•^)_2_] implies four total spins—two d^1^ Re­(VI) centers and two [isq^•^]^−^ ligands—and the μ-oxo bridge introduces additional complexity. In particular, π-donation from the μ-oxo bridge can provide a pathway for electronic communication between [Re­(O)­(ap)­(isq^•^)­(μ-O)] fragments. Such cross-bridge dπ–pπ–dπ orbital mixing has been thoroughly examined in structurally similar “blue dimer” Ru water oxidation catalysts, where the strength of the electronic coupling is highly sensitive to structural deformations about the μ-oxo bridge.^19,75^ It is not readily apparent if, or to what extent, electronic coupling between the Re centers in [Re_2_(μ-O)­(O)_2_(ap)_2_(isq^•^)_2_] is occurring. If each [Re­(O)­(ap)­(isq^•^)­(μ-O)] fragment is diamagnetic, coupling between Re centers is not a prerequisite for the observed S = 0 ground state. A complete description of the magnetism in [Re_2_(μ-O)­(O)_2_(ap)_2_(isq^•^)_2_] will require spectroscopic and magnetic measurements beyond the scope of this report.

In sum, the two new Re complexes reported herein are not oxyls, but both satisfy the masked oxyl design criteria. Differences in the symmetries and oxidation states in [Re­(O)_2_(ap)­(isq^•^)] and [Re_2_(μ-O)­(O)_2_(ap)_2_(isq^•^)_2_] lead to significantly altered ground state electronic structures and spin distributions, which in turn impact their oxidizing power. But both undergo RC-type C–O bond formation at the terminal oxo ligands in their ground electronic states. The thermodynamics and kinetics of these reactions are discussed below.

### C–O Bond Formation and Ph_3_COH Production

Three plausible mechanisms for C–O bond formation from Ph_3_C^•^ and [Re­(O)_2_(ap)­(isq^•^)] are illustrated in Scheme 3 **(a**–**c)**. Mechanism **c** of initial ET to generate [Ph_3_C]^−^ and [Re­(O)_2_(isq)_2_]^+^ is thermodynamically prohibitive, as the ET step is uphill by at least 2.5 V in CH_3_CN: no evidence for [Re­(O)­(isq^•^)_2_]^+^ is observed up to potentials >1.50 V vs Fc^+/0^; no reduction of Ph_3_C^•^ is observed to −1.05 ± 0.07 V.^76^

**Scheme 3:**
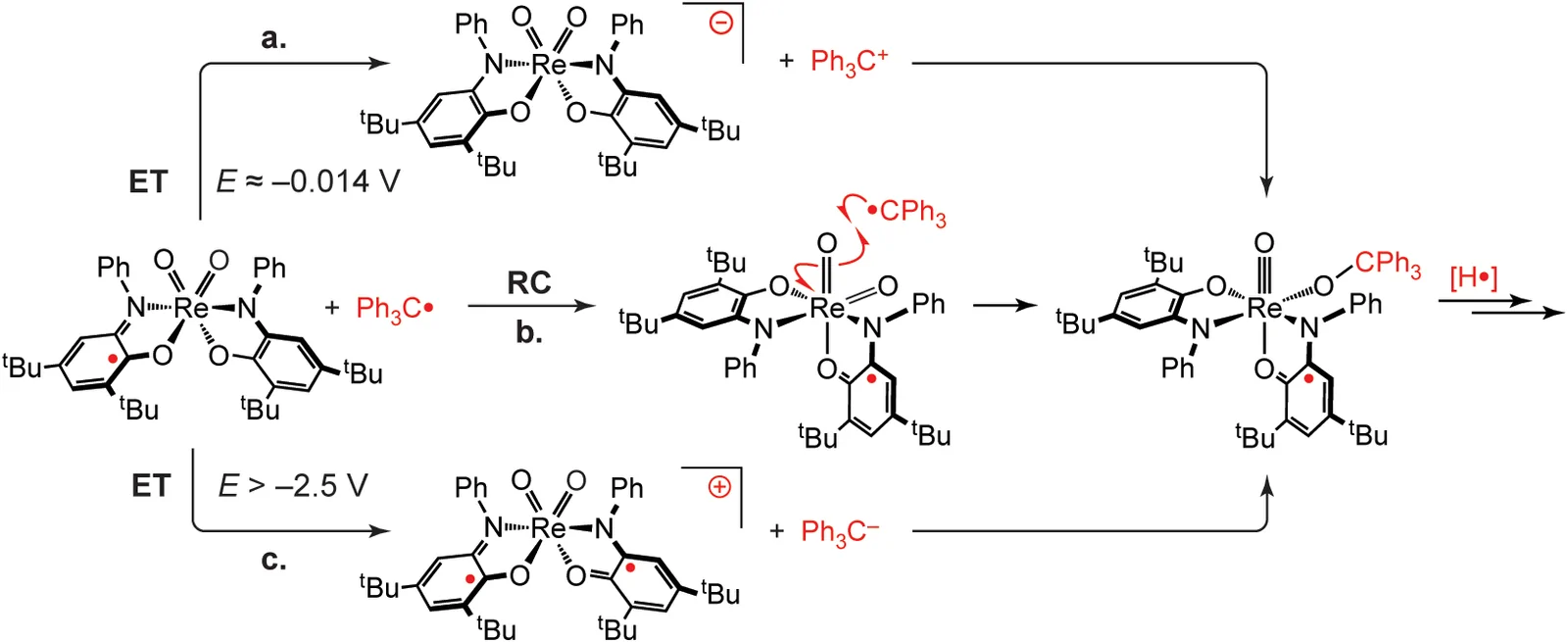
Potential Mechanisms for Generation of [Re­(O)­(ap)­(isq^•^)­(OCPh_3_)] from Ph_3_C^•^ and [Re­(O)_2_(ap)­(isq^•^)]

Mechanism **a** of initial ET to generate [Ph_3_C]^+^ and [Re­(O)_2_(ap)_2_]^−^ is uphill by only ∼14 mV in CH_3_CN. Facile C–O bond formation from those ionic intermediates was independently demonstrated in the reaction of isolated [Ph_3_C]^+^ with [Re­(O)_2_(ap)_2_]^−^. In this context, the solvent dependence on the rates of [Re_2_(μ-O)­(O)_2_(ap)_2_(isq^•^)_2_] formation from Ph_3_C^•^ and [Re­(O)_2_(ap)­(isq^•^)] might reflect differences in the rates of the initial ET step. The comparatively fast reaction in CH_3_CN vs C_6_H_6_ is expected based on stabilization of the charged ET products in polar solvent. This is evidenced in the DFT computations, where formation of the ion pair in C_6_H_6_ is disfavored by +31 kcal mol^–1^. Taken at face value, this result rules out a mechanism of initial rate-determining ET in C_6_H_6_, as it implies a ∼187 yr half-life at ambient temperature. Additionally, mixtures of isolated [Ph_3_C]^+^ and [Re­(O)_2_(ap)_2_]^−^ do not give back ET to regenerate Ph_3_C^•^ + [Re­(O)_2_(ap)­(isq^•^)] in C_6_H_6_, which would be readily apparent by UV–vis spectroscopy, despite the reaction being exoergic by the same 31 kcal mol^–1^. Accordingly, the observation of fast, quantitative conversion to [Re­(O)­(ap)­(isq^•^)­(OCPh_3_)] from [Ph_3_C]^+^ + [Re­(O)_2_(ap)_2_]^−^ in C_6_H_6_ would require the Lewis acid–base C–O coupling reaction to occur at a rate at least 100× faster than back ET. Whereas the former has steric and orientational requirements and extensive heavy atom motion, a simple Marcus treatment would assume the exoergic outer-sphere back ET occurs with a frequency factor of approximately unity. A large kinetic barrier for the ET step in mechanism **a** would require slow ET self-exchange from at least one of the [Ph_3_C]^+^/Ph_3_C^•^ or [Re­(O)_2_(ap)­(isq^•^)]/[Re­(O)_2_(ap)_2_]^−^ redox pairs. These were not measured, but the electrochemical data do not suggest either that redox interconversion is atypically slow or occurs with significant inner-sphere reorganization. The sum of the data suggests that mechanism **a** is likely the dominant mechanism, particularly in CH_3_CN. The case for slow, rate-determining ET in C_6_H_6_ is less clear-cut, but it cannot be ruled out without experimental data for the thermodynamics and kinetics of back ET, which are beyond the scope of this report.

This analysis leaves the RC direct attack **b** as a plausible mechanism for C–O bond formation from Ph_3_C^•^ and [Re­(O)_2_(ap)­(isq^•^)]. Although radical additions to an oxo ligand do not require unpaired spin at oxygen, radical trapping is a hallmark of Ph_3_C^•^ reactivity, as exemplified by its well-known reactions with O_2_ and I_2_. O atom transfer to Ph_3_C^•^ has, to our knowledge, little precedent in the extensive literature for transition metal oxo-transfer reactions, but recent reports from our group and others of trityl C–O coupling have been suggested to be an indicator of oxyl radical character in the metal–oxido unit.^42–44,77,78^ The extent to which RC-type C–O bond formation here reflects radical density at the oxo ligand is discussed below.

We previously attributed the apparent instability of the Re–OCPh_3_ alkoxide linkage to unfavorable sterics and/or “adventitious H^•^”.^44^ But the observation of long-lived [Re­(O)­(ap)­(isq^•^)­(OCAr_3_)] species implies the Re–OCAr_3_ bond is not inherently unstable, and Re–OCPh_3_ homolysis can be ruled out by the absence of rearrangement products deriving from triphenylmethoxyl Ph_3_CO^•^ radical.^70^ Accordingly, Ph_3_COH production from [Re­(O)_2_(ap)­(isq^•^)] + Ph_3_C^•^ requires that H^•^ addition occurs prior to, or concomitant with, Re–O bond cleavage. In this respect, the absence of observable [Re­(O)­(ap)­(isq^•^)­(OCPh_3_)] in reactions with Ph_3_C^•^ reflects the capacity of the Gomberg dimer to rapidly deliver H^•^ to an intact Re–OCPh_3_ bond following rate-limiting radical C–O bond formation. Generation of Ph_3_COH via treatment of independently prepared [Re­(O)­(ap)­(isq^•^)­(OCPh_3_)] with other H^•^ sources demonstrates that the proposed two-step mechanism is a viable route to Ph_3_COH. How the H atom is delivered, however, is unknown. Addition of H^•^ to the oxo in [Re­(O)­(ap)­(isq^•^)­(OCPh_3_)] to generate a Re­(OCPh_3_)­(OH)] complex followed by elimination via a four-center transition state is plausible, but the analogous reactivity of a previously reported Re­(OCPh_3_)Cl complex,^44^ and Ph_3_C^•^ hydroxylation by [Re_2_(μ-O)­(O)_2_(ap)_2_(isq^•^)_2_], suggests that an intramolecular pathway is not a prerequisite for the observed reactivity.

The direct attack RC mechanism **b** resembles the canonical “hydroxyl rebound” mechanism for C–H hydroxylation,^1^ with two key differences: (i) the order of the bond forming steps is reversed, so radical O–C bond formation precedes formation of the O–H bond; (ii) the R^•^ and H^•^ for O–R and O–H bond formation derive from separate reagents rather than a single C–H bond. In this context, the capacity of [Re­(O)_2_(ap)­(isq^•^)] to engage in classic rebound-type hydroxylation merits additional consideration. Initial H^•^ transfer to form an unobserved [Re­(O)­(ap)­(isq^•^)­(OH)] transient cannot be ruled out, but independent experiments to probe H^•^ transfer to [Re­(O)_2_(ap)­(isq^•^)] gave significant quantities of H_2_O without any observed intermediates, suggesting addition of a second H^•^ to [Re­(O)­(ap)­(isq^•^)­(OH)] is fast. Accordingly, for Ph_3_COH to form by initial H^•^ addition at [Re­(O)_2_(ap)­(isq^•^)] the putative [Re­(O)­(ap)­(isq^•^)­(OH)] intermediate would have to be rapidly intercepted by Ph_3_C^•^, which is in low concentration relative to the Gomberg dimer that is the source of H^•^. Furthermore, [Re­(O)_2_(ap)­(isq^•^)] shows little apparent propensity for O atom transfer to weak benzylic C–H bonds. For instance, [Re­(O)_2_(ap)­(isq^•^)] is indefinitely stable in toluene up to 45 °C. Finally, observation of [Re­(O)­(ap)­(isq^•^)­(OCAr_3_)] from reaction with a para-functionalized trityl derivative that is incapable of supplying an H atom confirms that preactivation by delivery of H^•^ is not necessary for radical C–O bond formation.

O-atom transfer from [Re­(O)_2_(ap)­(isq^•^)] or [Re_2_(μ-O)­(O)_2_(ap)_2_(isq^•^)_2_] to Ph_3_C^•^ functionally mimics our prior report of Ph_3_COH formation from Ph_3_C^•^ + [Re­(O)­(ap)­(isq^•^)­Cl],^44^ suggesting that radical Ph_3_C^•^ trapping is a general feature of this class of complexes. Observation of an alkoxide [Re­(O)­(ap)­(isq^•^)­(OCPh_3_)] intermediate here provides direct evidence for R^•^ trapping at a formally closed-shell oxido ligand. The role of [isq^•^]^−^ unpaired spin in bringing about this RC-type reactivity is discussed below.

### Thermodynamic vs Kinetic Effects on O-Atom Transfer

[Re­(O)_2_(ap)­(isq^•^)] hydroxylates trityl Ph_3_C^•^, whereas [Re­(O)_2_(ap)_2_]^−^ does not. Potential thermodynamic and kinetic contributors to this disparity are considered in turn.

[Re­(O)_2_(ap)­(isq^•^)] is necessarily a stronger 1e^–^ oxidant than [Re­(O)_2_(ap)_2_]^−^—it is obtained by oxidation of d^0^ metal complex—but neither is a powerful outer-sphere oxidant. [Re­(O)_2_(ap)­(isq^•^)] is produced at a potential −125 mV below Fc^+^ in CH_3_CN. The stability of [Re­(O)_2_(ap)­(isq^•^)] in solvents with relatively weak benzylic C–H bonds (cf. toluene) suggests the H-atom accepting power of [Re­(O)_2_(ap)­(isq^•^)] is modest at best, implying [Re­(O)_2_(ap)_2_]^−^ is also a weak base. Neither are thermodynamically strong O-atom donors. Both are reduced by PPh_3_. [Re­(O)_2_(ap)­(isq^•^)] transfers an O atom to thermodynamically weaker oxo acceptors, including AsPh_3_ and Me_2_S, but formation of the μ-oxo bridge in [Re_2_(μ-O)­(O)_2_(ap)_2_(isq^•^)_2_] provides additional driving force for the net O-atom transfer. Accordingly, per Figure 10, the intrinsic O-atom donor capacities of [Re­(O)_2_(ap)­(isq^•^)] and [Re­(O)_2_(ap)_2_ are the same within experimental error. DFT in fact suggests that the Re–O_oxo_ BDEs are identical.

An alternative estimate of the thermodynamic differences in Ph_3_C^•^ trapping by [Re­(O)_2_(ap)­(isq^•^)] vs [Re­(O)_2_(ap)_2_]^−^ can be obtained electrochemically, as summarized in Scheme 4. [Re­(O)­(ap)­(isq^•^)­(OCPh_3_)] is reduced at −0.58 V vs Fc^+/0^ in CH_3_CN. Accordingly, Ph_3_C^•^ trapping by [Re­(O)_2_(ap)­(isq^•^)] is preferred by 10 kcal mol^–1^ over Ph_3_C^•^ addition to Re­(O)_2_(ap)_2_]^−^. Whether the observed differences in reactivity have more to do with the barrier than the driving force is not settled by this observation, but the neutral species is indisputably a thermodynamically stronger oxidant toward Ph_3_C^•^.

**Scheme 4:**
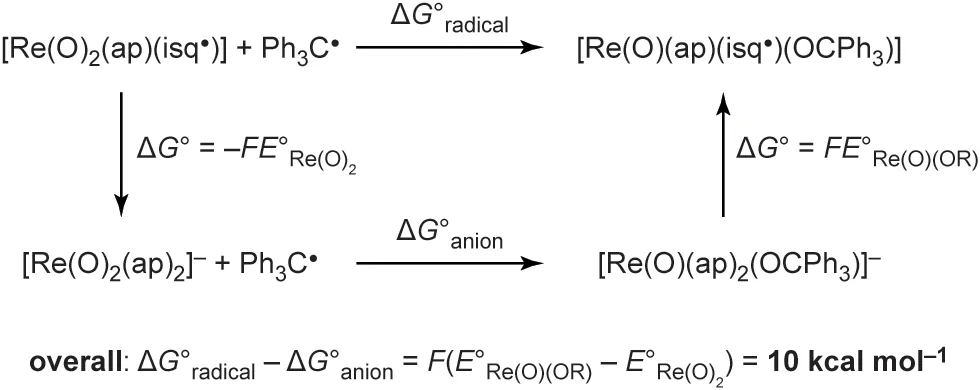
ΔΔ*G*° for Radical Ph_3_C^•^ Trapping by [Re­(O)_2_(ap)­(isq^•^)] vs. [Re­(O)_2_(ap)_2_]^−^

Despite its widespread use as a trap for stable radicals, Ph_3_C^•^ rarely appears in the extensive literature for metal-mediated oxo transfer reactions. The capacity of Gomberg’s dimer to also supply the H^•^ necessary for formation of Ph_3_COH, described herein, makes Ph_3_C^•^ a reasonably strong O atom acceptor. We posit, therefore, that the scarcity of metal-mediated interconversions of Ph_3_C^•^/Ph_3_CO^•^ reflects a kinetic rather than thermodynamic bias. The reactivity of Ph_3_C^•^ with O_2_ is typically ascribed to facile bond formation between open-shell radicals, for instance via chain autoxidation. It is tempting to draw parallels between such RC-type C–O bond forming reactions and trityl trapping at the oxo ligands in the [Re­(O)_2_(ap)­(isq^•^)] and [Re­(O)­(ap)­(isq^•^)­(X)] masked oxyl complexes. Our initial attempt to correlate the effects of radical distribution on the coupling reactivity were challenged by the fact that the [Re­(O)­(ap)­(isq^•^)­(X)] complexes are not free radicals.^44^ Although they are appropriately described as diradicals, with 1e^–^ in a rhenium d orbital and 1e^–^ on a redox-active [isq]^•–^ ligand, the complexes are S = 0 in solution ambient temperatures because the metal and ligand electrons are antiferromagnetically coupled. It has been argued that the experimental data are also consistent with a closed-shell Re^VII^(O)­(ap)_2_X formulation, wherein the observed bonding patterns arise from strong [ap^Ph^]^2–^ ligand-to-metal π donation.^47^ There is no reason to dismiss *a priori* the possibility that weakly coupled spins in spatially distinct regions of a given molecule might lead to different reactivity than a closed shell molecule with contiguous doubly occupied frontier orbitals, but this is difficult to assess experimentally.

We reasoned that oxidation of a d^0^ oxometal complex would address this ambiguity. The S = 1/2 [Re­(O)_2_(ap)­(isq^•^)] contains a single ligand-centered unpaired electron, and qualitatively, [Re­(O)_2_(ap)­(isq^•^)] is significantly more reactive than the S = 0 masked oxyls. It cleanly converts Ph_3_C^•^ to Ph_3_COH, even at very low effective concentrations of Ph_3_C^•^, and stoichiometric reactions of Ph_3_C^•^ with [Re­(O)_2_(ap)­(isq^•^)] are complete in hours at 25 °C vs ∼1 week for [Re­(O)­(ap)­(isq^•^)­Cl]. The accelerated degradation reactivity in ambient or low-energy light (*vide supra*), and enhanced stability in the dark, hints at enhanced reactivity from a low-energy excited-state. But the observation of dark reactivity with Ph_3_C^•^ implies the [Re­(O)_2_(ap)­(isq^•^)] radical is kinetically reactive without the additional driving force afforded by photolysis or thermal access to an excited state.

As discussed above, an ET mechanism for the reaction of [Re­(O)_2_(ap)­(isq^•^)] with Ph_3_C^•^ cannot be ruled out by the experimental data herein. Direct RC attack is, at best, slow in C_6_H_6_ and likely outcompeted by faster ET in polar solvents, if not in all solvents. But in aggregate, a compelling trend emerges. Facile Ph_3_C^•^ trapping has been observed at the oxo ligands in [Re­(O)_2_(ap)­(isq^•^)], [Re­(O)­(ap)­(isq^•^)­Cl], and [Re_2_(μ-O)­(O)_2_(ap)_2_(isq^•^)_2_]. [Re­(O)_2_(ap)_2_]^−^ and [Re­(O)­(ap)_2_]^−^ are inert to Ph_3_C^•^. The former series have different oxidation states, coordination geometries, oxidizing potentials, and steric profiles, but each contains at least one [isq^•^] ligand radical and satisfies the masked oxyl design criteria; the latter do not. We emphasize that a thermodynamic basis for this disparate reactivity cannot be ruled out. But we find it plausible, if not likely, that RC-type C–O bond formation at [Re­(O)_2_(ap)­(isq^•^)] reflects spin in the ground state, which permits the net 2e^–^ oxo transfer to Ph_3_C^•^ to occur via kinetically facile radical steps.

## Conclusions

Prior efforts to correlate the effects of radical spin density on reactivity in monomeric [Re­(O)­(ap)­(isq^•^)­(X)] complexes were challenged by the absence of unpaired electrons in the S = 0 ground states. And coordinatively unsaturated S = 1/2 masked oxyl monomers, such as five-coordinate [Re­(O)­(ap)­(isq^•^)], were compromised by radical additions at the Re centers.^44^ Isolation of [Re­(O)_2_(ap)­(isq^•^)] from [Re­(O)_2_(ap)_2_]^−^ addresses both of those issues and provides an isostructural comparison of the effects of unpaired spin on RC-type reactivity at a terminal oxo ligand. Nothing in the solid state structural or spectroscopic data suggests that the Re–O_oxo_ bonds in [Re­(O)_2_(ap)­(isq^•^)] are thermodynamically activated for O atom transfer, and the experimental and computational data suggest that the intrinsic O atom donor abilities of [Re­(O)­(ap)­(isq^•^)] [Re­(O)_2_(ap)_2_]^−^ are comparable. Rather, the propensity of [Re­(O)­(ap)­(isq^•^)] to undergo direct C–O bond formation with Ph_3_C^•^ is apparently kinetic in origin.

Unpaired spin at oxygen is not a prerequisite for radical addition to an oxido ligand, but a growing body of work suggests that radical density at a ligand gives access to kinetically facile bond-making steps that are inaccessible at closed-shell ligands. In this context, it is often difficult to parse the thermodynamic and kinetic contributions to the radical reactivity. For instance, excited state oxyl radicals such as the Ti^III^–O^•^ transients generated by photoexcitation of titanosilicates have the thermodynamic bias baked-in vs their closed-shell Ti^IV^=O precursors.^29^ Notably, the radical additions described here occur in the dark. The sum of the data makes a compelling case that this reactivity arises from radical character in the ground electronic state, wherein facile intraligand communication through the π-symmetry molecular orbitals imparts oxyl character to a closed-shell oxide ligand. This report adds two additional oxorhenium species that satisfy the masked oxyl design criteria. All the masked rhenium oxyls with [isq^•^]^−^ ligand radicals are prone to form C–O bonds by Ph_3_C^•^ capture. Structural analogs without the ligand radical are inert. Accordingly, the masked oxyl design principles are transferrable to entirely different classes of redox-active ligand complexes, opening avenues to pursue thermodynamically stable oxidants that are kinetically activated for selective odd-electron bond-making and -breaking redox reactions. Successes in utilizing these principles for aerobic oxidation catalysis will be the subject of a future report.

## Experimental Details

### General Considerations

All manipulations were performed under anaerobic conditions using standard vacuum-line techniques or in an inert-atmosphere glovebox under purified nitrogen unless specified otherwise. Routine NMR spectra were acquired on a Bruker Avance III spectrometer (500 MHz for ^1^H) equipped with a Prodigy Cryoprobe at ambient temperature. All chemical shifts are reported in parts per million (ppm) relative to TMS, with the residual solvent peak serving as an internal reference.^79^ Solution magnetic moments were obtained by Evan’s NMR method,^46,80^ and are reported as the average of three independent measurements, unless otherwise specified. UV–visible absorption spectra were acquired using a Varian Cary 50 spectrophotometer. Unless otherwise noted, all electronic absorption spectra were recorded at 25 °C in 1 cm quartz cells. IR spectra were obtained by attenuated total reflectance (ATR) through a diamond plate on a Bruker Optics Alpha-P Fourier transform infrared (FTIR) spectrometer. All mass spectra were recorded in the Georgia Institute of Technology Bioanalytical Mass Spectrometry Facility. Matrix-assisted laser desorption/ionization mass spectra (MALDI–MS) were obtained using an Applied Biosystems 4700 proteomics analyzer. Electrospray ionization mass spectrometry (ESI–MS) was carried out with acetonitrile solutions using a Micromass Quattro LC spectrometer. Electron impact mass spectra (EI–MS) were obtained using a VG instruments model 70-SE spectrometer. Gas chromatography mass spectrometry (GC–MS) analyses used an Agilent 6890 gas chromatograph equipped with an autosampler and a Restek Rxi-5 ms column (30 m × 0.25 mm i.d., 0.25 μm film thickness). Injections of 1 μL were made at a 50:1 split ratio. The GC oven program consisted of a hold at 30 °C for 1 min, followed by a 15 °C/min ramp to 300 °C, and then a hold at 300 °C for 11 min. The mass spectrometer used in tandem was a Micromass AutoSpec electronionization (EI) detector. For GC–MS quantification of organics, *n*-decane (1 μL) was added to serve as an internal standard and product yields were determined by a comparison of the EI–MS detector responses against calibration curves derived from the pure materials, as well as by a comparison to the detector response for *n*-decane. Gas chromatography with flame ionization detection (GC–FID) analyses were performed using a Shimadzu GC-2010 Gas Chromatograph equipped with Restek SHRXI-5MS column (15 m × 0.25 mm i.d., 0.25 μm film thickness). Injections of 1 μL were made at a 1:30 split ratio with the nitrogen carrier gas flow rate of 24.4 mL/min. Conditions of the GC are as follows: injector temperature, 350 °C; column starting temperature, 40 °C; column ramp, 250 °C at 25 °C/min; final column hold, 250 °C for 2 min. Cyclic voltammetry experiments were performed inside an N_2_-filled glovebox in MeCN or CH_2_Cl_2_ with 0.1 M [^n^Bu_4_N]­[PF_6_] as the supporting electrolyte, unless otherwise noted. The voltammograms were recorded with a CH Instruments 620C potentiostat, using a 2.5 mm (O.D) 1.0 mm (I.D.) Pt disk working electrode, Ag wire quasi-reference electrode, and a Pt wire auxiliary electrode, at a scan rate of 0.1 V s^–1^, unless reported otherwise. Reported potentials are referenced to the ferrocenium/ferrocene (Fc^+^/Fc) redox couple, added as an internal standard at the conclusion of each experiment. Elemental analyses were performed by Atlantic Microlab, Inc., Norcross, GA. All analyses were performed in duplicate, and the reported compositions are the average of the two runs. Full details of X-ray data collection and refinement are provided in the Supporting Information.

**Safety.** No uncommon hazards are noted.

### Synthesis of (Et_4_N)­[Re­(^18/16^O)_2_(ap)_2_]

A 100 mL round-bottom flask was charged with (Et_4_N)­[Re^V^(O)­(ap^Ph^)_2_] (120 mg, 0.130 mmol) and 40 mL of dry, degassed MeCN. ^18^OH_2_ (200 mL) was added and stirred vigorously for 2 days. The solution was exposed to air with continued stirring, and a color change from moss green to navy blue was observed over 24–48 h at ambient temperature. Concentration of the solution *in vacuo* afforded a dark blue precipitate, which was collected via filtration and dried to afford a mixture of (Et_4_N)­[Re^VII^(^16^O)_2_(ap^Ph^)_2_], (Et_4_N)­[Re^VII^(^18^O)­(^16^O)_2_(ap^Ph^)_2_] and (Et_4_N)­[Re^VII^(^18^O)_2_(ap^Ph^)_2_] (0.029 mg, 0.031 mmol, 24%). ESI–MS (*m/z):* 811 [M]^−^.

### Synthesis of Re^VII^(O)_2_(ap^Ph^)­(isq^Ph^)

A 20 mL scintillation vial was charged with (Et_4_N)­[Re^VII^(O)_2_(ap^Ph^)_2_] (40 mg, 0.043 mmol) and 3 mL MeCN and stirred vigorously. Dropwise addition of 0.031 M ferricenium hexafluorophosphate in MeCN (1.5 mL, 0.047 mmol) over 1 min gave a color change from navy blue to dark green, followed by precipitation of a dark solid over 1 h. The solution was concentrated *in vacuo* and filtered to afford Re^VII^(O)_2_(ap^Ph^)­(isq^Ph^) (30 mg, 0.037 mmol, 84%) as a dark green waxy solid. Oxidations performed with nitrosonium tetrafluoroborate or silver tetrafluoroborate gave the same product; ferricenium was found to give the best yield. Crystalline solids suitable for single crystal X-ray diffraction were obtained by slow diffusion of pentane into a saturated toluene solution at −20 °C. MALDI–MS (*m*/*z*): 809 [M]^+^. UV–vis (C_6_H_6_) λ_max_, nm (ε, M^–1^ cm^–1^): 294 (16000), 410 (8500), 596 (6000), 846 (2000). FTIR (ATR, cm^–1^): 2949 (m, b), 1487 (w), 1360 (w), 1251­(m), 1148 (m), 1102 (m), 10227 (m), 911 (vs), 876 (vs), 773 (m), 739 (m), 703 (s), 651 (m), 582 (m), 493 (m). Anal. Calcd for C_40_H_50_N_2_O_4_Re: C, 59.38; H, 6.23; N, 3.46. Found: C, 59.31; H, 6.15; N, 3.52.

### Synthesis of Re^VI^_2_(μ-O)­(O)_2_(ap^Ph^)_2_(isq^Ph^)_2_. **Method 1**

A 20 mL scintillation vial was charged with (Et_4_N)­[Re^VII^(O)_2_(ap^Ph^)_2_] (0.227 g, 0.242 mmol) and AgBF_4_ (47 mg, 0.24 mmol). Addition of acetone (10 mL) afforded a dark purple solution. The reaction mixture was stirred for 15 h and the solvent was removed *in vacuo* to give a dark residue, which was extracted into ether and filtered to remove a white precipitate. The purple filtrate was dried *in vacuo* to give Re^VI^_2_(μ-O)­(O)_2_(ap^Ph^)_2_(isq^Ph^)_2_ (0.165 g, 0.103 mmol, 43%) as a purple powder. Single crystals for X-ray diffraction were obtained by slow evaporation of a CH_3_CN solution at −20 °C. **Method 2.** A 10 mL scintillation vial was charged with (Et_4_N)­[Re^VII^(O)_2_(ap^Ph^)_2_] (100 mg, 0.106 mmol) and THF (2 mL) to afford a clear, dark blue solution. Addition of PPh_3_ (14 mg, 0.053 mmol) in THF (1 mL) followed by dropwise addition of AgBF_4_ (21 mg, 0.11 mmol) in THF (1 mL) and stirring for 15 h at ambient temperature yielded a turbid, darker purple solution. Filtration through a fritted funnel to remove Ag^0^ and removal of the solvent gave a dark residue, which was taken up in pentane and passed through a 0.2 μm PTFE syringe filter to remove OPPh_3_ solids. The filtrate was dried *in vacuo* to give a purple powder of Re^VI^_2_(μ-O)­(O)_2_(ap^Ph^)_2_(isq^Ph^)_2_ (80.1 mg, 0.0571 mmol, 94%). MALDI-MS (*m/*z): 1602 [M]^+^. UV–vis (C_6_H_6_) λ_max_, nm (ε, M^–1^ cm^–1^): 292 (25000), 430 (19000), 522 (19000), 792 (10000). FTIR (ATR, cm^–1^): 2951 (m, b), 1589 (v), 1483 (v), 1361 (v), 1239 (m), 1166 (m), 1119 (m), 996 (v), 910 (m), 753 (s), 721 (vs), 704 (vs), 623 (s), 540 (vs), 404 (m). Samples for combustion analysis were prepared by method 2, which produces OPPh_3_ as a byproduct that can be carried through the purification steps. The reported analysis is for Re^VI^_2_(μ-O)­(O)_2_(ap^Ph^)_2_(isq^Ph^)_2_•0.9OPPh_3_. The presence of the OPPh_3_ in the sample was confirmed by its diagnostic resonances in the ^1^H NMR spectrum. Anal. Calcd for C_96.2_H_113.5_N_4_O_7.9_P_0.9_Re_2_: C, 62.37; H, 6.18; N, 3.02; Found: C, 62.10; H, 6.45; N, 2.66.

### Reactions with Triphenylmethyl Radical

In a representative procedure, a C_6_H_6_ solution of 0.0247 M Re^VII^(O)_2_(ap^Ph^)­(isq^Ph^) (2.00 mL, 0.0494 mmol) was mixed with 0.0933 M triphenylmethyl radical dimer in C_6_H_6_ (267 μL, 0.0247 mmol Ph_3_C^•^) in a thick-walled flask equipped with a Kontes brand high-vacuum PTFE valve. The flask was sealed and stirred vigorously for 1 day. A 0.2 mL aliquot of the reaction solution was transferred to a 2 mL GC–MS glass vial under nitrogen, and 0.00513 M *n-*decane in benzene (5 μL, 2.6 × 10^–5^ mmol) was added as an internal standard. A 1 μL aliquot of this solution was then sampled by GC–FID and the Ph_3_COH yield (0.0170 mmol, 69%) was determined by a comparison of the FID responses against calibration curves derived from the pure materials, as well as by comparison to the detector response for a control solution of Gomberg’s dimer.

### Electron Paramagnetic Resonance Spectroscopy

Continuous-wave X-band spectra were collected at 77 K on a Bruker ELEXSYS-II E500 spectrometer operating in the X-band. Samples were prepared inside of an inert atmosphere glovebox (Vigor) under a dinitrogen atmosphere (<0.1 ppm of O_2_/H_2_O). Toluene was sparged with UHP-grade argon (Airgas), passed through columns containing Q-5 and molecular sieves in a solvent purification system (JC Meyer Solvent Systems), and stored in a bottle over 3 Å molecular sieves in the glovebox. Samples were prepared by first making a toluene solution of the compound at a known concentration and loading a standard 4 mm clear-fused quartz EPR tube with a small amount of the solution. The tube was then sealed with a Swagelok UltraTorr adapter, removed from the glovebox and cycled onto a Schlenk line. The solution was then frozen in a liquid nitrogen bath and the quartz tube was sealed under vacuum using a H_2_/O_2_ torch. As the sample was mildly light sensitive, the sample preparation and sealing process was carried out either in the dark, or with the sample covered in aluminum foil. Spectra were collected of the frozen toluene glass at 77 K from 3000 to 3600 G with a modulation field of 2 G and a modulation frequency of 100 kHz. Microwave attenuation was set to 19 dB with a receiver gain of 75 dB.

Spectra were fit using EasySpin 6.0.6 ^81^ as implemented in the MatLab (MathWorks) toolbox. Least-squares fittings were performed using the *esfit_legacy* GUI function and the *pepper* simulation function utilizing the Nelder–Mead simplex algorithm. The spectrum was best fit using an effective spin-1/2 Hamiltonian accounting for electronic and nuclear Zeeman terms, and a hyperfine interaction to natural abundance Re (^185^Re: 37.4%, *I* = 5/2; ^187^Re: 62.6%, *I* = 5/2):

$${Ĥ}_{spin}=\mu_{B}\mathrm{B⃗}\cdot \mathrm{g̃}\cdot Ŝ-\mu_{n}g_{Re}\mathrm{B⃗}\cdot {Î}_{Re}+Ŝ\cdot {Ã}_{Re}\cdot {Î}_{Re}$$

Where *μ_B_* is the Bohr magneton, B⃗ is the magnetic field vector, *g̃* is the electronic g-tensor, *Ŝ* is the electronic spin vector, *μ_n_* is the nuclear magneton, *g_Re_* is the nuclear gyromagnetic ratio (^185^Re = 1.2748, ^187^Re = 1.2879), *Î_Re_* is the nuclear spin vector for Re, and *Ã_Re_* is the hyperfine tensor for Re. To simplify this analysis, any nuclear quadrupolar interactions are neglected and *g̃* and *Ã_Re_* are presumed to be colinear along the principal axes. Spectral line-broadening was applied via isotropic H-strain (*σ_H_*) accounting for unresolvable hyperfine interactions with other spin-active nuclei in the compound. The use of phenomenological line broadenings via isotropic Gaussian and/or Lorentzian components was unable to provide a reasonable fit of the data. The best fit of the data was afforded with g_⊥_ = 2.00814(9), g_∥_ = 2.00464(15), A_⊥_(Re) = 36.57(14) MHz, A_∥_(Re) = 138.8(4) MHz, and σ_*H*_ = 73.6(6) MHz. Values presented in the main text are rounded to the thousandths place for g values and integer values for the hyperfine components.

### Computational Studies

All DFT calculations were performed in ORCA 6.0.1 ^54–59^ using the M06-L functional,^60^ D3,^61^ ZORA,^62–64^ SARC-ZORA-TZVPP^67^ basis set on Re, and ZORA-def2-TZVPP^62–66^ basis set on all other atoms. Solvation effects were modeled with CPCM.^68^ EPR parameters were calculated using coupled perturbed Kohn–Sham theory.^82–85^ Additional calculations in the Supporting Information were performed with the BP86,^86,87^ BLYP,^86,88^ B3LYP,^88,89–90^ PBE,^91^ PBE0,^92^ and TPSS^93^ functionals.

## Supplementary Material



## Notes

Kinetics data for degradation of [Re­(O)_2_(ap)­(isq^•^)]; NMR, UV–vis, and MS data for selected Re complexes are reaction products; complete X-ray structure reports for Re^VII^(O)_2_(ap^Ph^)­(isq^Ph^) and Re^VI^_2_(μ-O)­(O)_2_(ap^Ph^)_2_(isq^Ph^)_2_; spin-density plot of [Re­(O)_2_(ap)­(isq^•^)]; DFT-simulated EPR parameters; Cartesian coordinates (XYZ format) of DFT-optimized geometries (PDF)

## #

Dr. Kenneth Hardcastle passed away on Dec 8, 2018.
