# Supplementary material for: Radical Control in O‑Atom Transfer from an Unusually Oxidized Oxorhenium Complex
Source: Inorg Chem. 2026 Sep 1;65(36):20769–85. doi: 10.1021/acs.inorgchem.5c05970 (PMC13580191; doi:10.1021/acs.inorgchem.5c05970)
Supplement: Supplementary file 1 [file ic5c05970_si_001.pdf]

Supporting Information for:

Radical Control in O-atom Transfer from an Unusually Oxidized Oxorhenium Complex

Jennifer A. Hill,[a] Andrew G. Hill,[a] Sulaiman Somani,[a] Ashley M. McDaniel,[a] Cameron A. Lippert,[a] Grant R. Wilkinson,[a] Kenneth I. Hardcastle,[b] John Bacsa,[a,b] and Jake D. Soper*,[a]

[a]School of Chemistry and Biochemistry, Georgia Institute of Technology, Atlanta, GA, 30332-0400, United States

[b]X-ray Crystallography Center, Department of Chemistry, Emory University, 1515 Dickey Drive, Atlanta, GA, 30322, United States

Email: jake.soper@gatech.edu

Table of Contents

**Table S1**	Order determination for degradation of [Re(O)$_2$(ap)(isq$^\bullet$)] to [Re$_2$(μ-O)(O)$_2$(ap)$_2$(isq$^\bullet$)$_2$].	**S5**
**DFT Density Plots**		**S5**
**Figure S1**	Spin-density plot of [Re(O)$_2$(ap)(isq$^\bullet$)] and Lowdin spin population per atom	**S5**
**NMR Data**		**S6**
**Figure S2**	^{1}H NMR spectrum (300 MHz, CD_2Cl_2) of [Re$_2$(μ-O)(O)$_2$(ap)$_2$(isq$^\bullet$)$_2$]	**S6**
**Figure S3**	^{1}H NMR spectrum (500 MHz, C_6D_6) of the reaction products of [Re(O)$_2$(ap)(isq$^\bullet$)] and 9,10-dihydroanthracene.	**S7**
**Mass Spectrometry Data**		**S8**
**Figure S4**	Mass spectrum of $OPPh_3$ from GC-MS of reaction products of [(ap)(isq$^\bullet$)Re(O)$_2$] and PPh_3.	**S8**
**Figure S5**	Mass spectrum of $HOCPh_3$ from GC-MS of the reaction products of [Re(O)$_2$(ap)(isq$^\bullet$)] and trityl radical.	**S8**
**Figure S6**	Mass spectrum of benzophenone from GC-MS of the reaction products of [Re(O)$_2$(ap)(isq$^\bullet$)] and trityl radical.	**S9**
**Figure S7**	$HCPh_3$ from GC-MS of the reaction products of [Re(O)$_2$(ap)(isq$^\bullet$)] and trityl radical.	**S9**

**Figure S8**	MALDI-MS of [(ap)(isq•)Re(O)(OCPh$_3$)] + H$^+$ From the reaction of [Re(O)$_2$(ap)]$^-$ and [CPh$_3$]$^+$.	**S10**
**UV-vis Data**		**S10**
**Figure S9**	UV-vis spectra (CH$_2$Cl$_2$) of the reaction products of [Re(O)$_2$(ap)(isq•)] and para-tert-butyl trityl radical (green) and [NEt$_4$][Re(O)$_2$(ap)$_2$] and trityl cation (purple).	**S10**
**Figure S10**	UV-vis spectrum (CH$_2$Cl$_2$) of the reaction products of [Re(O)$_2$(ap)(isq•)], trityl radical, and cyclohexadiene.	**S11**
**Figure S11**	UV-vis spectrum (benzene) of the reaction products of [Re(O)$_2$(ap)(isq•)] and triphenylphosphine after 16h.	**S12**
**Figure S12**	UV-vis spectrum (CH$_2$Cl$_2$) of the reaction products of [Re(O)$_2$(ap)(isq•)] and triphenylarsine after 16h.	**S13**
**Figure S13**	UV-vis spectrum (CH$_2$Cl$_2$) of the reaction products of [Re(O)$_2$(ap)(isq•)] and dimethylsulfide after 24h.	**S14**
**Electrochemical Data**		**S15**
**Figure S14**	Cyclic voltammogram (acetonitrile) of [Re(O)(ap)(isq•)(OCPh$_3$)]	**S15**
**X-Ray Structure Reports**		**S16**
**[Re(O)$_2$(ap)(isq•)]**		**S16**
**[(ap)(isq•)Re(O)(μ-O)Re(O)(ap)(isq•)]**		**S24**
**DFT Simulated EPR Parameters**		
**Table S2**	Computed EPR Parameters	**S57**
**DFT Energetics**		**S57**
**Table S3**	Computed BDFEs for the Re–ORe bond Re–O(μ-oxo) bond in [Re$_2$(μ-O)(O)$_2$(ap)$_2$(isq•)$_2$] with various functionals.	**S57**
**DFT Coordinates**		**S57**
**List S1**	[Re(O)$_2$(ap)(isq•)], M06-L	**S57**
**List S2**	[Re(O)$_2$(ap)(isq•)], CPCM (toluene), M06-L	**S59**
**List S3**	[Re(O)$_2$(ap)(isq•)], CPCM (water), M06-L	**S61**
**List S4**	[Re(O)(ap)(isq•)], M06-L	**S63**
**List S5**	[Re(O)(DMSO)(ap)(isq•)], M06-L	**S65**
**List S6**	[(ap)(isq•)Re(O)(μ-O)Re(O)(ap)(isq•)], M06-L	**S67**
**List S7**	[Re(O)$_2$(ap)$_2$]$^-$, M06-L	**S71**
**List S8**	[Re(O)(ap)$_2$]$^-$, M06-L	**S73**

**List S9**	O_2, M06-L	**S75**
**List S10**	$[Re(O)_2(ap)(isq^{\bullet})]$, CPCM (benzene), M06-L	**S75**
**List S11**	$[Re(O)_2(ap)_2]^{-}$, CPCM (benzene), M06-L	**S77**
**List S12**	$^{\bullet}CPh_3$, CPCM (benzene), M06-L	**S79**
**List S13**	$[CPh3]^{+}$, CPCM (benzene), M06-L	**S80**
**List S14**	$[Re(O)_2(ap)(isq^{\bullet})]$, CPCM (acetonitrile), M06-L	**S81**
**List S15**	$[Re(O)_2(ap)_2]^{-}$, CPCM (acetonitrile), M06-L	**S83**
**List S16**	$^{\bullet}CPh_3$, CPCM (acetonitrile), M06-L	**S85**
**List S17**	$[CPh3]^{+}$, CPCM (acetonitrile), M06-L	**S85**
**List S18**	O_2, BP86	**S86**
**List S19**	$[Re(O)_2(ap)(isq^{\bullet})]$, BP86	**S86**
**List S20**	$[Re(O)_2(ap)_2]^{-}$, BP86	**S88**
**List S21**	$[Re(O)(ap)(isq^{\bullet})]$, BP86	**S91**
**List S22**	$[Re(O)(ap)_2]^{-}$, BP86	**S93**
**List S23**	$[(ap)(isq^{\bullet})Re(O)(\mu\text{-}O)Re(O)(ap)(isq^{\bullet})]$, BP86	**S95**
**List S24**	O_2, BLYP	**S99**
**List S25**	$[Re(O)_2(ap)(isq^{\bullet})]$, BLYP	**S99**
**List S26**	$[Re(O)_2(ap)_2]^{-}$, BLYP	**S101**
**List S27**	$[Re(O)(ap)(isq^{\bullet})]$, BLYP	**S103**
**List S28**	$[Re(O)(ap)_2]^{-}$, BLYP	**S106**
**List S29**	$[(ap)(isq^{\bullet})Re(O)(\mu\text{-}O)Re(O)(ap)(isq^{\bullet})]$, BLYP	**S108**
**List S30**	O_2, PBE	**S112**
**List S31**	$[Re(O)_2(ap)(isq^{\bullet})]$, PBE	**S112**
**List S32**	$[Re(O)_2(ap)_2]^{-}$, PBE	**S114**
**List S33**	$[Re(O)(ap)(isq^{\bullet})]$, PBE	**S116**
**List S34**	$[Re(O)(ap)_2]^{-}$, PBE	**S119**
**List S35**	$[(ap)(isq^{\bullet})Re(O)(\mu\text{-}O)Re(O)(ap)(isq^{\bullet})]$, PBE	**S121**
**List S36**	O_2, TPSS	**S125**
**List S37**	$[Re(O)_2(ap)(isq^{\bullet})]$, TPSS	**S125**
**List S38**	$[Re(O)_2(ap)_2]^{-}$, TPSS	**S127**

**List S39**	[Re(O)(ap)(isq$^{\bullet}$)], TPSS	**S129**
**List S40**	[Re(O)(ap)$_2$]$^{-}$, TPSS	**S131**
**List S41**	[(ap)(isq$^{\bullet}$)Re(O)(μ-O)Re(O)(ap)(isq$^{\bullet}$)], TPSS	**S134**
**List S42**	O_2, B3LYP	**S138**
**List S43**	[Re(O)$_2$(ap)(isq$^{\bullet}$)], B3LYP	**S138**
**List S44**	[Re(O)$_2$(ap)$_2$]$^{-}$, B3LYP	**S140**
**List S45**	[Re(O)(ap)(isq$^{\bullet}$)], B3LYP	**S142**
**List S46**	[Re(O)(ap)$_2$]$^{-}$, B3LYP	**S144**
**List S47**	[(ap)(isq$^{\bullet}$)Re(O)(μ-O)Re(O)(ap)(isq$^{\bullet}$)], B3LYP	**S146**
**List S48**	O_2, PBE0	**S151**
**List S49**	[Re(O)$_2$(ap)(isq$^{\bullet}$)], PBE0	**S151**
**List S50**	[Re(O)$_2$(ap)$_2$]$^{-}$, PBE0	**S153**
**List S51**	[Re(O)(ap)(isq$^{\bullet}$)], PBE0	**S155**
**List S52**	[Re(O)(ap)$_2$]$^{-}$, PBE0	**S157**
**List S53**	[(ap)(isq$^{\bullet}$)Re(O)(μ-O)Re(O)(ap)(isq$^{\bullet}$)], PBE0	**S159**
**References**		**S164**

Table S1. Order determination for degradation of [Re(O)$_2$(ap)(isq$^•$)] to [Re$_2$(μ-O)(O)$_2$(ap)$_2$(isq$^•$)$_2$]. Concentrations from UV-vis measurements of room temperature CH_2Cl_2 solutions at 3h, 19h, and 26h.

	Initial [Re] (M)	Rate (M/h)$_{3h}$	Rate (M/h)$_{19h}$	Rate (M/h)$_{26h}$
1	2.5 x 10^{-3}	1.1 x 10^{-3}	1.1 x 10^{-4}	7.4 x 10^{-5}
2	1.2 x 10^{-3}	5.6 x 10^{-4}	6.6 x 10^{-5}	3.9 x 10^{-5}
3	8.2 x 10^{-4}	3.6 x 10^{-4}	4.0 x 10^{-5}	3.0 x 10^{-5}
4	6.2 x 10^{-4}	3.3 x 10^{-4}	3.9 x 10^{-5}	2.9 x 10^{-5}

DFT Density Plots

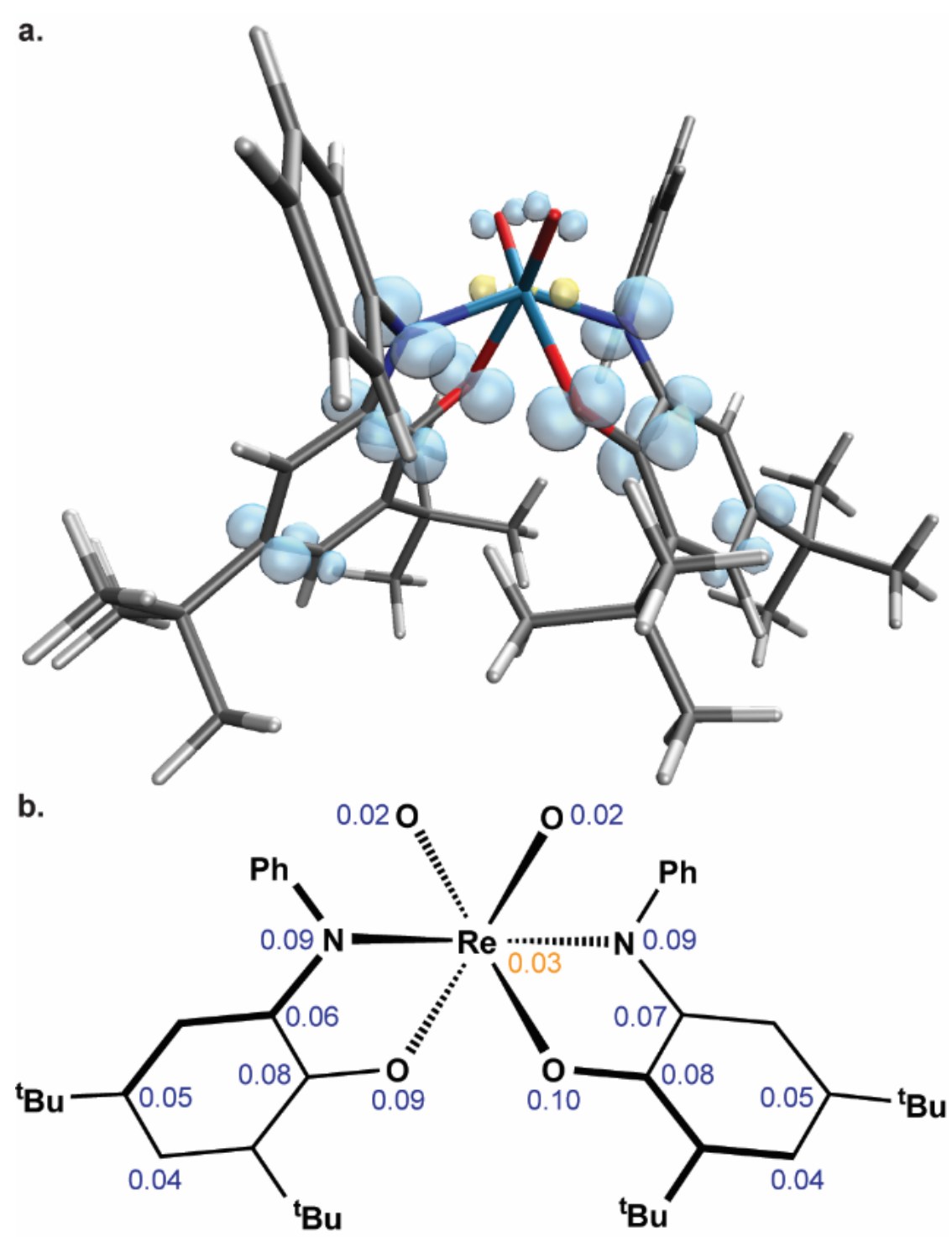

Figure S1. a. Spin-density plot of [Re(O)$_2$(ap)(isq$^•$)] generated in IQmol, isovalue 0.005. **b.** Lowdin spin population per atom (n > 0.02). Spin down density is shown in yellow.

NMR Data

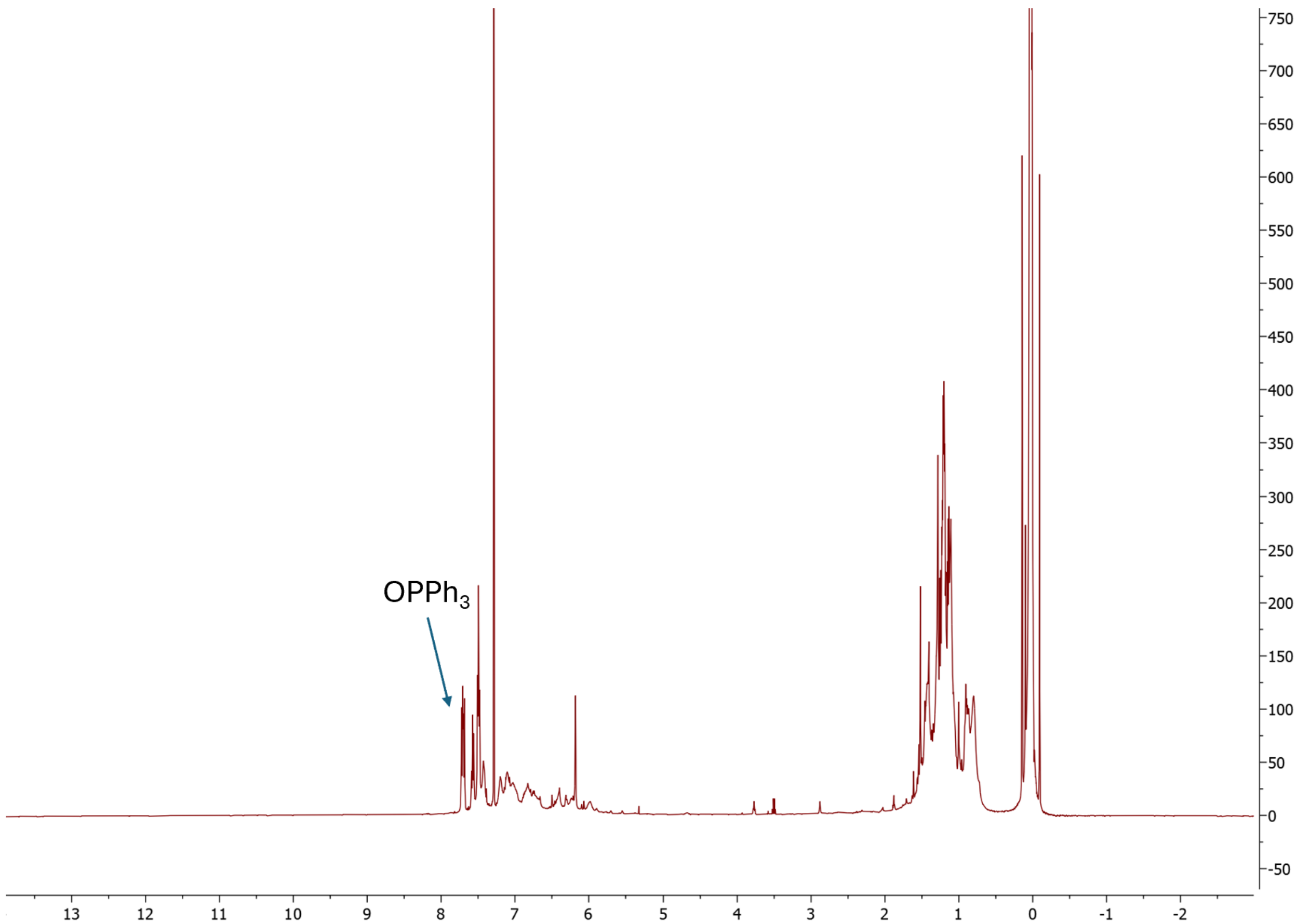

Figure S2. ^{1}H NMR spectrum (500 MHz, $CDCl_3$) of [Re_2(μ-O)$(O)_2$$(ap)_2$(isq•$)_2$] generated by reaction of [Re$(O)_2$(ap)(isq•)] with 0.5 eq PPh_3.

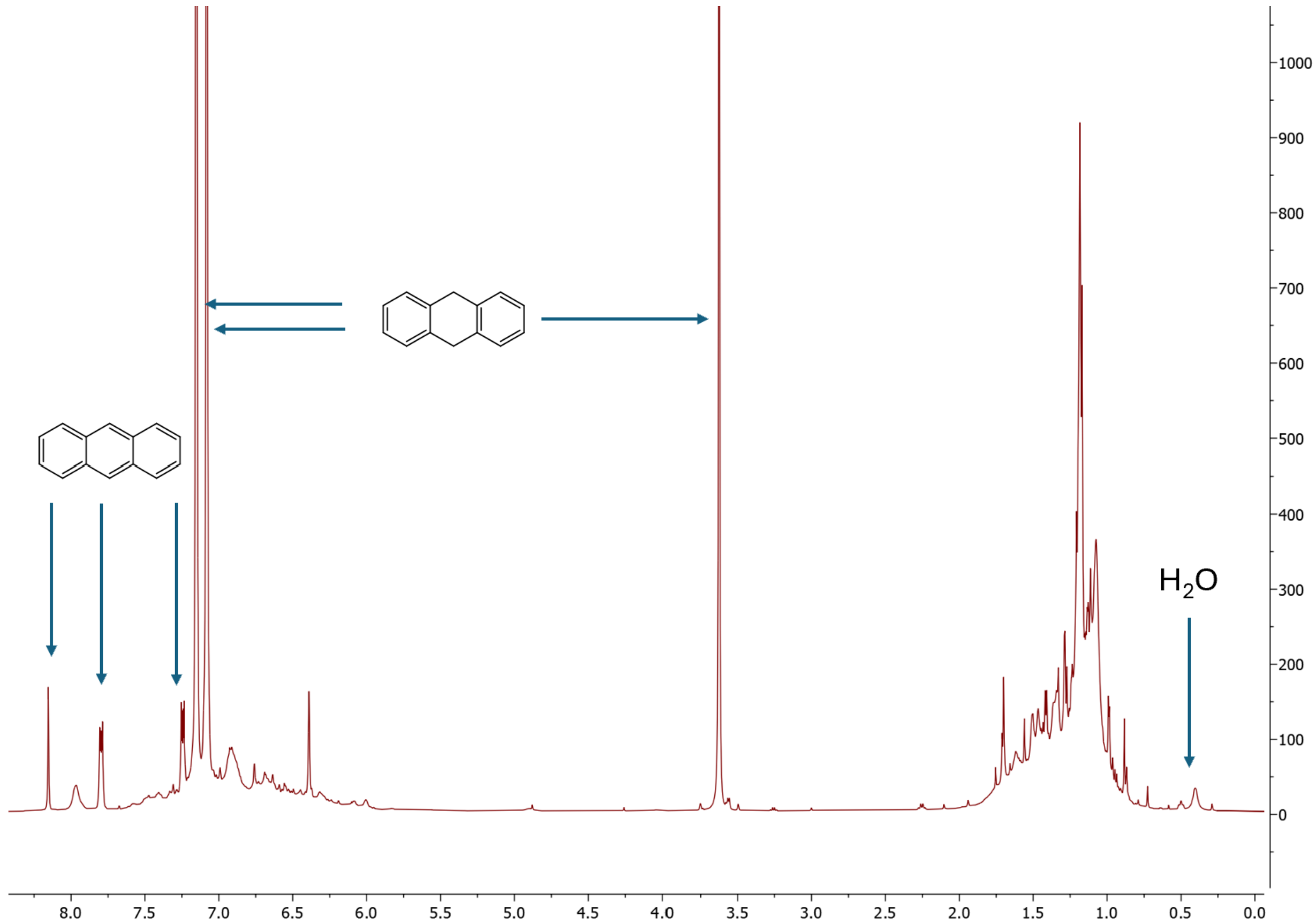

Figure S3. ^{1}H NMR spectrum (500 MHz, C_6D_6) of the reaction products of [Re(O)$_2$(ap)(isq$^•$)] and 9,10-dihydroanthracene after 24h.

Mass Spectrometry Data

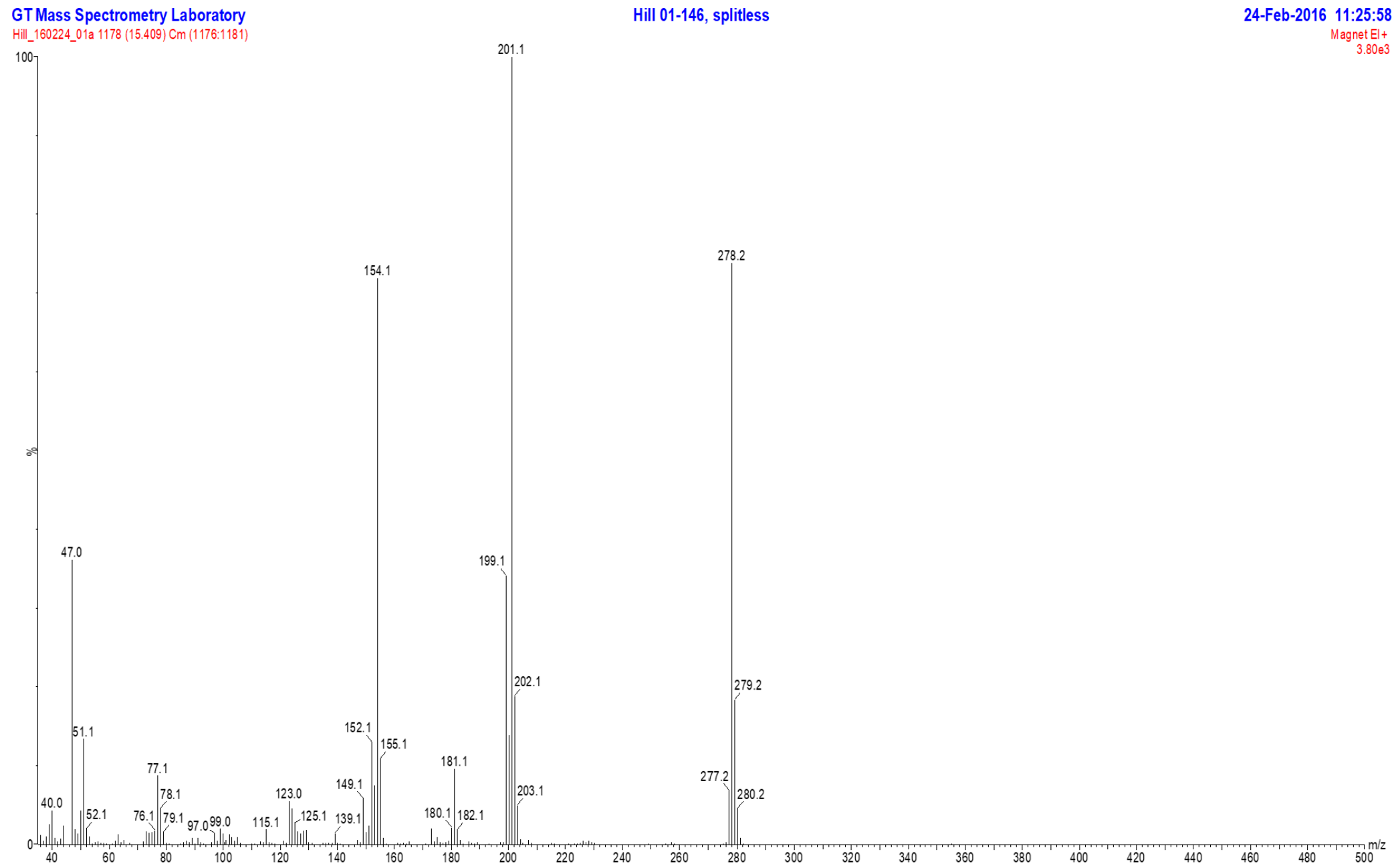

Figure S4. Mass spectrum of $OPPh_3$ from GC-MS of reaction products of [(ap)(isq•)Re(O)$_2$] and PPh_3. Assigned major peaks (m/Z): $OPPh_3^+$ (278.2); $OPPh_2^+$ (201.1); Ph_2^+ (154.1); OP^+ (47.0)

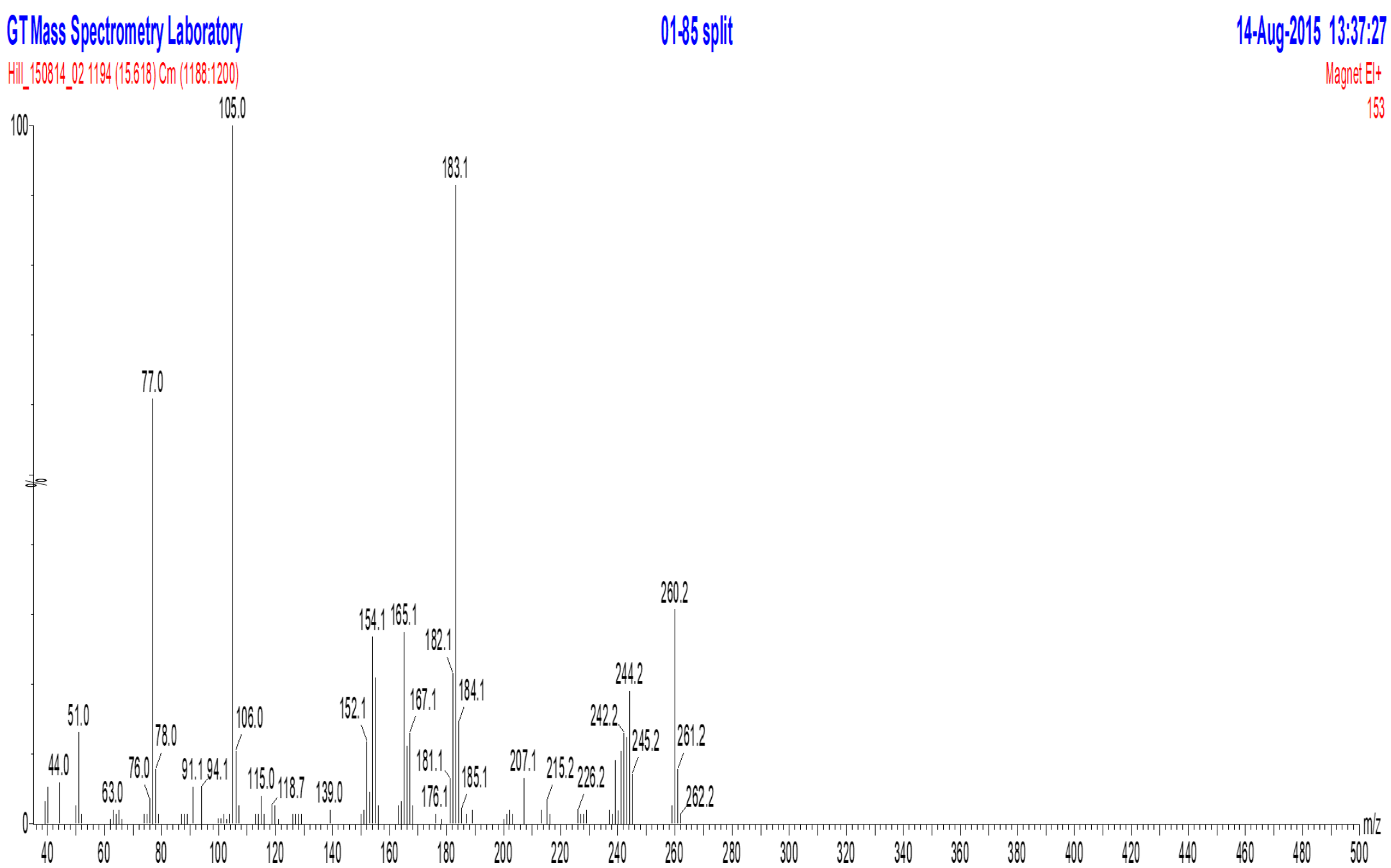

Figure S5. Mass spectrum of $HOCPh_3$ from GC-MS of the reaction products of [Re(O)$_2$(ap)(isq•)] and trityl radical. Assigned major peaks (m/Z): $HOCPh_3^+$ (260.2); $HOCPh_2^+$ (183.1); $OCPh^+$ (105.0); Ph^+ (77.0).

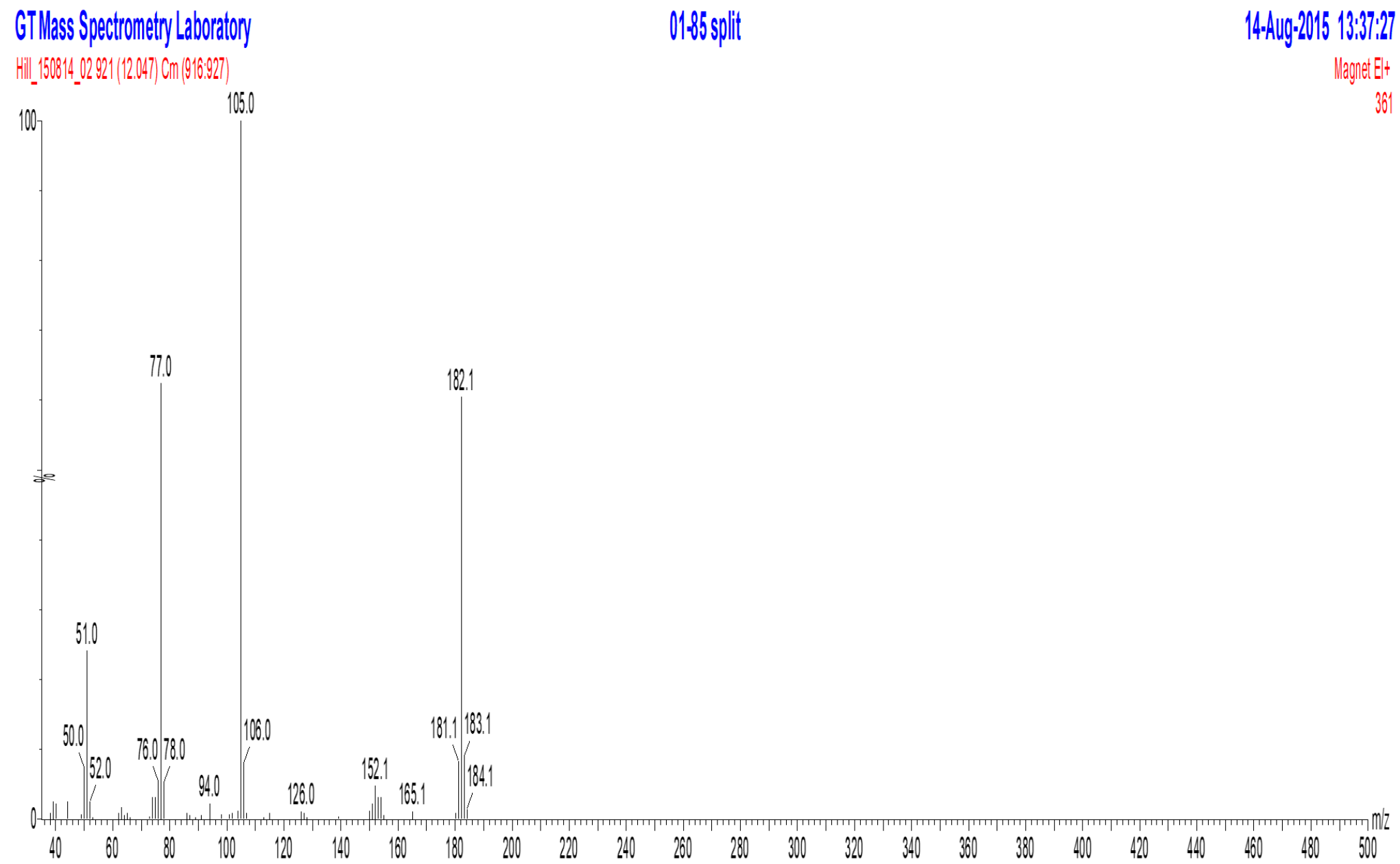

Figure S6. Benzophenone from GC-MS of the reaction products of [Re(O)$_2$(ap)(isq$^\bullet$)] and trityl radical. Assigned major peaks (m/Z): $OCPh_2^+$ (182.1); $OCPh^+$ (105.0); Ph^+ (77.0).

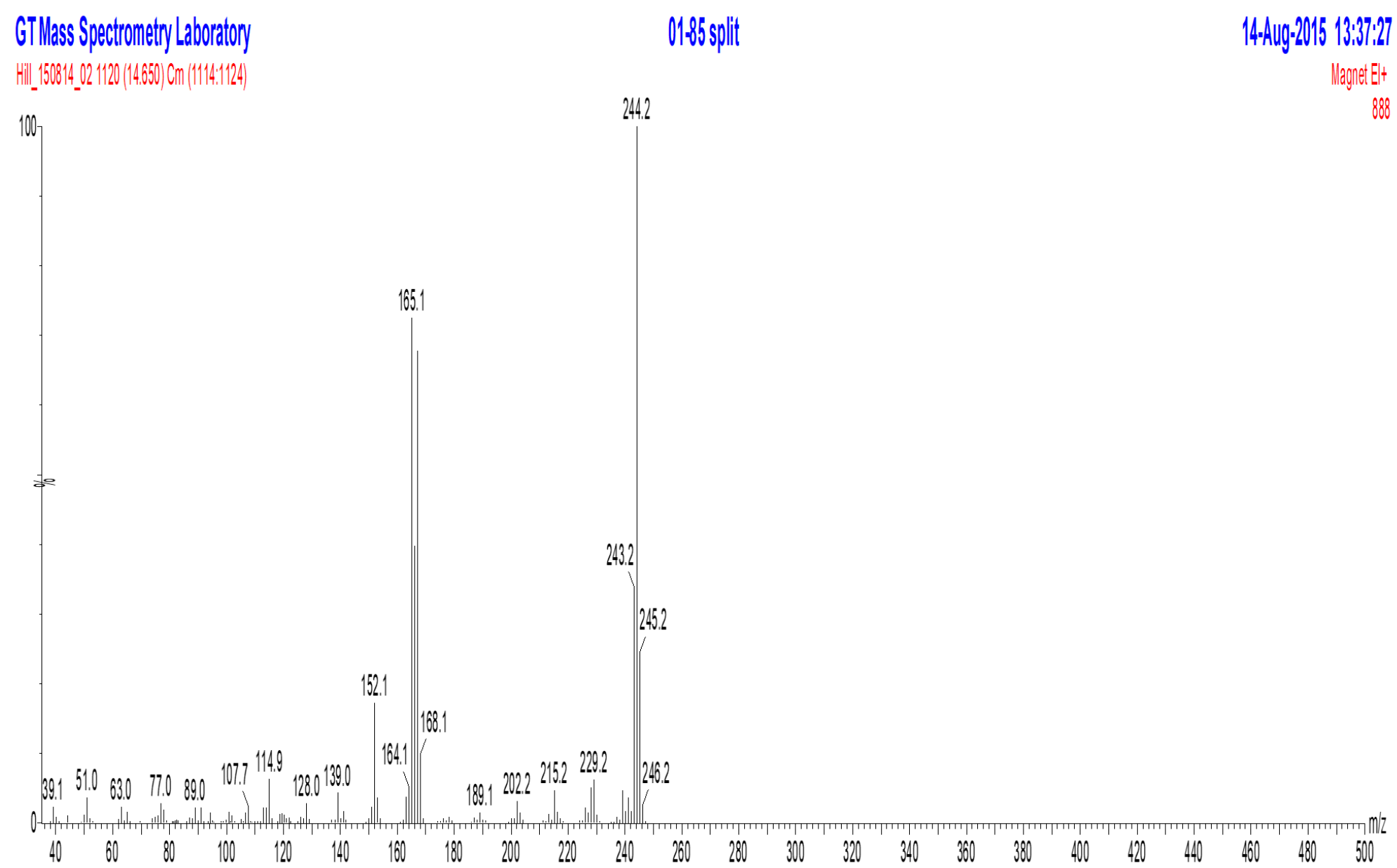

Figure S7. $HCPh_3$ from GC-MS of the reaction products of [Re(O)$_2$(ap)(isq$^\bullet$)] and trityl radical. Assigned major peaks (m/Z): $HCPh_3^+$ (244.2); $HCPh_2^+$ (167.1).

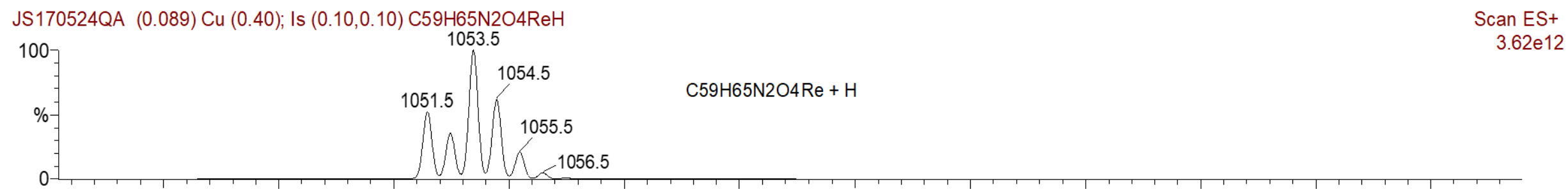

Figure S8. MALDI-MS of [(ap)(isq•)Re(O)(OCPh$_3$)] + H$^+$ From the reaction of [Re(O)$_2$(ap)]$^-$ and [CPh$_3$]$^+$.

UV-vis Data

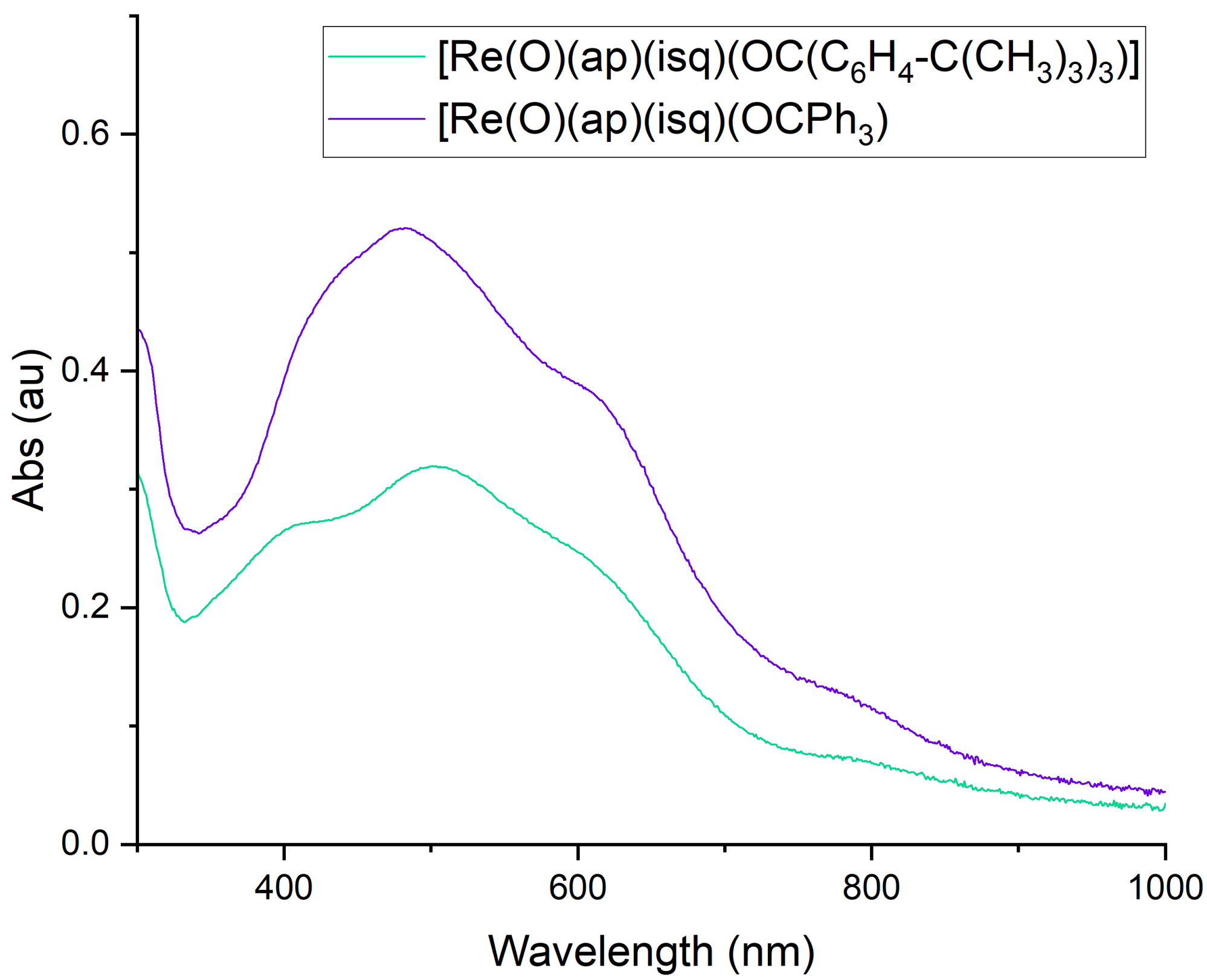

Figure S9. UV-vis spectra (CH_2Cl_2) of the reaction products of [Re(O)$_2$(ap)(isq•)] and *para-tert-*butyl trityl radical (green) and [NEt$_4$][Re(O)$_2$(ap)$_2$] and trityl cation (purple).

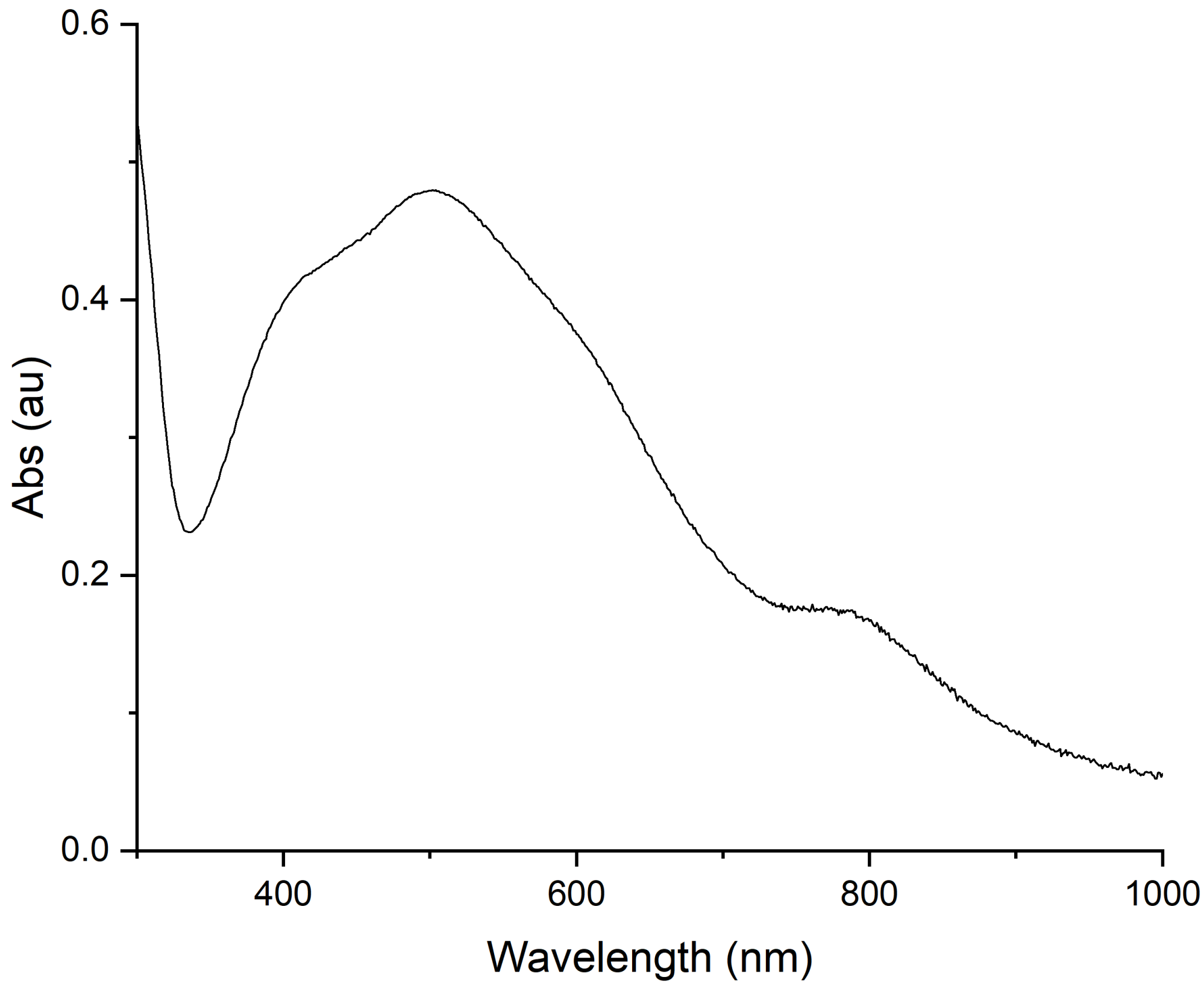

Figure S10. UV-vis spectrum (CH_2Cl_2) of the reaction products of [Re(O)$_2$(ap)(isq$^\bullet$)], trityl radical, and cyclohexadiene.

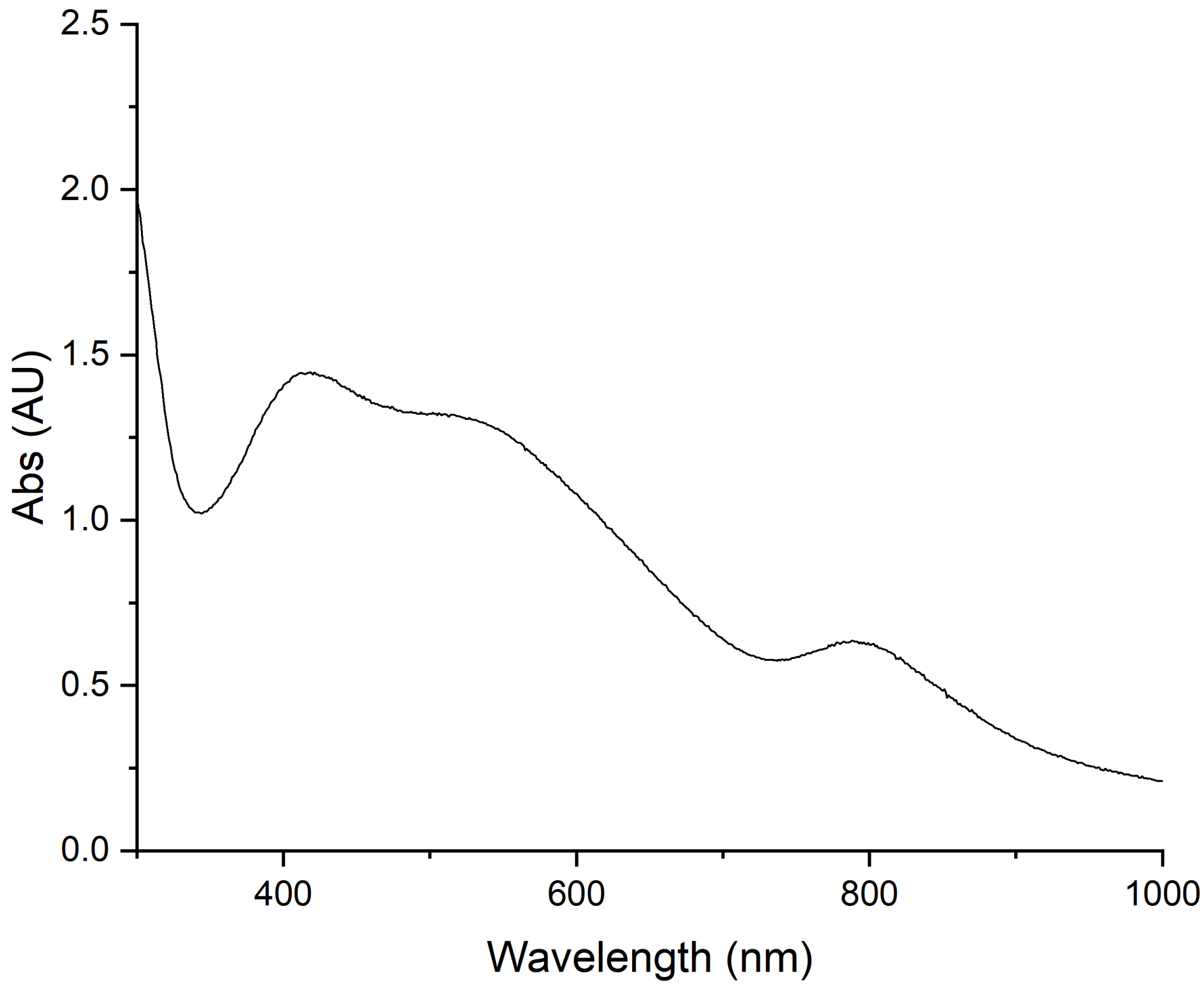

Figure S11. UV-vis spectrum (benzene) of the reaction products of [Re(O)$_2$(ap)(isq$^\bullet$)] and triphenylphosphine after 16h.

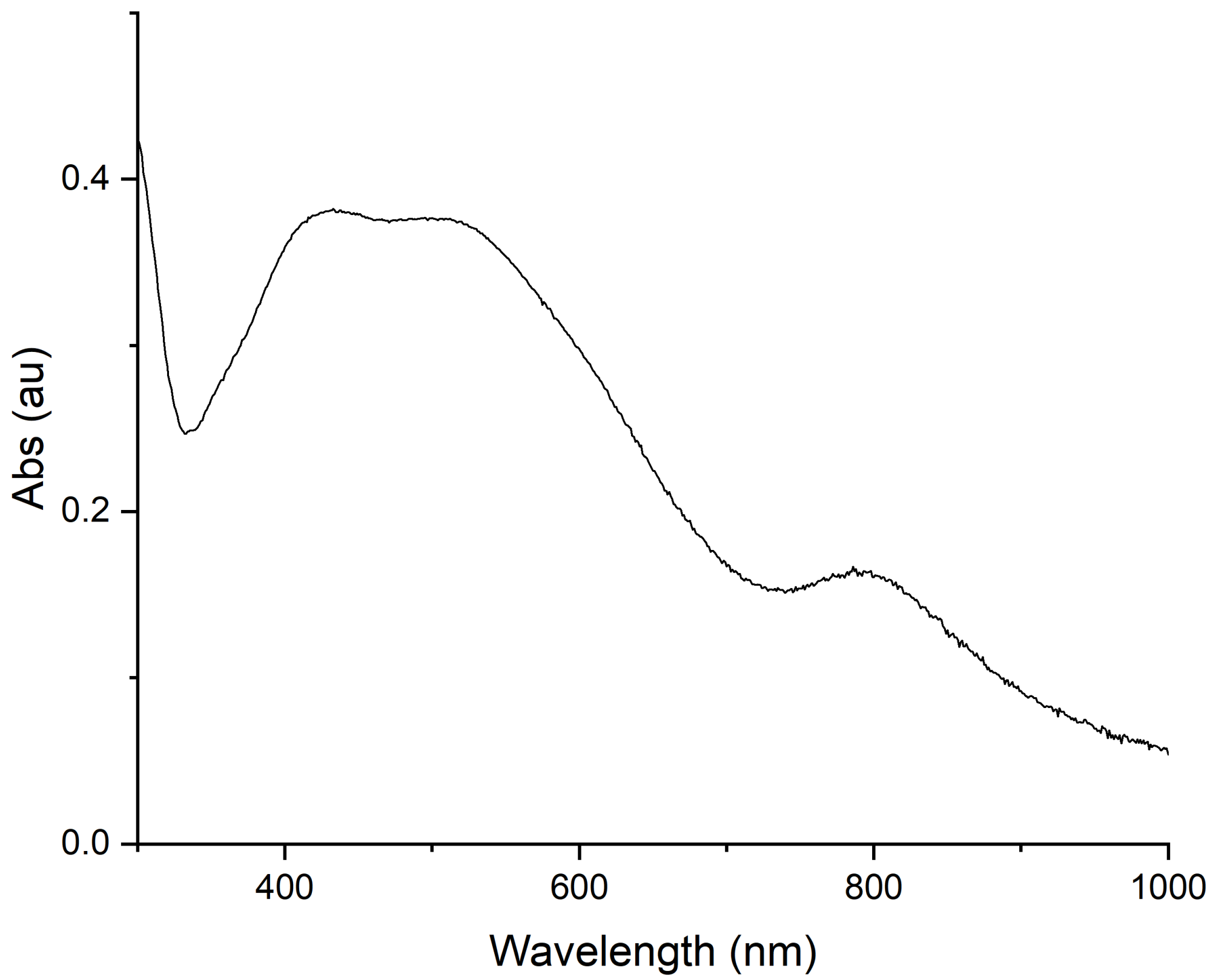

Figure S12. UV-vis spectrum (CH_2Cl_2) of the reaction products of [Re(O)$_2$(ap)(isq$^{\bullet}$)] and triphenylarsine after 16h.

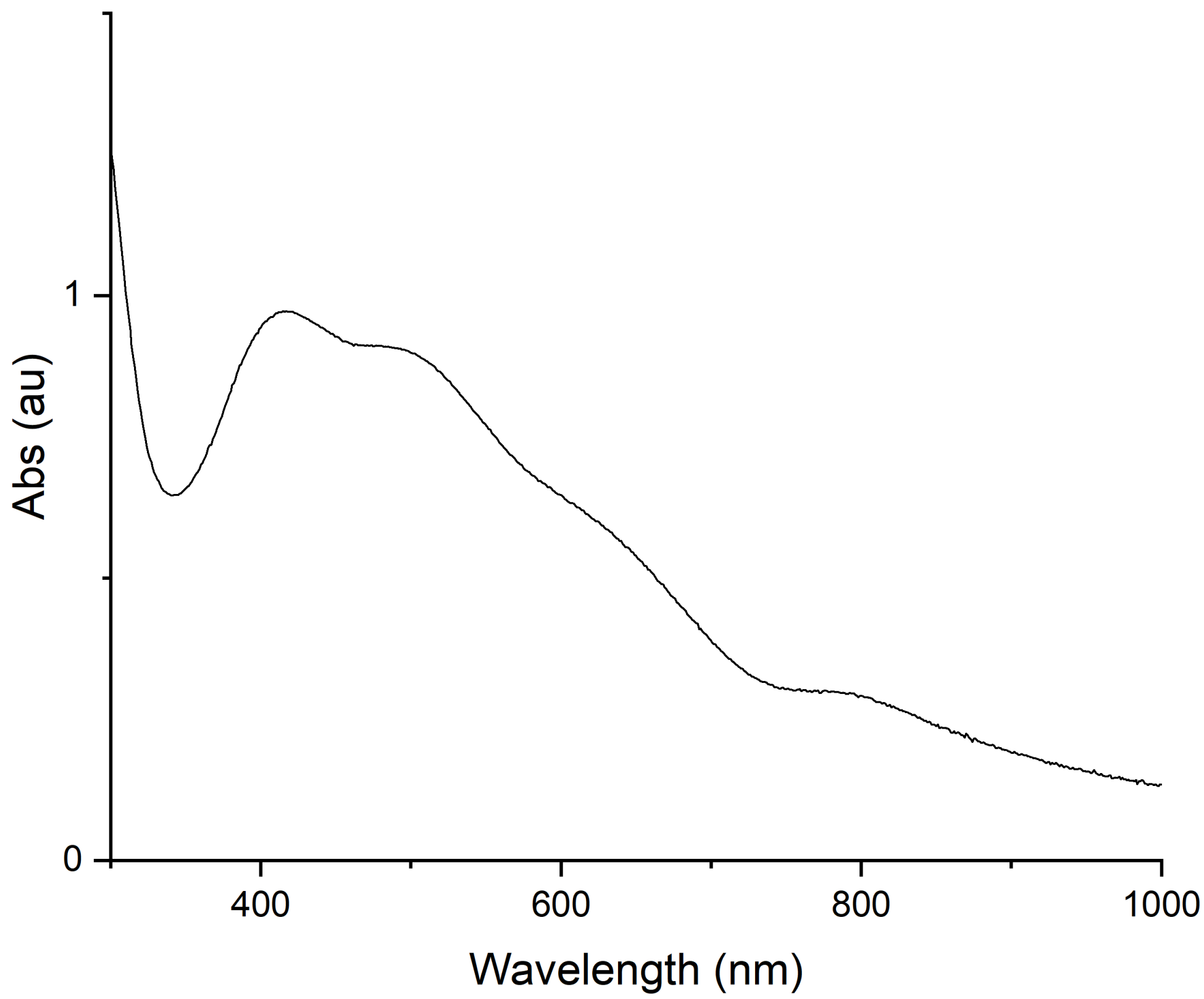

Figure S13. UV-vis spectrum (CH_2Cl_2) of the reaction products of [Re(O)$_2$(ap)(isq$^\bullet$)] and dimethylsulfide after 24h.

Electrochemical Data

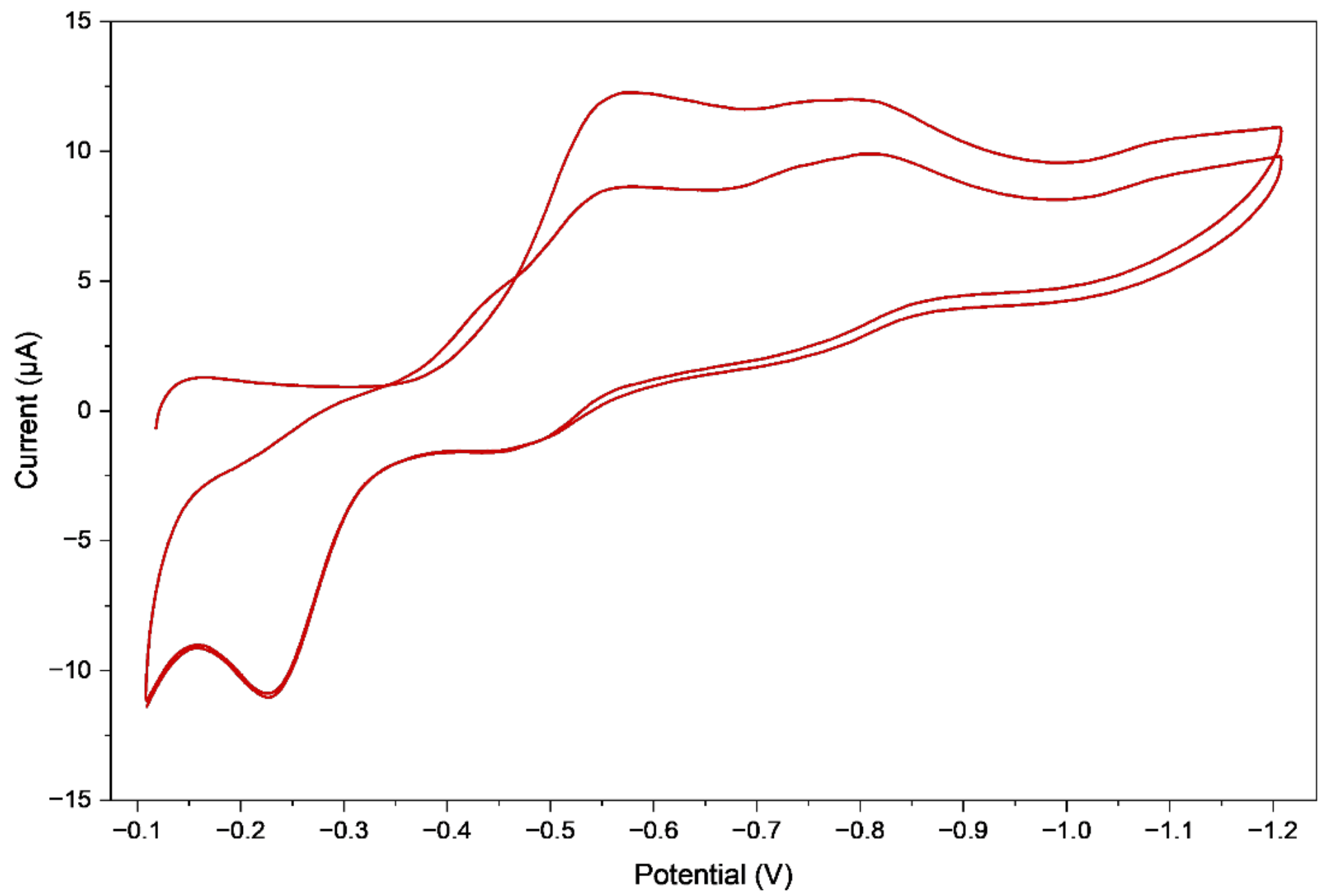

Figure S14. Cyclic voltammogram (CH_3CN, 0.1 M $[Bu_4N][PF_6]$) of $[Re(O)(ap)(isq^{\bullet})(OCPh_3)]$.

X-Ray Structure Reports

$C_{40}H_{50}N_2O_4Re$

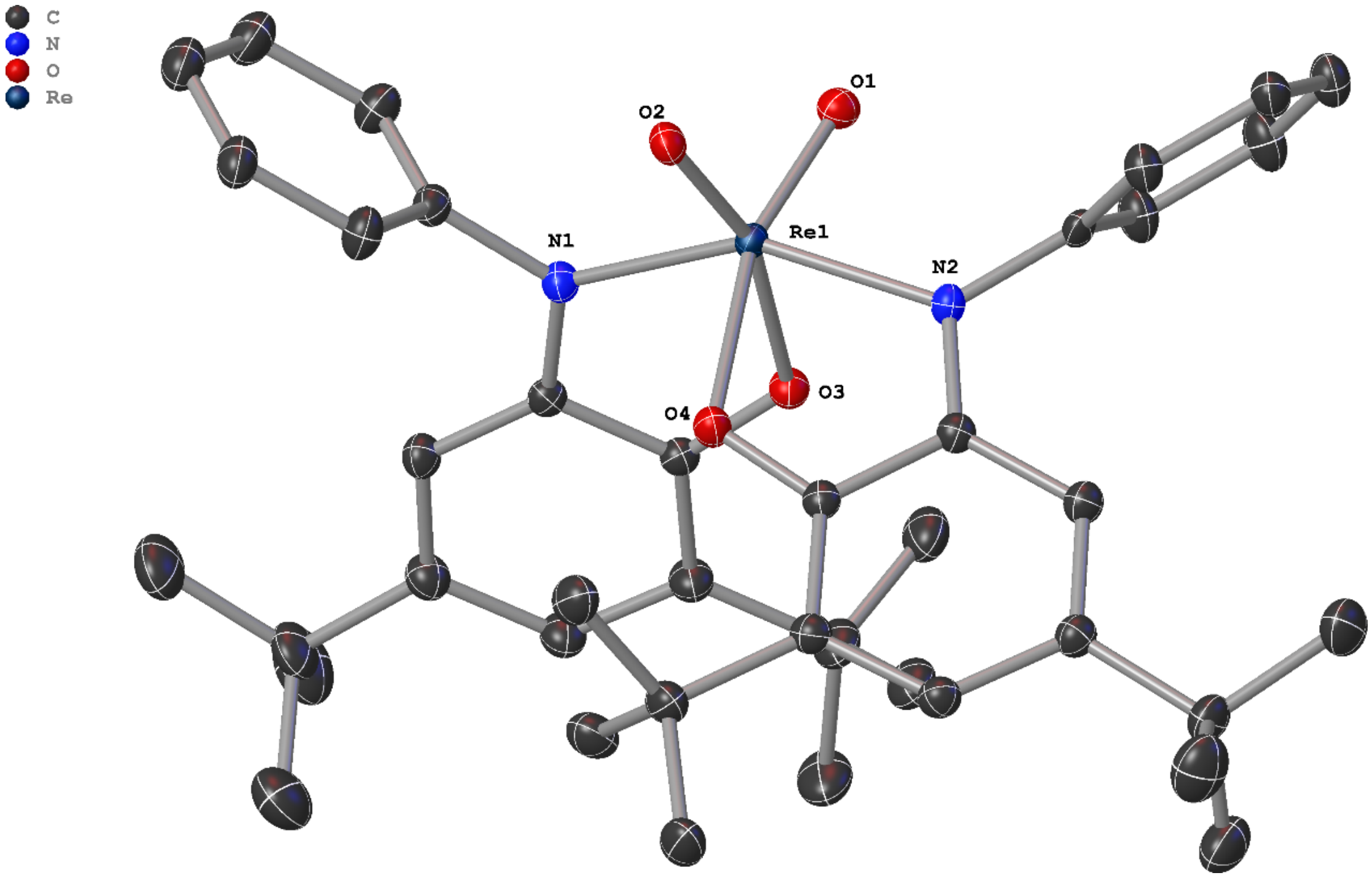

Experimental

A green, plate-shaped crystal of $C_{40}H_{50}N_2O_4Re$ was selected and mounted on a loop with paratone oil on a Bruker APEX-II CCD diffractometer equipped with an Oxford Cryostream 800 low-temperature apparatus operating at 100K. Data were collected using MoKα radiation (microfocus sealed tube, 50 kV). Using Olex2[1], the structure was solved with the XT[2] structure solution program using Intrinsic Phasing and refined with the XL[3] refinement package using Least Squares minimisation.

Crystal data and structure refinement for JAH_2_6.

Identification code	JAH_2_6
Empirical formula	$C_{40}H_{50}N_2O_4Re$
Formula weight	759.12
Temperature/K	99.98
Crystal system	triclinic
Space group	P-1
a/Å	8.7972(11)
b/Å	11.2623(15)
c/Å	20.013(3)
α/°	87.692(5)
β/°	82.308(4)
γ/°	71.598(5)

Volume/Å^3	1860.8(3)
Z	2
ρ_{calc}g/cm^3	1.355
μ/mm^{-1}	3.303
F(000)	723.0
Crystal size/mm^3	0.13 × 0.1 × 0.01
Radiation	MoKα (λ = 0.71073)
2Θ range for data collection/°	4.924 to 56.564
Index ranges	-11 ≤ h ≤ 11, -14 ≤ k ≤ 14, -26 ≤ l ≤ 26
Reflections collected	14716
Independent reflections	14716 [R_{int} = ?, R_{sigma} = 0.0748]
Data/restraints/parameters	14716/429/437
Goodness-of-fit on F^2	1.076
Final R indexes [I>=2σ (I)]	R_1 = 0.0510, wR_2 = 0.0954
Final R indexes [all data]	R_1 = 0.0653, wR_2 = 0.1025
Largest diff. peak/hole / e Å^{-3}	1.74/-2.08

Fractional Atomic Coordinates (×10^4) and Equivalent Isotropic Displacement Parameters (Å^2×10^3) for JAH_2_6. U_{eq} is defined as 1/3 of of the trace of the orthogonalised U_{IJ} tensor.

Atom	*x*	*y*	*z*	U(eq)
Re1	4026.4(4)	4335.5(3)	8033.5(2)	12.07(7)
O1	5816(6)	3481(5)	7300(2)	15.7(9)
O2	3575(6)	2602(5)	8002(3)	15.9(9)
O3	4915(6)	5455(5)	8177(2)	17.1(9)
O4	2131(6)	4955(5)	8481(2)	16.4(9)
N1	2989(7)	4679(6)	7184(3)	15.1(9)
N2	5514(7)	3156(6)	8670(3)	13.7(9)
C3	1363(8)	5499(7)	7140(4)	16.7(11)
C4	58(8)	5090(8)	7363(4)	21.5(14)
C1	5429(8)	3340(7)	6698(3)	13.9(12)
C5	-1498(9)	5865(8)	7306(4)	23.1(14)
C21	4527(8)	1641(7)	8285(3)	13.7(11)
C22	5629(8)	1926(7)	8670(3)	14.1(11)
C9	3245(9)	3993(7)	5997(4)	17.1(13)
C30	6756(8)	-268(7)	8937(4)	16.8(12)
C31	5613(8)	-509(7)	8563(4)	17.5(13)
C2	3837(8)	4031(7)	6605(4)	13.9(12)
C33	3238(9)	100(7)	7862(4)	20.2(11)
C6	-1726(9)	7041(8)	7024(4)	25.3(15)
C32	4469(8)	397(7)	8245(4)	16.5(11)

C24	7834(9)	3846(7)	8775(4)	21.2(15)
C23	6524(8)	3515(7)	9074(4)	14.4(11)
C29	6749(8)	957(7)	8993(4)	16.7(13)
C26	8436(9)	4206(8)	9876(4)	22.5(15)
C34	3353(9)	-1290(7)	7924(4)	25.8(15)
C37	7962(9)	-1357(7)	9255(4)	22.1(12)
C28	6156(9)	3525(7)	9774(4)	18.5(14)
C7	-418(9)	7435(8)	6794(4)	26.7(15)
C17	3669(10)	3112(9)	4803(4)	29.3(13)
C35	1523(9)	890(8)	8149(4)	24.3(15)
C10	4240(9)	3238(7)	5478(4)	20.1(13)
C27	7131(8)	3864(7)	10173(4)	19.7(14)
C12	6487(9)	2554(7)	6183(4)	20.0(13)
C8	1139(9)	6668(7)	6854(4)	22.4(14)
C25	8803(9)	4193(8)	9174(4)	25.4(16)
C11	5850(9)	2549(7)	5581(4)	21.1(15)
C36	3573(10)	395(8)	7111(4)	25.2(15)
C38	9173(10)	-2127(8)	8688(4)	31.6(17)
C14	9135(9)	2694(8)	6439(4)	28.2(16)
C13	8216(9)	1798(8)	6295(4)	26.5(12)
C39	8939(10)	-895(8)	9710(4)	31.4(16)
C15	8156(9)	922(8)	6896(4)	27.7(15)
C18	1962(11)	4003(9)	4754(4)	38.1(18)
C40	7111(11)	-2152(9)	9675(5)	36.2(17)
C19	4789(11)	3405(10)	4227(4)	43.0(19)
C16	9144(10)	999(9)	5679(4)	34.5(17)
C20	3637(12)	1760(9)	4730(5)	41.2(19)

Anisotropic Displacement Parameters (Å^2×10^3) for JAH_2_6. The Anisotropic displacement factor exponent takes the form: $-2\pi^2[h^2a^{*2}U_{11}+2hka^*b^*U_{12}+...]$.

Atom	U_{11}	U_{22}	U_{33}	U_{23}	U_{13}	U_{12}
Re1	11.57(12)	11.84(14)	11.84(13)	0.40(9)	-2.47(9)	-1.96(10)
O1	14.5(16)	18(2)	13.4(14)	-1.3(12)	-0.5(11)	-3.8(14)
O2	15.6(17)	15.8(15)	17.3(19)	0.5(12)	-4.3(15)	-5.2(12)
O3	18(2)	17.0(18)	16(2)	-1.1(15)	-1.7(16)	-4.7(15)
O4	15.3(15)	20(2)	14.2(19)	-1.4(15)	-3.7(13)	-5.4(14)
N1	13.5(15)	17.7(19)	13.5(14)	-0.4(12)	-1.9(11)	-4.1(13)
N2	11.4(18)	15.6(16)	14.2(17)	1.7(12)	-2.7(14)	-4.2(13)
C3	15.8(16)	19(2)	14(3)	-1.0(17)	-3.1(14)	-2.6(14)

C4	16.3(18)	24(3)	24(3)	5(2)	-4.8(17)	-5.0(17)
C1	14.3(17)	14(3)	13.0(16)	0.4(15)	-0.6(12)	-4.4(16)
C5	18(2)	29(3)	20(3)	-1(2)	-4.8(19)	-1.8(18)
C21	12(2)	16.0(16)	12(2)	-0.7(13)	0(2)	-4.0(13)
C22	14(2)	15.6(16)	12(2)	0.9(13)	-1(2)	-4.7(13)
C9	16(2)	20(3)	14.6(17)	1.2(17)	-3.0(15)	-5(2)
C30	17(2)	16.9(18)	16(2)	1.2(15)	-2.0(19)	-4.4(15)
C31	18(2)	16(2)	18(3)	0.2(18)	-2(2)	-5.4(16)
C2	15.0(17)	13(3)	13.6(15)	1.8(15)	-1.6(12)	-5.0(16)
C33	19(2)	19(2)	24(2)	-2.5(18)	-5.3(18)	-6.2(17)
C6	21(2)	28(3)	22(4)	0(2)	-5(2)	-0.8(19)
C32	16(2)	16.5(17)	17(2)	-0.9(15)	-1.3(19)	-5.5(14)
C24	19(2)	28(4)	20(2)	3(2)	-5.3(18)	-11(3)
C23	14(2)	11(3)	17.7(19)	2.2(16)	-5.8(15)	-2.2(19)
C29	17(2)	17.1(18)	16(3)	1.5(15)	-3(2)	-4.6(15)
C26	21(3)	27(4)	24(2)	3(2)	-8.6(19)	-11(3)
C34	25(3)	21(2)	35(4)	-1(2)	-11(3)	-9(2)
C37	23(2)	19(2)	23(2)	3.6(17)	-7.9(19)	-2.0(17)
C28	17(2)	23(4)	17.0(19)	1.6(17)	-4.5(16)	-7(2)
C7	22(2)	25(3)	28(4)	2(2)	-5(2)	-0.2(18)
C17	31(3)	39(3)	18(2)	-5.2(19)	-7.6(18)	-9(2)
C35	19(2)	24(3)	31(3)	-1(2)	-5(2)	-7.5(19)
C10	20(2)	24(3)	16.1(18)	-1.5(17)	-1.7(15)	-6.7(18)
C27	20(2)	19(4)	21(2)	1(2)	-7.2(18)	-6(2)
C12	16.5(17)	23(3)	17.6(18)	-4.9(17)	0.0(13)	-2.9(16)
C8	20(2)	20(2)	25(3)	3(2)	-4(2)	-3.6(16)
C25	24(3)	35(4)	24(2)	3(2)	-7.9(19)	-17(3)
C11	20(2)	25(3)	17(2)	-4.5(19)	-0.6(16)	-5(2)
C36	30(3)	22(3)	26(2)	-3(2)	-6(2)	-11(3)
C38	30(3)	29(3)	29(3)	1(2)	-8(2)	2(2)
C14	19(3)	33(3)	28(4)	-4(3)	-4(3)	-1(2)
C13	16.6(19)	31(3)	25(2)	-8.1(19)	-1.1(16)	1.8(17)
C39	35(3)	27(4)	32(3)	5(3)	-16(3)	-6(3)
C15	18(3)	30(3)	29(3)	-7(2)	-2(2)	2(2)
C18	37(3)	50(4)	26(4)	-6(3)	-12(2)	-7(2)
C40	35(3)	33(3)	40(3)	13(3)	-10(3)	-10(3)
C19	44(3)	65(5)	23(3)	-1(3)	-7(2)	-21(3)
C16	23(3)	41(4)	30(3)	-13(3)	0(2)	3(3)
C20	49(4)	45(3)	33(4)	-6(2)	-13(3)	-16(3)

Bond Lengths for JAH_2_6.

Atom	Atom	Length/Å	Atom	Atom	Length/Å
Re1	O1	2.023(5)	C31	C32	1.388(10)
Re1	O2	2.114(5)	C33	C32	1.531(10)
Re1	O3	1.728(5)	C33	C34	1.536(10)
Re1	O4	1.726(5)	C33	C35	1.535(10)
Re1	N1	2.002(6)	C33	C36	1.536(10)
Re1	N2	2.075(6)	C6	C7	1.376(11)
O1	C1	1.323(8)	C24	C23	1.375(10)
O2	C21	1.303(8)	C24	C25	1.390(10)
N1	C3	1.448(9)	C23	C28	1.393(10)
N1	C2	1.387(9)	C26	C27	1.377(10)
N2	C22	1.356(9)	C26	C25	1.398(11)
N2	C23	1.433(9)	C37	C38	1.532(11)
C3	C4	1.381(10)	C37	C39	1.539(11)
C3	C8	1.380(10)	C37	C40	1.503(11)
C4	C5	1.389(10)	C28	C27	1.391(9)
C1	C2	1.403(9)	C7	C8	1.387(10)
C1	C12	1.416(9)	C17	C10	1.529(10)
C5	C6	1.382(11)	C17	C18	1.533(12)
C21	C22	1.431(9)	C17	C19	1.508(12)
C21	C32	1.422(10)	C17	C20	1.544(13)
C22	C29	1.419(10)	C10	C11	1.421(10)
C9	C2	1.392(10)	C12	C11	1.395(10)
C9	C10	1.387(10)	C12	C13	1.531(10)
C30	C31	1.425(10)	C14	C13	1.533(11)
C30	C29	1.386(10)	C13	C15	1.530(11)
C30	C37	1.526(10)	C13	C16	1.533(10)

Bond Angles for JAH_2_6.

Atom	Atom	Atom	Angle/˚	Atom	Atom	Atom	Angle/˚
O1	Re1	O2	80.89(16)	C32	C33	C35	109.5(6)
O1	Re1	N2	83.9(2)	C32	C33	C36	109.3(6)
O3	Re1	O1	93.2(2)	C34	C33	C36	108.1(6)
O3	Re1	O2	161.7(2)	C35	C33	C34	108.5(6)
O3	Re1	N1	111.6(2)	C35	C33	C36	110.0(6)
O3	Re1	N2	87.7(2)	C7	C6	C5	120.3(7)

O4	Re1	O1	161.5(2)	C21	C32	C33	121.2(7)
O4	Re1	O2	88.8(2)	C31	C32	C21	115.5(7)
O4	Re1	O3	101.1(2)	C31	C32	C33	123.3(7)
O4	Re1	N1	88.3(2)	C23	C24	C25	119.7(7)
O4	Re1	N2	108.2(2)	C24	C23	N2	120.4(6)
N1	Re1	O1	75.4(2)	C24	C23	C28	120.7(7)
N1	Re1	O2	83.8(2)	C28	C23	N2	118.9(6)
N1	Re1	N2	152.1(2)	C30	C29	C22	119.2(7)
N2	Re1	O2	74.5(2)	C27	C26	C25	120.3(7)
C1	O1	Re1	118.5(4)	C30	C37	C38	108.2(6)
C21	O2	Re1	117.7(4)	C30	C37	C39	111.7(7)
C3	N1	Re1	124.5(4)	C38	C37	C39	106.9(7)
C2	N1	Re1	118.2(5)	C40	C37	C30	110.9(6)
C2	N1	C3	117.2(6)	C40	C37	C38	111.0(7)
C22	N2	Re1	117.6(5)	C40	C37	C39	108.1(7)
C22	N2	C23	116.8(6)	C27	C28	C23	119.6(7)
C23	N2	Re1	125.4(5)	C6	C7	C8	120.5(8)
C4	C3	N1	119.9(7)	C10	C17	C18	111.9(7)
C8	C3	N1	119.4(6)	C10	C17	C20	109.0(7)
C8	C3	C4	120.7(7)	C18	C17	C20	107.9(7)
C3	C4	C5	119.8(8)	C19	C17	C10	110.5(7)
O1	C1	C2	114.2(6)	C19	C17	C18	107.9(8)
O1	C1	C12	124.4(6)	C19	C17	C20	109.7(8)
C2	C1	C12	121.4(7)	C9	C10	C17	122.6(7)
C6	C5	C4	119.5(7)	C9	C10	C11	118.5(7)
O2	C21	C22	115.0(6)	C11	C10	C17	118.9(7)
O2	C21	C32	123.8(6)	C26	C27	C28	119.9(7)
C32	C21	C22	121.1(7)	C1	C12	C13	120.7(7)
N2	C22	C21	114.0(6)	C11	C12	C1	115.4(7)
N2	C22	C29	125.5(6)	C11	C12	C13	123.9(7)
C29	C22	C21	120.5(7)	C3	C8	C7	119.2(7)
C10	C9	C2	119.3(7)	C24	C25	C26	119.7(7)
C31	C30	C37	119.6(7)	C12	C11	C10	124.0(7)
C29	C30	C31	118.6(7)	C12	C13	C14	109.5(7)
C29	C30	C37	121.8(7)	C12	C13	C16	112.1(7)
C32	C31	C30	125.0(7)	C15	C13	C12	109.0(6)
N1	C2	C1	111.7(6)	C15	C13	C14	109.8(7)
N1	C2	C9	126.9(6)	C15	C13	C16	108.2(7)
C9	C2	C1	121.4(7)	C16	C13	C14	108.2(7)
C32	C33	C34	111.4(6)				

Hydrogen Atom Coordinates (Å×10^4) and Isotropic Displacement Parameters (Å^2×10^3) for JAH_2_6.

Atom	*x*	*y*	*z*	U(eq)
H4	226	4280	7555	26
H5	-2401	5590	7459	28
H9	2171	4478	5939	21
H31	5637	-1354	8529	21
H6	-2789	7580	6990	30
H24	8075	3838	8298	25
H29	7486	1146	9246	20
H26	9090	4452	10150	27
H34A	4421	-1806	7717	39
H34B	2515	-1442	7694	39
H34C	3198	-1510	8402	39
H28	5244	3301	9976	22
H7	-584	8237	6592	32
H35A	1354	754	8636	36
H35B	733	644	7932	36
H35C	1389	1779	8061	36
H27	6897	3859	10651	24
H8	2041	6945	6700	27
H25	9712	4420	8970	30
H11	6535	2055	5218	25
H36A	3497	1281	7059	38
H36B	2774	220	6865	38
H36C	4660	-127	6929	38
H38A	8611	-2507	8410	47
H38B	10016	-2787	8883	47
H38C	9667	-1579	8407	47
H14A	9160	3259	6055	42
H14B	10243	2210	6509	42
H14C	8589	3187	6845	42
H39A	9533	-396	9443	47
H39B	9705	-1617	9903	47
H39C	8202	-378	10075	47
H15A	7631	1418	7303	42
H15B	9258	417	6963	42
H15C	7541	368	6807	42

H18A	1194	3783	5099	57
H18B	1665	3929	4306	57
H18C	1935	4866	4826	57
H40A	6270	-1623	10005	54
H40B	7892	-2792	9911	54
H40C	6615	-2559	9381	54
H19A	4843	4251	4285	65
H19B	4381	3357	3800	65
H19C	5871	2797	4222	65
H16A	8555	441	5569	52
H16B	10221	499	5781	52
H16C	9251	1547	5295	52
H20A	4738	1177	4711	62
H20B	3194	1688	4315	62
H20C	2958	1556	5118	62

Crystal Data for $C_{40}H_{50}N_2O_4Re$ (M =759.12 g/mol): triclinic, space group P-1 (no. 2), a = 8.7919(9) Å, b = 11.2509(12) Å, c = 20.006(2) Å, α = 87.708(4)°, β = 82.298(4)°, γ = 71.599(4)°, V = 1860.8(3) Å^3, Z = 2, T = 99.98 K, μ(MoKα) = 3.303 mm^{-1}, $Dcalc$ = 1.355 g/cm^3, 14716 reflections measured (4.924° ≤ 2Θ ≤ 56.564°), 14716 unique (R_{int} = ?, R_{sigma} = 0.0748) which were used in all calculations. The final R_1 was 0.0510 (I > 2σ(I)) and wR_2 was 0.1025 (all data).

$C_{80}H_{100}N_4O_7Re_2$

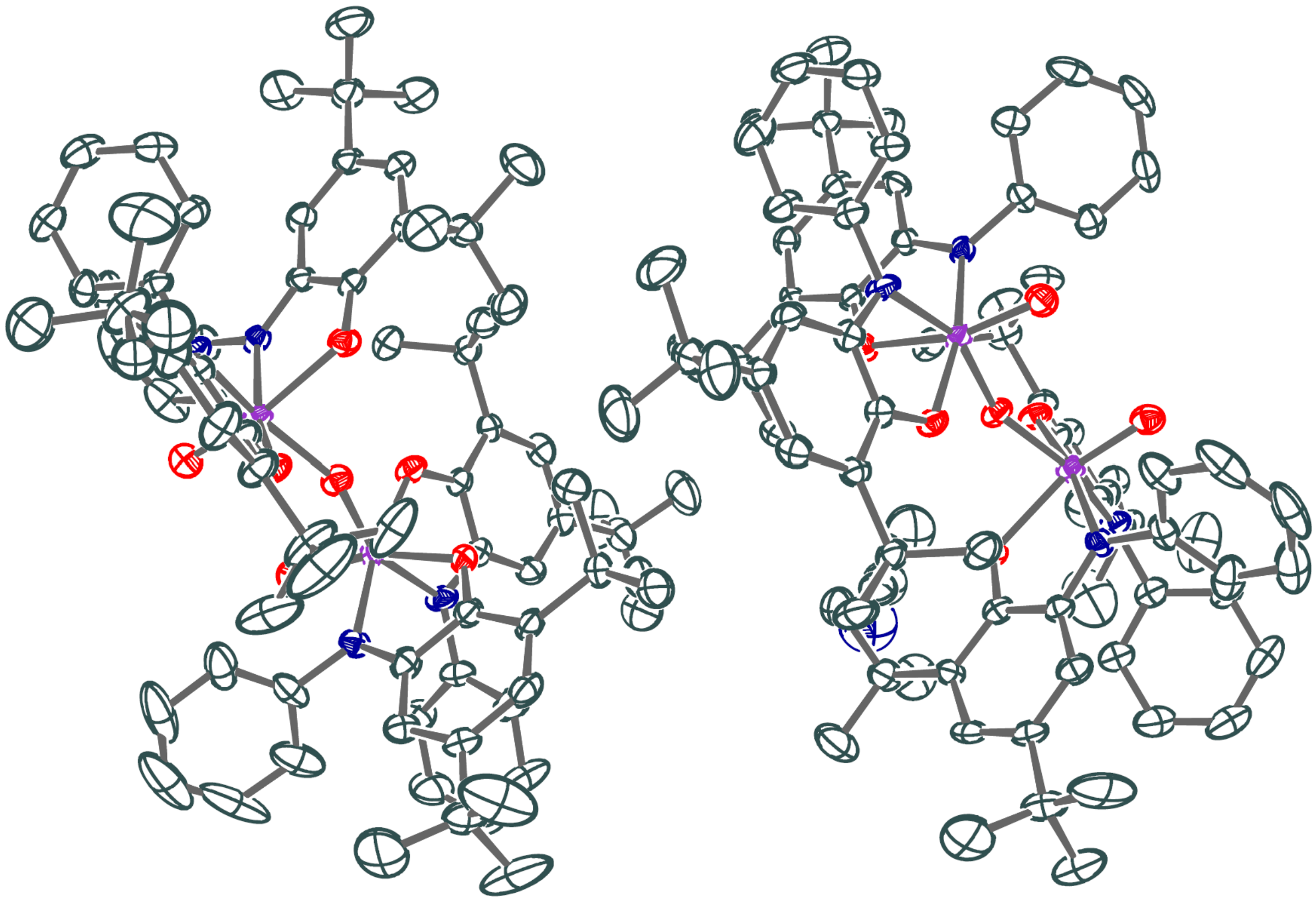

Experimental

A suitable crystal of $C_{80}H_{100}N_4O_7Re$ was selected and mounted on a loop with paratone oil on a Bruker APEX-II CCD diffractometer equipped with an Oxford Cryostream 800 low-temperature apparatus operating at 173K. Data were collected using MoKα radiation (microfocus sealed tube, 45 kV). Using Olex2[1], the structure was solved with the XT[2] structure solution program using Intrinsic Phasing and refined with the XL[3] refinement package using Least Squares minimisation.

Crystal data and structure refinement for CAL_9_227C.

Identification code cal_9_227c
Empirical formula C81 H111 N4.50 O7 Re2
Formula weight 1632.14
Temperature 173(2) K
Wavelength 0.71073 Å
Crystal system Triclinic
Space group P-1
Unit cell dimensions a = 13.979(3) Å a= 84.897(3)°.
b = 21.762(4) Å b= 83.355(3)°.
c = 26.405(5) Å g = 77.683(3)°.
Volume 7778(3) Å3
Z 4
Density (calculated) 1.394 Mg/m3

Absorption coefficient 3.164 mm-1
F(000) 3338
Crystal size 0.29 x 0.26 x 0.13 mm3
Theta range for data collection 1.19 to 29.34°.
Index ranges -19<=h<=19, -29<=k<=30, -36<=l<=36
Reflections collected 147107
Independent reflections 42370 [R(int) = 0.0665]
Completeness to theta = 29.34° 99.3 %
Absorption correction Numerical
Max. and min. transmission 0.6783 and 0.4565
Refinement method Full-matrix least-squares on F2
Data / restraints / parameters 42370 / 0 / 1685
Goodness-of-fit on F2 1.025
Final R indices [I>2sigma(I)] R1 = 0.0423, wR2 = 0.0937
R indices (all data) R1 = 0.0768, wR2 = 0.1154
Largest diff. peak and hole 1.675 and -1.281 e.Å-3

Atomic coordinates (x 104) and equivalent isotropic displacement parameters (Å2x 103) for CAL_9_227C. U(eq) is defined as one third of the trace of the orthogonalized Uij tensor.

	x	y	z	U(eq)
C(1)	3569(3)	-1176(2)	2799(2)	26(1)
C(2)	3364(4)	-1070(2)	2298(2)	34(1)
C(3)	2765(4)	-1425(3)	2121(2)	52(2)
C(4)	2403(4)	-1873(3)	2432(3)	58(2)
C(5)	2630(4)	-1988(3)	2928(3)	49(2)
C(6)	3213(3)	-1635(2)	3120(2)	32(1)
C(7)	4433(3)	-89(2)	3533(2)	20(1)
C(8)	3772(3)	-448(2)	3395(2)	22(1)
C(9)	2847(3)	-416(2)	3670(2)	25(1)
C(10)	2586(4)	-48(2)	4079(2)	27(1)
C(11)	3247(4)	317(2)	4195(2)	29(1)
C(12)	4153(3)	323(2)	3932(2)	23(1)
C(13)	1607(4)	-21(2)	4408(2)	35(1)
C(14)	1100(5)	-540(3)	4308(3)	62(2)
C(15)	944(4)	622(3)	4299(2)	53(2)
C(16)	1786(4)	-84(3)	4974(2)	53(2)
C(17)	4835(4)	735(2)	4065(2)	31(1)
C(18)	4325(5)	1191(3)	4472(2)	54(2)
C(19)	5764(4)	329(3)	4261(2)	46(2)
C(20)	5121(4)	1133(3)	3588(2)	42(1)
C(21)	5500(3)	-1745(2)	3679(2)	21(1)
C(22)	5110(4)	-1482(2)	4142(2)	28(1)
C(23)	4518(4)	-1788(3)	4482(2)	38(1)
C(24)	4325(4)	-2351(3)	4377(2)	40(1)
C(25)	4713(4)	-2615(2)	3921(2)	35(1)
C(26)	5289(3)	-2309(2)	3566(2)	26(1)
C(27)	7071(3)	-1454(2)	3417(2)	21(1)
C(28)	7555(3)	-1783(2)	3820(2)	26(1)
C(29)	8530(4)	-1759(2)	3856(2)	31(1)
C(30)	8991(4)	-1386(2)	3490(2)	37(1)
C(31)	8521(3)	-1037(2)	3083(2)	30(1)
C(32)	7555(3)	-1087(2)	3057(2)	24(1)
C(33)	9076(4)	-2152(3)	4282(2)	39(1)
C(34)	8510(5)	-2041(4)	4796(2)	75(2)

C(35)	9200(5)	-2855(3)	4179(2)	62(2)
C(36)	9972(10)	-1889(6)	4395(5)	55(4)
C(36A)	10180(8)	-2131(5)	4225(4)	37(3)
C(37)	9038(4)	-627(3)	2686(2)	46(2)
C(38)	8460(5)	50(3)	2685(3)	62(2)
C(39)	9098(5)	-890(4)	2161(2)	69(2)
C(40)	10083(4)	-624(4)	2809(3)	80(3)
C(41)	6954(3)	-479(3)	935(2)	33(1)
C(42)	7153(4)	-1078(3)	1162(3)	55(2)
C(43)	7516(5)	-1576(3)	865(4)	88(3)
C(44)	7667(5)	-1485(5)	350(4)	98(4)
C(45)	7438(5)	-889(5)	123(3)	85(3)
C(46)	7092(4)	-371(3)	411(2)	52(2)
C(47)	7283(3)	405(2)	1308(2)	25(1)
C(48)	8257(3)	323(2)	1081(2)	27(1)
C(49)	8805(4)	748(2)	1164(2)	28(1)
C(50)	8377(4)	1249(2)	1478(2)	31(1)
C(51)	7428(4)	1342(2)	1720(2)	28(1)
C(52)	6869(4)	908(2)	1627(2)	25(1)
C(53)	9853(4)	712(3)	903(2)	35(1)
C(54)	10315(5)	50(3)	735(3)	84(3)
C(55)	10517(5)	859(4)	1267(3)	87(3)
C(56)	9795(5)	1185(4)	447(3)	88(3)
C(57)	7001(4)	1888(2)	2060(2)	33(1)
C(58)	6574(4)	1636(3)	2582(2)	40(1)
C(59)	6189(5)	2347(2)	1800(2)	49(2)
C(60)	7799(5)	2233(3)	2160(2)	52(2)
C(61)	5169(4)	1098(2)	628(2)	30(1)
C(62)	5810(4)	1511(3)	590(2)	42(1)
C(63)	6327(5)	1624(3)	121(2)	61(2)
C(64)	6185(5)	1344(3)	-298(2)	57(2)
C(65)	5532(5)	956(3)	-266(2)	53(2)
C(66)	5037(4)	818(3)	195(2)	41(1)
C(67)	3749(3)	1320(2)	1263(2)	24(1)
C(68)	3269(4)	1857(2)	1001(2)	29(1)
C(69)	2388(4)	2190(2)	1223(2)	29(1)
C(70)	2007(4)	1972(2)	1701(2)	29(1)
C(71)	2478(3)	1437(2)	1980(2)	23(1)
C(72)	3374(3)	1124(2)	1745(2)	22(1)
C(73)	2057(3)	1206(2)	2510(2)	26(1)
C(74)	1933(4)	523(2)	2503(2)	35(1)
C(75)	2776(4)	1226(2)	2908(2)	33(1)
C(76)	1054(4)	1615(3)	2684(2)	39(1)
C(77)	1844(4)	2817(2)	972(2)	39(1)
C(78)	849(6)	2741(3)	836(3)	83(3)
C(79)	1695(6)	3339(3)	1330(2)	67(2)
C(80)	2437(6)	3020(3)	480(2)	72(2)
C(81)	3248(3)	5467(2)	4229(2)	22(1)
C(82)	3142(3)	6092(2)	4054(2)	28(1)
C(83)	2733(4)	6556(2)	4385(2)	38(1)
C(84)	2476(4)	6403(3)	4890(2)	43(2)
C(85)	2620(4)	5780(3)	5070(2)	39(1)
C(86)	3002(3)	5300(2)	4740(2)	29(1)
C(87)	2926(3)	4643(2)	3776(2)	20(1)
C(88)	1973(3)	4667(2)	4016(2)	24(1)
C(89)	1423(3)	4268(2)	3882(2)	24(1)

C(90)	1830(3)	3852(2)	3494(2)	26(1)
C(91)	2757(3)	3817(2)	3238(2)	21(1)
C(92)	3331(3)	4209(2)	3401(2)	21(1)
C(93)	397(4)	4276(2)	4158(2)	30(1)
C(94)	-274(4)	4916(3)	4046(2)	43(1)
C(95)	475(4)	4166(3)	4735(2)	48(2)
C(96)	-75(4)	3770(3)	3997(2)	53(2)
C(97)	3142(4)	3374(2)	2810(2)	29(1)
C(98)	2362(4)	3034(3)	2682(2)	40(1)
C(99)	4035(4)	2877(2)	2966(2)	39(1)
C(100)	3450(4)	3768(3)	2322(2)	41(1)
C(101)	4883(3)	3779(2)	4365(2)	24(1)
C(102)	4798(3)	4009(2)	4850(2)	27(1)
C(103)	4203(4)	3782(2)	5242(2)	37(1)
C(104)	3678(4)	3345(3)	5163(2)	43(1)
C(105)	3773(4)	3104(3)	4686(2)	42(1)
C(106)	4382(4)	3320(2)	4287(2)	32(1)
C(107)	6385(3)	3659(2)	3788(2)	22(1)
C(108)	6841(3)	3080(2)	4005(2)	25(1)
C(109)	7739(3)	2772(2)	3784(2)	22(1)
C(110)	8172(3)	3061(2)	3347(2)	24(1)
C(111)	7739(3)	3636(2)	3115(2)	20(1)
C(112)	6820(3)	3925(2)	3340(2)	20(1)
C(113)	8230(3)	3941(2)	2636(2)	23(1)
C(114)	9231(4)	3547(2)	2458(2)	37(1)
C(115)	8376(4)	4592(2)	2749(2)	34(1)
C(116)	7573(4)	4022(2)	2197(2)	31(1)
C(117)	9286(4)	2197(3)	4155(2)	52(2)
C(118)	8266(4)	2134(2)	4004(2)	29(1)
C(119)	7721(5)	1904(3)	4490(2)	61(2)
C(120)	8450(5)	1646(3)	3612(2)	58(2)
C(121)	6594(3)	6409(2)	2502(2)	25(1)
C(122)	6807(4)	6210(3)	2997(2)	37(1)
C(123)	7369(4)	6536(3)	3234(2)	52(2)
C(124)	7666(4)	7053(3)	2994(3)	56(2)
C(125)	7425(4)	7257(3)	2510(3)	52(2)
C(126)	6892(4)	6930(2)	2256(2)	38(1)
C(127)	6556(3)	5725(2)	1845(2)	21(1)
C(128)	5968(3)	5373(2)	1639(2)	22(1)
C(129)	6349(3)	5004(2)	1218(2)	23(1)
C(130)	7294(4)	5047(2)	1023(2)	28(1)
C(131)	7907(3)	5384(2)	1227(2)	25(1)
C(132)	7528(3)	5718(2)	1643(2)	26(1)
C(133)	8945(4)	5377(3)	970(2)	36(1)
C(134)	9468(4)	4707(3)	849(3)	65(2)
C(135)	9561(4)	5635(4)	1310(2)	71(2)
C(136)	8876(5)	5783(3)	471(2)	56(2)
C(137)	6370(5)	4182(3)	588(2)	61(2)
C(138)	5754(4)	4600(2)	994(2)	31(1)
C(139)	5342(5)	4178(3)	1423(2)	46(2)
C(140)	4904(5)	5032(3)	743(2)	58(2)
C(141)	4669(3)	6934(2)	1501(2)	26(1)
C(142)	4979(4)	6686(3)	1038(2)	40(1)
C(143)	5518(5)	7000(3)	666(2)	52(2)
C(144)	5737(4)	7554(3)	764(2)	51(2)
C(145)	5410(4)	7815(3)	1225(2)	47(2)

C(146)	4886(4)	7500(2)	1602(2)	31(1)
C(147)	3091(3)	6701(2)	1833(2)	24(1)
C(148)	2574(4)	7079(2)	1456(2)	30(1)
C(149)	1565(4)	7111(2)	1471(2)	35(1)
C(150)	1110(4)	6777(2)	1865(2)	33(1)
C(151)	1603(3)	6388(2)	2243(2)	25(1)
C(152)	2620(3)	6346(2)	2205(2)	22(1)
C(153)	1000(4)	7515(3)	1054(2)	50(2)
C(154)	1421(7)	7235(4)	539(3)	113(4)
C(155)	1163(6)	8174(3)	1020(3)	90(3)
C(156)	-136(8)	7600(5)	1184(4)	38(3)
C(161)	60(12)	7355(8)	1004(6)	76(5)
C(157)	1085(3)	6037(2)	2674(2)	30(1)
C(158)	1312(4)	6218(3)	3191(2)	44(1)
C(159)	-27(4)	6198(3)	2670(2)	48(2)
C(160)	1430(4)	5330(2)	2638(2)	41(1)
C(1S)	2398(6)	5276(4)	1304(3)	89(3)
C(2S)	2260(6)	5173(4)	793(3)	76(2)
N(1)	4173(3)	-802(2)	2993(1)	21(1)
N(2)	6115(3)	-1435(2)	3318(1)	22(1)
N(3)	6617(3)	41(2)	1252(1)	23(1)
N(4)	4648(3)	945(2)	1104(1)	28(1)
N(5)	3598(3)	4990(2)	3870(1)	20(1)
N(6)	5485(3)	4028(2)	3957(1)	23(1)
N(7)	6054(3)	6051(2)	2250(1)	21(1)
N(8)	4076(3)	6631(2)	1885(1)	25(1)
N(1S)	2114(7)	5124(4)	391(4)	135(3)
O(1)	5283(2)	-177(1)	3270(1)	23(1)
O(2)	6981(2)	-787(1)	2689(1)	23(1)
O(3)	5948(2)	927(1)	1796(1)	23(1)
O(4)	3973(2)	628(1)	1973(1)	24(1)
O(5)	5513(2)	-142(1)	2252(1)	25(1)
O(6)	5505(2)	-1396(2)	2333(1)	29(1)
O(7)	5021(2)	-370(2)	1324(1)	31(1)
O(8)	4239(2)	4206(1)	3226(1)	23(1)
O(9)	6258(2)	4466(1)	3149(1)	20(1)
O(10)	5078(2)	5432(1)	1863(1)	22(1)
O(11)	3237(2)	5964(1)	2516(1)	23(1)
O(12)	4765(2)	5292(1)	2893(1)	23(1)
O(13)	5216(2)	5357(2)	3872(1)	26(1)
O(14)	4640(2)	6565(2)	2877(1)	28(1)
Re(1)	5608(1)	-846(1)	2736(1)	19(1)
Re(2)	5234(1)	260(1)	1607(1)	21(1)
Re(3)	4978(1)	4801(1)	3512(1)	19(1)
Re(4)	4618(1)	6032(1)	2449(1)	19(1)

Bond lengths [Å] and angles [°] for CAL_9_227C.

C(1)-C(2)	1.373(7)
C(1)-C(6)	1.388(7)
C(1)-N(1)	1.451(5)
C(2)-C(3)	1.396(7)
C(2)-H(2A)	0.9500
C(3)-C(4)	1.360(9)
C(3)-H(3A)	0.9500
C(4)-C(5)	1.374(9)
C(4)-H(4A)	0.9500
C(5)-C(6)	1.394(7)
C(5)-H(5A)	0.9500
C(6)-H(6A)	0.9500
C(7)-O(1)	1.291(5)
C(7)-C(12)	1.410(6)
C(7)-C(8)	1.427(6)
C(8)-N(1)	1.362(5)

C(8)-C(9)	1.398(6)
C(9)-C(10)	1.369(6)
C(9)-H(9A)	0.9500
C(10)-C(11)	1.414(7)
C(10)-C(13)	1.527(7)
C(11)-C(12)	1.375(6)
C(11)-H(11A)	0.9500
C(12)-C(17)	1.528(6)
C(13)-C(14)	1.512(8)
C(13)-C(15)	1.528(7)
C(13)-C(16)	1.533(8)
C(14)-H(14A)	0.9800
C(14)-H(14B)	0.9800
C(14)-H(14C)	0.9800
C(15)-H(15A)	0.9800
C(15)-H(15B)	0.9800
C(15)-H(15C)	0.9800
C(16)-H(16A)	0.9800
C(16)-H(16B)	0.9800
C(16)-H(16C)	0.9800
C(17)-C(19)	1.525(7)
C(17)-C(20)	1.526(7)
C(17)-C(18)	1.537(7)
C(18)-H(18A)	0.9800
C(18)-H(18B)	0.9800
C(18)-H(18C)	0.9800
C(19)-H(19A)	0.9800
C(19)-H(19B)	0.9800
C(19)-H(19C)	0.9800
C(20)-H(20A)	0.9800
C(20)-H(20B)	0.9800
C(20)-H(20C)	0.9800
C(21)-C(26)	1.386(6)
C(21)-C(22)	1.399(6)
C(21)-N(2)	1.431(5)
C(22)-C(23)	1.375(7)
C(22)-H(22A)	0.9500
C(23)-C(24)	1.365(7)
C(23)-H(23A)	0.9500
C(24)-C(25)	1.383(7)
C(24)-H(24A)	0.9500
C(25)-C(26)	1.384(7)
C(25)-H(25A)	0.9500
C(26)-H(26A)	0.9500
C(27)-N(2)	1.382(5)
C(27)-C(28)	1.387(6)
C(27)-C(32)	1.395(6)
C(28)-C(29)	1.390(6)
C(28)-H(28A)	0.9500
C(29)-C(30)	1.397(7)
C(29)-C(33)	1.526(6)
C(30)-C(31)	1.404(7)
C(30)-H(30A)	0.9500
C(31)-C(32)	1.386(6)
C(31)-C(37)	1.533(7)
C(32)-O(2)	1.364(5)
C(33)-C(34)	1.502(8)
C(33)-C(36A)	1.544(12)
C(33)-C(35)	1.549(8)
C(33)-C(36)	1.552(14)
C(34)-H(34A)	0.9800
C(34)-H(34B)	0.9800
C(34)-H(34C)	0.9800
C(35)-H(35A)	0.9800
C(35)-H(35B)	0.9800
C(35)-H(35C)	0.9800
C(36)-H(36A)	0.9800
C(36)-H(36B)	0.9800
C(36)-H(36C)	0.9800
C(36A)-H(36D)	0.9800
C(36A)-H(36E)	0.9800
C(36A)-H(36F)	0.9800
C(37)-C(38)	1.522(8)
C(37)-C(39)	1.533(9)
C(37)-C(40)	1.534(8)
C(38)-H(38A)	0.9800
C(38)-H(38B)	0.9800
C(38)-H(38C)	0.9800
C(39)-H(39A)	0.9800
C(39)-H(39B)	0.9800
C(39)-H(39C)	0.9800
C(40)-H(40A)	0.9800
C(40)-H(40B)	0.9800
C(40)-H(40C)	0.9800
C(41)-C(42)	1.372(8)
C(41)-C(46)	1.381(8)
C(41)-N(3)	1.434(6)
C(42)-C(43)	1.369(9)
C(42)-H(42A)	0.9500
C(43)-C(44)	1.355(13)
C(43)-H(43A)	0.9500
C(44)-C(45)	1.370(12)
C(44)-H(44A)	0.9500
C(45)-C(46)	1.385(9)
C(45)-H(45A)	0.9500
C(46)-H(46A)	0.9500
C(47)-N(3)	1.373(5)
C(47)-C(48)	1.404(6)
C(47)-C(52)	1.422(6)
C(48)-C(49)	1.367(6)
C(48)-H(48A)	0.9500
C(49)-C(50)	1.415(7)
C(49)-C(53)	1.534(7)
C(50)-C(51)	1.386(7)
C(50)-H(50A)	0.9500
C(51)-C(52)	1.404(6)
C(51)-C(57)	1.529(7)
C(52)-O(3)	1.306(5)
C(53)-C(55)	1.511(7)
C(53)-C(56)	1.511(8)
C(53)-C(54)	1.528(8)
C(54)-H(54A)	0.9800

C(54)-H(54B)	0.9800
C(54)-H(54C)	0.9800
C(55)-H(55A)	0.9800
C(55)-H(55B)	0.9800
C(55)-H(55C)	0.9800
C(56)-H(56A)	0.9800
C(56)-H(56B)	0.9800
C(56)-H(56C)	0.9800
C(57)-C(59)	1.527(7)
C(57)-C(60)	1.529(7)
C(57)-C(58)	1.539(7)
C(58)-H(58A)	0.9800
C(58)-H(58B)	0.9800
C(58)-H(58C)	0.9800
C(59)-H(59A)	0.9800
C(59)-H(59B)	0.9800
C(59)-H(59C)	0.9800
C(60)-H(60A)	0.9800
C(60)-H(60B)	0.9800
C(60)-H(60C)	0.9800
C(61)-C(62)	1.389(7)
C(61)-C(66)	1.390(7)
C(61)-N(4)	1.428(6)
C(62)-C(63)	1.388(8)
C(62)-H(62A)	0.9500
C(63)-C(64)	1.363(9)
C(63)-H(63A)	0.9500
C(64)-C(65)	1.361(9)
C(64)-H(64A)	0.9500
C(65)-C(66)	1.371(8)
C(65)-H(65A)	0.9500
C(66)-H(66A)	0.9500
C(67)-C(72)	1.386(6)
C(67)-N(4)	1.387(6)
C(67)-C(68)	1.388(6)
C(68)-C(69)	1.384(7)
C(68)-H(68A)	0.9500
C(69)-C(70)	1.396(7)
C(69)-C(77)	1.546(7)
C(70)-C(71)	1.401(6)
C(70)-H(70A)	0.9500
C(71)-C(72)	1.396(6)
C(71)-C(73)	1.540(6)
C(72)-O(4)	1.357(5)
C(73)-C(74)	1.534(6)
C(73)-C(76)	1.535(6)
C(73)-C(75)	1.544(6)
C(74)-H(74A)	0.9800
C(74)-H(74B)	0.9800
C(74)-H(74C)	0.9800
C(75)-H(75A)	0.9800
C(75)-H(75B)	0.9800
C(75)-H(75C)	0.9800
C(76)-H(76A)	0.9800
C(76)-H(76B)	0.9800
C(76)-H(76C)	0.9800
C(77)-C(79)	1.507(8)
C(77)-C(78)	1.522(9)
C(77)-C(80)	1.542(8)
C(78)-H(78A)	0.9800
C(78)-H(78B)	0.9800
C(78)-H(78C)	0.9800
C(79)-H(79A)	0.9800
C(79)-H(79B)	0.9800
C(79)-H(79C)	0.9800
C(80)-H(80A)	0.9800
C(80)-H(80B)	0.9800
C(80)-H(80C)	0.9800
C(81)-C(82)	1.382(6)
C(81)-C(86)	1.388(6)
C(81)-N(5)	1.439(5)
C(82)-C(83)	1.380(7)
C(82)-H(82A)	0.9500
C(83)-C(84)	1.371(8)
C(83)-H(83A)	0.9500
C(84)-C(85)	1.376(8)
C(84)-H(84A)	0.9500
C(85)-C(86)	1.397(7)
C(85)-H(85A)	0.9500
C(86)-H(86A)	0.9500
C(87)-N(5)	1.380(5)
C(87)-C(88)	1.399(6)
C(87)-C(92)	1.415(6)
C(88)-C(89)	1.369(6)
C(88)-H(88A)	0.9500
C(89)-C(90)	1.415(6)
C(89)-C(93)	1.528(6)
C(90)-C(91)	1.381(6)
C(90)-H(90A)	0.9500
C(91)-C(92)	1.413(6)
C(91)-C(97)	1.525(6)
C(92)-O(8)	1.298(5)
C(93)-C(96)	1.513(7)
C(93)-C(94)	1.529(7)
C(93)-C(95)	1.535(7)
C(94)-H(94A)	0.9800
C(94)-H(94B)	0.9800
C(94)-H(94C)	0.9800
C(95)-H(95A)	0.9800
C(95)-H(95B)	0.9800
C(95)-H(95C)	0.9800
C(96)-H(96A)	0.9800
C(96)-H(96B)	0.9800
C(96)-H(96C)	0.9800
C(97)-C(98)	1.526(7)
C(97)-C(99)	1.536(7)
C(97)-C(100)	1.553(7)
C(98)-H(98A)	0.9800
C(98)-H(98B)	0.9800
C(98)-H(98C)	0.9800
C(99)-H(99A)	0.9800
C(99)-H(99B)	0.9800

C(99)-H(99C)	0.9800
C(100)-H(10A)	0.9800
C(100)-H(10B)	0.9800
C(100)-H(10C)	0.9800
C(101)-C(106)	1.378(7)
C(101)-C(102)	1.399(6)
C(101)-N(6)	1.431(6)
C(102)-C(103)	1.376(7)
C(102)-H(10D)	0.9500
C(103)-C(104)	1.362(8)
C(103)-H(10E)	0.9500
C(104)-C(105)	1.388(8)
C(104)-H(10F)	0.9500
C(105)-C(106)	1.390(7)
C(105)-H(10G)	0.9500
C(106)-H(10H)	0.9500
C(107)-N(6)	1.389(6)
C(107)-C(108)	1.391(6)
C(107)-C(112)	1.402(6)
C(108)-C(109)	1.381(6)
C(108)-H(10I)	0.9500
C(109)-C(110)	1.401(6)
C(109)-C(118)	1.528(6)
C(110)-C(111)	1.392(6)
C(110)-H(11B)	0.9500
C(111)-C(112)	1.393(6)
C(111)-C(113)	1.533(6)
C(112)-O(9)	1.360(5)
C(113)-C(114)	1.525(6)
C(113)-C(115)	1.533(6)
C(113)-C(116)	1.536(6)
C(114)-H(11C)	0.9800
C(114)-H(11D)	0.9800
C(114)-H(11E)	0.9800
C(115)-H(11F)	0.9800
C(115)-H(11G)	0.9800
C(115)-H(11H)	0.9800
C(116)-H(11I)	0.9800
C(116)-H(11J)	0.9800
C(116)-H(11K)	0.9800
C(117)-C(118)	1.560(7)
C(117)-H(11L)	0.9800
C(117)-H(11M)	0.9800
C(117)-H(11N)	0.9800
C(118)-C(120)	1.510(7)
C(118)-C(119)	1.515(7)
C(119)-H(11O)	0.9800
C(119)-H(11P)	0.9800
C(119)-H(11Q)	0.9800
C(120)-H(12A)	0.9800
C(120)-H(12B)	0.9800
C(120)-H(12C)	0.9800
C(121)-C(126)	1.373(7)
C(121)-C(122)	1.386(7)
C(121)-N(7)	1.441(5)
C(122)-C(123)	1.395(7)
C(122)-H(12D)	0.9500
C(123)-C(124)	1.360(9)
C(123)-H(12E)	0.9500
C(124)-C(125)	1.368(9)
C(124)-H(12F)	0.9500
C(125)-C(126)	1.393(7)
C(125)-H(12G)	0.9500
C(126)-H(12H)	0.9500
C(127)-N(7)	1.375(5)
C(127)-C(132)	1.399(6)
C(127)-C(128)	1.415(6)
C(128)-O(10)	1.300(5)
C(128)-C(129)	1.415(6)
C(129)-C(130)	1.380(6)
C(129)-C(138)	1.529(6)
C(130)-C(131)	1.418(7)
C(130)-H(13A)	0.9500
C(131)-C(132)	1.362(6)
C(131)-C(133)	1.527(6)
C(132)-H(13B)	0.9500
C(133)-C(136)	1.519(7)
C(133)-C(135)	1.528(8)
C(133)-C(134)	1.528(8)
C(134)-H(13C)	0.9800
C(134)-H(13D)	0.9800
C(134)-H(13E)	0.9800
C(135)-H(13F)	0.9800
C(135)-H(13G)	0.9800
C(135)-H(13H)	0.9800
C(136)-H(13I)	0.9800
C(136)-H(13J)	0.9800
C(136)-H(13K)	0.9800
C(137)-C(138)	1.530(7)
C(137)-H(13L)	0.9800
C(137)-H(13M)	0.9800
C(137)-H(13N)	0.9800
C(138)-C(140)	1.526(7)
C(138)-C(139)	1.532(7)
C(139)-H(13O)	0.9800
C(139)-H(13P)	0.9800
C(139)-H(13Q)	0.9800
C(140)-H(14D)	0.9800
C(140)-H(14E)	0.9800
C(140)-H(14F)	0.9800
C(141)-C(142)	1.368(7)
C(141)-C(146)	1.383(7)
C(141)-N(8)	1.438(6)
C(142)-C(143)	1.389(7)
C(142)-H(14G)	0.9500
C(143)-C(144)	1.357(8)
C(143)-H(14H)	0.9500
C(144)-C(145)	1.378(8)
C(144)-H(14I)	0.9500
C(145)-C(146)	1.386(7)
C(145)-H(14J)	0.9500
C(146)-H(14K)	0.9500

C(147)-N(8)	1.375(6)
C(147)-C(152)	1.387(6)
C(147)-C(148)	1.399(6)
C(148)-C(149)	1.393(7)
C(148)-H(14L)	0.9500
C(149)-C(150)	1.388(7)
C(149)-C(153)	1.531(7)
C(150)-C(151)	1.395(6)
C(150)-H(15D)	0.9500
C(151)-C(152)	1.398(6)
C(151)-C(157)	1.515(7)
C(152)-O(11)	1.360(5)
C(153)-C(161)	1.452(16)
C(153)-C(155)	1.493(9)
C(153)-C(154)	1.542(10)
C(153)-C(156)	1.559(12)
C(154)-H(15E)	0.9800
C(154)-H(15F)	0.9800
C(154)-H(15G)	0.9800
C(155)-H(15H)	0.9800
C(155)-H(15I)	0.9800
C(155)-H(15J)	0.9800
C(156)-H(15K)	0.9800
C(156)-H(15L)	0.9800
C(156)-H(15M)	0.9800
C(161)-H(16D)	0.9800
C(161)-H(16E)	0.9800
C(161)-H(16F)	0.9800
C(157)-C(159)	1.519(7)
C(157)-C(160)	1.519(7)
C(157)-C(158)	1.537(7)
C(158)-H(15N)	0.9800
C(158)-H(15O)	0.9800
C(158)-H(15P)	0.9800
C(159)-H(15Q)	0.9800
C(159)-H(15R)	0.9800
C(159)-H(15S)	0.9800
C(160)-H(16G)	0.9800
C(160)-H(16H)	0.9800
C(160)-H(16I)	0.9800
C(1S)-C(2S)	1.426(11)
C(2S)-N(1S)	1.123(10)
N(1)-Re(1)	2.026(4)
N(2)-Re(1)	2.005(3)
N(3)-Re(2)	2.027(4)
N(4)-Re(2)	2.010(4)
N(5)-Re(3)	2.023(4)
N(6)-Re(3)	2.011(4)
N(7)-Re(4)	2.024(4)
N(8)-Re(4)	1.995(4)
O(1)-Re(1)	2.062(3)
O(2)-Re(1)	1.940(3)
O(3)-Re(2)	2.051(3)
O(4)-Re(2)	1.954(3)
O(5)-Re(2)	1.885(3)
O(5)-Re(1)	1.898(3)
O(6)-Re(1)	1.709(3)
O(7)-Re(2)	1.713(3)
O(8)-Re(3)	2.059(3)
O(9)-Re(3)	1.955(3)
O(10)-Re(4)	2.070(3)
O(11)-Re(4)	1.954(3)
O(12)-Re(3)	1.888(3)
O(12)-Re(4)	1.896(3)
O(13)-Re(3)	1.710(3)
O(14)-Re(4)	1.698(3)
C(2)-C(1)-C(6)	121.3(5)
C(2)-C(1)-N(1)	119.1(4)
C(6)-C(1)-N(1)	119.6(4)
C(1)-C(2)-C(3)	118.3(6)
C(1)-C(2)-H(2A)	120.8
C(3)-C(2)-H(2A)	120.8
C(4)-C(3)-C(2)	121.2(6)
C(4)-C(3)-H(3A)	119.4
C(2)-C(3)-H(3A)	119.4
C(3)-C(4)-C(5)	120.2(5)
C(3)-C(4)-H(4A)	119.9
C(5)-C(4)-H(4A)	119.9
C(4)-C(5)-C(6)	120.0(6)
C(4)-C(5)-H(5A)	120.0
C(6)-C(5)-H(5A)	120.0
C(1)-C(6)-C(5)	118.9(5)
C(1)-C(6)-H(6A)	120.5
C(5)-C(6)-H(6A)	120.5
O(1)-C(7)-C(12)	124.1(4)
O(1)-C(7)-C(8)	115.0(4)
C(12)-C(7)-C(8)	120.9(4)
N(1)-C(8)-C(9)	128.4(4)
N(1)-C(8)-C(7)	111.6(4)
C(9)-C(8)-C(7)	120.0(4)
C(10)-C(9)-C(8)	119.9(4)
C(10)-C(9)-H(9A)	120.0
C(8)-C(9)-H(9A)	120.0
C(9)-C(10)-C(11)	118.6(4)
C(9)-C(10)-C(13)	122.0(5)
C(11)-C(10)-C(13)	119.4(4)
C(12)-C(11)-C(10)	124.7(5)
C(12)-C(11)-H(11A)	117.7
C(10)-C(11)-H(11A)	117.7
C(11)-C(12)-C(7)	115.7(4)
C(11)-C(12)-C(17)	122.9(4)
C(7)-C(12)-C(17)	121.3(4)
C(14)-C(13)-C(10)	112.3(4)
C(14)-C(13)-C(15)	109.9(5)
C(10)-C(13)-C(15)	108.5(4)
C(14)-C(13)-C(16)	108.5(5)
C(10)-C(13)-C(16)	109.4(4)
C(15)-C(13)-C(16)	108.2(5)
C(13)-C(14)-H(14A)	109.5
C(13)-C(14)-H(14B)	109.5
H(14A)-C(14)-H(14B)	109.5

C(13)-C(14)-H(14C)	109.5
H(14A)-C(14)-H(14C)	109.5
H(14B)-C(14)-H(14C)	109.5
C(13)-C(15)-H(15A)	109.5
C(13)-C(15)-H(15B)	109.5
H(15A)-C(15)-H(15B)	109.5
C(13)-C(15)-H(15C)	109.5
H(15A)-C(15)-H(15C)	109.5
H(15B)-C(15)-H(15C)	109.5
C(13)-C(16)-H(16A)	109.5
C(13)-C(16)-H(16B)	109.5
H(16A)-C(16)-H(16B)	109.5
C(13)-C(16)-H(16C)	109.5
H(16A)-C(16)-H(16C)	109.5
H(16B)-C(16)-H(16C)	109.5
C(19)-C(17)-C(20)	109.2(5)
C(19)-C(17)-C(12)	110.6(4)
C(20)-C(17)-C(12)	109.3(4)
C(19)-C(17)-C(18)	108.8(4)
C(20)-C(17)-C(18)	107.5(4)
C(12)-C(17)-C(18)	111.4(4)
C(17)-C(18)-H(18A)	109.5
C(17)-C(18)-H(18B)	109.5
H(18A)-C(18)-H(18B)	109.5
C(17)-C(18)-H(18C)	109.5
H(18A)-C(18)-H(18C)	109.5
H(18B)-C(18)-H(18C)	109.5
C(17)-C(19)-H(19A)	109.5
C(17)-C(19)-H(19B)	109.5
H(19A)-C(19)-H(19B)	109.5
C(17)-C(19)-H(19C)	109.5
H(19A)-C(19)-H(19C)	109.5
H(19B)-C(19)-H(19C)	109.5
C(17)-C(20)-H(20A)	109.5
C(17)-C(20)-H(20B)	109.5
H(20A)-C(20)-H(20B)	109.5
C(17)-C(20)-H(20C)	109.5
H(20A)-C(20)-H(20C)	109.5
H(20B)-C(20)-H(20C)	109.5
C(26)-C(21)-C(22)	120.0(4)
C(26)-C(21)-N(2)	119.5(4)
C(22)-C(21)-N(2)	120.5(4)
C(23)-C(22)-C(21)	119.3(5)
C(23)-C(22)-H(22A)	120.3
C(21)-C(22)-H(22A)	120.3
C(24)-C(23)-C(22)	120.9(5)
C(24)-C(23)-H(23A)	119.6
C(22)-C(23)-H(23A)	119.6
C(23)-C(24)-C(25)	120.1(5)
C(23)-C(24)-H(24A)	119.9
C(25)-C(24)-H(24A)	119.9
C(24)-C(25)-C(26)	120.3(5)
C(24)-C(25)-H(25A)	119.9
C(26)-C(25)-H(25A)	119.9
C(25)-C(26)-C(21)	119.3(5)
C(25)-C(26)-H(26A)	120.3
C(21)-C(26)-H(26A)	120.3
N(2)-C(27)-C(28)	127.5(4)
N(2)-C(27)-C(32)	112.6(4)
C(28)-C(27)-C(32)	120.0(4)
C(27)-C(28)-C(29)	119.9(4)
C(27)-C(28)-H(28A)	120.1
C(29)-C(28)-H(28A)	120.1
C(28)-C(29)-C(30)	118.6(4)
C(28)-C(29)-C(33)	119.6(4)
C(30)-C(29)-C(33)	121.7(4)
C(29)-C(30)-C(31)	123.2(5)
C(29)-C(30)-H(30A)	118.4
C(31)-C(30)-H(30A)	118.4
C(32)-C(31)-C(30)	116.0(4)
C(32)-C(31)-C(37)	121.6(4)
C(30)-C(31)-C(37)	122.4(4)
O(2)-C(32)-C(31)	124.5(4)
O(2)-C(32)-C(27)	113.1(4)
C(31)-C(32)-C(27)	122.4(4)
C(34)-C(33)-C(29)	111.5(5)
C(34)-C(33)-C(36A)	117.9(6)
C(29)-C(33)-C(36A)	112.5(6)
C(34)-C(33)-C(35)	108.8(5)
C(29)-C(33)-C(35)	107.7(4)
C(36A)-C(33)-C(35)	97.2(6)
C(34)-C(33)-C(36)	95.7(7)
C(29)-C(33)-C(36)	112.2(6)
C(36A)-C(33)-C(36)	26.7(6)
C(35)-C(33)-C(36)	120.4(7)
C(33)-C(34)-H(34A)	109.5
C(33)-C(34)-H(34B)	109.5
H(34A)-C(34)-H(34B)	109.5
C(33)-C(34)-H(34C)	109.5
H(34A)-C(34)-H(34C)	109.5
H(34B)-C(34)-H(34C)	109.5
C(33)-C(35)-H(35A)	109.5
C(33)-C(35)-H(35B)	109.5
H(35A)-C(35)-H(35B)	109.5
C(33)-C(35)-H(35C)	109.5
H(35A)-C(35)-H(35C)	109.5
H(35B)-C(35)-H(35C)	109.5
C(33)-C(36)-H(36A)	109.5
C(33)-C(36)-H(36B)	109.5
H(36A)-C(36)-H(36B)	109.5
C(33)-C(36)-H(36C)	109.5
H(36A)-C(36)-H(36C)	109.5
H(36B)-C(36)-H(36C)	109.5
C(33)-C(36A)-H(36D)	109.5
C(33)-C(36A)-H(36E)	109.5
H(36D)-C(36A)-H(36E)	109.5
C(33)-C(36A)-H(36F)	109.5
H(36D)-C(36A)-H(36F)	109.5
H(36E)-C(36A)-H(36F)	109.5
C(38)-C(37)-C(39)	110.9(5)
C(38)-C(37)-C(31)	109.2(5)
C(39)-C(37)-C(31)	108.5(5)

C(38)-C(37)-C(40)	107.5(5)
C(39)-C(37)-C(40)	108.8(6)
C(31)-C(37)-C(40)	111.9(4)
C(37)-C(38)-H(38A)	109.5
C(37)-C(38)-H(38B)	109.5
H(38A)-C(38)-H(38B)	109.5
C(37)-C(38)-H(38C)	109.5
H(38A)-C(38)-H(38C)	109.5
H(38B)-C(38)-H(38C)	109.5
C(37)-C(39)-H(39A)	109.5
C(37)-C(39)-H(39B)	109.5
H(39A)-C(39)-H(39B)	109.5
C(37)-C(39)-H(39C)	109.5
H(39A)-C(39)-H(39C)	109.5
H(39B)-C(39)-H(39C)	109.5
C(37)-C(40)-H(40A)	109.5
C(37)-C(40)-H(40B)	109.5
H(40A)-C(40)-H(40B)	109.5
C(37)-C(40)-H(40C)	109.5
H(40A)-C(40)-H(40C)	109.5
H(40B)-C(40)-H(40C)	109.5
C(42)-C(41)-C(46)	121.2(6)
C(42)-C(41)-N(3)	118.9(5)
C(46)-C(41)-N(3)	119.9(5)
C(43)-C(42)-C(41)	119.4(7)
C(43)-C(42)-H(42A)	120.3
C(41)-C(42)-H(42A)	120.3
C(44)-C(43)-C(42)	120.9(9)
C(44)-C(43)-H(43A)	119.6
C(42)-C(43)-H(43A)	119.6
C(43)-C(44)-C(45)	119.7(8)
C(43)-C(44)-H(44A)	120.2
C(45)-C(44)-H(44A)	120.2
C(44)-C(45)-C(46)	121.2(8)
C(44)-C(45)-H(45A)	119.4
C(46)-C(45)-H(45A)	119.4
C(41)-C(46)-C(45)	117.6(7)
C(41)-C(46)-H(46A)	121.2
C(45)-C(46)-H(46A)	121.2
N(3)-C(47)-C(48)	126.5(4)
N(3)-C(47)-C(52)	111.8(4)
C(48)-C(47)-C(52)	121.7(4)
C(49)-C(48)-C(47)	118.4(5)
C(49)-C(48)-H(48A)	120.8
C(47)-C(48)-H(48A)	120.8
C(48)-C(49)-C(50)	119.1(4)
C(48)-C(49)-C(53)	121.3(5)
C(50)-C(49)-C(53)	119.5(4)
C(51)-C(50)-C(49)	124.7(4)
C(51)-C(50)-H(50A)	117.7
C(49)-C(50)-H(50A)	117.7
C(50)-C(51)-C(52)	115.7(4)
C(50)-C(51)-C(57)	122.8(4)
C(52)-C(51)-C(57)	121.5(4)
O(3)-C(52)-C(51)	125.6(4)
O(3)-C(52)-C(47)	113.9(4)
C(51)-C(52)-C(47)	120.4(4)
C(55)-C(53)-C(56)	110.2(6)
C(55)-C(53)-C(54)	106.1(6)
C(56)-C(53)-C(54)	110.5(6)
C(55)-C(53)-C(49)	110.7(5)
C(56)-C(53)-C(49)	107.6(5)
C(54)-C(53)-C(49)	111.8(5)
C(53)-C(54)-H(54A)	109.5
C(53)-C(54)-H(54B)	109.5
H(54A)-C(54)-H(54B)	109.5
C(53)-C(54)-H(54C)	109.5
H(54A)-C(54)-H(54C)	109.5
H(54B)-C(54)-H(54C)	109.5
C(53)-C(55)-H(55A)	109.5
C(53)-C(55)-H(55B)	109.5
H(55A)-C(55)-H(55B)	109.5
C(53)-C(55)-H(55C)	109.5
H(55A)-C(55)-H(55C)	109.5
H(55B)-C(55)-H(55C)	109.5
C(53)-C(56)-H(56A)	109.5
C(53)-C(56)-H(56B)	109.5
H(56A)-C(56)-H(56B)	109.5
C(53)-C(56)-H(56C)	109.5
H(56A)-C(56)-H(56C)	109.5
H(56B)-C(56)-H(56C)	109.5
C(59)-C(57)-C(51)	109.2(4)
C(59)-C(57)-C(60)	109.7(5)
C(51)-C(57)-C(60)	111.0(4)
C(59)-C(57)-C(58)	109.5(5)
C(51)-C(57)-C(58)	110.0(4)
C(60)-C(57)-C(58)	107.4(4)
C(57)-C(58)-H(58A)	109.5
C(57)-C(58)-H(58B)	109.5
H(58A)-C(58)-H(58B)	109.5
C(57)-C(58)-H(58C)	109.5
H(58A)-C(58)-H(58C)	109.5
H(58B)-C(58)-H(58C)	109.5
C(57)-C(59)-H(59A)	109.5
C(57)-C(59)-H(59B)	109.5
H(59A)-C(59)-H(59B)	109.5
C(57)-C(59)-H(59C)	109.5
H(59A)-C(59)-H(59C)	109.5
H(59B)-C(59)-H(59C)	109.5
C(57)-C(60)-H(60A)	109.5
C(57)-C(60)-H(60B)	109.5
H(60A)-C(60)-H(60B)	109.5
C(57)-C(60)-H(60C)	109.5
H(60A)-C(60)-H(60C)	109.5
H(60B)-C(60)-H(60C)	109.5
C(62)-C(61)-C(66)	119.6(5)
C(62)-C(61)-N(4)	121.7(5)
C(66)-C(61)-N(4)	118.8(5)
C(63)-C(62)-C(61)	119.2(5)
C(63)-C(62)-H(62A)	120.4
C(61)-C(62)-H(62A)	120.4
C(64)-C(63)-C(62)	120.2(6)

C(64)-C(63)-H(63A) 119.9
C(62)-C(63)-H(63A) 119.9
C(65)-C(64)-C(63) 120.8(6)
C(65)-C(64)-H(64A) 119.6
C(63)-C(64)-H(64A) 119.6
C(64)-C(65)-C(66) 120.3(6)
C(64)-C(65)-H(65A) 119.9
C(66)-C(65)-H(65A) 119.9
C(65)-C(66)-C(61) 119.9(6)
C(65)-C(66)-H(66A) 120.1
C(61)-C(66)-H(66A) 120.1
C(72)-C(67)-N(4) 112.5(4)
C(72)-C(67)-C(68) 121.3(4)
N(4)-C(67)-C(68) 126.1(4)
C(69)-C(68)-C(67) 118.8(4)
C(69)-C(68)-H(68A) 120.6
C(67)-C(68)-H(68A) 120.6
C(68)-C(69)-C(70) 119.0(4)
C(68)-C(69)-C(77) 121.9(5)
C(70)-C(69)-C(77) 119.0(5)
C(69)-C(70)-C(71) 123.7(5)
C(69)-C(70)-H(70A) 118.1
C(71)-C(70)-H(70A) 118.1
C(72)-C(71)-C(70) 115.2(4)
C(72)-C(71)-C(73) 121.5(4)
C(70)-C(71)-C(73) 123.3(4)
O(4)-C(72)-C(67) 114.2(4)
O(4)-C(72)-C(71) 123.8(4)
C(67)-C(72)-C(71) 121.9(4)
C(74)-C(73)-C(76) 108.4(4)
C(74)-C(73)-C(71) 110.2(4)
C(76)-C(73)-C(71) 112.1(4)
C(74)-C(73)-C(75) 108.3(4)
C(76)-C(73)-C(75) 108.3(4)
C(71)-C(73)-C(75) 109.5(4)
C(73)-C(74)-H(74A) 109.5
C(73)-C(74)-H(74B) 109.5
H(74A)-C(74)-H(74B) 109.5
C(73)-C(74)-H(74C) 109.5
H(74A)-C(74)-H(74C) 109.5
H(74B)-C(74)-H(74C) 109.5
C(73)-C(75)-H(75A) 109.5
C(73)-C(75)-H(75B) 109.5
H(75A)-C(75)-H(75B) 109.5
C(73)-C(75)-H(75C) 109.5
H(75A)-C(75)-H(75C) 109.5
H(75B)-C(75)-H(75C) 109.5
C(73)-C(76)-H(76A) 109.5
C(73)-C(76)-H(76B) 109.5
H(76A)-C(76)-H(76B) 109.5
C(73)-C(76)-H(76C) 109.5
H(76A)-C(76)-H(76C) 109.5
H(76B)-C(76)-H(76C) 109.5
C(79)-C(77)-C(78) 109.4(6)
C(79)-C(77)-C(80) 107.3(5)
C(78)-C(77)-C(80) 108.3(5)
C(79)-C(77)-C(69) 110.5(4)
C(78)-C(77)-C(69) 109.8(5)
C(80)-C(77)-C(69) 111.4(5)
C(77)-C(78)-H(78A) 109.5
C(77)-C(78)-H(78B) 109.5
H(78A)-C(78)-H(78B) 109.5
C(77)-C(78)-H(78C) 109.5
H(78A)-C(78)-H(78C) 109.5
H(78B)-C(78)-H(78C) 109.5
C(77)-C(79)-H(79A) 109.5
C(77)-C(79)-H(79B) 109.5
H(79A)-C(79)-H(79B) 109.5
C(77)-C(79)-H(79C) 109.5
H(79A)-C(79)-H(79C) 109.5
H(79B)-C(79)-H(79C) 109.5
C(77)-C(80)-H(80A) 109.5
C(77)-C(80)-H(80B) 109.5
H(80A)-C(80)-H(80B) 109.5
C(77)-C(80)-H(80C) 109.5
H(80A)-C(80)-H(80C) 109.5
H(80B)-C(80)-H(80C) 109.5
C(82)-C(81)-C(86) 120.8(4)
C(82)-C(81)-N(5) 118.7(4)
C(86)-C(81)-N(5) 120.5(4)
C(83)-C(82)-C(81) 119.4(5)
C(83)-C(82)-H(82A) 120.3
C(81)-C(82)-H(82A) 120.3
C(84)-C(83)-C(82) 120.7(5)
C(84)-C(83)-H(83A) 119.6
C(82)-C(83)-H(83A) 119.6
C(83)-C(84)-C(85) 119.8(5)
C(83)-C(84)-H(84A) 120.1
C(85)-C(84)-H(84A) 120.1
C(84)-C(85)-C(86) 120.7(5)
C(84)-C(85)-H(85A) 119.7
C(86)-C(85)-H(85A) 119.7
C(81)-C(86)-C(85) 118.4(5)
C(81)-C(86)-H(86A) 120.8
C(85)-C(86)-H(86A) 120.8
N(5)-C(87)-C(88) 127.9(4)
N(5)-C(87)-C(92) 111.0(4)
C(88)-C(87)-C(92) 121.1(4)
C(89)-C(88)-C(87) 119.4(4)
C(89)-C(88)-H(88A) 120.3
C(87)-C(88)-H(88A) 120.3
C(88)-C(89)-C(90) 118.6(4)
C(88)-C(89)-C(93) 119.5(4)
C(90)-C(89)-C(93) 121.9(4)
C(91)-C(90)-C(89) 124.5(4)
C(91)-C(90)-H(90A) 117.8
C(89)-C(90)-H(90A) 117.8
C(90)-C(91)-C(92) 115.9(4)
C(90)-C(91)-C(97) 122.0(4)
C(92)-C(91)-C(97) 122.1(4)
O(8)-C(92)-C(91) 124.2(4)
O(8)-C(92)-C(87) 115.5(4)

C(91)-C(92)-C(87)	120.3(4)
C(96)-C(93)-C(89)	112.8(4)
C(96)-C(93)-C(94)	108.2(5)
C(89)-C(93)-C(94)	109.7(4)
C(96)-C(93)-C(95)	107.8(5)
C(89)-C(93)-C(95)	109.0(4)
C(94)-C(93)-C(95)	109.3(4)
C(93)-C(94)-H(94A)	109.5
C(93)-C(94)-H(94B)	109.5
H(94A)-C(94)-H(94B)	109.5
C(93)-C(94)-H(94C)	109.5
H(94A)-C(94)-H(94C)	109.5
H(94B)-C(94)-H(94C)	109.5
C(93)-C(95)-H(95A)	109.5
C(93)-C(95)-H(95B)	109.5
H(95A)-C(95)-H(95B)	109.5
C(93)-C(95)-H(95C)	109.5
H(95A)-C(95)-H(95C)	109.5
H(95B)-C(95)-H(95C)	109.5
C(93)-C(96)-H(96A)	109.5
C(93)-C(96)-H(96B)	109.5
H(96A)-C(96)-H(96B)	109.5
C(93)-C(96)-H(96C)	109.5
H(96A)-C(96)-H(96C)	109.5
H(96B)-C(96)-H(96C)	109.5
C(91)-C(97)-C(98)	112.0(4)
C(91)-C(97)-C(99)	110.1(4)
C(98)-C(97)-C(99)	108.4(4)
C(91)-C(97)-C(100)	108.8(4)
C(98)-C(97)-C(100)	107.9(4)
C(99)-C(97)-C(100)	109.6(4)
C(97)-C(98)-H(98A)	109.5
C(97)-C(98)-H(98B)	109.5
H(98A)-C(98)-H(98B)	109.5
C(97)-C(98)-H(98C)	109.5
H(98A)-C(98)-H(98C)	109.5
H(98B)-C(98)-H(98C)	109.5
C(97)-C(99)-H(99A)	109.5
C(97)-C(99)-H(99B)	109.5
H(99A)-C(99)-H(99B)	109.5
C(97)-C(99)-H(99C)	109.5
H(99A)-C(99)-H(99C)	109.5
H(99B)-C(99)-H(99C)	109.5
C(97)-C(100)-H(10A)	109.5
C(97)-C(100)-H(10B)	109.5
H(10A)-C(100)-H(10B)	109.5
C(97)-C(100)-H(10C)	109.5
H(10A)-C(100)-H(10C)	109.5
H(10B)-C(100)-H(10C)	109.5
C(106)-C(101)-C(102)	119.7(5)
C(106)-C(101)-N(6)	121.0(4)
C(102)-C(101)-N(6)	119.3(4)
C(103)-C(102)-C(101)	119.7(5)
C(103)-C(102)-H(10D)	120.2
C(101)-C(102)-H(10D)	120.2
C(104)-C(103)-C(102)	120.9(5)
C(104)-C(103)-H(10E)	119.5
C(102)-C(103)-H(10E)	119.5
C(103)-C(104)-C(105)	119.8(5)
C(103)-C(104)-H(10F)	120.1
C(105)-C(104)-H(10F)	120.1
C(104)-C(105)-C(106)	120.2(5)
C(104)-C(105)-H(10G)	119.9
C(106)-C(105)-H(10G)	119.9
C(101)-C(106)-C(105)	119.6(5)
C(101)-C(106)-H(10H)	120.2
C(105)-C(106)-H(10H)	120.2
N(6)-C(107)-C(108)	127.2(4)
N(6)-C(107)-C(112)	112.2(4)
C(108)-C(107)-C(112)	120.5(4)
C(109)-C(108)-C(107)	119.6(4)
C(109)-C(108)-H(10I)	120.2
C(107)-C(108)-H(10I)	120.2
C(108)-C(109)-C(110)	118.6(4)
C(108)-C(109)-C(118)	121.7(4)
C(110)-C(109)-C(118)	119.7(4)
C(111)-C(110)-C(109)	123.7(4)
C(111)-C(110)-H(11B)	118.2
C(109)-C(110)-H(11B)	118.2
C(110)-C(111)-C(112)	116.2(4)
C(110)-C(111)-C(113)	122.6(4)
C(112)-C(111)-C(113)	121.2(4)
O(9)-C(112)-C(111)	124.6(4)
O(9)-C(112)-C(107)	114.1(4)
C(111)-C(112)-C(107)	121.3(4)
C(114)-C(113)-C(115)	108.1(4)
C(114)-C(113)-C(111)	112.3(4)
C(115)-C(113)-C(111)	109.7(4)
C(114)-C(113)-C(116)	108.0(4)
C(115)-C(113)-C(116)	108.7(4)
C(111)-C(113)-C(116)	109.9(4)
C(113)-C(114)-H(11C)	109.5
C(113)-C(114)-H(11D)	109.5
H(11C)-C(114)-H(11D)	109.5
C(113)-C(114)-H(11E)	109.5
H(11C)-C(114)-H(11E)	109.5
H(11D)-C(114)-H(11E)	109.5
C(113)-C(115)-H(11F)	109.5
C(113)-C(115)-H(11G)	109.5
H(11F)-C(115)-H(11G)	109.5
C(113)-C(115)-H(11H)	109.5
H(11F)-C(115)-H(11H)	109.5
H(11G)-C(115)-H(11H)	109.5
C(113)-C(116)-H(11I)	109.5
C(113)-C(116)-H(11J)	109.5
H(11I)-C(116)-H(11J)	109.5
C(113)-C(116)-H(11K)	109.5
H(11I)-C(116)-H(11K)	109.5
H(11J)-C(116)-H(11K)	109.5
C(118)-C(117)-H(11L)	109.5
C(118)-C(117)-H(11M)	109.5
H(11L)-C(117)-H(11M)	109.5

C(118)-C(117)-H(11N)	109.5
H(11L)-C(117)-H(11N)	109.5
H(11M)-C(117)-H(11N)	109.5
C(120)-C(118)-C(119)	110.3(5)
C(120)-C(118)-C(109)	110.8(4)
C(119)-C(118)-C(109)	112.9(4)
C(120)-C(118)-C(117)	107.7(5)
C(119)-C(118)-C(117)	105.8(5)
C(109)-C(118)-C(117)	109.0(4)
C(118)-C(119)-H(11O)	109.5
C(118)-C(119)-H(11P)	109.5
H(11O)-C(119)-H(11P)	109.5
C(118)-C(119)-H(11Q)	109.5
H(11O)-C(119)-H(11Q)	109.5
H(11P)-C(119)-H(11Q)	109.5
C(118)-C(120)-H(12A)	109.5
C(118)-C(120)-H(12B)	109.5
H(12A)-C(120)-H(12B)	109.5
C(118)-C(120)-H(12C)	109.5
H(12A)-C(120)-H(12C)	109.5
H(12B)-C(120)-H(12C)	109.5
C(126)-C(121)-C(122)	121.1(5)
C(126)-C(121)-N(7)	120.8(4)
C(122)-C(121)-N(7)	118.1(4)
C(121)-C(122)-C(123)	118.3(6)
C(121)-C(122)-H(12D)	120.8
C(123)-C(122)-H(12D)	120.8
C(124)-C(123)-C(122)	120.8(6)
C(124)-C(123)-H(12E)	119.6
C(122)-C(123)-H(12E)	119.6
C(123)-C(124)-C(125)	120.3(6)
C(123)-C(124)-H(12F)	119.8
C(125)-C(124)-H(12F)	119.8
C(124)-C(125)-C(126)	120.3(6)
C(124)-C(125)-H(12G)	119.9
C(126)-C(125)-H(12G)	119.9
C(121)-C(126)-C(125)	119.0(6)
C(121)-C(126)-H(12H)	120.5
C(125)-C(126)-H(12H)	120.5
N(7)-C(127)-C(132)	126.7(4)
N(7)-C(127)-C(128)	111.8(4)
C(132)-C(127)-C(128)	121.5(4)
O(10)-C(128)-C(127)	114.5(4)
O(10)-C(128)-C(129)	124.9(4)
C(127)-C(128)-C(129)	120.7(4)
C(130)-C(129)-C(128)	114.6(4)
C(130)-C(129)-C(138)	122.8(4)
C(128)-C(129)-C(138)	122.6(4)
C(129)-C(130)-C(131)	125.9(4)
C(129)-C(130)-H(13A)	117.1
C(131)-C(130)-H(13A)	117.1
C(132)-C(131)-C(130)	117.9(4)
C(132)-C(131)-C(133)	122.5(4)
C(130)-C(131)-C(133)	119.6(4)
C(131)-C(132)-C(127)	119.3(4)
C(131)-C(132)-H(13B)	120.3
C(127)-C(132)-H(13B)	120.3
C(136)-C(133)-C(131)	108.9(4)
C(136)-C(133)-C(135)	109.0(5)
C(131)-C(133)-C(135)	111.4(4)
C(136)-C(133)-C(134)	108.3(5)
C(131)-C(133)-C(134)	110.7(5)
C(135)-C(133)-C(134)	108.6(5)
C(133)-C(134)-H(13C)	109.5
C(133)-C(134)-H(13D)	109.5
H(13C)-C(134)-H(13D)	109.5
C(133)-C(134)-H(13E)	109.5
H(13C)-C(134)-H(13E)	109.5
H(13D)-C(134)-H(13E)	109.5
C(133)-C(135)-H(13F)	109.5
C(133)-C(135)-H(13G)	109.5
H(13F)-C(135)-H(13G)	109.5
C(133)-C(135)-H(13H)	109.5
H(13F)-C(135)-H(13H)	109.5
H(13G)-C(135)-H(13H)	109.5
C(133)-C(136)-H(13I)	109.5
C(133)-C(136)-H(13J)	109.5
H(13I)-C(136)-H(13J)	109.5
C(133)-C(136)-H(13K)	109.5
H(13I)-C(136)-H(13K)	109.5
H(13J)-C(136)-H(13K)	109.5
C(138)-C(137)-H(13L)	109.5
C(138)-C(137)-H(13M)	109.5
H(13L)-C(137)-H(13M)	109.5
C(138)-C(137)-H(13N)	109.5
H(13L)-C(137)-H(13N)	109.5
H(13M)-C(137)-H(13N)	109.5
C(140)-C(138)-C(129)	108.9(4)
C(140)-C(138)-C(137)	108.0(5)
C(129)-C(138)-C(137)	112.5(4)
C(140)-C(138)-C(139)	109.3(5)
C(129)-C(138)-C(139)	109.3(4)
C(137)-C(138)-C(139)	108.7(5)
C(138)-C(139)-H(13O)	109.5
C(138)-C(139)-H(13P)	109.5
H(13O)-C(139)-H(13P)	109.5
C(138)-C(139)-H(13Q)	109.5
H(13O)-C(139)-H(13Q)	109.5
H(13P)-C(139)-H(13Q)	109.5
C(138)-C(140)-H(14D)	109.5
C(138)-C(140)-H(14E)	109.5
H(14D)-C(140)-H(14E)	109.5
C(138)-C(140)-H(14F)	109.5
H(14D)-C(140)-H(14F)	109.5
H(14E)-C(140)-H(14F)	109.5
C(142)-C(141)-C(146)	120.2(5)
C(142)-C(141)-N(8)	121.2(4)
C(146)-C(141)-N(8)	118.5(4)
C(141)-C(142)-C(143)	120.2(5)
C(141)-C(142)-H(14G)	119.9
C(143)-C(142)-H(14G)	119.9
C(144)-C(143)-C(142)	119.7(6)

C(144)-C(143)-H(14H)	120.1
C(142)-C(143)-H(14H)	120.1
C(143)-C(144)-C(145)	120.6(6)
C(143)-C(144)-H(14I)	119.7
C(145)-C(144)-H(14I)	119.7
C(144)-C(145)-C(146)	120.0(6)
C(144)-C(145)-H(14J)	120.0
C(146)-C(145)-H(14J)	120.0
C(141)-C(146)-C(145)	119.2(5)
C(141)-C(146)-H(14K)	120.4
C(145)-C(146)-H(14K)	120.4
N(8)-C(147)-C(152)	112.7(4)
N(8)-C(147)-C(148)	126.3(4)
C(152)-C(147)-C(148)	121.0(4)
C(149)-C(148)-C(147)	118.7(5)
C(149)-C(148)-H(14L)	120.7
C(147)-C(148)-H(14L)	120.7
C(150)-C(149)-C(148)	118.7(4)
C(150)-C(149)-C(153)	122.5(5)
C(148)-C(149)-C(153)	118.8(5)
C(149)-C(150)-C(151)	124.2(5)
C(149)-C(150)-H(15D)	117.9
C(151)-C(150)-H(15D)	117.9
C(150)-C(151)-C(152)	115.6(4)
C(150)-C(151)-C(157)	123.1(4)
C(152)-C(151)-C(157)	121.3(4)
O(11)-C(152)-C(147)	113.9(4)
O(11)-C(152)-C(151)	124.5(4)
C(147)-C(152)-C(151)	121.6(4)
C(161)-C(153)-C(155)	123.9(8)
C(161)-C(153)-C(149)	113.6(8)
C(155)-C(153)-C(149)	110.8(5)
C(161)-C(153)-C(154)	90.2(8)
C(155)-C(153)-C(154)	107.2(6)
C(149)-C(153)-C(154)	107.6(5)
C(161)-C(153)-C(156)	27.9(7)
C(155)-C(153)-C(156)	103.3(7)
C(149)-C(153)-C(156)	111.8(6)
C(154)-C(153)-C(156)	116.0(7)
C(153)-C(154)-H(15E)	109.5
C(153)-C(154)-H(15F)	109.5
H(15E)-C(154)-H(15F)	109.5
C(153)-C(154)-H(15G)	109.5
H(15E)-C(154)-H(15G)	109.5
H(15F)-C(154)-H(15G)	109.5
C(153)-C(155)-H(15H)	109.5
C(153)-C(155)-H(15I)	109.5
H(15H)-C(155)-H(15I)	109.5
C(153)-C(155)-H(15J)	109.5
H(15H)-C(155)-H(15J)	109.5
H(15I)-C(155)-H(15J)	109.5
C(153)-C(156)-H(15K)	109.5
C(153)-C(156)-H(15L)	109.5
H(15K)-C(156)-H(15L)	109.5
C(153)-C(156)-H(15M)	109.5
H(15K)-C(156)-H(15M)	109.5
H(15L)-C(156)-H(15M)	109.5
C(153)-C(161)-H(16D)	109.5
C(153)-C(161)-H(16E)	109.5
H(16D)-C(161)-H(16E)	109.5
C(153)-C(161)-H(16F)	109.5
H(16D)-C(161)-H(16F)	109.5
H(16E)-C(161)-H(16F)	109.5
C(151)-C(157)-C(159)	112.4(4)
C(151)-C(157)-C(160)	110.3(4)
C(159)-C(157)-C(160)	108.3(4)
C(151)-C(157)-C(158)	109.7(4)
C(159)-C(157)-C(158)	106.9(4)
C(160)-C(157)-C(158)	109.0(4)
C(157)-C(158)-H(15N)	109.5
C(157)-C(158)-H(15O)	109.5
H(15N)-C(158)-H(15O)	109.5
C(157)-C(158)-H(15P)	109.5
H(15N)-C(158)-H(15P)	109.5
H(15O)-C(158)-H(15P)	109.5
C(157)-C(159)-H(15Q)	109.5
C(157)-C(159)-H(15R)	109.5
H(15Q)-C(159)-H(15R)	109.5
C(157)-C(159)-H(15S)	109.5
H(15Q)-C(159)-H(15S)	109.5
H(15R)-C(159)-H(15S)	109.5
C(157)-C(160)-H(16G)	109.5
C(157)-C(160)-H(16H)	109.5
H(16G)-C(160)-H(16H)	109.5
C(157)-C(160)-H(16I)	109.5
H(16G)-C(160)-H(16I)	109.5
H(16H)-C(160)-H(16I)	109.5
N(1S)-C(2S)-C(1S)	176.1(11)
C(8)-N(1)-C(1)	117.7(4)
C(8)-N(1)-Re(1)	119.1(3)
C(1)-N(1)-Re(1)	122.9(3)
C(27)-N(2)-C(21)	118.8(3)
C(27)-N(2)-Re(1)	116.9(3)
C(21)-N(2)-Re(1)	123.4(3)
C(47)-N(3)-C(41)	116.7(4)
C(47)-N(3)-Re(2)	119.6(3)
C(41)-N(3)-Re(2)	123.7(3)
C(67)-N(4)-C(61)	120.2(4)
C(67)-N(4)-Re(2)	116.8(3)
C(61)-N(4)-Re(2)	122.6(3)
C(87)-N(5)-C(81)	115.8(4)
C(87)-N(5)-Re(3)	119.6(3)
C(81)-N(5)-Re(3)	124.6(3)
C(107)-N(6)-C(101)	118.9(4)
C(107)-N(6)-Re(3)	116.9(3)
C(101)-N(6)-Re(3)	122.8(3)
C(127)-N(7)-C(121)	116.6(4)
C(127)-N(7)-Re(4)	119.7(3)
C(121)-N(7)-Re(4)	123.6(3)
C(147)-N(8)-C(141)	118.4(4)
C(147)-N(8)-Re(4)	117.2(3)
C(141)-N(8)-Re(4)	124.0(3)

C(7)-O(1)-Re(1)	119.1(3)
C(32)-O(2)-Re(1)	119.8(3)
C(52)-O(3)-Re(2)	120.4(3)
C(72)-O(4)-Re(2)	119.2(3)
Re(2)-O(5)-Re(1)	152.30(18)
C(92)-O(8)-Re(3)	119.2(3)
C(112)-O(9)-Re(3)	119.2(3)
C(128)-O(10)-Re(4)	119.7(3)
C(152)-O(11)-Re(4)	118.5(3)
Re(3)-O(12)-Re(4)	156.52(18)
O(6)-Re(1)-O(5)	97.76(14)
O(6)-Re(1)-O(2)	108.02(14)
O(5)-Re(1)-O(2)	83.62(13)
O(6)-Re(1)-N(2)	98.38(15)
O(5)-Re(1)-N(2)	157.94(14)
O(2)-Re(1)-N(2)	77.15(13)
O(6)-Re(1)-N(1)	88.11(15)
O(5)-Re(1)-N(1)	101.14(14)
O(2)-Re(1)-N(1)	162.55(14)
N(2)-Re(1)-N(1)	94.32(14)
O(6)-Re(1)-O(1)	162.63(14)
O(5)-Re(1)-O(1)	84.62(13)
O(2)-Re(1)-O(1)	89.33(12)
N(2)-Re(1)-O(1)	84.46(14)
N(1)-Re(1)-O(1)	74.57(13)
O(7)-Re(2)-O(5)	98.68(15)
O(7)-Re(2)-O(4)	105.23(14)
O(5)-Re(2)-O(4)	83.92(13)
O(7)-Re(2)-N(4)	97.98(16)
O(5)-Re(2)-N(4)	157.54(15)
O(4)-Re(2)-N(4)	77.24(14)
O(7)-Re(2)-N(3)	85.79(15)
O(5)-Re(2)-N(3)	97.33(14)
O(4)-Re(2)-N(3)	168.63(14)
N(4)-Re(2)-N(3)	98.81(15)
O(7)-Re(2)-O(3)	160.02(14)
O(5)-Re(2)-O(3)	85.60(13)
O(4)-Re(2)-O(3)	94.60(13)
N(4)-Re(2)-O(3)	83.87(14)
N(3)-Re(2)-O(3)	74.28(13)
O(13)-Re(3)-O(12)	99.71(14)
O(13)-Re(3)-O(9)	103.79(14)
O(12)-Re(3)-O(9)	83.49(12)
O(13)-Re(3)-N(6)	98.60(15)
O(12)-Re(3)-N(6)	156.23(14)
O(9)-Re(3)-N(6)	77.51(13)
O(13)-Re(3)-N(5)	85.96(14)
O(12)-Re(3)-N(5)	99.56(14)
O(9)-Re(3)-N(5)	169.23(13)
N(6)-Re(3)-N(5)	96.73(14)
O(13)-Re(3)-O(8)	160.46(13)
O(12)-Re(3)-O(8)	84.86(13)
O(9)-Re(3)-O(8)	95.58(12)
N(6)-Re(3)-O(8)	82.93(14)
N(5)-Re(3)-O(8)	74.53(13)
O(14)-Re(4)-O(12)	99.02(14)
O(14)-Re(4)-O(11)	103.19(14)
O(12)-Re(4)-O(11)	83.66(13)
O(14)-Re(4)-N(8)	98.46(16)
O(12)-Re(4)-N(8)	156.72(15)
O(11)-Re(4)-N(8)	77.46(14)
O(14)-Re(4)-N(7)	86.23(15)
O(12)-Re(4)-N(7)	99.27(14)
O(11)-Re(4)-N(7)	169.65(13)
N(8)-Re(4)-N(7)	97.11(15)
O(14)-Re(4)-O(10)	160.24(13)
O(12)-Re(4)-O(10)	85.91(13)
O(11)-Re(4)-O(10)	96.35(12)
N(8)-Re(4)-O(10)	82.79(14)
N(7)-Re(4)-O(10)	74.06(13)

Symmetry transformations used to generate equivalent atoms:

Anisotropic displacement parameters (Å2x 103) for CAL_9_227C. The anisotropic displacement factor exponent takes the form: -2p2[h2 a*2U11 + ... + 2 h k a* b* U12]

U11	U22	U33	U23	U13	U12	
C(1)	17(2)	22(2)	39(3)	-8(2)	-5(2)	-2(2)
C(2)	26(3)	35(3)	40(3)	-16(2)	-6(2)	1(2)
C(3)	31(3)	68(4)	60(4)	-32(4)	-13(3)	-5(3)
C(4)	24(3)	50(4)	107(6)	-42(4)	-6(3)	-9(3)
C(5)	29(3)	30(3)	88(5)	-8(3)	0(3)	-11(2)
C(6)	19(2)	25(3)	50(3)	-1(2)	-5(2)	-2(2)
C(7)	18(2)	20(2)	20(2)	4(2)	-4(2)	-4(2)
C(8)	20(2)	23(2)	20(2)	0(2)	2(2)	-4(2)
C(9)	19(2)	25(2)	31(3)	1(2)	0(2)	-6(2)
C(10)	25(3)	25(3)	27(2)	1(2)	7(2)	-2(2)
C(11)	36(3)	25(3)	23(2)	3(2)	1(2)	-6(2)
C(12)	26(2)	18(2)	27(2)	4(2)	-3(2)	-8(2)
C(13)	27(3)	33(3)	39(3)	1(2)	11(2)	-2(2)
C(14)	43(4)	67(5)	77(5)	-16(4)	29(3)	-26(3)
C(15)	32(3)	55(4)	57(4)	9(3)	13(3)	10(3)
C(16)	44(4)	60(4)	43(3)	11(3)	14(3)	-4(3)
C(17)	41(3)	33(3)	23(2)	-4(2)	-3(2)	-14(2)
C(18)	69(5)	57(4)	41(3)	-23(3)	-1(3)	-20(4)
C(19)	41(3)	58(4)	45(3)	3(3)	-17(3)	-16(3)
C(20)	61(4)	38(3)	35(3)	-2(2)	-6(3)	-28(3)
C(21)	17(2)	24(2)	19(2)	5(2)	-2(2)	-1(2)
C(22)	33(3)	30(3)	25(2)	0(2)	-3(2)	-12(2)
C(23)	38(3)	51(4)	24(3)	-2(2)	5(2)	-13(3)
C(24)	43(3)	48(4)	33(3)	8(3)	-1(3)	-23(3)
C(25)	35(3)	31(3)	39(3)	9(2)	-4(2)	-14(2)
C(26)	21(2)	21(2)	36(3)	0(2)	-3(2)	-4(2)
C(27)	18(2)	22(2)	23(2)	-3(2)	-3(2)	-4(2)
C(28)	26(3)	23(2)	29(2)	6(2)	-5(2)	-8(2)
C(29)	27(3)	29(3)	37(3)	6(2)	-14(2)	-6(2)
C(30)	24(3)	36(3)	51(3)	12(3)	-12(2)	-12(2)
C(31)	21(2)	31(3)	35(3)	9(2)	-1(2)	-6(2)
C(32)	23(2)	23(2)	26(2)	4(2)	-5(2)	-4(2)
C(33)	33(3)	45(3)	40(3)	17(3)	-16(2)	-13(3)
C(34)	75(5)	103(6)	39(4)	3(4)	-25(4)	8(5)
C(35)	80(5)	46(4)	48(4)	14(3)	-18(4)	11(4)
C(37)	28(3)	56(4)	54(4)	31(3)	-13(3)	-19(3)
C(38)	51(4)	52(4)	88(5)	32(4)	-21(4)	-30(3)
C(39)	48(4)	99(6)	53(4)	26(4)	8(3)	-19(4)
C(40)	36(4)	102(6)	106(6)	60(5)	-25(4)	-39(4)
C(41)	15(2)	42(3)	44(3)	-24(3)	4(2)	-9(2)
C(42)	34(3)	35(3)	95(5)	-22(3)	14(3)	-13(3)
C(43)	34(4)	48(4)	183(10)	-59(6)	34(5)	-15(3)
C(44)	30(4)	101(7)	174(11)	-100(8)	41(5)	-31(5)
C(45)	31(4)	166(9)	74(5)	-87(6)	20(3)	-36(5)
C(46)	25(3)	91(5)	45(4)	-29(3)	3(3)	-14(3)
C(47)	24(2)	25(2)	26(2)	-1(2)	-5(2)	-9(2)
C(48)	24(3)	32(3)	24(2)	-1(2)	0(2)	-8(2)

C(49)	26(3)	35(3)	23(2)	3(2)	2(2)	-9(2)
C(50)	40(3)	32(3)	26(3)	3(2)	-5(2)	-21(2)
C(51)	39(3)	24(3)	24(2)	0(2)	1(2)	-18(2)
C(52)	30(3)	23(2)	22(2)	1(2)	-2(2)	-9(2)
C(53)	25(3)	51(3)	32(3)	-4(2)	0(2)	-15(2)
C(54)	41(4)	80(6)	127(7)	-30(5)	40(4)	-20(4)
C(55)	32(4)	164(9)	78(5)	-55(6)	0(3)	-33(5)
C(56)	50(5)	123(7)	75(5)	48(5)	18(4)	-17(5)
C(57)	44(3)	23(3)	33(3)	-7(2)	3(2)	-13(2)
C(58)	52(4)	39(3)	32(3)	-6(2)	3(3)	-16(3)
C(59)	69(4)	25(3)	48(4)	-3(3)	3(3)	-2(3)
C(60)	71(5)	47(4)	46(4)	-19(3)	13(3)	-34(3)
C(61)	31(3)	38(3)	20(2)	3(2)	0(2)	-7(2)
C(62)	52(4)	46(3)	35(3)	5(3)	-4(3)	-24(3)
C(63)	59(4)	86(5)	47(4)	21(4)	-2(3)	-46(4)
C(64)	61(5)	73(5)	33(3)	10(3)	1(3)	-14(4)
C(65)	68(5)	65(4)	24(3)	-3(3)	-4(3)	-11(4)
C(66)	55(4)	48(3)	26(3)	2(2)	-11(3)	-19(3)
C(67)	26(3)	23(2)	25(2)	1(2)	-5(2)	-6(2)
C(68)	34(3)	28(3)	22(2)	1(2)	-3(2)	-2(2)
C(69)	32(3)	25(3)	29(3)	1(2)	-13(2)	-2(2)
C(70)	25(3)	26(3)	33(3)	-2(2)	-9(2)	4(2)
C(71)	22(2)	20(2)	26(2)	-3(2)	-3(2)	-2(2)
C(72)	22(2)	20(2)	24(2)	3(2)	-5(2)	-4(2)
C(73)	23(2)	23(2)	29(2)	2(2)	-1(2)	-1(2)
C(74)	35(3)	32(3)	40(3)	3(2)	-3(2)	-12(2)
C(75)	35(3)	34(3)	28(3)	-1(2)	-4(2)	-8(2)
C(76)	29(3)	43(3)	40(3)	2(3)	7(2)	-2(2)
C(77)	53(4)	29(3)	30(3)	0(2)	-13(3)	8(3)
C(78)	81(6)	61(5)	108(7)	7(5)	-58(5)	3(4)
C(79)	102(6)	35(4)	55(4)	-8(3)	-18(4)	17(4)
C(80)	94(6)	47(4)	54(4)	17(3)	-7(4)	22(4)
C(81)	12(2)	25(2)	28(2)	-9(2)	0(2)	-2(2)
C(82)	23(2)	28(3)	34(3)	-5(2)	-2(2)	-7(2)
C(83)	33(3)	26(3)	58(4)	-18(3)	-1(3)	-8(2)
C(84)	27(3)	48(4)	55(4)	-36(3)	7(3)	-8(3)
C(85)	22(3)	69(4)	29(3)	-19(3)	7(2)	-14(3)
C(86)	15(2)	39(3)	31(3)	-4(2)	2(2)	-5(2)
C(87)	20(2)	19(2)	21(2)	-2(2)	-2(2)	-4(2)
C(88)	22(2)	27(2)	23(2)	-9(2)	5(2)	-6(2)
C(89)	24(2)	27(3)	23(2)	2(2)	-2(2)	-10(2)
C(90)	27(3)	26(3)	27(2)	-3(2)	-2(2)	-13(2)
C(91)	23(2)	19(2)	21(2)	-6(2)	2(2)	-7(2)
C(92)	21(2)	19(2)	22(2)	1(2)	2(2)	-4(2)
C(93)	24(3)	34(3)	32(3)	-5(2)	7(2)	-12(2)
C(94)	33(3)	49(4)	45(3)	-2(3)	4(3)	-12(3)
C(95)	38(3)	65(4)	39(3)	4(3)	8(3)	-18(3)
C(96)	38(3)	56(4)	71(4)	-28(3)	20(3)	-31(3)
C(97)	34(3)	28(3)	26(2)	-7(2)	1(2)	-13(2)
C(98)	41(3)	40(3)	44(3)	-17(3)	3(3)	-17(3)
C(99)	37(3)	34(3)	44(3)	-17(3)	2(3)	1(2)
C(100)	55(4)	45(3)	29(3)	-11(2)	6(3)	-22(3)
C(101)	20(2)	25(2)	24(2)	4(2)	-2(2)	1(2)
C(102)	26(3)	32(3)	21(2)	1(2)	1(2)	-4(2)
C(103)	50(4)	35(3)	21(2)	0(2)	3(2)	-5(3)
C(104)	43(3)	51(4)	34(3)	5(3)	11(3)	-16(3)

C(105)	41(3)	47(3)	45(3)	9(3)	-9(3)	-25(3)
C(106)	34(3)	38(3)	26(3)	4(2)	-2(2)	-16(2)
C(107)	20(2)	21(2)	23(2)	0(2)	-4(2)	-3(2)
C(108)	29(3)	27(2)	18(2)	2(2)	-1(2)	-8(2)
C(109)	22(2)	20(2)	23(2)	2(2)	-3(2)	-1(2)
C(110)	20(2)	26(2)	23(2)	-1(2)	-3(2)	2(2)
C(111)	20(2)	22(2)	19(2)	-1(2)	-1(2)	-6(2)
C(112)	17(2)	20(2)	23(2)	-1(2)	-4(2)	-1(2)
C(113)	21(2)	20(2)	27(2)	3(2)	2(2)	-2(2)
C(114)	29(3)	38(3)	35(3)	8(2)	10(2)	-1(2)
C(115)	29(3)	35(3)	39(3)	2(2)	1(2)	-12(2)
C(116)	33(3)	32(3)	24(2)	2(2)	-4(2)	-2(2)
C(117)	54(4)	32(3)	64(4)	1(3)	-23(3)	6(3)
C(118)	34(3)	22(2)	26(2)	1(2)	-3(2)	2(2)
C(119)	74(5)	38(4)	54(4)	22(3)	13(4)	6(3)
C(120)	86(5)	31(3)	53(4)	-5(3)	-20(4)	4(3)
C(121)	15(2)	25(2)	36(3)	-11(2)	-2(2)	0(2)
C(122)	29(3)	44(3)	38(3)	-18(2)	-8(2)	0(2)
C(123)	29(3)	76(5)	49(4)	-38(3)	-13(3)	10(3)
C(124)	23(3)	55(4)	93(5)	-51(4)	-4(3)	2(3)
C(125)	25(3)	38(3)	93(5)	-21(3)	0(3)	-8(3)
C(126)	21(3)	31(3)	61(4)	-4(3)	-3(3)	-7(2)
C(127)	21(2)	22(2)	20(2)	3(2)	-2(2)	-4(2)
C(128)	19(2)	24(2)	23(2)	3(2)	-2(2)	-5(2)
C(129)	23(2)	23(2)	20(2)	-3(2)	2(2)	-3(2)
C(130)	31(3)	35(3)	15(2)	1(2)	4(2)	-6(2)
C(131)	24(2)	31(3)	17(2)	6(2)	1(2)	-5(2)
C(132)	22(2)	30(3)	25(2)	4(2)	-3(2)	-6(2)
C(133)	25(3)	50(3)	30(3)	3(2)	5(2)	-6(2)
C(134)	28(3)	66(5)	89(5)	-5(4)	16(3)	5(3)
C(135)	29(3)	139(7)	52(4)	-21(4)	13(3)	-37(4)
C(136)	48(4)	69(5)	46(4)	18(3)	8(3)	-17(3)
C(137)	53(4)	75(5)	63(4)	-43(4)	18(3)	-29(4)
C(138)	25(3)	39(3)	33(3)	-10(2)	0(2)	-13(2)
C(139)	61(4)	44(3)	41(3)	-8(3)	-1(3)	-29(3)
C(140)	56(4)	75(5)	52(4)	-3(3)	-27(3)	-26(4)
C(141)	19(2)	26(3)	29(2)	6(2)	-3(2)	1(2)
C(142)	46(4)	39(3)	32(3)	0(2)	7(3)	-15(3)
C(143)	63(4)	53(4)	37(3)	1(3)	14(3)	-15(3)
C(144)	47(4)	62(4)	45(4)	13(3)	4(3)	-26(3)
C(145)	51(4)	42(3)	53(4)	15(3)	-10(3)	-26(3)
C(146)	28(3)	30(3)	34(3)	6(2)	-3(2)	-8(2)
C(147)	24(2)	21(2)	24(2)	0(2)	-1(2)	-1(2)
C(148)	35(3)	26(3)	28(3)	9(2)	-11(2)	-5(2)
C(149)	31(3)	33(3)	38(3)	11(2)	-10(2)	-2(2)
C(150)	16(2)	39(3)	42(3)	4(2)	-7(2)	-3(2)
C(151)	22(2)	29(3)	25(2)	-2(2)	-1(2)	-5(2)
C(152)	20(2)	19(2)	25(2)	4(2)	-4(2)	-1(2)
C(153)	45(4)	52(4)	52(4)	23(3)	-20(3)	-14(3)
C(154)	161(10)	97(7)	73(6)	10(5)	-66(6)	12(7)
C(155)	105(7)	55(5)	108(7)	38(5)	-62(6)	-7(5)
C(157)	15(2)	38(3)	34(3)	2(2)	-3(2)	-2(2)
C(158)	36(3)	63(4)	32(3)	-5(3)	7(2)	-13(3)
C(159)	23(3)	70(4)	50(4)	19(3)	-5(3)	-15(3)
C(160)	43(3)	37(3)	43(3)	7(3)	0(3)	-13(3)
N(1)	16(2)	21(2)	25(2)	3(2)	-1(2)	-4(2)

N(2)	19(2)	23(2)	23(2)	9(2)	-5(2)	-6(2)
N(3)	21(2)	26(2)	21(2)	-4(2)	-2(2)	-6(2)
N(4)	30(2)	30(2)	20(2)	7(2)	-1(2)	-3(2)
N(5)	17(2)	18(2)	24(2)	-1(2)	1(2)	-4(2)
N(6)	21(2)	24(2)	20(2)	5(2)	4(2)	-3(2)
N(7)	22(2)	19(2)	22(2)	-3(2)	-1(2)	-2(2)
N(8)	21(2)	27(2)	23(2)	8(2)	0(2)	-4(2)
O(1)	20(2)	27(2)	23(2)	-2(1)	0(1)	-8(1)
O(2)	17(2)	26(2)	24(2)	6(1)	0(1)	-5(1)
O(3)	24(2)	20(2)	26(2)	-3(1)	3(1)	-7(1)
O(4)	20(2)	26(2)	24(2)	6(1)	-1(1)	-3(1)
O(5)	23(2)	25(2)	25(2)	3(1)	-4(1)	-3(1)
O(6)	28(2)	30(2)	29(2)	-4(2)	-5(2)	-4(2)
O(7)	25(2)	37(2)	33(2)	-5(2)	-4(2)	-9(2)
O(8)	16(2)	22(2)	30(2)	-9(1)	4(1)	-4(1)
O(9)	17(2)	20(2)	22(2)	3(1)	1(1)	-3(1)
O(10)	17(2)	26(2)	23(2)	-3(1)	-1(1)	-7(1)
O(11)	16(2)	28(2)	24(2)	5(1)	1(1)	-3(1)
O(12)	21(2)	22(2)	25(2)	6(1)	-1(1)	-4(1)
O(13)	24(2)	28(2)	28(2)	-6(1)	1(1)	-9(1)
O(14)	23(2)	31(2)	28(2)	-1(1)	1(1)	-4(2)
Re(1)	16(1)	22(1)	20(1)	1(1)	-1(1)	-3(1)
Re(2)	19(1)	22(1)	20(1)	2(1)	-1(1)	-4(1)
Re(3)	15(1)	19(1)	21(1)	0(1)	1(1)	-3(1)
Re(4)	15(1)	20(1)	20(1)	0(1)	0(1)	-3(1)

Hydrogen coordinates (x 104) and isotropic displacement parameters (Å2x 10 3) for CAL_9_227C.

x	y	z	U(eq)	
H(2A)	3624	-763	2078	41
H(3A)	2608	-1353	1778	62
H(4A)	1991	-2107	2305	70
H(5A)	2390	-2308	3142	58
H(6A)	3365	-1706	3464	38
H(9A)	2401	-648	3574	30
H(11A)	3051	576	4476	35
H(14A)	1527	-950	4381	93
H(14B)	966	-500	3949	93
H(14C)	480	-506	4528	93
H(15A)	814	669	3939	79
H(15B)	1271	956	4371	79
H(15C)	320	652	4516	79
H(16A)	2203	-496	5053	79
H(16B)	1155	-45	5186	79
H(16C)	2115	250	5045	79
H(18A)	4134	949	4785	81
H(18B)	3737	1463	4346	81
H(18C)	4778	1450	4547	81
H(19A)	6092	33	4005	69
H(19B)	5588	92	4580	69
H(19C)	6210	599	4325	69
H(20A)	5447	856	3319	63
H(20B)	5570	1391	3670	63
H(20C)	4528	1408	3468	63
H(22A)	5252	-1096	4221	34
H(23A)	4240	-1607	4793	45
H(24A)	3923	-2560	4617	49
H(25A)	4584	-3009	3852	42
H(26A)	5537	-2483	3248	31
H(28A)	7221	-2025	4070	31
H(30A)	9655	-1367	3518	44
H(34A)	7881	-2173	4806	113
H(34B)	8389	-1591	4856	113
H(34C)	8892	-2285	5062	113
H(35A)	8562	-2940	4119	92
H(35B)	9447	-3120	4475	92
H(35C)	9669	-2951	3877	92
H(36A)	9761	-1439	4453	82
H(36B)	10477	-1945	4104	82
H(36C)	10244	-2116	4701	82
H(36D)	10538	-2440	3987	55
H(36E)	10438	-2230	4559	55
H(36F)	10265	-1708	4094	55
H(38A)	7785	63	2610	93
H(38B)	8775	311	2423	93
H(38C)	8448	212	3021	93
H(39A)	9476	-1325	2170	103
H(39B)	9424	-630	1903	103
H(39C)	8432	-882	2074	103

H(40A)	10478	-1054	2807	121
H(40B)	10054	-464	3147	121
H(40C)	10384	-351	2551	121
H(42A)	7040	-1147	1523	65
H(43A)	7664	-1991	1021	105
H(44A)	7930	-1833	147	117
H(45A)	7518	-829	-239	102
H(46A)	6954	44	254	63
H(48A)	8529	-20	874	32
H(50A)	8768	1542	1527	37
H(54A)	10983	45	572	126
H(54B)	9916	-67	492	126
H(54C)	10342	-251	1035	126
H(55A)	11183	832	1095	130
H(55B)	10536	556	1565	130
H(55C)	10264	1287	1379	130
H(56A)	10455	1173	272	133
H(56B)	9522	1609	562	133
H(56C)	9369	1082	211	133
H(58A)	6054	1415	2531	61
H(58B)	6300	1989	2797	61
H(58C)	7097	1343	2749	61
H(59A)	6461	2507	1469	74
H(59B)	5917	2699	2016	74
H(59C)	5666	2128	1750	74
H(60A)	8087	2401	1834	78
H(60B)	8313	1939	2332	78
H(60C)	7508	2581	2377	78
H(62A)	5894	1714	881	51
H(63A)	6780	1897	93	74
H(64A)	6547	1421	-616	69
H(65A)	5419	779	-565	63
H(66A)	4604	532	219	50
H(68A)	3541	1994	674	34
H(70A)	1392	2198	1845	35
H(74A)	1477	501	2253	53
H(74B)	1669	380	2842	53
H(74C)	2573	252	2408	53
H(75A)	3421	968	2803	49
H(75B)	2522	1061	3242	49
H(75C)	2840	1663	2930	49
H(76A)	587	1608	2436	59
H(76B)	1126	2049	2707	59
H(76C)	808	1448	3020	59
H(78A)	459	2615	1145	124
H(78B)	946	2417	589	124
H(78C)	500	3142	685	124
H(79A)	1312	3227	1647	101
H(79B)	1339	3730	1168	101
H(79C)	2336	3399	1408	101
H(80A)	3076	3079	562	108
H(80B)	2073	3417	330	108
H(80C)	2538	2693	235	108
H(82A)	3348	6202	3708	33
H(83A)	2629	6986	4261	46
H(84A)	2198	6726	5115	51

H(85A)	2457	5676	5422	47
H(86A)	3092	4870	4862	34
H(88A)	1711	4958	4269	29
H(90A)	1439	3579	3404	31
H(94A)	-333	4984	3678	64
H(94B)	7	5250	4159	64
H(94C)	-927	4923	4230	64
H(95A)	902	3756	4808	71
H(95B)	-182	4174	4914	71
H(95C)	753	4500	4852	71
H(96A)	-135	3830	3628	79
H(96B)	-730	3797	4182	79
H(96C)	334	3354	4075	79
H(98A)	2161	2778	2983	60
H(98B)	2635	2761	2402	60
H(98C)	1790	3345	2577	60
H(99A)	3838	2627	3272	59
H(99B)	4553	3088	3038	59
H(99C)	4284	2600	2686	59
H(10A)	2879	4084	2222	62
H(10B)	3696	3488	2043	62
H(10C)	3969	3979	2391	62
H(10D)	5149	4320	4908	32
H(10E)	4157	3932	5573	44
H(10F)	3249	3204	5433	52
H(10G)	3421	2792	4632	50
H(10H)	4452	3153	3961	38
H(10I)	6536	2897	4305	29
H(11B)	8796	2851	3200	29
H(11C)	9665	3491	2731	55
H(11D)	9522	3763	2156	55
H(11E)	9151	3134	2370	55
H(11F)	8798	4546	3027	51
H(11G)	7736	4860	2849	51
H(11H)	8688	4785	2442	51
H(11I)	7473	3609	2121	46
H(11J)	7892	4216	1893	46
H(11K)	6936	4293	2297	46
H(11L)	9188	2508	4413	77
H(11M)	9680	2335	3852	77
H(11N)	9629	1788	4296	77
H(11O)	7072	1853	4417	92
H(11P)	7641	2213	4748	92
H(11Q)	8097	1499	4618	92
H(12A)	7819	1593	3513	87
H(12B)	8804	1244	3758	87
H(12C)	8845	1786	3310	87
H(12D)	6575	5860	3171	44
H(12E)	7546	6396	3568	62
H(12F)	8042	7273	3162	67
H(12G)	7622	7624	2348	62
H(12H)	6736	7065	1918	45
H(13A)	7557	4830	725	33
H(13B)	7920	5945	1793	31
H(13C)	9083	4537	629	97
H(13D)	9535	4441	1167	97

H(13E)	10122	4715	672	97
H(13F)	9235	6065	1390	106
H(13G)	10214	5638	1130	106
H(13H)	9629	5367	1627	106
H(13I)	8490	5617	249	84
H(13J)	9539	5776	301	84
H(13K)	8555	6217	542	84
H(13L)	6638	4447	311	91
H(13M)	5955	3939	451	91
H(13N)	6913	3892	743	91
H(13O)	4940	4442	1683	69
H(13P)	5887	3892	1578	69
H(13Q)	4936	3932	1282	69
H(14D)	5166	5301	468	86
H(14E)	4486	5295	999	86
H(14F)	4514	4776	602	86
H(14G)	4826	6299	970	48
H(14H)	5732	6828	344	63
H(14I)	6118	7763	512	61
H(14J)	5545	8211	1285	57
H(14K)	4679	7670	1925	37
H(14L)	2904	7310	1195	36
H(15D)	418	6815	1878	40
H(15E)	2136	7199	495	169
H(15F)	1125	7511	260	169
H(15G)	1268	6816	534	169
H(15H)	891	8371	1340	134
H(15I)	834	8416	736	134
H(15J)	1870	8168	963	134
H(15K)	-325	7777	1519	57
H(15L)	-323	7190	1193	57
H(15M)	-472	7887	923	57
H(16D)	-403	7490	1301	113
H(16E)	151	6898	987	113
H(16F)	-202	7569	692	113
H(15N)	1093	6674	3216	66
H(15O)	2023	6099	3216	66
H(15P)	967	5996	3469	66
H(15Q)	-203	6088	2347	73
H(15R)	-266	6651	2708	73
H(15S)	-329	5960	2954	73
H(16G)	1284	5212	2311	62
H(16H)	1088	5109	2919	62
H(16I)	2141	5214	2663	62

Torsion angles [°] for CAL_9_227C.

C(6)-C(1)-C(2)-C(3)	1.6(7)
N(1)-C(1)-C(2)-C(3)	-178.7(4)
C(1)-C(2)-C(3)-C(4)	-1.0(8)
C(2)-C(3)-C(4)-C(5)	-0.6(9)
C(3)-C(4)-C(5)-C(6)	1.6(9)
C(2)-C(1)-C(6)-C(5)	-0.6(7)
N(1)-C(1)-C(6)-C(5)	179.7(4)
C(4)-C(5)-C(6)-C(1)	-1.0(8)
O(1)-C(7)-C(8)-N(1)	-2.2(5)
C(12)-C(7)-C(8)-N(1)	178.3(4)
O(1)-C(7)-C(8)-C(9)	176.7(4)
C(12)-C(7)-C(8)-C(9)	-2.8(6)
N(1)-C(8)-C(9)-C(10)	177.4(4)
C(7)-C(8)-C(9)-C(10)	-1.3(7)
C(8)-C(9)-C(10)-C(11)	3.2(7)
C(8)-C(9)-C(10)-C(13)	-176.9(4)
C(9)-C(10)-C(11)-C(12)	-1.2(7)
C(13)-C(10)-C(11)-C(12)	179.0(4)
C(10)-C(11)-C(12)-C(7)	-2.7(7)
C(10)-C(11)-C(12)-C(17)	178.7(4)
O(1)-C(7)-C(12)-C(11)	-174.9(4)
C(8)-C(7)-C(12)-C(11)	4.6(6)
O(1)-C(7)-C(12)-C(17)	3.8(7)
C(8)-C(7)-C(12)-C(17)	-176.8(4)
C(9)-C(10)-C(13)-C(14)	13.0(7)
C(11)-C(10)-C(13)-C(14)	-167.1(5)
C(9)-C(10)-C(13)-C(15)	-108.6(6)
C(11)-C(10)-C(13)-C(15)	71.2(6)
C(9)-C(10)-C(13)-C(16)	133.6(5)
C(11)-C(10)-C(13)-C(16)	-46.6(6)
C(11)-C(12)-C(17)-C(19)	113.7(5)
C(7)-C(12)-C(17)-C(19)	-64.8(6)
C(11)-C(12)-C(17)-C(20)	-126.1(5)
C(7)-C(12)-C(17)-C(20)	55.4(6)
C(11)-C(12)-C(17)-C(18)	-7.4(7)
C(7)-C(12)-C(17)-C(18)	174.1(4)
C(26)-C(21)-C(22)-C(23)	0.0(7)
N(2)-C(21)-C(22)-C(23)	179.8(4)
C(21)-C(22)-C(23)-C(24)	1.4(8)
C(22)-C(23)-C(24)-C(25)	-1.0(8)
C(23)-C(24)-C(25)-C(26)	-0.8(8)
C(24)-C(25)-C(26)-C(21)	2.2(7)
C(22)-C(21)-C(26)-C(25)	-1.8(7)
N(2)-C(21)-C(26)-C(25)	178.4(4)
N(2)-C(27)-C(28)-C(29)	178.7(4)
C(32)-C(27)-C(28)-C(29)	-1.5(7)
C(27)-C(28)-C(29)-C(30)	1.7(7)
C(27)-C(28)-C(29)-C(33)	-176.6(5)
C(28)-C(29)-C(30)-C(31)	-0.6(8)
C(33)-C(29)-C(30)-C(31)	177.6(5)
C(29)-C(30)-C(31)-C(32)	-0.6(8)
C(29)-C(30)-C(31)-C(37)	179.6(5)
C(30)-C(31)-C(32)-O(2)	-179.7(5)
C(37)-C(31)-C(32)-O(2)	0.0(8)

C(30)-C(31)-C(32)-C(27)	0.8(7)
C(37)-C(31)-C(32)-C(27)	-179.4(5)
N(2)-C(27)-C(32)-O(2)	0.5(6)
C(28)-C(27)-C(32)-O(2)	-179.3(4)
N(2)-C(27)-C(32)-C(31)	180.0(4)
C(28)-C(27)-C(32)-C(31)	0.2(7)
C(28)-C(29)-C(33)-C(34)	-52.1(7)
C(30)-C(29)-C(33)-C(34)	129.7(6)
C(28)-C(29)-C(33)-C(36A)	173.0(6)
C(30)-C(29)-C(33)-C(36A)	-5.2(9)
C(28)-C(29)-C(33)-C(35)	67.1(7)
C(30)-C(29)-C(33)-C(35)	-111.1(6)
C(28)-C(29)-C(33)-C(36)	-158.2(7)
C(30)-C(29)-C(33)-C(36)	23.6(9)
C(32)-C(31)-C(37)-C(38)	58.5(7)
C(30)-C(31)-C(37)-C(38)	-121.8(6)
C(32)-C(31)-C(37)-C(39)	-62.6(7)
C(30)-C(31)-C(37)-C(39)	117.2(6)
C(32)-C(31)-C(37)-C(40)	177.3(6)
C(30)-C(31)-C(37)-C(40)	-2.9(8)
C(46)-C(41)-C(42)-C(43)	1.5(8)
N(3)-C(41)-C(42)-C(43)	-177.2(5)
C(41)-C(42)-C(43)-C(44)	-1.0(10)
C(42)-C(43)-C(44)-C(45)	-1.1(11)
C(43)-C(44)-C(45)-C(46)	2.7(11)
C(42)-C(41)-C(46)-C(45)	0.0(8)
N(3)-C(41)-C(46)-C(45)	178.7(5)
C(44)-C(45)-C(46)-C(41)	-2.1(9)
N(3)-C(47)-C(48)-C(49)	177.4(4)
C(52)-C(47)-C(48)-C(49)	-1.3(7)
C(47)-C(48)-C(49)-C(50)	0.7(7)
C(47)-C(48)-C(49)-C(53)	-176.3(4)
C(48)-C(49)-C(50)-C(51)	0.8(7)
C(53)-C(49)-C(50)-C(51)	177.9(5)
C(49)-C(50)-C(51)-C(52)	-1.6(7)
C(49)-C(50)-C(51)-C(57)	179.5(5)
C(50)-C(51)-C(52)-O(3)	-176.5(4)
C(57)-C(51)-C(52)-O(3)	2.4(7)
C(50)-C(51)-C(52)-C(47)	0.9(7)
C(57)-C(51)-C(52)-C(47)	179.9(4)
N(3)-C(47)-C(52)-O(3)	-0.7(6)
C(48)-C(47)-C(52)-O(3)	178.2(4)
N(3)-C(47)-C(52)-C(51)	-178.4(4)
C(48)-C(47)-C(52)-C(51)	0.5(7)
C(48)-C(49)-C(53)-C(55)	-140.3(6)
C(50)-C(49)-C(53)-C(55)	42.7(7)
C(48)-C(49)-C(53)-C(56)	99.2(6)
C(50)-C(49)-C(53)-C(56)	-77.8(6)
C(48)-C(49)-C(53)-C(54)	-22.3(7)
C(50)-C(49)-C(53)-C(54)	160.7(5)
C(50)-C(51)-C(57)-C(59)	111.9(5)
C(52)-C(51)-C(57)-C(59)	-66.9(6)
C(50)-C(51)-C(57)-C(60)	-9.2(7)
C(52)-C(51)-C(57)-C(60)	172.0(5)
C(50)-C(51)-C(57)-C(58)	-127.8(5)
C(52)-C(51)-C(57)-C(58)	53.3(6)

C(66)-C(61)-C(62)-C(63)	-1.7(9)
N(4)-C(61)-C(62)-C(63)	177.5(5)
C(61)-C(62)-C(63)-C(64)	1.7(10)
C(62)-C(63)-C(64)-C(65)	0.6(11)
C(63)-C(64)-C(65)-C(66)	-2.9(11)
C(64)-C(65)-C(66)-C(61)	2.8(10)
C(62)-C(61)-C(66)-C(65)	-0.5(9)
N(4)-C(61)-C(66)-C(65)	-179.8(5)
C(72)-C(67)-C(68)-C(69)	-1.7(7)
N(4)-C(67)-C(68)-C(69)	-177.8(4)
C(67)-C(68)-C(69)-C(70)	-0.6(7)
C(67)-C(68)-C(69)-C(77)	175.9(4)
C(68)-C(69)-C(70)-C(71)	1.7(7)
C(77)-C(69)-C(70)-C(71)	-174.9(4)
C(69)-C(70)-C(71)-C(72)	-0.4(7)
C(69)-C(70)-C(71)-C(73)	179.1(4)
N(4)-C(67)-C(72)-O(4)	2.8(6)
C(68)-C(67)-C(72)-O(4)	-173.8(4)
N(4)-C(67)-C(72)-C(71)	179.7(4)
C(68)-C(67)-C(72)-C(71)	3.1(7)
C(70)-C(71)-C(72)-O(4)	174.6(4)
C(73)-C(71)-C(72)-O(4)	-4.8(7)
C(70)-C(71)-C(72)-C(67)	-2.0(6)
C(73)-C(71)-C(72)-C(67)	178.5(4)
C(72)-C(71)-C(73)-C(74)	-57.7(6)
C(70)-C(71)-C(73)-C(74)	122.8(5)
C(72)-C(71)-C(73)-C(76)	-178.6(4)
C(70)-C(71)-C(73)-C(76)	2.0(6)
C(72)-C(71)-C(73)-C(75)	61.2(6)
C(70)-C(71)-C(73)-C(75)	-118.2(5)
C(68)-C(69)-C(77)-C(79)	-122.4(6)
C(70)-C(69)-C(77)-C(79)	54.1(7)
C(68)-C(69)-C(77)-C(78)	116.7(6)
C(70)-C(69)-C(77)-C(78)	-66.7(6)
C(68)-C(69)-C(77)-C(80)	-3.3(7)
C(70)-C(69)-C(77)-C(80)	173.2(5)
C(86)-C(81)-C(82)-C(83)	-3.8(7)
N(5)-C(81)-C(82)-C(83)	174.8(4)
C(81)-C(82)-C(83)-C(84)	3.1(7)
C(82)-C(83)-C(84)-C(85)	-0.4(8)
C(83)-C(84)-C(85)-C(86)	-1.7(8)
C(82)-C(81)-C(86)-C(85)	1.7(7)
N(5)-C(81)-C(86)-C(85)	-176.9(4)
C(84)-C(85)-C(86)-C(81)	1.0(7)
N(5)-C(87)-C(88)-C(89)	-177.8(4)
C(92)-C(87)-C(88)-C(89)	-0.5(7)
C(87)-C(88)-C(89)-C(90)	-1.6(7)
C(87)-C(88)-C(89)-C(93)	177.7(4)
C(88)-C(89)-C(90)-C(91)	0.3(7)
C(93)-C(89)-C(90)-C(91)	-179.0(4)
C(89)-C(90)-C(91)-C(92)	3.0(7)
C(89)-C(90)-C(91)-C(97)	-177.8(4)
C(90)-C(91)-C(92)-O(8)	175.4(4)
C(97)-C(91)-C(92)-O(8)	-3.8(7)
C(90)-C(91)-C(92)-C(87)	-4.9(6)
C(97)-C(91)-C(92)-C(87)	175.9(4)

N(5)-C(87)-C(92)-O(8)	1.3(5)
C(88)-C(87)-C(92)-O(8)	-176.4(4)
N(5)-C(87)-C(92)-C(91)	-178.4(4)
C(88)-C(87)-C(92)-C(91)	3.9(7)
C(88)-C(89)-C(93)-C(96)	-175.0(5)
C(90)-C(89)-C(93)-C(96)	4.2(7)
C(88)-C(89)-C(93)-C(94)	64.4(6)
C(90)-C(89)-C(93)-C(94)	-116.4(5)
C(88)-C(89)-C(93)-C(95)	-55.3(6)
C(90)-C(89)-C(93)-C(95)	123.9(5)
C(90)-C(91)-C(97)-C(98)	4.6(7)
C(92)-C(91)-C(97)-C(98)	-176.3(4)
C(90)-C(91)-C(97)-C(99)	-116.1(5)
C(92)-C(91)-C(97)-C(99)	63.0(6)
C(90)-C(91)-C(97)-C(100)	123.8(5)
C(92)-C(91)-C(97)-C(100)	-57.1(6)
C(106)-C(101)-C(102)-C(103)	-0.9(7)
N(6)-C(101)-C(102)-C(103)	178.6(4)
C(101)-C(102)-C(103)-C(104)	-1.3(8)
C(102)-C(103)-C(104)-C(105)	2.6(9)
C(103)-C(104)-C(105)-C(106)	-1.6(9)
C(102)-C(101)-C(106)-C(105)	1.9(7)
N(6)-C(101)-C(106)-C(105)	-177.6(4)
C(104)-C(105)-C(106)-C(101)	-0.7(8)
N(6)-C(107)-C(108)-C(109)	179.5(4)
C(112)-C(107)-C(108)-C(109)	1.4(7)
C(107)-C(108)-C(109)-C(110)	0.9(7)
C(107)-C(108)-C(109)-C(118)	-179.5(4)
C(108)-C(109)-C(110)-C(111)	-1.4(7)
C(118)-C(109)-C(110)-C(111)	178.9(4)
C(109)-C(110)-C(111)-C(112)	-0.4(7)
C(109)-C(110)-C(111)-C(113)	-180.0(4)
C(110)-C(111)-C(112)-O(9)	-175.5(4)
C(113)-C(111)-C(112)-O(9)	4.1(7)
C(110)-C(111)-C(112)-C(107)	2.7(6)
C(113)-C(111)-C(112)-C(107)	-177.7(4)
N(6)-C(107)-C(112)-O(9)	-3.3(5)
C(108)-C(107)-C(112)-O(9)	175.1(4)
N(6)-C(107)-C(112)-C(111)	178.4(4)
C(108)-C(107)-C(112)-C(111)	-3.3(7)
C(110)-C(111)-C(113)-C(114)	-0.8(6)
C(112)-C(111)-C(113)-C(114)	179.6(4)
C(110)-C(111)-C(113)-C(115)	-121.1(5)
C(112)-C(111)-C(113)-C(115)	59.3(5)
C(110)-C(111)-C(113)-C(116)	119.5(5)
C(112)-C(111)-C(113)-C(116)	-60.1(5)
C(108)-C(109)-C(118)-C(120)	121.9(5)
C(110)-C(109)-C(118)-C(120)	-58.4(6)
C(108)-C(109)-C(118)-C(119)	-2.3(7)
C(110)-C(109)-C(118)-C(119)	177.3(5)
C(108)-C(109)-C(118)-C(117)	-119.7(5)
C(110)-C(109)-C(118)-C(117)	60.0(6)
C(126)-C(121)-C(122)-C(123)	-2.8(7)
N(7)-C(121)-C(122)-C(123)	177.1(4)
C(121)-C(122)-C(123)-C(124)	2.7(8)
C(122)-C(123)-C(124)-C(125)	-0.7(9)

C(123)-C(124)-C(125)-C(126)	-1.4(9)
C(122)-C(121)-C(126)-C(125)	0.7(8)
N(7)-C(121)-C(126)-C(125)	-179.1(4)
C(124)-C(125)-C(126)-C(121)	1.4(8)
N(7)-C(127)-C(128)-O(10)	1.0(5)
C(132)-C(127)-C(128)-O(10)	-179.2(4)
N(7)-C(127)-C(128)-C(129)	179.9(4)
C(132)-C(127)-C(128)-C(129)	-0.3(7)
O(10)-C(128)-C(129)-C(130)	175.9(4)
C(127)-C(128)-C(129)-C(130)	-2.9(6)
O(10)-C(128)-C(129)-C(138)	-3.2(7)
C(127)-C(128)-C(129)-C(138)	178.0(4)
C(128)-C(129)-C(130)-C(131)	4.5(7)
C(138)-C(129)-C(130)-C(131)	-176.4(4)
C(129)-C(130)-C(131)-C(132)	-2.7(7)
C(129)-C(130)-C(131)-C(133)	179.1(5)
C(130)-C(131)-C(132)-C(127)	-1.0(7)
C(133)-C(131)-C(132)-C(127)	177.2(4)
N(7)-C(127)-C(132)-C(131)	-177.9(4)
C(128)-C(127)-C(132)-C(131)	2.3(7)
C(132)-C(131)-C(133)-C(136)	-105.3(6)
C(130)-C(131)-C(133)-C(136)	72.8(6)
C(132)-C(131)-C(133)-C(135)	14.9(7)
C(130)-C(131)-C(133)-C(135)	-167.0(5)
C(132)-C(131)-C(133)-C(134)	135.8(5)
C(130)-C(131)-C(133)-C(134)	-46.1(6)
C(130)-C(129)-C(138)-C(140)	-111.3(5)
C(128)-C(129)-C(138)-C(140)	67.7(6)
C(130)-C(129)-C(138)-C(137)	8.4(7)
C(128)-C(129)-C(138)-C(137)	-172.6(5)
C(130)-C(129)-C(138)-C(139)	129.3(5)
C(128)-C(129)-C(138)-C(139)	-51.7(6)
C(146)-C(141)-C(142)-C(143)	0.2(8)
N(8)-C(141)-C(142)-C(143)	177.4(5)
C(141)-C(142)-C(143)-C(144)	0.1(9)
C(142)-C(143)-C(144)-C(145)	-1.5(10)
C(143)-C(144)-C(145)-C(146)	2.5(9)
C(142)-C(141)-C(146)-C(145)	0.8(7)
N(8)-C(141)-C(146)-C(145)	-176.5(4)
C(144)-C(145)-C(146)-C(141)	-2.2(8)
N(8)-C(147)-C(148)-C(149)	-177.8(5)
C(152)-C(147)-C(148)-C(149)	2.2(7)
C(147)-C(148)-C(149)-C(150)	1.3(8)
C(147)-C(148)-C(149)-C(153)	-179.0(5)
C(148)-C(149)-C(150)-C(151)	-2.3(8)
C(153)-C(149)-C(150)-C(151)	178.0(5)
C(149)-C(150)-C(151)-C(152)	-0.3(8)
C(149)-C(150)-C(151)-C(157)	178.9(5)
N(8)-C(147)-C(152)-O(11)	-5.3(6)
C(148)-C(147)-C(152)-O(11)	174.7(4)
N(8)-C(147)-C(152)-C(151)	175.0(4)
C(148)-C(147)-C(152)-C(151)	-5.0(7)
C(150)-C(151)-C(152)-O(11)	-175.7(4)
C(157)-C(151)-C(152)-O(11)	5.0(7)
C(150)-C(151)-C(152)-C(147)	3.9(7)
C(157)-C(151)-C(152)-C(147)	-175.3(4)

C(150)-C(149)-C(153)-C(161)	-19.7(11)
C(148)-C(149)-C(153)-C(161)	160.6(9)
C(150)-C(149)-C(153)-C(155)	125.2(7)
C(148)-C(149)-C(153)-C(155)	-54.5(8)
C(150)-C(149)-C(153)-C(154)	-117.9(7)
C(148)-C(149)-C(153)-C(154)	62.4(8)
C(150)-C(149)-C(153)-C(156)	10.6(9)
C(148)-C(149)-C(153)-C(156)	-169.1(7)
C(150)-C(151)-C(157)-C(159)	-4.6(7)
C(152)-C(151)-C(157)-C(159)	174.6(5)
C(150)-C(151)-C(157)-C(160)	116.4(5)
C(152)-C(151)-C(157)-C(160)	-64.4(6)
C(150)-C(151)-C(157)-C(158)	-123.5(5)
C(152)-C(151)-C(157)-C(158)	55.7(6)
C(9)-C(8)-N(1)-C(1)	3.2(7)
C(7)-C(8)-N(1)-C(1)	-177.9(4)
C(9)-C(8)-N(1)-Re(1)	-171.2(4)
C(7)-C(8)-N(1)-Re(1)	7.6(5)
C(2)-C(1)-N(1)-C(8)	120.5(5)
C(6)-C(1)-N(1)-C(8)	-59.8(6)
C(2)-C(1)-N(1)-Re(1)	-65.3(5)
C(6)-C(1)-N(1)-Re(1)	114.4(4)
C(28)-C(27)-N(2)-C(21)	5.1(7)
C(32)-C(27)-N(2)-C(21)	-174.7(4)
C(28)-C(27)-N(2)-Re(1)	174.6(4)
C(32)-C(27)-N(2)-Re(1)	-5.2(5)
C(26)-C(21)-N(2)-C(27)	-105.8(5)
C(22)-C(21)-N(2)-C(27)	74.5(5)
C(26)-C(21)-N(2)-Re(1)	85.4(5)
C(22)-C(21)-N(2)-Re(1)	-94.4(4)
C(48)-C(47)-N(3)-C(41)	0.9(7)
C(52)-C(47)-N(3)-C(41)	179.7(4)
C(48)-C(47)-N(3)-Re(2)	-178.8(4)
C(52)-C(47)-N(3)-Re(2)	0.0(5)
C(42)-C(41)-N(3)-C(47)	105.5(5)
C(46)-C(41)-N(3)-C(47)	-73.2(6)
C(42)-C(41)-N(3)-Re(2)	-74.9(5)
C(46)-C(41)-N(3)-Re(2)	106.4(5)
C(72)-C(67)-N(4)-C(61)	-176.5(4)
C(68)-C(67)-N(4)-C(61)	-0.1(7)
C(72)-C(67)-N(4)-Re(2)	-3.9(5)
C(68)-C(67)-N(4)-Re(2)	172.5(4)
C(62)-C(61)-N(4)-C(67)	87.0(6)
C(66)-C(61)-N(4)-C(67)	-93.8(6)
C(62)-C(61)-N(4)-Re(2)	-85.2(6)
C(66)-C(61)-N(4)-Re(2)	94.1(5)
C(88)-C(87)-N(5)-C(81)	-5.2(6)
C(92)-C(87)-N(5)-C(81)	177.3(4)
C(88)-C(87)-N(5)-Re(3)	174.2(4)
C(92)-C(87)-N(5)-Re(3)	-3.3(5)
C(82)-C(81)-N(5)-C(87)	-112.2(5)
C(86)-C(81)-N(5)-C(87)	66.4(5)
C(82)-C(81)-N(5)-Re(3)	68.4(5)
C(86)-C(81)-N(5)-Re(3)	-113.0(4)
C(108)-C(107)-N(6)-C(101)	-7.2(7)
C(112)-C(107)-N(6)-C(101)	171.0(4)

C(108)-C(107)-N(6)-Re(3)	-174.2(4)
C(112)-C(107)-N(6)-Re(3)	4.1(5)
C(106)-C(101)-N(6)-C(107)	-73.8(6)
C(102)-C(101)-N(6)-C(107)	106.7(5)
C(106)-C(101)-N(6)-Re(3)	92.3(5)
C(102)-C(101)-N(6)-Re(3)	-87.2(5)
C(132)-C(127)-N(7)-C(121)	-2.0(6)
C(128)-C(127)-N(7)-C(121)	177.8(4)
C(132)-C(127)-N(7)-Re(4)	176.2(3)
C(128)-C(127)-N(7)-Re(4)	-4.0(5)
C(126)-C(121)-N(7)-C(127)	67.8(6)
C(122)-C(121)-N(7)-C(127)	-112.0(5)
C(126)-C(121)-N(7)-Re(4)	-110.4(4)
C(122)-C(121)-N(7)-Re(4)	69.8(5)
C(152)-C(147)-N(8)-C(141)	175.2(4)
C(148)-C(147)-N(8)-C(141)	-4.8(7)
C(152)-C(147)-N(8)-Re(4)	2.2(5)
C(148)-C(147)-N(8)-Re(4)	-177.9(4)
C(142)-C(141)-N(8)-C(147)	-77.6(6)
C(146)-C(141)-N(8)-C(147)	99.7(5)
C(142)-C(141)-N(8)-Re(4)	94.9(5)
C(146)-C(141)-N(8)-Re(4)	-87.8(5)
C(12)-C(7)-O(1)-Re(1)	175.5(3)
C(8)-C(7)-O(1)-Re(1)	-3.9(5)
C(31)-C(32)-O(2)-Re(1)	-174.9(4)
C(27)-C(32)-O(2)-Re(1)	4.6(5)
C(51)-C(52)-O(3)-Re(2)	178.6(4)
C(47)-C(52)-O(3)-Re(2)	1.1(5)
C(67)-C(72)-O(4)-Re(2)	-0.5(5)
C(71)-C(72)-O(4)-Re(2)	-177.3(3)
C(91)-C(92)-O(8)-Re(3)	-179.2(3)
C(87)-C(92)-O(8)-Re(3)	1.1(5)
C(111)-C(112)-O(9)-Re(3)	179.4(3)
C(107)-C(112)-O(9)-Re(3)	1.1(5)
C(127)-C(128)-O(10)-Re(4)	2.4(5)
C(129)-C(128)-O(10)-Re(4)	-176.5(3)
C(147)-C(152)-O(11)-Re(4)	6.4(5)
C(151)-C(152)-O(11)-Re(4)	-173.9(4)
Re(2)-O(5)-Re(1)-O(6)	12.4(4)
Re(2)-O(5)-Re(1)-O(2)	119.8(4)
Re(2)-O(5)-Re(1)-N(2)	149.1(4)
Re(2)-O(5)-Re(1)-N(1)	-77.2(4)
Re(2)-O(5)-Re(1)-O(1)	-150.3(4)
C(32)-O(2)-Re(1)-O(6)	-100.5(3)
C(32)-O(2)-Re(1)-O(5)	163.4(3)
C(32)-O(2)-Re(1)-N(2)	-5.7(3)
C(32)-O(2)-Re(1)-N(1)	56.4(6)
C(32)-O(2)-Re(1)-O(1)	78.8(3)
C(27)-N(2)-Re(1)-O(6)	112.5(3)
C(21)-N(2)-Re(1)-O(6)	-78.4(4)
C(27)-N(2)-Re(1)-O(5)	-24.1(6)
C(21)-N(2)-Re(1)-O(5)	144.9(4)
C(27)-N(2)-Re(1)-O(2)	5.8(3)
C(21)-N(2)-Re(1)-O(2)	174.9(4)
C(27)-N(2)-Re(1)-N(1)	-158.7(3)
C(21)-N(2)-Re(1)-N(1)	10.3(4)

C(27)-N(2)-Re(1)-O(1)	-84.7(3)
C(21)-N(2)-Re(1)-O(1)	84.3(3)
C(8)-N(1)-Re(1)-O(6)	173.9(3)
C(1)-N(1)-Re(1)-O(6)	-0.2(3)
C(8)-N(1)-Re(1)-O(5)	-88.5(3)
C(1)-N(1)-Re(1)-O(5)	97.3(3)
C(8)-N(1)-Re(1)-O(2)	15.9(6)
C(1)-N(1)-Re(1)-O(2)	-158.3(4)
C(8)-N(1)-Re(1)-N(2)	75.7(3)
C(1)-N(1)-Re(1)-N(2)	-98.5(3)
C(8)-N(1)-Re(1)-O(1)	-7.4(3)
C(1)-N(1)-Re(1)-O(1)	178.5(4)
C(7)-O(1)-Re(1)-O(6)	10.4(6)
C(7)-O(1)-Re(1)-O(5)	109.2(3)
C(7)-O(1)-Re(1)-O(2)	-167.1(3)
C(7)-O(1)-Re(1)-N(2)	-90.0(3)
C(7)-O(1)-Re(1)-N(1)	6.1(3)
Re(1)-O(5)-Re(2)-O(7)	-5.7(4)
Re(1)-O(5)-Re(2)-O(4)	98.8(4)
Re(1)-O(5)-Re(2)-N(4)	131.8(4)
Re(1)-O(5)-Re(2)-N(3)	-92.5(4)
Re(1)-O(5)-Re(2)-O(3)	-166.1(4)
C(72)-O(4)-Re(2)-O(7)	-96.2(3)
C(72)-O(4)-Re(2)-O(5)	166.4(3)
C(72)-O(4)-Re(2)-N(4)	-1.3(3)
C(72)-O(4)-Re(2)-N(3)	69.5(8)
C(72)-O(4)-Re(2)-O(3)	81.4(3)
C(67)-N(4)-Re(2)-O(7)	106.8(3)
C(61)-N(4)-Re(2)-O(7)	-80.8(4)
C(67)-N(4)-Re(2)-O(5)	-30.8(6)
C(61)-N(4)-Re(2)-O(5)	141.6(4)
C(67)-N(4)-Re(2)-O(4)	2.8(3)
C(61)-N(4)-Re(2)-O(4)	175.2(4)
C(67)-N(4)-Re(2)-N(3)	-166.3(3)
C(61)-N(4)-Re(2)-N(3)	6.1(4)
C(67)-N(4)-Re(2)-O(3)	-93.3(3)
C(61)-N(4)-Re(2)-O(3)	79.1(4)
C(47)-N(3)-Re(2)-O(7)	178.8(3)
C(41)-N(3)-Re(2)-O(7)	-0.8(4)
C(47)-N(3)-Re(2)-O(5)	-82.9(3)
C(41)-N(3)-Re(2)-O(5)	97.5(4)
C(47)-N(3)-Re(2)-O(4)	12.7(9)
C(41)-N(3)-Re(2)-O(4)	-166.9(6)
C(47)-N(3)-Re(2)-N(4)	81.4(3)
C(41)-N(3)-Re(2)-N(4)	-98.2(4)
C(47)-N(3)-Re(2)-O(3)	0.4(3)
C(41)-N(3)-Re(2)-O(3)	-179.2(4)
C(52)-O(3)-Re(2)-O(7)	-5.3(6)
C(52)-O(3)-Re(2)-O(5)	98.1(3)
C(52)-O(3)-Re(2)-O(4)	-178.4(3)
C(52)-O(3)-Re(2)-N(4)	-101.8(3)
C(52)-O(3)-Re(2)-N(3)	-0.8(3)
Re(4)-O(12)-Re(3)-O(13)	-4.5(5)
Re(4)-O(12)-Re(3)-O(9)	-107.5(4)
Re(4)-O(12)-Re(3)-N(6)	-144.4(4)
Re(4)-O(12)-Re(3)-N(5)	83.0(4)

Re(4)-O(12)-Re(3)-O(8)	156.3(5)
C(112)-O(9)-Re(3)-O(13)	96.8(3)
C(112)-O(9)-Re(3)-O(12)	-164.8(3)
C(112)-O(9)-Re(3)-N(6)	0.8(3)
C(112)-O(9)-Re(3)-N(5)	-57.7(8)
C(112)-O(9)-Re(3)-O(8)	-80.6(3)
C(107)-N(6)-Re(3)-O(13)	-105.1(3)
C(101)-N(6)-Re(3)-O(13)	88.6(4)
C(107)-N(6)-Re(3)-O(12)	35.0(6)
C(101)-N(6)-Re(3)-O(12)	-131.4(4)
C(107)-N(6)-Re(3)-O(9)	-2.7(3)
C(101)-N(6)-Re(3)-O(9)	-169.1(4)
C(107)-N(6)-Re(3)-N(5)	168.0(3)
C(101)-N(6)-Re(3)-N(5)	1.7(4)
C(107)-N(6)-Re(3)-O(8)	94.6(3)
C(101)-N(6)-Re(3)-O(8)	-71.8(3)
C(87)-N(5)-Re(3)-O(13)	-175.9(3)
C(81)-N(5)-Re(3)-O(13)	3.4(3)
C(87)-N(5)-Re(3)-O(12)	84.9(3)
C(81)-N(5)-Re(3)-O(12)	-95.8(3)
C(87)-N(5)-Re(3)-O(9)	-20.8(9)
C(81)-N(5)-Re(3)-O(9)	158.6(6)
C(87)-N(5)-Re(3)-N(6)	-77.7(3)
C(81)-N(5)-Re(3)-N(6)	101.6(3)
C(87)-N(5)-Re(3)-O(8)	3.0(3)
C(81)-N(5)-Re(3)-O(8)	-177.7(4)
C(92)-O(8)-Re(3)-O(13)	1.0(6)
C(92)-O(8)-Re(3)-O(12)	-103.6(3)
C(92)-O(8)-Re(3)-O(9)	173.5(3)
C(92)-O(8)-Re(3)-N(6)	96.8(3)
C(92)-O(8)-Re(3)-N(5)	-2.2(3)
Re(3)-O(12)-Re(4)-O(14)	-6.2(5)
Re(3)-O(12)-Re(4)-O(11)	-108.6(4)
Re(3)-O(12)-Re(4)-N(8)	-144.4(4)
Re(3)-O(12)-Re(4)-N(7)	81.4(4)
Re(3)-O(12)-Re(4)-O(10)	154.6(4)
C(152)-O(11)-Re(4)-O(14)	91.9(3)
C(152)-O(11)-Re(4)-O(12)	-170.3(3)
C(152)-O(11)-Re(4)-N(8)	-4.0(3)
C(152)-O(11)-Re(4)-N(7)	-63.2(8)
C(152)-O(11)-Re(4)-O(10)	-85.1(3)
C(147)-N(8)-Re(4)-O(14)	-100.8(3)
C(141)-N(8)-Re(4)-O(14)	86.6(4)
C(147)-N(8)-Re(4)-O(12)	37.5(6)
C(141)-N(8)-Re(4)-O(12)	-135.1(4)
C(147)-N(8)-Re(4)-O(11)	0.9(3)
C(141)-N(8)-Re(4)-O(11)	-171.7(4)
C(147)-N(8)-Re(4)-N(7)	171.9(3)
C(141)-N(8)-Re(4)-N(7)	-0.7(4)
C(147)-N(8)-Re(4)-O(10)	99.1(3)
C(141)-N(8)-Re(4)-O(10)	-73.5(4)
C(127)-N(7)-Re(4)-O(14)	-174.5(3)
C(121)-N(7)-Re(4)-O(14)	3.6(3)
C(127)-N(7)-Re(4)-O(12)	87.0(3)
C(121)-N(7)-Re(4)-O(12)	-95.0(3)
C(127)-N(7)-Re(4)-O(11)	-18.7(9)

C(121)-N(7)-Re(4)-O(11)	159.3(6)
C(127)-N(7)-Re(4)-N(8)	-76.4(3)
C(121)-N(7)-Re(4)-N(8)	101.6(3)
C(127)-N(7)-Re(4)-O(10)	4.0(3)
C(121)-N(7)-Re(4)-O(10)	-178.0(4)
C(128)-O(10)-Re(4)-O(14)	1.2(6)
C(128)-O(10)-Re(4)-O(12)	-104.3(3)
C(128)-O(10)-Re(4)-O(11)	172.6(3)
C(128)-O(10)-Re(4)-N(8)	96.1(3)
C(128)-O(10)-Re(4)-N(7)	-3.4(3)

DFT Simulated EPR Parameters

Table S2. Computed EPR parameters

Component[a]	*g*	*A(^{187}Re) [MHz]*	*A(^{14}N-1) [MHz]*	*A(^{14}N-2) [MHz]*	*A(^{1}H-1) [MHz]*	*A(^{1}H-2) [MHz]*	*A(^{1}H-3) [MHz]*	*A(^{1}H-4) [MHz]*
x (*α*)	1.967	-128.63 (82.0)	3.66 (145.4)	20.16 (-23.7)	2.24 (-173.8)	-6.75 (25.7)	-1.01 (10.4)	-6.64 (-101.4)
y (*β*)	2.023	-71.82 (40.1)	20.29 (18.3)	3.78 (36.5)	-0.94 (29.6)	-2.15 (32.4)	0.15 (37.5)	-8.65 (42.6)
z (*γ*)	2.031	-52.37 (-70.6)	3.89 (171.6)	3.58 (13.3)	0.24 (129.8)	-8.79 (-39.0)	2.16 (-13.4)	-2.09 (138.0)
|avg|	2.007	84.27	9.28	9.17	1.14	5.90	1.11	5.79

[a]Euler angles necessary to rotate hyperfine tensors into the *g* tensor frame given in parentheses

DFT Energetics

Table S3. Computed BDFEs (ΔG) for the Re–ORe bond in $[Re_2(\mu\text{-}O)(O)_2(ap)_2(isq^{\bullet})_2]$ with various functionals.

**Functional**	$\boldsymbol{D}_{[((\boldsymbol{L})(\boldsymbol{L}^{\bullet})\boldsymbol{Re}^{V}(\boldsymbol{O}))_2(\mu-\boldsymbol{O})]/[(\boldsymbol{L})(\boldsymbol{L}^{\bullet})\boldsymbol{Re}^{V}(\boldsymbol{O})]}$
M06-L	-27 (kcal mol^{-1})
BP86	-30 (kcal mol^{-1})
BLYP	-20 (kcal mol^{-1})
PBE	-23 (kcal mol^{-1})
TPSS	-27 (kcal mol^{-1})
B3LYP	-18 (kcal mol^{-1})
PBE0	-18 (kcal mol^{-1})

DFT Coordinates, .xyz format

List S1. $[Re(O)_2(ap)(isq^{\bullet})]$, M06-L

```
Re    7.27502736884518    4.73959811574034    16.05238825825868
O     8.39424084585549    3.78840578712417    14.56656219379696
O     6.21901221119756    2.93543914687119    15.92722943927383
O     8.53679872158603    5.87545601077945    16.28708660698627
```

O	5.96842318146225	5.46472671884359	16.89177117714947
N	6.25394061248563	5.04792445183241	14.28672954127902
N	8.28368244633548	3.40892846159207	17.25022191045721
C	4.97124550149193	5.65361911893314	14.29575208259495
C	3.82973364245150	4.88406740019916	14.07906962630707
H	3.93332021966124	3.82179441424119	13.89908088923809
C	7.84522357636908	3.59725943712128	13.40593742173218
C	2.58044074778922	5.47574700453043	14.13006532917346
H	1.69771015413648	4.87036747531612	13.97544085306807
C	6.75756132639960	1.86281105093875	16.41921968802403
C	7.91832828146440	2.08920852891427	17.20542472957933
C	5.97422056978194	4.23100853217630	11.96668606385247
H	5.07702117967286	4.80804411653198	11.81009069438163
C	8.08277745938206	-0.26090638613129	17.65857731364235
C	6.94121065727068	-0.45964747360234	16.84881523896916
H	6.59213542160040	-1.47494027459960	16.72192137755140
C	6.63850083256773	4.31712899206010	13.19376409126244
C	5.00455246604950	0.33873809572637	15.37542108205997
C	2.45873758800706	6.83265493662980	14.39427837557237
H	1.48093185155993	7.29195905008717	14.43501910046840
C	6.24898631771113	0.55888732385922	16.22603338164733
C	10.70976841959197	3.19755052082309	17.35987469186998
H	10.66411597138968	2.47855301597971	16.55222325263164
C	9.52762896571389	3.76150780852644	17.83592529394634
C	8.55454882155543	1.02208915442812	17.84670203930198
H	9.41380339340467	1.21847935016025	18.46792197218740
C	11.96568670282420	4.51894273651877	18.92542247172633
H	12.91513648045261	4.81722292628764	19.34767124194059
C	4.62783709944063	-1.13687362754261	15.29527814419441
H	5.41791322377312	-1.73605536460065	14.84258938727462
H	3.73706355701242	-1.25110666099396	14.67937952975793
H	4.40445136879050	-1.55666540820044	16.27587471459719
C	8.75535376044159	-1.47804597762107	18.28074379645991
C	9.57132243586566	4.71264312039646	18.84794228721173
H	8.64739472133645	5.15562760244112	19.19397290423503
C	3.59514959194535	7.59382135673335	14.62138592471488
H	3.50704188126992	8.64913622682639	14.83933143318011
C	5.73341704604108	3.16416710683119	9.66910216776327
C	3.82548501303327	1.10145505790140	15.99570012360971
H	3.62060543092408	0.74622178245566	17.00501535943691
H	2.92460861995679	0.94765295654571	15.40058149947796
H	4.02034482853332	2.16932844064765	16.04722208066442
C	6.45943666480400	3.38631986556929	10.98933005768151
C	10.78847373246108	5.08361757451628	19.39263688248885
H	10.81761463180869	5.82168048048779	20.18207487905373
C	8.37476617529753	2.75018855408369	12.40704981173806
C	4.84869666788339	7.00857027661693	14.57913881698125
H	5.74103045614823	7.58866713259625	14.77143599667038
C	11.92237035426432	3.57945123935099	17.90515824330597
H	12.83856043988044	3.15078877156237	17.52245738558008

C	7.65023376328520	2.66639869976312	11.23631119580057
H	8.00622599503605	2.01080897284530	10.45402376454295
C	5.24753653727383	0.84399663475915	13.94664450285517
H	5.53358537896104	1.89379223608122	13.93039021025886
H	4.34041266028499	0.73293343211758	13.35019806297733
H	6.03549347767119	0.27364328675678	13.45405345618692
C	9.19223989776363	-2.43150542269634	17.16161776764424
H	8.35008730224904	-2.76805357448948	16.55918151847159
H	9.67317631480591	-3.31626738195628	17.57919923912588
H	9.90322168101530	-1.94745335045752	16.49281499283789
C	10.80345051737054	2.98257994189093	12.91905452681643
H	10.95975796821506	3.63338820632253	12.05936267593436
H	11.73680567113334	2.45054850673850	13.10584826678092
H	10.59168613870615	3.60941214796134	13.78076425989572
C	9.66850672382767	1.98475887480668	12.65253780900824
C	9.98888557541759	-1.10440975411413	19.09595155761969
H	10.74141411790156	-0.60440742760237	18.48611032855144
H	10.44585035243546	-2.00316258458035	19.50786089507163
H	9.74500690744136	-0.44929948665666	19.93167292102011
C	9.50805241300352	1.05162388209451	13.86029932640820
H	9.22054711806864	1.59539330136766	14.75755222357739
H	10.44854584397796	0.53742784445051	14.06337286049942
H	8.75118349539751	0.28955079102171	13.67332074370116
C	4.48917305227033	4.03628274563208	9.54297754753970
H	3.76274731999527	3.82795471026610	10.32832464751286
H	3.99967548239020	3.85072738818939	8.58782517453621
H	4.73269591775167	5.09751453336535	9.58504710428127
C	7.76082491746066	-2.19658870696882	19.19814043914883
H	7.43948293651662	-1.55028990594266	20.01410166532055
H	8.21563483665355	-3.08638932348826	19.63413929284545
H	6.86981761118020	-2.51361995365385	18.65862145242739
C	6.66804591358370	3.48368055718397	8.49827806545096
H	6.98934958062874	4.52412832871228	8.52458548049774
H	6.15933374941643	3.31433856412120	7.54911698786528
H	7.56174122968075	2.86186802747601	8.50464703311721
C	10.05540549271465	1.13131032691448	11.44895063661571
H	9.29762944465421	0.38126469512212	11.22207168451947
H	10.98465268721615	0.60317375566451	11.65858789598867
H	10.21522693723206	1.73377035228560	10.55497513349236
C	5.30672935046871	1.69265918193687	9.59051614756684
H	6.16379681868411	1.02115324375987	9.62466831674155
H	4.77152526001131	1.49781342549667	8.66092135273222
H	4.64997997326759	1.43107222245911	10.41993698020136

List S2. [Re(O)$_2$(ap)(isq$^\bullet$)], CPCM (toluene), M06-L

Re	7.27549938173157	4.72627268089599	16.04133100405516
O	8.38564434759906	3.77032020820596	14.55656499582347
O	6.21803581874865	2.92593726892111	15.93558541660299
O	8.54149680371797	5.86648761198275	16.26672673442352
O	5.97085967278454	5.45935843131084	16.88652826732535

N	6.25057404920034	5.03228311156881	14.28180428210095
N	8.28748799450914	3.40572094589311	17.24258827839858
C	4.97142954919517	5.64864527224180	14.28902230959258
C	3.82438320151897	4.88288515591973	14.08875481368337
H	3.91917717546786	3.81804718083798	13.91885912499891
C	7.83621381695351	3.58086607078391	13.39438126095994
C	2.57860613751566	5.48346597049399	14.13885078140942
H	1.69124028904315	4.88186364012167	13.99673807675616
C	6.75945281872581	1.85397321156262	16.42871423590970
C	7.92556725421215	2.08543854629822	17.20608275233375
C	5.96741567649662	4.23024991671982	11.95734244731445
H	5.07287776087596	4.81189792373263	11.80247377621758
C	8.09561387703021	-0.26124524718096	17.67096211031801
C	6.94740807174706	-0.46521515135083	16.86995700519265
H	6.59821089283583	-1.48130530540917	16.75105426479804
C	6.63104826353986	4.30413860195986	13.18566380772358
C	4.99723890699363	0.32093667964074	15.40924902821945
C	2.46750113993864	6.84527568051501	14.38471093546843
H	1.49293121002249	7.31163709552191	14.42439095736568
C	6.24924630324184	0.54913846783549	16.24663757202867
C	10.71625703479176	3.22170800222166	17.35434616754678
H	10.68251376115528	2.51383509267250	16.53637587133464
C	9.52708088938799	3.76449160584272	17.83727448143172
C	8.56808250474435	1.02296489023076	17.84917299931836
H	9.43142494806305	1.22230830109454	18.46376155368335
C	11.95285801981169	4.53002190363981	18.94763164964677
H	12.89765302742854	4.83103339933585	19.37845021499653
C	4.62540524606913	-1.15669016981212	15.34136234618254
H	5.41365649441151	-1.75441772011875	14.88370323931477
H	3.72817673431749	-1.27626021990508	14.73600575638235
H	4.41598739573215	-1.57122328091109	16.32723362432667
C	8.77298233039765	-1.47438131814136	18.29499146805168
C	9.55527615075503	4.69619163816920	18.86784848791772
H	8.62491355453674	5.11874678158208	19.22286038916426
C	3.61058866154624	7.60319224045208	14.59243280438478
H	3.53080946546702	8.66275681572487	14.79261653660229
C	5.73939221508904	3.19797829350134	9.64273235943863
C	3.82154402477863	1.08287515995063	16.03664253053428
H	3.62815002169952	0.73277907209655	17.05015005520448
H	2.91654398992582	0.92182588918245	15.45008090054406
H	4.01247773239387	2.15216984057751	16.07789530535831
C	6.45446194810515	3.39492068185167	10.97274265788688
C	10.76708825112514	5.07148317539715	19.42330975635293
H	10.78446783752291	5.79253344210317	20.22879045898870
C	8.36371155023859	2.73642046154745	12.39206005547185
C	4.86082455250737	7.00896145425633	14.55151344532332
H	5.75825265046188	7.58730538426697	14.72555011298575
C	11.92346626135397	3.60843845710673	17.91005907744076
H	12.84524580543563	3.19535530220686	17.52361335932295
C	7.64102529260184	2.66546652017667	11.21887372667638

H	7.99667721738615	2.01562949091420	10.43177849747392
C	5.22464891781475	0.81975518805200	13.97599804911922
H	5.49509730263474	1.87387417210969	13.95173866377816
H	4.31547480265814	0.69192269063186	13.38667186867045
H	6.01817760727774	0.25634236211258	13.48417357211334
C	9.20406538796932	-2.43255243403445	17.17737645928316
H	8.35868532891111	-2.77034863817658	16.58001167936739
H	9.68460425428727	-3.31546365546557	17.59912674135826
H	9.91510452029524	-1.95253684766256	16.50546096891621
C	10.79551754555721	2.94884215414594	12.89909965907235
H	10.95387599938044	3.60119753028426	12.04081165802331
H	11.72440350206456	2.40784387346749	13.08147161517409
H	10.59190603423615	3.57405047657990	13.76421604392413
C	9.65215946123362	1.96059877835686	12.63280242232471
C	10.01025061749213	-1.09539831272962	19.10189604626138
H	10.75905634747221	-0.59814451679478	18.48511284126326
H	10.46845435823237	-1.99291990894047	19.51500330862743
H	9.76945341609308	-0.43617311402402	19.93540138267905
C	9.48695572162261	1.02534672639573	13.83842293348632
H	9.20986887506305	1.56947338313623	14.73907929985392
H	10.42333018219879	0.50182398374905	14.03511373034463
H	8.72092065851075	0.27253168771421	13.65022595899860
C	4.50203874679059	4.08026611838248	9.51981749132448
H	3.76768491824605	3.86407492394352	10.29561940069513
H	4.02131874138426	3.91183264932826	8.55711123342436
H	4.75310416077461	5.13891977152899	9.58093574606388
C	7.78329058951420	-2.18979748828049	19.22059636267341
H	7.46543190007257	-1.53975267358612	20.03518792676070
H	8.24437708508032	-3.07550533331683	19.65812391633870
H	6.89128504192417	-2.51254715353260	18.68606516136464
C	6.68865396471310	3.53153615562370	8.48736007536831
H	7.01569677120632	4.56965276696806	8.53475186448536
H	6.18613862128552	3.38198926604314	7.53167068636423
H	7.57707985944080	2.90209136709882	8.49170703961953
C	10.03014143229566	1.10760397817471	11.42600889132919
H	9.26469086101898	0.36600236375742	11.19738203263429
H	10.95462139391204	0.57102210781758	11.63532684546378
H	10.19433538489367	1.71191243226976	10.53412343623239
C	5.30643754538460	1.73050772543424	9.53316487014463
H	6.15976119243051	1.05423023983338	9.56807671846451
H	4.78558513557950	1.55773001258128	8.59119978307094
H	4.63292383015914	1.45940863478625	10.34602648722654

List S3. $[Re(O)_2(ap)(isq^{\bullet})]$, CPCM (water), M06-L

Re	7.24764871556098	4.73829867222094	16.05524055942604
O	8.36275793471697	3.77535794063125	14.59163461748722
O	6.18001050558706	2.94551147328262	15.96754724767436
O	8.51318745954376	5.88671649643203	16.28790170076534
O	5.93366930506986	5.48012008040264	16.89064830771609
N	6.23812946034002	5.03561747172672	14.28863233552004

N	8.25681714357162	3.42204894081853	17.25843957471810
C	4.96315912949555	5.66280889011696	14.27566446803441
C	3.81194153990963	4.90260391963129	14.07734163853163
H	3.89824161692532	3.83495384466721	13.92046497376465
C	7.82602747520292	3.57517337154882	13.42341073278170
C	2.57039908428207	5.51458631451889	14.10631091288200
H	1.67948344610787	4.91869547196540	13.96238366840520
C	6.72430809503019	1.87056215013243	16.45232678719169
C	7.90126709724073	2.10079135334290	17.21515003809076
C	5.97534537642370	4.22174136356110	11.96368833271792
H	5.08457918627070	4.80466523690448	11.79257644212720
C	8.09769472512823	-0.24913472093718	17.64346559456012
C	6.92789719976312	-0.45007859761763	16.87101000457795
H	6.58170023805784	-1.46648914865649	16.74753395866271
C	6.62534400040191	4.29970615182294	13.19882734973615
C	4.95134499769508	0.33609480989379	15.44824278507526
C	2.46873330766229	6.88150635739171	14.32874944815625
H	1.49786081825997	7.35681329064583	14.34953480703502
C	6.21227667248472	0.56586521704030	16.27157551338461
C	10.69013890523496	3.27483398972559	17.30977003062120
H	10.64989015466866	2.60719141608135	16.45836584974154
C	9.50454936244790	3.77664856842117	17.84301967307046
C	8.56528210118653	1.03548506906054	17.83023670237094
H	9.44419050385414	1.23018249503439	18.42384994487982
C	11.94060713212654	4.52513235921910	18.93987507938828
H	12.88952395131965	4.81744057278024	19.36768173535333
C	4.57780094898552	-1.14154971170776	15.39153525674603
H	5.35938958775153	-1.74082887475411	14.92494562011803
H	3.67168310322306	-1.26148207466677	14.79976910845372
H	4.38411269349271	-1.55140303104731	16.38254606372368
C	8.81359363320785	-1.46704026430092	18.20961804637626
C	9.53881318295489	4.65905419263975	18.91563717980634
H	8.60953758069418	5.04757879920954	19.31085065282849
C	3.61667576735276	7.63444352880057	14.53230302036397
H	3.54384506477733	8.69865942378476	14.70962022140945
C	5.77365659793561	3.17647103889825	9.65259073582376
C	3.78114733759006	1.10227961043377	16.08053689350747
H	3.59727490553724	0.76029176311910	17.09866802211175
H	2.87204992427865	0.93504492169629	15.50255048879385
H	3.97095648825288	2.17246633842387	16.10914027245583
C	6.47280980579546	3.37902081639473	10.98994764832555
C	10.75699766744436	5.02638154780691	19.46443754759007
H	10.78032398439289	5.70604396203084	20.30518250940878
C	8.36412585713884	2.72154028316976	12.43551074855976
C	4.86261245073756	7.02895038781798	14.51218361688220
H	5.76373161203425	7.60447157119842	14.67777326016583
C	11.90340502241363	3.65294497922578	17.85986871204038
H	12.82279919463979	3.26940133152627	17.43880756925891
C	7.65369464742655	2.64510403709244	11.25496918960128
H	8.01462930905055	1.98703349610659	10.47734046221056

C	5.16862896878249	0.82786418320195	14.01077566090642
H	5.43523455464678	1.88322515819856	13.97993304494470
H	4.25511799694685	0.69467112065252	13.42972661326858
H	5.96043580127455	0.26355998518000	13.51698512610388
C	9.21643517858852	-2.38268033900614	17.04597208280449
H	8.35110304088298	-2.73614366626905	16.48749482090909
H	9.74731642565411	-3.25662857997664	17.42292232266733
H	9.87383192189644	-1.86098000138538	16.35026036768200
C	10.78953991519702	2.91232167806500	12.99967917094391
H	10.95912148372531	3.58771772468332	12.16139552342145
H	11.71281797334717	2.35944464531233	13.17390456178714
H	10.58367868744014	3.51182558223828	13.88287408472519
C	9.64423845695300	1.93721027673734	12.69730040654746
C	10.07358714283675	-1.09029672871573	18.98127453388678
H	10.79585549780756	-0.57290604969493	18.34926312518540
H	10.55333475096172	-1.99175698832513	19.35942948599256
H	9.85382968256979	-0.45068860444886	19.83576469462440
C	9.44771534386300	0.99088084007135	13.88972841529606
H	9.15527071696032	1.52751105101937	14.79043049956104
H	10.37810972615313	0.46219202708943	14.10215767474663
H	8.68296920604842	0.24305808448134	13.67716253736241
C	4.54883786704186	4.07166620880887	9.50334186735659
H	3.80107771361585	3.87226869400031	10.27085514184986
H	4.08100280730035	3.89750270918367	8.53547433658576
H	4.81229807140177	5.12788511038277	9.55683960885506
C	7.86996665011825	-2.22607060031617	19.14902706501112
H	7.57636403522193	-1.60662511888980	19.99608092285113
H	8.36301337207927	-3.11673356481278	19.53808139058198
H	6.96334158080240	-2.54713547926449	18.63871101115978
C	6.74306732419188	3.48543826826024	8.50698600023891
H	7.08279177710699	4.51991861867577	8.54884793316211
H	6.24829293314459	3.33360276571078	7.54777404255143
H	7.62175875242810	2.84292692160252	8.52816221558820
C	10.04222172962193	1.09399247226452	11.48990263246700
H	9.27539340562226	0.36226179030526	11.23652089429729
H	10.95708018062894	0.54781052892020	11.71553858754878
H	10.23020869946423	1.70712958665946	10.60879722497349
C	5.32647618691252	1.71211349904318	9.55512571860895
H	6.17236695021658	1.02798623459855	9.61241229860033
H	4.81986095399681	1.53489156497392	8.60637694531666
H	4.63549752786776	1.46017749607539	10.35958074801670

List S4. [Re(O)(ap)(isq$^{•}$)], M06-L

C	8.355940	3.414075	4.815599
C	9.214895	4.474155	5.131494
C	10.563512	4.231405	5.345348
H	11.229735	5.055319	5.557564
C	11.050417	2.930676	5.268939
C	10.156907	1.896792	4.984455
H	10.532352	0.887435	4.938831

C	8.795374	2.094806	4.749761
C	9.222562	6.886388	5.528451
C	9.137600	8.032559	4.744703
H	8.599100	7.989534	3.806137
C	9.732489	9.210450	5.165531
H	9.661979	10.094208	4.546194
C	10.418643	9.257727	6.368942
H	10.883603	10.177579	6.695084
C	10.494890	8.119072	7.158783
H	11.011060	8.151267	8.108695
C	9.896718	6.940612	6.748566
H	9.929785	6.060876	7.376844
C	5.138944	7.279142	6.430913
C	4.704972	8.446150	7.055696
C	3.372940	8.460424	7.472482
H	3.009939	9.345988	7.969342
C	2.491326	7.396032	7.273350
C	2.956310	6.261296	6.619773
H	2.294956	5.427400	6.427062
C	4.277925	6.193136	6.208786
C	4.288180	3.850775	5.525193
C	4.128076	3.177299	4.317115
H	4.452451	3.659639	3.403623
C	3.564427	1.913051	4.291688
H	3.454211	1.398829	3.346355
C	3.141029	1.312701	5.467560
H	2.695873	0.327774	5.445804
C	3.299834	1.980754	6.674147
H	2.982691	1.514953	7.597245
C	3.873149	3.239789	6.707879
H	4.008182	3.758748	7.647754
C	7.830464	0.945501	4.476868
C	7.128441	1.146976	3.129682
H	6.575855	2.081812	3.097729
H	6.420663	0.336344	2.951829
H	7.847174	1.149284	2.310722
C	6.789734	0.894357	5.601271
H	7.268139	0.733771	6.567308
H	6.089842	0.075042	5.434349
H	6.210942	1.813921	5.657597
C	8.549350	-0.400090	4.436788
H	9.299835	-0.441635	3.647866
H	7.826785	-1.191817	4.243616
H	9.039759	-0.630523	5.382153
C	12.534686	2.679647	5.528805
C	13.379721	3.552335	4.594928
H	14.441621	3.377665	4.768540
H	13.194109	4.613742	4.749214
H	13.168918	3.328820	3.549895
C	12.851177	3.047914	6.982115

H	12.267376	2.443511	7.675726
H	12.625645	4.094480	7.184824
H	13.907085	2.886595	7.201248
C	12.923981	1.222882	5.298942
H	12.399890	0.546542	5.973070
H	13.991199	1.093084	5.473519
H	12.717561	0.905808	4.277029
C	5.642225	9.634276	7.241247
C	4.957639	10.790314	7.964665
H	4.091868	11.160659	7.416817
H	5.658130	11.617822	8.068646
H	4.630505	10.510778	8.966036
C	6.093829	10.136903	5.864115
H	6.592178	9.355057	5.295465
H	6.795369	10.964290	5.976888
H	5.244980	10.492771	5.281055
C	6.867440	9.218321	8.064411
H	6.569191	8.830774	9.038449
H	7.515285	10.079288	8.232171
H	7.454332	8.455696	7.558614
C	1.039904	7.426248	7.744909
C	0.108331	7.264770	6.539518
H	-0.934867	7.279804	6.856373
H	0.278692	6.323435	6.019128
H	0.251359	8.069096	5.818921
C	0.684955	8.727241	8.456679
H	1.296368	8.885202	9.344517
H	-0.356202	8.701955	8.775654
H	0.805488	9.592898	7.806171
C	0.805418	6.264260	8.716678
H	1.449526	6.346765	9.591491
H	1.006171	5.301253	8.248579
H	-0.229110	6.254200	9.060828
N	8.561798	5.705500	5.110233
N	4.889779	5.129303	5.536566
O	6.264845	6.359346	3.171971
O	7.083472	3.797231	4.560395
O	6.417105	7.090062	6.007325
Re	6.601006	5.672752	4.686343

List S5. [Re(O)(DMSO)(ap)(isq•)], M06-L

Re	7.41359375107283	4.71111357489964	15.78275512463755
O	8.45100211429784	3.67429956372078	14.49531324620193
O	6.13495784097873	3.04686761252592	15.96481384554393
O	8.21718676734129	6.18233870166796	16.11073760138271
S	5.89880438516272	5.38526572239379	17.69954171022317
N	6.35093186918571	5.00691368920329	14.09614519948068
N	8.34587314996087	3.44066276791768	17.13803025049861
C	5.01738024168749	5.45793299785615	14.13756869346319
C	3.93654381540375	4.58765464869223	13.97326378544789

H	4.13113865360847	3.54544356711960	13.75848717819509
C	7.93275039921961	3.51114393875314	13.25757754694265
C	2.63960534097550	5.05160277056409	14.10934632418226
H	1.81393683687158	4.36061791114763	14.00074669549486
C	6.69470332145529	1.95918751705155	16.44994894460318
C	7.93003912087783	2.15064473501445	17.13374547656890
C	6.12040235477313	4.10532563479347	11.78333754166789
H	5.22640416799672	4.68191647893132	11.59807428770479
C	8.09168989519452	-0.20528458968724	17.58145782297549
C	6.89510710497829	-0.37338557889948	16.85252807766759
H	6.53039880735034	-1.38299838943686	16.72480771166923
C	6.76316852968664	4.22598943430212	13.00921213888646
C	4.92767141195588	0.42085167027434	15.43119889742703
C	2.39593289833325	6.38996125879460	14.39552820220096
H	1.38144511557081	6.74851626535629	14.49990999019733
C	6.17137658145507	0.66215185770171	16.28239638209104
C	10.77568058546236	3.22285653070928	17.07280991284394
H	10.67404283504488	2.49378240897919	16.27924129527229
C	9.62826595495992	3.78638067835749	17.62861913942294
C	8.59718690736753	1.06523019957262	17.72461654426845
H	9.51138239149167	1.25139203717684	18.26653770902560
C	12.13486783704650	4.56575979132944	18.53082537048822
H	13.11048565043585	4.87237672442676	18.88168323082637
C	4.50581688843212	-1.04628012912635	15.44160790715791
H	5.27142208921045	-1.69821983136217	15.02232296005602
H	3.60937374204700	-1.16905505287818	14.83559229223843
H	4.27549322004321	-1.39819385170726	16.44728508912870
C	8.79429480308895	-1.43804021971757	18.13781630867418
C	9.73800562461605	4.74430607680301	18.63146204506101
H	8.83315785019733	5.16487206396704	19.05012550602402
C	3.46446701244818	7.26442574404660	14.53712619686355
H	3.28315582611080	8.31153119099035	14.74134408282930
C	5.92021597957082	2.99812928366498	9.49031472713741
C	3.74480392643480	1.24706547891403	15.94924231674126
H	3.52835079598003	1.01408785448784	16.99208889753884
H	2.84922184527176	1.02484579304723	15.36727809904967
H	3.94079363257969	2.31226104181727	15.86843715350706
C	6.62372599048709	3.23274158845001	10.82455590565939
C	10.99042003761519	5.12431253994299	19.08182085619606
H	11.07437197858349	5.86296317296708	19.86718664100446
C	8.47995368418560	2.63596666997619	12.31965619379638
C	4.76608239631587	6.80360564142196	14.40650170333105
H	5.61086481647985	7.47426450629363	14.50230510888702
C	12.02342025877716	3.61720258677502	17.52449316631025
H	12.91175107146057	3.18651431286251	17.08252416096499
C	7.78801182122064	2.51860813613034	11.11635310650278
H	8.16423801090865	1.83195133629350	10.37111715268191
C	5.23575992887686	0.81565214452232	13.97930185618159
H	5.56576799249420	1.85005131368685	13.89742268578908
H	4.34864456360993	0.69298645885047	13.35503574475570

H	6.02326899160122	0.18827893876305	13.56179490023421
C	9.16665230151136	-2.36617084320509	16.97561388661940
H	8.29007969404389	-2.68752224404895	16.41500572352163
H	9.66752658630898	-3.26085410858451	17.34627489237208
H	9.83897387134363	-1.86816383533287	16.27804914935965
C	10.88977686321977	2.79982600762198	12.96607428533828
H	11.11707273982283	3.44141050404353	12.11514187843441
H	11.79383034442336	2.23972443710724	13.21001072507496
H	10.64840064813015	3.43804261908130	13.81152050833405
C	9.74147440087719	1.83828170697525	12.63630113827953
C	10.07085191237758	-1.07912878478093	18.89048407468771
H	10.79451089527753	-0.57958659972799	18.24609231447275
H	10.54304153612493	-1.98327746879678	19.27248476030084
H	9.87335754462229	-0.42735495230038	19.74102679770152
C	9.48361071243478	0.91148305340097	13.83270218780846
H	9.15626014827664	1.46235875466550	14.71195242882315
H	10.39493179181191	0.36979524352548	14.09373535102115
H	8.71623078770032	0.17183729256407	13.60273425467894
C	4.69786036048139	3.89337755970229	9.31813092116785
H	3.94819348368595	3.71399082505042	10.08887788081004
H	4.22720909917656	3.70177304206181	8.35438447240271
H	4.96247712340762	4.95013343787980	9.34905962145971
C	7.85518615154505	-2.17942995784471	19.09398240499982
H	7.57518641713886	-1.55106565211974	19.93865399269968
H	8.33679275755115	-3.07521126935924	19.48673911167065
H	6.93785722679117	-2.49206731818597	18.59761220443252
C	6.88501170980874	3.27597435589955	8.33438219170301
H	7.23071497753875	4.30898122734262	8.35266496540008
H	6.39543850398942	3.10321098920369	7.37527454042336
H	7.76373759933812	2.63460973566475	8.37478321073132
C	10.17552719757874	0.97165641125802	11.45803542008469
H	9.41302838142053	0.24141163559638	11.18821210167592
H	11.07708127987611	0.41951139448895	11.72139277902632
H	10.40121128780926	1.56778065146292	10.57402433433774
C	5.46185137941315	1.53663144594737	9.42599945098546
H	6.30388161282338	0.84993939330161	9.50224507033271
H	4.94931904005102	1.33131361471945	8.48531699396703
H	4.77592659346088	1.30709402137940	10.24153755819179
O	6.28763432228465	4.85624635200315	19.01089754116270
C	4.17898748555489	4.99748043780767	17.44231741864666
H	4.11870206941038	3.92838424342016	17.26454901644770
H	3.79119638953861	5.54495920868516	16.58805534337694
H	3.65298192531594	5.26458819912334	18.35517482765290
C	5.81636697264908	7.16508330431884	17.81786998885282
H	5.21122518686483	7.41866671882699	18.68410418228920
H	5.39127775461289	7.57248282069476	16.90508713051612
H	6.83670151672019	7.51515546836849	17.94017232590884

List S6. [(ap)(isq•)Re(O)(μ-O)Re(O)(ap)(isq•)], M06-L

C	5.026065	-2.245543	7.517899

C	4.532328	-2.166290	6.220805
H	5.032870	-1.532130	5.501497
C	3.409702	-2.894977	5.870268
H	3.022423	-2.823000	4.863631
C	2.790370	-3.717755	6.800392
H	1.920683	-4.294894	6.518445
C	3.293897	-3.804425	8.089886
H	2.825690	-4.454830	8.816475
C	4.405523	-3.063821	8.455342
H	4.812374	-3.127256	9.455785
C	7.318764	0.005726	9.215432
C	6.083728	-0.617755	8.921290
C	4.934437	-0.305110	9.657144
H	3.990786	-0.744200	9.375473
C	5.028686	0.566222	10.719710
C	6.290536	1.111453	11.038161
H	6.347239	1.771677	11.892951
C	7.446513	0.874304	10.318049
C	3.822538	0.954218	11.567450
C	2.540561	0.295769	11.068557
H	2.599295	-0.791616	11.115136
H	2.314319	0.573894	10.039479
H	1.697141	0.603893	11.684770
C	3.638194	2.474458	11.522105
H	3.465454	2.816822	10.502179
H	4.512192	3.001134	11.902121
H	2.782567	2.776774	12.126223
C	4.061545	0.513147	13.014745
H	4.191797	-0.567268	13.076645
H	3.213710	0.786180	13.643555
H	4.950334	0.977425	13.439944
C	8.787121	1.517747	10.646185
C	8.697544	2.411990	11.878078
H	8.410863	1.854000	12.769830
H	7.982136	3.223186	11.742125
H	9.669305	2.863393	12.072436
C	9.846565	0.438580	10.907558
H	10.003611	-0.196831	10.037491
H	9.567676	-0.195876	11.750534
H	10.800845	0.906855	11.153336
C	9.221386	2.374673	9.453932
H	9.309463	1.775564	8.551523
H	10.189970	2.837432	9.647935
H	8.502956	3.170454	9.258924
C	7.920852	-3.585003	9.448979
C	7.713617	-2.971327	10.687188
H	8.239332	-2.051405	10.912946
C	6.843991	-3.534105	11.605615
H	6.679626	-3.040643	12.554645
C	6.185893	-4.721671	11.310369

H	5.511570	-5.162628	12.031456
C	6.395092	-5.335701	10.084419
H	5.879648	-6.255673	9.843359
C	7.248382	-4.766580	9.150828
H	7.398828	-5.224536	8.182761
C	10.086705	-2.744179	8.779190
C	10.801635	-3.164284	9.891366
H	10.318741	-3.789901	10.629846
C	12.119752	-2.749977	10.059390
C	12.698322	-1.930794	9.088721
H	13.710480	-1.590363	9.236428
C	12.024843	-1.510287	7.938259
C	10.716141	-1.956889	7.802904
C	12.870861	-3.191536	11.313339
C	12.083241	-2.756945	12.555490
H	11.087359	-3.197155	12.580450
H	11.966619	-1.674123	12.584700
H	12.600718	-3.063835	13.464820
C	12.998791	-4.717983	11.310276
H	12.022914	-5.199989	11.275737
H	13.510519	-5.063387	12.209132
H	13.566875	-5.061830	10.446558
C	14.267830	-2.587154	11.401636
H	14.236195	-1.498112	11.429280
H	14.895078	-2.883760	10.561579
H	14.760247	-2.924977	12.312583
C	12.636746	-0.556244	6.918636
C	11.801109	0.731102	6.881092
H	10.761979	0.531394	6.618579
H	12.208627	1.431343	6.147488
H	11.808785	1.222100	7.855574
C	12.655110	-1.198608	5.527865
H	13.206565	-2.138570	5.534648
H	13.141763	-0.528846	4.816254
H	11.649454	-1.396889	5.162622
C	14.068916	-0.180216	7.285446
H	14.727552	-1.048330	7.309833
H	14.125346	0.314662	8.254557
H	14.465088	0.512002	6.543181
C	9.915890	-1.046803	2.426647
C	9.812692	-2.330508	2.951866
H	9.491300	-2.460863	3.977182
C	10.124502	-3.421445	2.161062
H	10.049960	-4.416719	2.576190
C	10.518378	-3.241000	0.842569
H	10.751860	-4.097089	0.224887
C	10.606288	-1.960217	0.317669
H	10.898565	-1.811001	-0.713177
C	10.313665	-0.860732	1.107114
H	10.371345	0.142046	0.704705

C	10.515127	1.053426	3.424484
C	11.824685	1.070532	2.933506
H	12.193943	0.216882	2.388661
C	12.621283	2.169498	3.167173
C	12.083193	3.250251	3.897481
H	12.719589	4.109755	4.057527
C	10.813122	3.268720	4.442422
C	10.024763	2.122283	4.211269
C	14.060935	2.256228	2.675549
C	14.480354	1.007169	1.907912
H	15.514311	1.101735	1.579127
H	13.866727	0.851213	1.020686
H	14.414148	0.110397	2.523675
C	14.993321	2.419598	3.880356
H	16.031522	2.500794	3.557566
H	14.918372	1.562968	4.550897
H	14.757495	3.311063	4.460209
C	14.209435	3.467995	1.749975
H	15.236578	3.556217	1.395792
H	13.955263	4.398591	2.255170
H	13.560407	3.376677	0.879388
C	10.270993	4.428864	5.266096
C	9.908943	3.916152	6.662159
H	9.141815	3.148209	6.610617
H	9.532093	4.731179	7.281603
H	10.779869	3.490960	7.161557
C	9.017197	5.009947	4.600719
H	9.244768	5.427021	3.617856
H	8.611472	5.816544	5.212697
H	8.237036	4.260248	4.481180
C	11.299495	5.544584	5.416524
H	11.593819	5.966233	4.455254
H	12.200699	5.201539	5.924943
H	10.876008	6.352577	6.011239
C	7.803713	1.826565	1.345100
C	8.781847	2.808631	1.166758
H	8.982383	3.502652	1.974008
C	9.481734	2.884000	-0.025185
H	10.244482	3.641582	-0.147568
C	9.211451	1.990570	-1.054486
H	9.757355	2.053468	-1.985697
C	8.242576	1.013404	-0.878204
H	8.035480	0.306324	-1.670123
C	7.547892	0.922328	0.317965
H	6.806320	0.150906	0.474021
C	6.390248	2.758794	3.079167
C	6.108232	3.975327	2.463490
H	6.458350	4.147203	1.457383
C	5.422326	4.952731	3.170063
C	5.005971	4.678062	4.479635

H	4.491584	5.457663	5.023723
C	5.236367	3.463700	5.121716
C	5.925749	2.510106	4.375830
C	4.840906	3.182572	6.567092
C	3.903456	1.971590	6.632412
H	2.980493	2.159731	6.084642
H	3.640722	1.755365	7.669610
H	4.367977	1.080476	6.218145
C	6.105926	2.897304	7.391603
H	6.643068	2.023302	7.023847
H	5.838762	2.715155	8.435416
H	6.787772	3.748606	7.360395
C	4.125294	4.372831	7.198349
H	3.204162	4.622916	6.672089
H	4.753649	5.263135	7.219820
H	3.862220	4.135704	8.228461
C	5.143300	6.331139	2.574066
C	3.632734	6.575332	2.532928
H	3.189644	6.523641	3.526432
H	3.132969	5.834137	1.910387
H	3.414729	7.561600	2.122132
C	5.803873	7.402741	3.448471
H	5.425745	7.381745	4.469336
H	5.613090	8.397763	3.045198
H	6.883329	7.261203	3.494100
C	5.694634	6.466204	1.158247
H	6.775259	6.322561	1.128784
H	5.485089	7.462367	0.770921
H	5.243533	5.747281	0.475096
N	6.179960	-1.492675	7.868832
N	8.765303	-2.991330	8.482125
N	9.581997	0.062401	3.251438
N	7.113161	1.703660	2.572097
O	8.291751	-0.317382	8.413444
O	9.915379	-1.644042	6.764819
O	8.814647	1.955180	4.659971
O	6.246776	1.287935	4.838728
O	7.906395	-0.465721	5.674901
O	7.294588	-3.076849	6.111404
O	7.220840	-1.039491	3.101079
Re	7.993442	-1.801887	7.004259
Re	7.705639	0.337159	3.983377

List S7. $[Re(O)_2(ap)_2]^{-}$, M06-L

Re	7.226369	4.655746	16.033864
O	8.348137	3.694922	14.556516
O	6.175148	2.847378	15.943749
O	8.497688	5.789608	16.291428
O	5.902495	5.409256	16.839055
N	6.282428	5.052944	14.273272

N	8.182377	3.398796	17.311027
C	5.078953	5.794279	14.179066
C	3.888161	5.275651	14.677701
H	3.912981	4.310629	15.166234
C	7.806113	3.549781	13.368126
C	2.713455	5.999114	14.556113
H	1.792027	5.593145	14.953110
C	6.734056	1.784113	16.476214
C	7.870905	2.048639	17.260756
C	5.953319	4.243913	11.950140
H	5.056819	4.832747	11.824526
C	8.171643	-0.300419	17.677053
C	7.022475	-0.538944	16.918861
H	6.703770	-1.565752	16.794902
C	6.643189	4.312330	13.157367
C	5.009487	0.190966	15.506497
C	2.712531	7.237468	13.929254
H	1.792963	7.800364	13.838093
C	6.276884	0.468042	16.309478
C	10.572789	3.884182	17.329120
H	10.581046	3.736636	16.257432
C	9.364907	3.755343	18.007505
C	8.585625	1.013688	17.856871
H	9.465582	1.251475	18.436341
C	11.684973	4.415179	19.396044
H	12.586240	4.676518	19.934779
C	4.684328	-1.299186	15.449565
H	5.474610	-1.868205	14.958764
H	3.766511	-1.452921	14.881402
H	4.532392	-1.723559	16.442560
C	8.935213	-1.485311	18.262027
C	9.325020	3.955575	19.382186
H	8.377989	3.853590	19.895258
C	3.898765	7.752562	13.426776
H	3.908107	8.719416	12.940176
C	5.675423	3.248348	9.612298
C	3.830058	0.908483	16.175366
H	3.668287	0.529486	17.184902
H	2.912131	0.750562	15.605624
H	4.012760	1.978041	16.242492
C	6.412862	3.406289	10.940293
C	10.481507	4.284999	20.072820
H	10.440155	4.445147	21.142530
C	8.299556	2.710017	12.358522
C	5.077990	7.034494	13.550953
H	6.011374	7.422213	13.164728
C	11.724787	4.215656	18.022810
H	12.659333	4.321448	17.487225
C	7.574464	2.663483	11.169355
H	7.921381	2.010237	10.379471

C	5.158049	0.697282	14.066869
H	5.353108	1.766572	14.034122
H	4.242994	0.500060	13.504931
H	5.977120	0.193183	13.553311
C	9.368422	-2.421681	17.127788
H	8.512697	-2.805450	16.574233
H	9.923638	-3.277090	17.518288
H	10.007162	-1.897616	16.417100
C	10.732125	2.892005	12.851145
H	10.898264	3.544257	11.993261
H	11.659776	2.350084	13.045996
H	10.513145	3.518565	13.712272
C	9.581215	1.913892	12.581185
C	10.188046	-1.054636	19.018635
H	10.883532	-0.517575	18.374024
H	10.707307	-1.930777	19.408974
H	9.952490	-0.406894	19.862929
C	9.432787	0.968892	13.780314
H	9.182465	1.508590	14.690198
H	10.368407	0.431875	13.948870
H	8.651617	0.227934	13.607726
C	4.475336	4.184105	9.503787
H	3.735485	3.990594	10.279798
H	3.983692	4.051735	8.539084
H	4.771839	5.229901	9.584018
C	8.032140	-2.257759	19.229410
H	7.722789	-1.626547	20.062399
H	8.549255	-3.128060	19.639006
H	7.128497	-2.610112	18.734142
C	6.620122	3.556853	8.446386
H	6.991743	4.579544	8.507066
H	6.107304	3.440674	7.489238
H	7.485185	2.895396	8.439333
C	9.950751	1.070516	11.364166
H	9.181223	0.332623	11.135070
H	10.876397	0.528665	11.561397
H	10.107522	1.681043	10.474340
C	5.174944	1.806046	9.480310
H	5.999532	1.095550	9.525202
H	4.652942	1.656044	8.532592
H	4.487167	1.559860	10.288791

List S8. $[Re(O)(ap)_2]^{-}$, M06-L

C	8.340644	3.375446	4.945126
C	9.210042	4.457675	5.149833
C	10.571561	4.228420	5.271542
H	11.243347	5.069015	5.385453
C	11.080082	2.930943	5.236347
C	10.190526	1.875511	5.069902
H	10.570777	0.866135	5.049095

C	8.810291	2.062718	4.915685
C	9.207622	6.833533	5.633999
C	9.046829	8.054184	4.974526
H	8.432599	8.074619	4.082483
C	9.641771	9.206477	5.457294
H	9.493979	10.141513	4.931808
C	10.417536	9.171758	6.607694
H	10.881402	10.073712	6.984166
C	10.578642	7.965439	7.276041
H	11.164689	7.925287	8.185664
C	9.981825	6.810912	6.800385
H	10.096742	5.877796	7.335695
C	5.078983	7.330295	6.292695
C	4.671390	8.462993	6.996873
C	3.350661	8.483492	7.463677
H	3.018747	9.350124	8.014215
C	2.458118	7.440000	7.244053
C	2.902403	6.322894	6.537850
H	2.224532	5.502451	6.341773
C	4.208710	6.250160	6.080104
C	4.192092	3.945810	5.320405
C	4.183117	3.220824	4.126774
H	4.626875	3.676821	3.250096
C	3.643092	1.948134	4.071955
H	3.660410	1.403336	3.136457
C	3.089230	1.367469	5.204151
H	2.667553	0.372189	5.159688
C	3.095519	2.077170	6.397570
H	2.685508	1.631543	7.295354
C	3.639854	3.348379	6.459262
H	3.658951	3.888864	7.396235
C	7.848035	0.895315	4.708335
C	7.174329	1.026788	3.337043
H	6.635483	1.967360	3.252535
H	6.462326	0.213398	3.181382
H	7.914975	0.983959	2.537731
C	6.779776	0.891406	5.807685
H	7.238247	0.774717	6.790373
H	6.084119	0.062809	5.661194
H	6.205013	1.813492	5.807215
C	8.563665	-0.452727	4.753428
H	9.316437	-0.549157	3.970473
H	7.838201	-1.254537	4.612315
H	9.055264	-0.618756	5.712619
C	12.587428	2.734698	5.379589
C	13.308888	3.473816	4.246907
H	14.391171	3.354820	4.328899
H	13.089105	4.540126	4.262550
H	12.998763	3.090768	3.274973
C	13.052685	3.307391	6.723127

H	12.571924	2.789482	7.553031
H	12.807278	4.365510	6.813211
H	14.133363	3.203650	6.840015
C	12.990271	1.264825	5.321189
H	12.523855	0.687504	6.119240
H	14.071094	1.166424	5.428617
H	12.708231	0.808910	4.372425
C	5.640628	9.616682	7.239879
C	4.998198	10.754684	8.029501
H	4.148727	11.192260	7.503969
H	5.732453	11.545649	8.185155
H	4.652665	10.428910	9.011125
C	6.112622	10.187755	5.897418
H	6.623299	9.431235	5.307570
H	6.804857	11.016579	6.059613
H	5.267714	10.562995	5.319125
C	6.848966	9.115904	8.040178
H	6.533055	8.730967	9.010596
H	7.558260	9.927126	8.215409
H	7.373187	8.322264	7.513804
C	1.019631	7.452930	7.756208
C	0.049023	7.317749	6.578242
H	-0.987022	7.319125	6.923418
H	0.212832	6.391875	6.029043
H	0.171124	8.140438	5.874113
C	0.679622	8.735010	8.509104
H	1.318520	8.870333	9.381410
H	-0.353807	8.702756	8.856266
H	0.788608	9.615405	7.876151
C	0.811185	6.269605	8.708152
H	1.473531	6.343513	9.570497
H	1.020698	5.320134	8.216286
H	-0.217410	6.236820	9.073504
N	8.555951	5.692842	5.141850
N	4.795757	5.212234	5.351816
O	6.467171	6.061806	2.938086
O	7.040998	3.695774	4.789006
O	6.318061	7.185805	5.783326
Re	6.614264	5.629852	4.570331

List S9. O_2, M06-L

O	0.000000	0.000000	-0.102633
O	0.000000	0.000000	1.102633

List S10. $[Re(O)_2(ap)(isq^{\bullet})]$, CPCM (benzene), M06-L

Re	7.27554596704794	4.72744790165886	16.04309763576752
O	8.38715662469444	3.77318974405684	14.55775856230660
O	6.21862376853901	2.92723908596693	15.93440599346529
O	8.54134160202113	5.86718232196873	16.27022647496699

O	5.97035839232915	5.46003649866969	16.88751192760720
N	6.25121559948176	5.03432902001778	14.28278845253732
N	8.28676964477594	3.40583302031928	17.24353968693611
C	4.97179889997144	5.64987680315308	14.28986651829489
C	3.82518807422563	4.88378468210599	14.08842126287473
H	3.92061446144251	3.81905270624766	13.91823713391073
C	7.83783282880293	3.58359210769922	13.39576346983460
C	2.57919404470931	5.48391587268465	14.13779732747230
H	1.69210985575553	4.88216898698202	13.99450353580215
C	6.75964525659320	1.85471592834695	16.42698471858119
C	7.92486184521107	2.08544744868275	17.20564068293256
C	5.96807372809312	4.23073944216285	11.95886997173086
H	5.07295051650230	4.81150646462943	11.80401896802528
C	8.09477516909252	-0.26168403416191	17.66824020624683
C	6.94739170087780	-0.46490340427421	16.86607519818487
H	6.59835222195887	-1.48089661038948	16.74579820377084
C	6.63216558213222	4.30629804511164	13.18693691023591
C	4.99869553909761	0.32266362596186	15.40402022453170
C	2.46739497446349	6.84549038203526	14.38433558467179
H	1.49261087111408	7.31146340129805	14.42345616119199
C	6.24971548740803	0.55008518507753	16.24309460913389
C	10.71544494563557	3.22202778475104	17.35577753990376
H	10.68163115271065	2.51540927204951	16.53672308436312
C	9.52606834637515	3.76399055851217	17.83919552013038
C	8.56700593324486	1.02242891990475	17.84813129351526
H	9.42979703124103	1.22139614599637	18.46360752647725
C	11.95145620407006	4.52843191994504	18.95095057990803
H	12.89608686424195	4.82915941809222	19.38232225233151
C	4.62676648784708	-1.15478935987268	15.33448613174447
H	5.41541412630618	-1.75222572198131	14.87712606452465
H	3.73015055739361	-1.27384709942461	14.72811289547180
H	4.41622374705859	-1.57020734209183	16.31974853172927
C	8.77171516290691	-1.47543291474610	18.29162467979926
C	9.55389239411048	4.69439868907116	18.87088737794990
H	8.62341071665143	5.11647243061688	19.22613530805079
C	3.61001780113082	7.60372196697235	14.59358519399780
H	3.52957177405273	8.66307431465224	14.79461069478547
C	5.73797377182086	3.19383123734446	9.64660155977618
C	3.82236043521038	1.08435795876910	16.03074660988942
H	3.62768456746791	0.73356062643447	17.04373937886081
H	2.91797217255699	0.92410607029265	15.44300828797231
H	4.01374246876859	2.15352190176666	16.07302716025155
C	6.45479363903411	3.39431930249838	10.97514052601152
C	10.76558246043386	5.06912537753703	19.42703779217401
H	10.78280300691825	5.78915492946305	20.23344234513553
C	8.36560469289286	2.73894700003266	12.39365655538005
C	4.86044397668457	7.00999929902417	14.55323276487483
H	5.75757069587944	7.58839435996151	14.72860372554520
C	11.92245144206486	3.60818448793035	17.91216325591124
H	12.84446864514836	3.19597168327413	17.52537192940418

C	7.64237448366758	2.66638756848240	11.22099482564851
H	7.99789119954825	2.01609686918496	10.43422854252331
C	5.22744771043687	0.82272425731794	13.97140617082679
H	5.49881384881765	1.87661499382079	13.94855036903656
H	4.31810702388781	0.69650917581203	13.38185335385519
H	6.02022741905934	0.25923516472152	13.47849868178040
C	9.20296639357289	-2.43286072241536	17.17346776042229
H	8.35761090662515	-2.77042593846489	16.57593028098513
H	9.68374975383241	-3.31599527893696	17.59446043380060
H	9.91371348134673	-1.95219122191550	16.50172791212043
C	10.79719622976484	2.95280061909470	12.90136729752609
H	10.95582041948927	3.60505575629358	12.04308256256577
H	11.72623636709424	2.41230742697556	13.08448228348934
H	10.59283354624976	3.57803110914394	13.76626617251025
C	9.65443669383936	1.96398161859140	12.63476051392823
C	10.00883765681437	-1.09725229519753	19.09915457376158
H	10.75794191355292	-0.59974226598262	18.48295101843150
H	10.46678801858602	-1.99505992643249	19.51192293796120
H	9.76791237422591	-0.43845278385564	19.93296129497757
C	9.48944297572284	1.02865666483901	13.84037008985674
H	9.21142216604205	1.57246230008810	14.74087701985081
H	10.42636015521893	0.50614980209573	14.03737437721644
H	8.72444399674923	0.27485944597484	13.65192062749736
C	4.49920734830107	4.07417249202111	9.52371258146692
H	3.76621448434461	3.85827995564959	10.30090932622693
H	4.01742844156026	3.90340305929420	8.56196123289767
H	4.74895124346665	5.13325584255175	9.58265069738497
C	7.78164625235151	-2.19124292014426	19.21646920696270
H	7.46381086405831	-1.54155214083160	20.03135778301171
H	8.24219087360373	-3.07742535433120	19.65359792814054
H	6.88956762025620	-2.51312599564784	18.68153843018349
C	6.68513092942359	3.52620949157162	8.48915695475995
H	7.01050728493149	4.56497317433715	8.53358688865422
H	6.18173119245733	3.37347354581438	7.53441175683318
H	7.57474488014350	2.89846840579130	8.49386982733064
C	10.03314046784536	1.11109434395601	11.42812636261943
H	9.26835391108763	0.36873910030337	11.19973792461701
H	10.95813167129223	0.57536194207904	11.63735128030726
H	10.19668832124604	1.71533461873571	10.53607434304026
C	5.30651605204506	1.72559118812690	9.54096880661412
H	6.16078086392582	1.05044130595225	9.57478195051591
H	4.78316329719194	1.55024579383315	8.60089096596882
H	4.63579539012241	1.45508996891408	10.35635566500556

List S11. $[Re(O)_2(ap)_2]^{-}$, CPCM (benzene), M06-L

Re	7.23520935957955	4.69281979862670	16.03905123633525
O	8.36258886346837	3.74737479915157	14.57354321878872
O	6.19148681217413	2.89378769742383	15.92224302835666
O	8.49742115152098	5.84908362592805	16.28201060487662
O	5.90709838555268	5.42084543520267	16.87232583510367

N	6.26344618528365	5.06561257053078	14.28074090455558
N	8.21512647534026	3.42394733288111	17.29495305700790
C	4.97261648733424	5.64612967449679	14.28107991840776
C	3.83335351332026	4.86569791827906	14.08290438584353
H	3.94642717499275	3.80104979735066	13.92034479523278
C	7.82405854378440	3.58583064329646	13.38483750034188
C	2.57655259423121	5.44512177380176	14.12164845649977
H	1.70005300695432	4.82649178071324	13.97964639337063
C	6.73108616213014	1.82598944110487	16.46638225725497
C	7.87088659588490	2.07556389044691	17.24898999164227
C	5.98892525228452	4.25750210336332	11.94186802253000
H	5.09668842349851	4.84576452447995	11.79094341443509
C	8.07561217100640	-0.27152125332282	17.74275994862197
C	6.94334306111882	-0.49542683035940	16.95178242500799
H	6.60088930442526	-1.51577778513979	16.84039671211138
C	6.64262249324362	4.31649990791439	13.17065471535109
C	4.99266485434245	0.26244432086732	15.46726221108696
C	2.43797573461624	6.80687630729576	14.35764313861574
H	1.45495664887919	7.25698374528114	14.38971692284828
C	6.24033600839092	0.51931937025076	16.30768949585689
C	10.64734180627433	3.20526899221202	17.22692278002307
H	10.54275266361263	2.50894121081771	16.40438695030575
C	9.49863780350795	3.75575618946893	17.79575935074102
C	8.52589161199743	1.03275726951088	17.90016570140576
H	9.38578929740161	1.25548847755538	18.51371989673214
C	12.02208309932677	4.47385987239516	18.73938067450572
H	13.00073313940543	4.75524460065297	19.10410694806775
C	4.61988141707457	-1.21705264732096	15.43619206473541
H	5.40900056744894	-1.82588056017525	14.99431742028865
H	3.72194128294105	-1.35411270223167	14.83393043972439
H	4.41305562452349	-1.60762669603965	16.43284558895332
C	8.77659912310826	-1.46220724711353	18.39237695371172
C	9.62537369704360	4.67125789020120	18.83514701649683
H	8.72947506515054	5.10757678047503	19.25647913550900
C	3.56930196716297	7.58314687108955	14.56328861986920
H	3.47196803376094	8.64325328773872	14.75678743814756
C	5.78865464353994	3.30432966982167	9.57913300502632
C	3.80936942860819	1.03710347293342	16.06305376973709
H	3.59999843728406	0.70376432556106	17.07973668079163
H	2.90993496684670	0.87509521472347	15.46605835936545
H	4.01133525097512	2.10495880462355	16.09224946268994
C	6.48079228302255	3.43785558923624	10.93381686330545
C	10.88078915724979	5.02644805672713	19.30294154497121
H	10.96773599470078	5.74258473816124	20.10937244837720
C	8.34890245884930	2.75478743235102	12.38154787749379
C	4.82926467234131	7.00801007527122	14.52655042936445
H	5.71716327854053	7.60112867306890	14.70148522305115
C	11.89898679278852	3.56417091292311	17.69679125825520
H	12.78303634997032	3.14002259574970	17.23857739931386
C	7.64947096903712	2.70796253590159	11.17912151959262

H	8.02294383574624	2.07168484009784	10.38783832115349
C	5.22391338301211	0.72218388288856	14.02229582492332
H	5.48085592770925	1.77852609725077	13.97435841614624
H	4.32360382919988	0.56051627680869	13.42602392517198
H	6.03448705911903	0.16006075265455	13.55728160821062
C	9.23023577407674	-2.43949253594550	17.30151940776424
H	8.38995493695389	-2.79873027008353	16.70891495236265
H	9.72560122785201	-3.30824072984567	17.73875585409747
H	9.93211958206949	-1.95996054534987	16.61929917003048
C	10.77441554861000	2.87885330826290	12.98587860241292
H	10.99334456809333	3.54901056144042	12.15405291049495
H	11.67751132547788	2.30329121082723	13.19755787923539
H	10.54588333743846	3.48656866352667	13.85747039429437
C	9.61256230090295	1.93931065556970	12.64087624840336
C	10.00682303130129	-1.04472101096501	19.19236269730129
H	10.74363625322495	-0.54151262383440	18.56580794416899
H	10.48528122320632	-1.92386620780209	19.62440891401816
H	9.75180019522619	-0.37208721662364	20.01104371125694
C	9.37555951961935	0.97110619706967	13.80819587689628
H	9.05626121011509	1.49684435553037	14.70568947460293
H	10.29113318643204	0.42345559911546	14.04053944434105
H	8.60626846849776	0.23926920027283	13.55895229027208
C	4.49296165844346	4.10709874314101	9.51419387988261
H	3.78081273292196	3.78312899961541	10.27384521602974
H	4.02075461590152	3.97674218078037	8.54029651960575
H	4.66695558620052	5.17344982584173	9.65551577507264
C	7.80723294266027	-2.18123104042539	19.33580247981209
H	7.47424519105337	-1.51823898656260	20.13440355727535
H	8.28248921548001	-3.04956106043506	19.79578691294557
H	6.92104397892332	-2.52881342673181	18.80665855639493
C	6.72523711542059	3.80447136646587	8.47399404404389
H	6.97925200030863	4.85373780635463	8.62298336065971
H	6.25720721758316	3.70628814210196	7.49281296717562
H	7.65571738379343	3.23812536904337	8.45669918244480
C	10.01962025073774	1.11244709871081	11.42420947695643
H	9.24485753509015	0.39985442146598	11.14032806295066
H	10.92065139443349	0.54344597667583	11.65424937083572
H	10.23547230426683	1.73790463914949	10.55756621827720
C	5.44880928616548	1.83357976975756	9.31311037920324
H	6.33992809476335	1.20758751646572	9.30143205385264
H	4.95450935972898	1.72214791275335	8.34647615350041
H	4.78012426538511	1.44059800881039	10.07915555488252

List S12. •CPh_3, CPCM (benzene), M06-L

C	-0.00193891910451	-0.10634361779316	-0.07906211070209
C	0.84540852780157	1.07358285804392	-0.05763786787553
C	0.59455621897394	-1.42880719287007	-0.16024632208664
C	-1.44565755946870	0.03376717997113	-0.00423162977894
C	0.42001174752964	2.29346206701445	-0.61652128140988

C	2.12487449967564	1.04657929276640	0.52757796730819
C	2.92695306030526	2.17189094211036	0.55515856743864
C	1.22862396407073	3.41410158990125	-0.59562808235281
C	2.48677263833561	3.36348611537445	-0.00812469464457
H	2.47217770750036	0.12997494180378	0.98498889900243
H	3.89962252697217	2.12282061051451	1.02619481703120
H	0.87878860821173	4.33327507650015	-1.04641959212569
H	3.11627642170977	4.24250414100892	0.00956371311771
H	-0.55133142691637	2.34181610227938	-1.09030457297491
C	-0.00447620230670	-2.54076523432382	0.46102832553590
C	1.79535954875992	-1.65381189557190	-0.85934939539990
C	2.35729365832101	-2.91374588762531	-0.93495459990822
C	0.56573281562808	-3.79732899476971	0.39194467557103
C	1.74931174874901	-3.99514449121656	-0.30842233198754
H	-0.91763546154882	-2.39676367063274	1.02282781081905
H	0.08875728127008	-4.62826943728894	0.89448988943414
H	2.27039653274179	-0.82487506002645	-1.36676826767399
H	3.27308035333190	-3.05734139266457	-1.49280981209131
H	2.19280999303278	-4.97964507813863	-0.36686010809302
C	-2.30542463629298	-0.87797124038558	-0.64504663740832
C	-2.04496852528395	1.08508089361877	0.71517273072701
C	-3.41872975589374	1.21058528896910	0.79334436135431
C	-3.67862869636811	-0.74339232464447	-0.57292798960674
C	-4.24646650968876	0.29930589678308	0.14931598392962
H	-1.87573202431076	-1.68309030946281	-1.22547052493859
H	-4.31268521020247	-1.45161890609464	-1.08938648851008
H	-3.84989717688003	2.01939951628466	1.36797889376387
H	-5.32137101589004	0.40031511308327	0.20946762820095
H	-1.41221473276505	1.78999710748183	1.23729804633472

List S13. $[CPh3]^+$, CPCM (benzene), M06-L

C	-0.00470213595756	-0.10658220014604	-0.07572261019518
C	0.83460291829150	1.06056603104834	-0.05602531361722
C	0.58657640557196	-1.41473974983175	-0.15920594177152
C	-1.43367754140900	0.03226117530030	-0.00330812710839
C	0.39023701832411	2.27919843409604	-0.60956957333802
C	2.11971133840688	1.01083098505494	0.52177596910926
C	2.91618113781501	2.13507170419964	0.55356104440342
C	1.20488941504180	3.39052265128256	-0.59643033168085
C	2.46415924711879	3.32301791833838	-0.01048806577968
H	2.45212777409244	0.09684819725404	0.99268898050318
H	3.88749535362469	2.09377453256160	1.02471821526065
H	0.86639974227477	4.31135239671040	-1.04888228916173
H	3.09562353500422	4.20053520463842	0.00560443238202
H	-0.57346671935170	2.31818364073717	-1.09707709737646
C	-0.02959086690456	-2.52219240751004	0.45927131113622
C	1.79693148196171	-1.61782195633036	-0.85403511524560
C	2.35331608058938	-2.87550675189590	-0.93462659617282
C	0.54920329215257	-3.77061109686048	0.39583927004413
C	1.73581230198731	-3.95144944060800	-0.30569219778313

H	-0.93141205087185	-2.37088959959854	1.03525003627313
H	0.08404298613570	-4.60575256332476	0.89930048554292
H	2.25534192535912	-0.78783192808362	-1.37260410273975
H	3.26547654620551	-3.02615782909192	-1.49364870346167
H	2.18163702133339	-4.93479583450090	-0.36429844383694
C	-2.28109209437931	-0.89681661990760	-0.64143583291848
C	-2.01847590756784	1.09867596835554	0.71093567656502
C	-3.38861203655225	1.21732906593555	0.79181127992361
C	-3.64972683217770	-0.75389879165587	-0.57676033918525
C	-4.20551281127443	0.29702102046998	0.14469408911306
H	-1.84496310655137	-1.69024407318703	-1.23144816236473
H	-4.28958373848001	-1.45433880340895	-1.09353217167336
H	-3.82740299964705	2.02010388937232	1.36632978783636
H	-5.28034051146703	0.39869399889436	0.20357351104278
H	-1.38155616869920	1.78867283169220	1.24561692627499

List S14. [Re(O)$_2$(ap)(isq$^\bullet$)], CPCM (acetonitrile), M06-L

Re	7.26916477624510	4.70483634001398	16.02805324941825
O	8.36749250520672	3.74010049873212	14.54617228202445
O	6.21142176214006	2.90914059391234	15.95104978814746
O	8.54012292390833	5.85110570282171	16.23834155525689
O	5.96848271319863	5.45120294812772	16.87919383565736
N	6.23911926495049	5.00521225984942	14.27573400912003
N	8.28871494593883	3.39935086267957	17.23608248265257
C	4.96482937830296	5.63484886990576	14.27869622857087
C	3.81038541018313	4.87402066046209	14.10518372781523
H	3.89467143972559	3.80528595863497	13.95452492294894
C	7.81917372068582	3.55361994179159	13.38143669488384
C	2.56998455167533	5.48752093726233	14.14905689886150
H	1.67603818050339	4.89158138331251	14.02589658826836
C	6.75538456882796	1.83937230554918	16.44966513958238
C	7.92811980897171	2.07840703936541	17.21512906623870
C	5.95134433975933	4.21633730800257	11.94771218282477
H	5.05574262265955	4.79724144847292	11.79608417238689
C	8.10147773973096	-0.26195998967454	17.70358660180879
C	6.94644111871290	-0.47385522010887	16.91268360270185
H	6.59657610646505	-1.49092028123322	16.80622364456180
C	6.61473102304798	4.27908638019879	13.17653083744688
C	4.98277319707643	0.29360060295495	15.46233398801407
C	2.47357438910954	6.85646394371583	14.36113121444389
H	1.50366272539032	7.33302715389435	14.39414522726748
C	6.24254344230902	0.53349649891714	16.28463813946168
C	10.71954484985313	3.22679449750242	17.33558442324747
H	10.68640722921909	2.51846206018162	16.51796162725088
C	9.53040426058069	3.76276142463787	17.82646180551075
C	8.57624608176202	1.02356933377184	17.86497134830613
H	9.44432581128417	1.22822966773031	18.47104813709145
C	11.95757906948287	4.53313982075231	18.93053472862296
H	12.90277753637913	4.83508977876051	19.35989919427504
C	4.61527358581967	-1.18599815642861	15.41583494068431

H	5.40009151212728	-1.78523596543215	14.95453323912209
H	3.71012264770725	-1.31442357685474	14.82437763500260
H	4.42215379497780	-1.58962384377599	16.40946108979535
C	8.78255326707476	-1.46863153470910	18.33450759938918
C	9.55789725524858	4.68760373762521	18.86350956414609
H	8.62689577270288	5.10002098651466	19.22930268771138
C	3.62529478018385	7.61007618353140	14.53871142751665
H	3.55631124986326	8.67600030638164	14.70709735170293
C	5.76216790638410	3.25485381651380	9.60030755715428
C	3.81125599420752	1.05901570047124	16.09335136568441
H	3.62693778410937	0.71728133874310	17.11147637586699
H	2.90249038597710	0.89164999090252	15.51478672475919
H	4.00081079366343	2.12924976853652	16.12189244679661
C	6.44798668793713	3.40036073443356	10.95183432880846
C	10.77094201801274	5.06560084596275	19.41602169991338
H	10.78859228741470	5.77902575246871	20.22839115965437
C	8.34920834841541	2.71723203487397	12.37371656745754
C	4.87024868748045	7.00301354594788	14.50426290052783
H	5.77474664437525	7.57875736582592	14.64913186020953
C	11.92795072520994	3.61660398931314	17.88797666956696
H	12.84957798573807	3.20713450183657	17.49737004369378
C	7.63593090307493	2.66924641625137	11.19333190687065
H	8.00270201261384	2.04045258368756	10.39448472306702
C	5.19313847336141	0.77763551670011	14.02153491794270
H	5.43834800807126	1.83758934929547	13.98255474525730
H	4.28300939823756	0.62031625417879	13.44192230736835
H	5.99654628695415	0.22484403194559	13.53395134967797
C	9.20972909437848	-2.43402825724161	17.22128907002287
H	8.36171491176725	-2.77617195658608	16.63010779501807
H	9.69154137951336	-3.31300571300907	17.64941844175918
H	9.91936010682397	-1.95827629454881	16.54466467723847
C	10.78394233509499	2.90115579451416	12.88354971948838
H	10.95305404294766	3.55216055642351	12.02620771559338
H	11.70452772395391	2.34743407414344	13.06880167984681
H	10.58595104224297	3.52725309314160	13.74970128611654
C	9.62944867460546	1.92729947842722	12.61293472008011
C	10.02195662160507	-1.08142552517231	19.13396611182630
H	10.76802397162501	-0.58858192257870	18.51029478052352
H	10.48114575345432	-1.97597782203995	19.55214669833846
H	9.78237797679835	-0.41564385550166	19.96274827931481
C	9.45247086451575	0.98986841883965	13.81582180645608
H	9.18973531457479	1.53497699853700	14.72036140059033
H	10.38081682888021	0.45014797507758	14.00536909639488
H	8.67269301395959	0.25144234085109	13.62673786720366
C	4.46453180173045	4.05240924240065	9.52769275397229
H	3.74935037766075	3.72757049107200	10.28402501892316
H	4.00016385708531	3.91331595165967	8.55255366800825
H	4.63405191383884	5.12036187943945	9.66161966323111
C	7.79640519931450	-2.17818334323421	19.26892510162339
H	7.48029354801639	-1.52151469313491	20.07909702376055

H	8.26340108435656	-3.05783572216724	19.71200593673556
H	6.90432749509144	-2.50893408095991	18.73950314737677
C	6.70932152578560	3.75254571615727	8.50200501384591
H	6.95794841470400	4.80362910767496	8.64516906330634
H	6.24009602169664	3.64667943862435	7.52385853834406
H	7.63930610439536	3.18583752649110	8.48607457272777
C	9.99860428930581	1.07367237926714	11.40389177639843
H	9.22192937505618	0.34580673830551	11.16935232664687
H	10.91365048115728	0.52247658072819	11.61639775782096
H	10.17558616012022	1.67897699021620	10.51526816110123
C	5.43541004819698	1.77789587545193	9.34914891917958
H	6.32900630587394	1.15594368389589	9.33901069144087
H	4.94533399613280	1.66434097489580	8.38227931435551
H	4.76311365350842	1.38729347430023	10.11292287536931

List S15. $[Re(O)_2(ap)_2]^-$, CPCM (acetonitrile), M06-L

Re	7.27998887438500	4.63871944019781	15.99436316644971
O	8.39786469892350	3.73430121852137	14.49596983736267
O	6.25461078755604	2.83391473987744	15.86155594265184
O	8.54535517789775	5.80267371830892	16.23869131851498
O	5.95637758593979	5.35533914112064	16.85949964416810
N	6.28926442559269	5.04323649411836	14.26236533676660
N	8.25351509973210	3.38097344036083	17.25471126290341
C	4.99884246762228	5.63282611653817	14.28133415663187
C	3.85510073445164	4.85110151243528	14.12243318245492
H	3.96310091481501	3.78483039734445	13.96691060149535
C	7.84228545479023	3.59298719767530	13.30781957295777
C	2.60000265924237	5.43394740232169	14.18174454273101
H	1.71859352179724	4.81666035431322	14.06959517863280
C	6.77061764290439	1.77254322253021	16.44985769124198
C	7.89576248113138	2.03578515282089	17.24730651175713
C	5.97078175341422	4.28538615612684	11.91532293853354
H	5.07138652774532	4.87022044715044	11.79791565075630
C	8.06022722681646	-0.29157812711218	17.83847904124136
C	6.94608652436844	-0.53141530308108	17.02485631054011
H	6.59180104162394	-1.55035791078296	16.94573058125766
C	6.65264363932584	4.32015573618398	13.13048901889936
C	5.04350040767677	0.18188811664070	15.46059109062098
C	2.47157203517304	6.80057559699328	14.39669733720354
H	1.49095186361337	7.25390600447861	14.44402549584367
C	6.27120936654043	0.46481022929489	16.32257017175591
C	10.68724553184011	3.16986474085983	17.27585840992511
H	10.61438500498503	2.47034936998222	16.45260492860913
C	9.51855812191039	3.71704172232551	17.80458891246812
C	8.52619767940595	1.01151119256011	17.95117404274655
H	9.37376381314935	1.24635706458097	18.57712184266775
C	12.00103579912546	4.42553256120645	18.85196088030276
H	12.96427983109807	4.70193122492645	19.25825990439874
C	4.66718741300675	-1.29701374192516	15.47011285252270
H	5.46679275669605	-1.92330588931363	15.07467396941616

H	3.78765241400402	-1.45273790292474	14.84639965644337
H	4.42861267893600	-1.65117331885531	16.47291370782332
C	8.72017917061988	-1.46143727775896	18.56504496023770
C	9.60192277810373	4.61970775226688	18.85911641496687
H	8.69025713478683	5.04448245580118	19.25883577868491
C	3.60893753262414	7.57935117732029	14.55624378626989
H	3.51814051334237	8.64391546842677	14.72566696931574
C	5.72364195103409	3.37563836480181	9.53993625546179
C	3.84739929209029	0.97809586762881	15.99836144179063
H	3.60551423025283	0.67419163601626	17.01708833372529
H	2.96588215504528	0.80241940119411	15.38008006730578
H	4.05211101991016	2.04602470845276	16.00309936045081
C	6.44600302841162	3.49187523643415	10.88015800940127
C	10.83839061292827	4.97052295052812	19.37950221756461
H	10.89327162314474	5.67189450425540	20.20129118420164
C	8.35490026972456	2.79192223068251	12.27301082393248
C	4.86747345149051	6.99973124840314	14.50058246025174
H	5.75979396448488	7.59780053698569	14.63282778273873
C	11.92026040340107	3.52514460690716	17.79704163793709
H	12.82195994640456	3.10205195303860	17.37440435305447
C	7.62784773780368	2.76912610735881	11.08525256124100
H	7.99024176068414	2.15717673323457	10.27044727819150
C	5.31748390340316	0.59063998712929	14.00721490416489
H	5.57476757350519	1.64521881389618	13.93221448725063
H	4.43640206649246	0.40773190173974	13.38978456985507
H	6.14201361685509	0.01176582087580	13.58948840322856
C	9.20610172477705	-2.49123783444352	17.53888193741996
H	8.38537449322967	-2.86711708641580	16.92931383462363
H	9.66692108194801	-3.34400567794668	18.03893076807977
H	9.94725446280538	-2.05687583060945	16.86745333224462
C	10.79732568249190	2.96861936854346	12.76173147933817
H	10.95371312851236	3.65392872820940	11.92839056732873
H	11.72586495632009	2.41791477589298	12.91817508216510
H	10.59886648463824	3.56003609674017	13.65255095199100
C	9.64494177058984	2.00040692101018	12.46642894845369
C	9.91725888874042	-1.01738711072986	19.40024501042873
H	10.68692096615152	-0.54914358462737	18.78609261508346
H	10.36713752410729	-1.87883931180515	19.89271970898645
H	9.63001684199165	-0.30667528927430	20.17495553425384
C	9.49267502975206	1.01982605523133	13.63649243000762
H	9.22910045384900	1.53635015806821	14.55713875928253
H	10.42954339909317	0.48492201801642	13.79983096733640
H	8.71874081209777	0.27983958376701	13.43031482787151
C	4.43767772249488	4.19540801246996	9.50706975678100
H	3.73243715023189	3.87074813575195	10.27303726500441
H	3.94986308968823	4.08111444542391	8.53951329553236
H	4.62805378570528	5.25797719879939	9.65685417185057
C	7.70063663075343	-2.12914733112867	19.49400667737718
H	7.33307315498019	-1.42837447821365	20.24382763406168
H	8.15189904770587	-2.97398531988871	20.01575867656570

H	6.84072216754031	-2.50223488468743	18.93934087510891
C	6.64431765939532	3.86853991021516	8.41834979001679
H	6.91235787004294	4.91504296942866	8.56340911820738
H	6.15378022707906	3.77839020548808	7.44835477965253
H	7.56653547682784	3.29019455487323	8.37849085350529
C	10.01266510177581	1.19113172859111	11.22572478364253
H	9.24173416796365	0.46302283139221	10.97336964993013
H	10.93605434690889	0.64270871516124	11.41026555950280
H	10.17351297993396	1.82652154429079	10.35499420215858
C	5.36305230973895	1.90889107964539	9.27804294640710
H	6.24835394282775	1.27532573939204	9.24354033705106
H	4.84641627473597	1.80798820146152	8.32278936454377
H	4.70591349879309	1.52013305848745	10.05671977628309

List S16. •CPh_3, CPCM (acetonitrile), M06-L

C	-0.00161007254553	-0.10571056737590	-0.07963546547669
C	0.84582290813535	1.07431371380613	-0.05806279823658
C	0.59470160859935	-1.42849455147305	-0.16045059312683
C	-1.44561838147586	0.03417478996813	-0.00490288391850
C	0.42023526164849	2.29510682866328	-0.61576505020789
C	2.12622646231002	1.04723876509045	0.52591851754786
C	2.92874746584436	2.17276994515989	0.55375950492746
C	1.22904660917189	3.41614625111683	-0.59479354575002
C	2.48816231142249	3.36536973820578	-0.00825171618805
H	2.47579593337087	0.13106456566004	0.98256179125760
H	3.90194904979229	2.12229736439969	1.02365882289034
H	0.87796024399678	4.33547043045565	-1.04443499858694
H	3.11769724449819	4.24441606011538	0.00944119839135
H	-0.55126444639418	2.34583896872323	-1.08902815225404
C	-0.00525941280834	-2.54060135148643	0.46041564280391
C	1.79599585080651	-1.65461626166413	-0.85895559509059
C	2.35774940522613	-2.91515874729243	-0.93391930118802
C	0.56451858091187	-3.79786785832047	0.39223090421397
C	1.74897480245249	-3.99666191478327	-0.30729463810474
H	-0.91921580646786	-2.39795483599379	1.02137967383183
H	0.08590020743303	-4.62809797230011	0.89450259890136
H	2.27337349181608	-0.82714152899367	-1.36667680575567
H	3.27413646116586	-3.05846294372175	-1.49096137751050
H	2.19219318585376	-4.98136786239344	-0.36525108328826
C	-2.30545372943393	-0.87779172097012	-0.64593037312912
C	-2.04580462763451	1.08461646342746	0.71559677859826
C	-3.42005264415643	1.20949247123355	0.79421748261220
C	-3.67918825857596	-0.74406694412435	-0.57370259918171
C	-4.24790457548999	0.29813037682832	0.14946389810958
H	-1.87714429604669	-1.68346948155404	-1.22671769891268
H	-4.31233815889216	-1.45308087554336	-1.09029424975781
H	-3.85091865206931	2.01810153757686	1.36945739701249
H	-5.32289874132083	0.39846365661792	0.21001324366525
H	-1.41486528114424	1.79056349094171	1.23859147090116

List S17. [CPh3]+, CPCM (acetonitrile), M06-L

C	-0.00525494892294	-0.10655755707152	-0.07513147669658
C	0.83406905630753	1.06015039043201	-0.05520615449869
C	0.58607482541865	-1.41430805579699	-0.15900432744201
C	-1.43388631875274	0.03238404192088	-0.00297576615976
C	0.38952380519635	2.27797096292226	-0.60930015388844
C	2.11856749563309	1.00907322123203	0.52283474320912
C	2.91580144603337	2.13309267795491	0.55368448414705
C	1.20484399166473	3.38908902844947	-0.59630114458437
C	2.46427171130507	3.32110245481230	-0.01068800126157
H	2.45047661802276	0.09516576711599	0.99403440993288
H	3.88760260454735	2.09079513130661	1.02393369906653
H	0.86598302630411	4.31003337707462	-1.04845069778436
H	3.09629468875607	4.19837380493143	0.00488239237644
H	-0.57406281791446	2.31717511218424	-1.09685819580900
C	-0.03004246052468	-2.52127479380578	0.45947508347315
C	1.79602164336733	-1.61562246401641	-0.85424905494123
C	2.35312568891700	-2.87323614062469	-0.93494126912962
C	0.54934357562764	-3.76962618279192	0.39517893970682
C	1.73608416320352	-3.94969127648936	-0.30639353438034
H	-0.93171035893134	-2.37061481595799	1.03571190620874
H	0.08380884106172	-4.60514310374545	0.89786280621693
H	2.25400546658124	-0.78524379377650	-1.37243158209858
H	3.26597517599915	-3.02270757709208	-1.49331445513916
H	2.18242788039037	-4.93291472380268	-0.36523970848227
C	-2.28013955130024	-0.89749806289611	-0.64059463886372
C	-2.01773423748773	1.09945502246343	0.71027861471348
C	-3.38811314229604	1.21827869333303	0.79044178208368
C	-3.64900521401256	-0.75473877167815	-0.57570759205635
C	-4.20492258487447	0.29707096246548	0.14440953836953
H	-1.84379969502144	-1.69141751431900	-1.22963165120267
H	-4.28852484901214	-1.45673951079404	-1.09094708349466
H	-3.82665888340240	2.02245852677245	1.36333469162738
H	-5.27987098795889	0.39878565973237	0.20315005425807
H	-1.38092565392499	1.78990950955515	1.24433334252356

List S18. O_2, BP86

O	0.00000000000000	0.00000000000000	-0.11041408640969
O	0.00000000000000	0.00000000000000	1.11041408640969

List S19. [Re(O)$_2$(ap)(isq•)], BP86

Re	7.23899736045332	4.71325771219196	16.05316290473255
O	8.34153460604243	3.72635118668632	14.58879291756375
O	6.18217938685348	2.92112726826526	15.97911482115460
O	8.51829898818520	5.85677928394715	16.25962420696545
O	5.92822164971144	5.46466370296385	16.89234521294555
N	6.22691035164325	5.04189270648606	14.29634667014486
N	8.26127705482447	3.42838679284777	17.28361210659459

C	4.95855660349981	5.68552039344094	14.28438336335319
C	3.79549333584872	4.93225275246043	14.07444075932064
H	3.87848842427265	3.85669902730425	13.92497330412287
C	7.79095803985803	3.54999984919413	13.41317418135381
C	2.55310924028128	5.56150129390392	14.09361661518629
H	1.64832506795670	4.97152634633313	13.94616356143846
C	6.75181472982762	1.84632965159780	16.46603640054090
C	7.91283534373796	2.09856909830909	17.25468051860047
C	5.95512643489781	4.27536869112711	11.94964162136777
H	5.07576766391523	4.89172998823178	11.79133130296041
C	8.13127992603944	-0.25725169694071	17.70723327729731
C	7.00292974179463	-0.48412427142439	16.87536782526222
H	6.68335128208882	-1.51321839629671	16.72636955535677
C	6.60807306536265	4.31599526755263	13.19248845407312
C	5.06892468484749	0.27110036514299	15.35702527864813
C	2.46519061945583	6.93876325003771	14.31639347727070
H	1.49160181593107	7.42846983032091	14.33166282733770
C	6.28735876216741	0.52593237057602	16.24569480805996
C	10.69745186608620	3.25483186524214	17.34482817236685
H	10.64643045926156	2.56322021514561	16.50507836842742
C	9.51020459144214	3.79236558381511	17.85949008459156
C	8.57672237143895	1.04325744316586	17.90120548967387
H	9.43895271817949	1.26033310569923	18.52405462818148
C	11.96477526028928	4.54891886471718	18.94913775046400
H	12.92350700431980	4.84709408381510	19.37322655945821
C	4.77275477970586	-1.22898383034330	15.21852405016057
H	5.61501787165083	-1.77169326622487	14.76580234630646
H	3.89843348729635	-1.36708877112688	14.56781257978148
H	4.54194316969590	-1.69279394749236	16.18808447348949
C	8.83093364759953	-1.46008721729550	18.34323175471573
C	9.55022480203264	4.71527289155876	18.90804333623563
H	8.61692687840309	5.13526310813850	19.28036177565696
C	3.62698897744727	7.68325057555183	14.53293107880743
H	3.56318222342285	8.75571278088312	14.71663195857318
C	5.73180507251503	3.27908890454213	9.60853905017148
C	3.83452294121703	0.95847417354279	15.98242754738577
H	3.62721902031543	0.54769427996848	16.98038992313102
H	2.94979524649313	0.78619201883242	15.35161630030834
H	3.98782475319314	2.03926088546227	16.07955339103955
C	6.43140066473108	3.42504988918883	10.96121666929826
C	10.77841662773414	5.08678202448018	19.45341869035625
H	10.80808523094323	5.80506824291156	20.27266973454267
C	8.29459893444317	2.67645715454452	12.41748966507364
C	4.87403131219511	7.05999452425303	14.52331871735906
H	5.78791654049697	7.62429514292325	14.70334843077754
C	11.92060832861575	3.63719657469007	17.89029081457571

H	12.84454401920281	3.23055615934532	17.47867733236760
C	7.58573966135124	2.64064229898883	11.22383214214196
H	7.93402115480603	1.97464588824775	10.43753554371558
C	5.31610637997292	0.84452874517617	13.94369546772223
H	5.48418744174708	1.92588224415633	13.97366369376266
H	4.44451832702655	0.64626410486412	13.30320071593561
H	6.19451696668949	0.38213939937497	13.4736926803320 2
C	9.33861361207302	-2.39340971951973	17.22225703498546
H	8.51674977764682	-2.75432017677748	16.59006207091882
H	9.84312507786766	-3.26989763654029	17.65435366126726
H	10.05381109857014	-1.86639165391872	16.57584764172431
C	10.73958370030302	2.78252683773734	12.95354487563647
H	10.92724373187501	3.43240145020807	12.08738422890364
H	11.64861554275994	2.19162543155233	13.13949305669606
H	10.55167547125202	3.41762403923198	13.82646080151854
C	9.54693405987805	1.83878736652645	12.67984481126390
C	10.03090591168207	-1.04356783629286	19.20565727126949
H	10.79431091477961	-0.52114977489957	18.61241792924536
H	10.50007846373358	-1.93629287441819	19.64148334027496
H	9.72943083085366	-0.38650948757407	20.03311643856670
C	9.32470884681245	0.92195536747888	13.90379501129307
H	9.12930337716652	1.50293302285849	14.81095144140654
H	10.21882921835524	0.30457850287260	14.07326812905159
H	8.47152931745461	0.24875539626034	13.74575304280390
C	4.53268002555372	4.22800406770031	9.47186962883577
H	3.76408785280780	4.02731206847858	10.23123128736715
H	4.06701343757312	4.09572952430443	8.48548585421485
H	4.83593963598202	5.28054968428416	9.55988938124024
C	7.82660272525283	-2.22150727897720	19.23447855595759
H	7.45495660761407	-1.57484792820537	20.04128551060413
H	8.30890686277126	-3.09872546418598	19.68996219295900
H	6.95987331494328	-2.57377878746062	18.65994721698490
C	6.72886647701955	3.58797313325806	8.47195355994529
H	7.10333977888852	4.61780052924112	8.55115136551529
H	6.23671214146721	3.47469568887855	7.49504596710444
H	7.59370984218929	2.91212152748653	8.49169781499930
C	9.89570822151935	0.95157532127467	11.47663823681452
H	9.09128138140261	0.23784116550378	11.24735735782347
H	10.80046118921566	0.37098778798874	11.70353277679088
H	10.09937110749138	1.54604188689015	10.57477768371909
C	5.22462354228402	1.82691993708534	9.46870360965940
H	6.04964788775743	1.10368201913508	9.51223962601395
H	4.70830491778098	1.69475879604657	8.50665684476411
H	4.52031518596382	1.58324746148530	10.27601285331934

List S20. $[Re(O)_2(ap)_2]^-$, BP86

Re	7.22583594419521	4.73318654329685	16.06962927741574
O	8.35457789014000	3.74152116195807	14.63489054393995
O	6.15684717019288	2.95514647646380	15.96609887674887
O	8.51688889126539	5.86882773134715	16.31520289268270
O	5.89499486861813	5.50011038149578	16.88025150041851
N	6.28286611761642	5.12072775860783	14.30030807866639
N	8.18635490536223	3.46865763095308	17.35244303444274
C	4.99952517478046	5.72241093658710	14.27785520550274
C	3.84641758750513	4.94642651286087	14.08573083312462
H	3.95296030660364	3.87008581856412	13.95887312276394
C	7.81407544008645	3.57530356474241	13.43846322379467
C	2.58974206078971	5.54769850042846	14.08934100501491
H	1.69859834566462	4.93253183173452	13.95461954010115
C	6.72188678961756	1.87009162687867	16.47090686324150
C	7.85642776847699	2.11512406557576	17.27632398724054
C	5.99790958321759	4.28006030724147	11.96620524612786
H	5.11850699664592	4.89661660948880	11.80262182101582
C	8.10810821345908	-0.25740037806444	17.67525681898540
C	6.99271985551564	-0.47756135491919	16.84774200860481
H	6.67791479027366	-1.50476672435755	16.67014892775143
C	6.65422209088259	4.34654429949491	13.20043506446893
C	5.06119892756927	0.30611428138594	15.33172220416000
C	2.46667598912293	6.92746716578096	14.28273388489356
H	1.48145966692694	7.39575318932111	14.28775532973036
C	6.27333011280124	0.55409985014266	16.23435356754934
C	10.62768988824388	3.26437973196417	17.32040537673589
H	10.53765055459780	2.59199877027284	16.46847758730251
C	9.45923798351213	3.80116047241366	17.88144249917179
C	8.52981706597109	1.05625213254634	17.89583523223655
H	9.38903105459126	1.27349359494622	18.52435010532529
C	11.97169626209710	4.50447101439044	18.90843065439926
H	12.94924863633475	4.78086834598522	19.30591020669469
C	4.74289159957628	-1.19016907928585	15.19524606345447
H	5.58072434541824	-1.74457094858248	14.74782443674949
H	3.86877656493079	-1.31678207842582	14.53965980702447
H	4.50472567227885	-1.64886918853246	16.16631139372805
C	8.83826540308376	-1.45983613580175	18.28594609990079
C	9.55739550004499	4.69659473882404	18.95186544971683
H	8.64124110047118	5.12181787770024	19.35986009428538
C	3.61416942839830	7.69874338990340	14.48315645788569
H	3.52809957936968	8.77409838420347	14.64685753762282
C	5.73348878974749	3.22987033601003	9.64150318032708
C	3.82703283731078	1.00990949874341	15.93882220969746
H	3.60200921781695	0.59969458352737	16.93420603294799
H	2.94608491091531	0.85588299713265	15.29536806593313
H	4.00467939778006	2.08670222721087	16.04174456705989

C	6.46096756648943	3.40485499200195	10.98025842442799
C	10.80831334965722	5.04540092070846	19.46135199365954
H	10.87426030288615	5.74840982622540	20.29322877608042
C	8.31119373230091	2.69508937439876	12.45506212077496
C	4.87458969347346	7.10126414026094	14.48094926893311
H	5.77769647993467	7.68596235523498	14.65173505072966
C	11.87453601969349	3.61623082706039	17.83211369650755
H	12.77792649994393	3.20546974024109	17.37839398291603
C	7.60551177556690	2.63322012027943	11.24835723299616
H	7.95611696716606	1.94706352952088	10.47895437758722
C	5.32771702616485	0.86976387060770	13.91902412569494
H	5.53840530900888	1.94342006410086	13.95130286010912
H	4.45117855056135	0.70200641236814	13.27387922834740
H	6.19252616705280	0.37907794512315	13.45238523548965
C	9.35269638143681	-2.37340325290628	17.15284214504813
H	8.52721550310471	-2.73734195256944	16.52685040398572
H	9.88139357940002	-3.24768421457607	17.56515663457581
H	10.04477910385921	-1.82171961099667	16.50150493866927
C	10.74049962060828	2.77477610106914	13.08259014483771
H	10.96571953133762	3.44131612060314	12.23698963239016
H	11.63966842368593	2.17580037401396	13.29728989780542
H	10.51194056551412	3.39504534141372	13.95654345158369
C	9.55432248660782	1.84719369129182	12.73986710776436
C	10.04289306832057	-1.03585156971307	19.13941875386847
H	10.78420826810566	-0.48492127632933	18.54406648448487
H	10.53688273047936	-1.92689540391790	19.55489928492516
H	9.73879709604479	-0.39563180434066	19.97925905788082
C	9.27898497747443	0.89944636163071	13.92793070900031
H	8.95584478552686	1.45005012437116	14.81769996800893
H	10.18732857941217	0.32928584280616	14.17822908561397
H	8.48701022855169	0.18172424517779	13.67454369431737
C	4.53856667178170	4.18451397691403	9.50188198740367
H	3.78683542298545	4.01046822057593	10.28424088722470
H	4.05112321865869	4.03229799870073	8.52716567736640
H	4.85311079040218	5.23589999770281	9.56242093696749
C	7.86686616396343	-2.25631338858834	19.18247088399195
H	7.49910858720753	-1.62599067550852	20.00446611448677
H	8.36663859678453	-3.13726086901738	19.61629027461678
H	6.99435868740573	-2.60410665381605	18.61359295582296
C	6.70408554092007	3.50174226465871	8.47375505907476
H	7.08384721276196	4.53215475940494	8.52070447399984
H	6.19731220690969	3.36220659029211	7.50550244643909
H	7.56873127599585	2.82570331815003	8.50487216894976
C	9.95292249992994	0.98873677925303	11.53025605068077
H	9.15988386901919	0.27707258527127	11.25811694476685
H	10.85319193268463	0.40661359349864	11.77699909261722

H	10.18055913052653	1.60579133646211	10.64860303633071
C	5.20725529313040	1.78185821063219	9.54126671720088
H	6.02931646471727	1.05536568752327	9.59095023759651
H	4.66700871481437	1.62581050240632	8.59365656327403
H	4.52291607020934	1.56526207816838	10.37327880558699

List S21. [Re(O)(ap)(isq•)], BP86

C	8.33396585351678	3.42083128817026	4.79243091779468
C	9.19542801412917	4.49890437410786	5.08381655632613
C	10.54791330960479	4.27162737783790	5.33784892680737
H	11.20632674130472	5.11585089983084	5.53174793662727
C	11.03728716934030	2.96175805805047	5.32631288331396
C	10.14590636111705	1.90909159454837	5.05837012828513
H	10.52801022154632	0.89344764322471	5.06315651675933
C	8.78266191511545	2.09427202836227	4.78091803734909
C	9.21161351499517	6.93280632175735	5.35595830049848
C	9.18996173450125	8.01168857147328	4.46609639736887
H	8.69911501832490	7.89114119089453	3.50048891601675
C	9.77802559785644	9.22386643440382	4.82623006710389
H	9.75100575597850	10.06215757525871	4.13018093705579
C	10.39168457004380	9.36614039460897	6.07216798838455
H	10.84647725361817	10.31564066725963	6.35369694168872
C	10.41105175234788	8.28809294862893	6.96126619928435
H	10.87268090019938	8.39703151977330	7.94286375681642
C	9.82039401542325	7.07575784572952	6.61081524043705
H	9.80458504530184	6.24069239754267	7.31005987883560
C	5.15137158768374	7.24752024441752	6.44063503565561
C	4.73902525734657	8.39589434457952	7.12902667023109
C	3.40021247790788	8.41270814125586	7.54804140828861
H	3.04796534097276	9.28517558371121	8.08917572482219
C	2.49496940466810	7.36667443832348	7.30046666099825
C	2.94160818664807	6.24295757693249	6.59868526170894
H	2.26999546411039	5.41611725096170	6.37513894817933
C	4.26915300090319	6.17692823521375	6.17847512666138
C	4.25733925440040	3.85256574418805	5.42945033989582
C	4.05062060903287	3.21858856633811	4.19860965515342
H	4.34894868948430	3.73289383989533	3.28538239434847
C	3.47071700689317	1.95166056053782	4.15433994321874
H	3.31914728610888	1.46300256373170	3.19192929219308
C	3.08143888365858	1.31397746774307	5.33381536320154
H	2.62629868377960	0.32441226524370	5.29727279005070
C	3.28471164342146	1.94798891238728	6.56327099166182
H	2.99556959338248	1.45017985767358	7.48907628768420
C	3.87361441240001	3.20935844327166	6.61525342148928
H	4.04965386651181	3.70466250145675	7.56966673596365
C	7.82978018632930	0.92830317684717	4.50634471405657

C	7.18094536739956	1.09992935678245	3.11609534990926
H	6.60767839627640	2.03068867037986	3.05113899411907
H	6.49699497551768	0.26161910306295	2.91812538104750
H	7.94880057771832	1.10846463122182	2.32968763858426
C	6.73922684326675	0.90374740951049	5.59796043470268
H	7.19048624808772	0.76008172134456	6.58978709086189
H	6.03958463983532	0.07612513770812	5.41500793524936
H	6.16237802601562	1.83395707219275	5.61173331570025
C	8.55913812654314	-0.42354884304758	4.52726117172058
H	9.34762616272116	-0.47700026459427	3.76326381673637
H	7.83700197822689	-1.22489145640405	4.31833788980761
H	9.01113464256286	-0.63127247464412	5.50741348719479
C	12.52622364694496	2.72299832434600	5.60351525740743
C	13.36390991911390	3.45171258121534	4.53103807422570
H	14.43750516688281	3.29893956646097	4.71538657435539
H	13.17046031101566	4.53232918782606	4.53287592726293
H	13.12842622567183	3.06943669730674	3.52814446780143
C	12.87932281866971	3.28313341807730	6.99766611605332
H	12.29463234548009	2.77791024283475	7.77905520423333
H	12.67297852439224	4.35956429524064	7.06578959445506
H	13.94712126811820	3.13021441529442	7.21262229719326
C	12.89685258918431	1.23286186828790	5.57484712063468
H	12.34691218216948	0.66089635082913	6.33527890107633
H	13.96979830210460	1.11752758410527	5.78222487233535
H	12.69739838897274	0.78228553860162	4.59264555932365
C	5.70655306466307	9.55187234027328	7.39477672160865
C	5.02244609161273	10.70903543090229	8.13803627051474
H	4.18629629343531	11.12736152484101	7.56016872206632
H	5.75243769197026	11.51405568883759	8.30009726580791
H	4.64356121048998	10.39975212471539	9.12246147305830
C	6.24045368734539	10.09531318917702	6.05148201698516
H	6.78408257398234	9.32609219939114	5.49328918611767
H	6.92966059090717	10.93256816656495	6.23486950645659
H	5.41368970811780	10.46161424072712	5.42677133152793
C	6.88238797693198	9.04985508953601	8.26041870598324
H	6.51701722044295	8.66843960639652	9.22454837963180
H	7.58041127518300	9.87605647622344	8.46100553503740
H	7.43970308313165	8.25214583046141	7.75798869937383
C	1.03498670826987	7.41848572637551	7.76649171469504
C	0.10812777907618	7.34774848531024	6.53391141058675
H	-0.94637240531335	7.38297108206749	6.84491496093529
H	0.26243640841429	6.42073646347871	5.96623424360093
H	0.29758137119054	8.19222968221206	5.85712249336721
C	0.71271701579803	8.70368151027833	8.54306626042963
H	1.33339547746528	8.79887013348725	9.44491109357343
H	-0.33900357587498	8.68734859144481	8.86098658435532

H	0.86012007754690	9.60095776372966	7.92602377555046
C	0.75424094849565	6.21274681803796	8.68832354727884
H	1.39939743951992	6.24379978759929	9.57759341399916
H	0.93581390721980	5.25974692288644	8.17405579462668
H	-0.29361907656419	6.22219996381857	9.02276091755923
N	8.54312682226427	5.72870826246631	5.00702809799146
N	4.86966344026942	5.12912332873769	5.46385307048110
O	6.23062327625498	6.39430007268514	3.11252457212086
O	7.05544297010575	3.79656162469190	4.50604593775755
O	6.43148729141868	7.05688657980582	5.99558352332490
Re	6.58230040342810	5.68170631272709	4.62544900938852

List S22. $[Re(O)(ap)_2]^{-}$, BP86

C	8.33483260366066	3.37409659875945	4.93258885036836
C	9.20705646418934	4.46135854058401	5.14227271812526
C	10.57540541346076	4.23658906995469	5.28839683045487
H	11.24096855541911	5.08878208930925	5.42171387420258
C	11.08476662945954	2.92961330853374	5.25318647209139
C	10.19187881896112	1.86784053141092	5.07082546834716
H	10.57728461852861	0.85281124560027	5.04953194681257
C	8.80586136741910	2.05471951972781	4.90504853714489
C	9.19367696573664	6.84659404599678	5.61998437932585
C	9.11025069951533	8.04863709108027	4.90131166011853
H	8.56045542644036	8.04729233228610	3.95998794381829
C	9.69476708251079	9.21328699368772	5.39469496232424
H	9.60647948623076	10.14017464067450	4.82612252805612
C	10.38286269380635	9.20116047348441	6.61055450324585
H	10.83539004401980	10.11440619663583	6.99865685246226
C	10.46907067387325	8.00714356724498	7.33355285478733
H	10.98281739785097	7.98771544046768	8.29637209533568
C	9.88031641329663	6.84152671275907	6.84720993359207
H	9.92738958353904	5.91670187648285	7.42153628236250
C	5.08891986720328	7.33661930616842	6.28328381963657
C	4.67798000010236	8.47313083098762	6.99235157270248
C	3.35359635156308	8.48262406930086	7.47139014849321
H	3.01450993361455	9.35337908179249	8.02484073662590
C	2.46233891244153	7.42508170547458	7.26004815491946
C	2.90881224006139	6.30260641116885	6.54668579239424
H	2.23928066513406	5.46493795043923	6.35497386601582
C	4.21845672176876	6.24813582350318	6.07141885618543
C	4.20770819698824	3.94390676048880	5.29236624576791
C	4.11472207271930	3.26085798266831	4.07044230987815
H	4.49408151341267	3.75612048268724	3.17651192242011
C	3.57457645173420	1.97768510589766	4.01298643222435
H	3.52428882778127	1.45923822330472	3.05441454117248
C	3.10724865287330	1.35301126529592	5.17208179707811

H	2.68922488882318	0.34667992697186	5.12725925182368
C	3.19833410587752	2.02666000282561	6.39421299877362
H	2.85939237578889	1.54141294277142	7.31126749565536
C	3.74390047331308	3.30724387074138	6.45733635304855
H	3.83822235397814	3.82252655824078	7.41275053016162
C	7.83854605546209	0.88484738484546	4.69556515524729
C	7.14134088581654	1.03835185793965	3.32670212461425
H	6.59507532590662	1.98569033863330	3.27175283600052
H	6.42713906400515	0.21516271881998	3.16779232389031
H	7.88399091265173	1.01508850845980	2.51560599411944
C	6.78364433191855	0.87878212780141	5.82230843101190
H	7.27121453840471	0.74777727001441	6.79964115471919
H	6.07153343966679	0.05213269850475	5.67719300327359
H	6.21819564623475	1.81578667352917	5.83916393841936
C	8.55938433701969	-0.47208053719316	4.71186205385943
H	9.30759713355725	-0.54899350110723	3.90936272366198
H	7.82226413879309	-1.27475912461181	4.56167897736362
H	9.06393533114611	-0.65478913348968	5.67191495055926
C	12.59665730986256	2.71991062032074	5.40883531904305
C	13.33586740593714	3.45855756217244	4.27272383192856
H	14.42600195476707	3.32984613487931	4.36755396557739
H	13.11637671375059	4.53406150453090	4.28688281375692
H	13.02349090369920	3.06740107478967	3.29419232774443
C	13.05793776678251	3.28768210897095	6.76766137763882
H	12.55105113549572	2.76787911105640	7.59305260995673
H	12.82454298252868	4.35721195167711	6.85376908171561
H	14.14535716365600	3.16337616511434	6.89365318199244
C	12.99150282714261	1.23670360511921	5.34978404007424
H	12.51321203715666	0.65935028533523	6.15357018957029
H	14.08136548132227	1.13686086420050	5.46362383088634
H	12.70872471950582	0.78396111896529	4.38909365676867
C	5.64322518999083	9.63993687543871	7.22425669573890
C	4.98644869625377	10.78767975326529	8.00670967855933
H	4.11903267124439	11.20252056977037	7.47267615820311
H	5.71859350168615	11.59759369406239	8.14122724230665
H	4.65510405596077	10.46650860098015	9.00504281264315
C	6.11023894537346	10.19791905552083	5.86283307693109
H	6.60945187347414	9.42008116263356	5.27628939292066
H	6.81766494970428	11.02811197955810	6.01509019068566
H	5.25116632409277	10.57496218275796	5.28855405492537
C	6.86314286001493	9.14762148900151	8.03190340004703
H	6.54144147685536	8.76046583466436	9.01002642187027
H	7.56862844234652	9.97518302205020	8.20311373172105
H	7.39635351562732	8.35174926615867	7.50172951029599
C	1.02088516391643	7.44158210622154	7.78579569544280
C	0.03693663190441	7.30723295733259	6.60469828855643

H	-1.00465134143504	7.30138562593166	6.96389179629884
H	0.20842990585876	6.37883886741880	6.04435730609282
H	0.15751727561688	8.14593309079437	5.90441939714847
C	0.68768099241604	8.73605780505428	8.54250232267309
H	1.33829173892723	8.86763687387394	9.41848119772133
H	-0.35379984744412	8.70343661929042	8.89592873772824
H	0.79901570319486	9.61962584940426	7.89839783220112
C	0.81776280391457	6.25493377806289	8.75225955499552
H	1.50117540404585	6.33864850931905	9.60914010329495
H	1.02029930615129	5.29692089519884	8.25547869110745
H	-0.21608777931089	6.23254501125506	9.13260240371445
N	8.54581833888128	5.69863034400737	5.12221649991856
N	4.81058563768925	5.21712978966578	5.32677233568421
O	6.47478745162439	6.07446541750916	2.91760203329781
O	7.02433557742525	3.70153796062465	4.77234766357333
O	6.33803961518192	7.19269517676117	5.76431006119923
Re	6.61934983352958	5.63537187172435	4.56241430115610

List S23. [(ap)(isq•)Re(O)(μ-O)Re(O)(ap)(isq•)], BP86

C	5.04530025358033	-2.20526593061128	7.46686364537393
C	4.55533149454143	-2.08774798079037	6.16263510237775
H	5.06879209810996	-1.43983147679432	5.45443683948796
C	3.41727180777316	-2.79982928609807	5.79248593788590
H	3.03060419574231	-2.70313706661810	4.77825188546690
C	2.78083645682571	-3.63879396702192	6.71191240995277
H	1.89658527983487	-4.20254566795204	6.41450887635649
C	3.28370291444458	-3.75919500855426	8.00963260225527
H	2.79947239704865	-4.42198397903760	8.72703239279792
C	4.41289175019876	-3.03842542742053	8.39469272676242
H	4.82168240003216	-3.13036528431122	9.39899758661923
C	7.32630158938344	0.05293452155047	9.18908106722040
C	6.09078603050415	-0.57374192285239	8.88019043102556
C	4.92286952737968	-0.25779949412523	9.59343263718007
H	3.98051898958614	-0.70599313579558	9.29602259213714
C	5.00046420199568	0.62810603765477	10.65664767378604
C	6.26372102893443	1.17063661518801	10.99970030235443
H	6.31321771691075	1.83897365600600	11.85694552772851
C	7.44136650288739	0.91817271491565	10.30164146637490
C	3.77035657355835	1.02565532587727	11.47639364025233
C	2.48647948521757	0.37983356315263	10.93560157689465
H	2.53489033082049	-0.71728154606370	10.97446308145247
H	2.29087513402413	0.67841996407833	9.89626449723782
H	1.62791763300266	0.69820221313183	11.54268266408174
C	3.59838684804588	2.55809516501754	11.43089099463163
H	3.44670888826761	2.90002183977938	10.39778771321510
H	4.47769240762025	3.07924565248484	11.83132744191282

H	2.72478452409090	2.86331947786834	12.02507804301531
C	3.97002055791832	0.57099975939244	12.93731634063827
H	4.08652821287971	-0.52053711781536	12.98948291589263
H	3.10155224250626	0.85494494476453	13.54965077062480
H	4.86289231323190	1.02677637763078	13.38583384405610
C	8.79227624130525	1.52082609483167	10.68813598468608
C	8.68005005555877	2.42352569046284	11.92436091419182
H	8.32252675462026	1.87090713372670	12.80523633723283
H	8.00510866279836	3.27390575352825	11.75076855652444
H	9.67154739811578	2.82999420825440	12.16663080316271
C	9.79615634204729	0.38925910570779	10.99992357792713
H	9.94865665379682	-0.26631232850250	10.13611628225398
H	9.45274971968207	-0.22201994103335	11.84712618684620
H	10.77003115163475	0.82120285571734	11.27199424349236
C	9.32391677052871	2.36356206948402	9.51245091795306
H	9.45389228623852	1.74958582144817	8.61583021392043
H	10.29730346385079	2.80295756239538	9.77464804034774
H	8.63061956901300	3.18012330837417	9.27188817665074
C	7.90311967453625	-3.52673245838392	9.48782047352624
C	7.64504122386893	-2.89274707973547	10.71328082060840
H	8.14252816274898	-1.95153817023371	10.93945337930610
C	6.74506327894984	-3.46009162596808	11.61300883701164
H	6.53396156602678	-2.95137662031381	12.55384281724080
C	6.11135570605378	-4.66956578604290	11.30909161016939
H	5.41041242170895	-5.11262346460176	12.01659363316488
C	6.37508175852672	-5.30318905192244	10.09232021603806
H	5.87768838891313	-6.24088701702828	9.84429293362070
C	7.25935429610778	-4.73026066639447	9.17772008303088
H	7.45003439084728	-5.19374877432642	8.21095402305046
C	10.09985245365488	-2.72057951607464	8.82697154360744
C	10.80031403664640	-3.12226810335801	9.96425590626157
H	10.28662204828770	-3.69631611572473	10.73334985647866
C	12.14124476141567	-2.74664530702047	10.11155801946735
C	12.75309216750987	-1.98766275464071	9.10090195867523
H	13.78719572982518	-1.68532385549677	9.23154717737341
C	12.08939694189958	-1.58141032747640	7.93081886915596
C	10.75481595589963	-1.97860591452279	7.82098957474006
C	12.88017926213179	-3.14082037638982	11.39601512068190
C	12.18034132754015	-2.46860265362094	12.59704230875302
H	11.13213764650130	-2.78504704324195	12.68114107614316
H	12.19163812489261	-1.37529348042754	12.48913346003859
H	12.69082701841499	-2.72977761246455	13.53597381213025
C	12.83417877308276	-4.67324323152532	11.56802291385347
H	11.80193768612078	-5.04197699826611	11.62701131491499
H	13.35236975743820	-4.97033818174058	12.49174601874608
H	13.32275687926981	-5.17511401034303	10.72138783979500

C	14.35141118236753	-2.70009508175974	11.38709001250432
H	14.44945527047488	-1.60764652259060	11.31941162708142
H	14.90454563310249	-3.14844556182847	10.54998864916792
H	14.83651330524319	-3.02050446099567	12.31968518685015
C	12.74254288195751	-0.71073329248895	6.85542902462121
C	11.99125286211936	0.63633265340986	6.76915792426167
H	10.93676154755084	0.49499072813638	6.50793488287667
H	12.45181319991568	1.27592878786340	6.00352597919689
H	12.03737592882525	1.16250687784513	7.73278541319693
C	12.68361911773422	-1.42599726815141	5.48940659268012
H	13.19154030229436	-2.39959581722995	5.53469957302720
H	13.18196091326926	-0.81213925629293	4.72574101724258
H	11.65005002544347	-1.58976381503526	5.16612676352650
C	14.21452290318355	-0.41488546358937	7.17701754453753
H	14.81181120081961	-1.33417391610573	7.25661565998043
H	14.32317950281266	0.15137796003092	8.11273323575788
H	14.64223701325940	0.19482311586327	6.36904933002941
C	9.94571532089360	-1.03471697822961	2.39218833634297
C	9.90012135525414	-2.32443945345155	2.93053067642801
H	9.60021969268649	-2.46195628560061	3.96787556702500
C	10.24324550451649	-3.41265823278421	2.13256321937393
H	10.21550148879357	-4.41675014217490	2.55511676529262
C	10.61265361715431	-3.21972242471295	0.79816369128113
H	10.87081058143983	-4.07534543446426	0.17412860147722
C	10.64320525618400	-1.92932423184726	0.26414267744752
H	10.91721946934867	-1.77256072163959	-0.77929067423912
C	10.31648633490529	-0.83204003553656	1.05930240998508
H	10.32639466512780	0.17687352168165	0.65160688080234
C	10.55021626755878	1.05198745133259	3.41330750682274
C	11.86081854975751	1.06751795884774	2.90912469846190
H	12.22351213466045	0.21535433713131	2.34407276822684
C	12.66567396300623	2.16983908909980	3.15218142029640
C	12.13215394127859	3.25118667656404	3.89547543834778
H	12.76924794658142	4.11839687411898	4.05610162938266
C	10.85386013544303	3.26828738288105	4.44654440857388
C	10.06641285884372	2.11655268553736	4.21797984820762
C	14.10534091236874	2.25561449012067	2.63989013293088
C	14.51836369947437	0.99143591110400	1.87263318501154
H	15.55819107914341	1.08834969700069	1.53136736765569
H	13.88870893653993	0.83160629136331	0.98632484272793
H	14.45640533286553	0.09455191809787	2.50463452041855
C	15.06397193578393	2.43305272615987	3.83536593799560
H	16.10357050895215	2.50903182249322	3.48456415027226
H	14.99556975346524	1.57534145906912	4.51893160327209
H	14.83648815014799	3.33929041228533	4.41203455626869
C	14.23312807238993	3.46590902395227	1.69057065847870

H	15.26466739415324	3.55442971017650	1.31959436282983
H	13.97370994096046	4.40659056618941	2.19412863370688
H	13.56420588274078	3.34939964269036	0.82657274771566
C	10.30075070135464	4.44401981124025	5.25154519543765
C	9.91869930510577	3.95422437061979	6.66120057374610
H	9.16287774398003	3.16424783990349	6.61416212040368
H	9.51302046589915	4.78850832127160	7.25187229812337
H	10.79729100661553	3.55810862644893	7.18772650902197
C	9.04945204604368	5.01310684056850	4.54713714867463
H	9.30297938863453	5.39376978991082	3.54688247338441
H	8.64199483872522	5.85004949565259	5.13224888153953
H	8.25985257179289	4.26107106386295	4.44367861647285
C	11.33193221867418	5.57210396284689	5.39358338990520
H	11.62665129264222	5.98621209636998	4.41846688877857
H	12.23841803718630	5.23278632076621	5.91477391797731
H	10.89517748000602	6.38970583905139	5.98336637057501
C	7.80601828403355	1.86162461791303	1.36968978075456
C	8.79723371600130	2.84204597987376	1.20517935812219
H	9.01964541279156	3.51507702259073	2.03123590236464
C	9.49495938358389	2.92985669467676	0.00242590984262
H	10.27623741726500	3.68174639480625	-0.11060370670364
C	9.20251306717659	2.05431439961624	-1.04865834406968
H	9.74870765944333	2.12721142878188	-1.98915007600220
C	8.21330020934156	1.08170170317864	-0.88475265203670
H	7.98707625457440	0.38929933822015	-1.69567343587560
C	7.52265363465389	0.97666134141732	0.32286478923294
H	6.77307912499490	0.20224004436722	0.47845269737409
C	6.37027385038780	2.74703943023740	3.12350743906795
C	6.06346239834572	3.97108559154422	2.51788576396643
H	6.41749759089044	4.16688374542708	1.50951824741143
C	5.34406957539919	4.92411672178719	3.23992386544427
C	4.93270351198780	4.62273150316472	4.55391380249576
H	4.39499839378732	5.38490038667678	5.11336196210190
C	5.19525447129550	3.40154956224383	5.18601387522902
C	5.91187386933505	2.46889423557239	4.42570500974045
C	4.79497264103080	3.09422233311414	6.63020263746424
C	3.86888915002243	1.86023882795082	6.66367103830502
H	2.94068184955568	2.05335512473568	6.10797225516032
H	3.60825231531863	1.61752876602312	7.70394485492682
H	4.35780419295051	0.98296773340450	6.22621829035758
C	6.06680199292091	2.80890232101314	7.45969679759746
H	6.60793638420358	1.93821988994764	7.07434292398690
H	5.79753664637700	2.61116306654261	8.50666205591144
H	6.74709931609351	3.67141364571821	7.43329106016802
C	4.05427948520386	4.27112967226974	7.27994009979969
H	3.12565852087192	4.51855652243383	6.74621576202956

H	4.68038598166714	5.17359658164094	7.32435536985128
H	3.78668748191910	4.00292157612889	8.31141185666443
C	5.01913065217368	6.30318757143039	2.65539584668596
C	3.48966457966753	6.50483588102947	2.63612804448213
H	3.05914988526260	6.44141920385726	3.64421149719620
H	3.00585198309182	5.73793838657029	2.01545467517395
H	3.23946042897600	7.49267174101576	2.22163226188922
C	5.67218630723052	7.39101133952308	3.53440800494632
H	5.30403568249702	7.34921426418855	4.56775594715883
H	5.44954375526741	8.39146793005883	3.13461502650685
H	6.76339547175846	7.26417409096680	3.56071479380889
C	5.54439793606596	6.46424888062423	1.22126692071221
H	6.63789710132513	6.36434164739920	1.17424518470074
H	5.28338119907266	7.46265041508415	0.84353698270128
H	5.10202627526389	5.72310410680343	0.54112527097412
N	6.20497868372764	-1.46407966445731	7.83632913608357
N	8.76832095351414	-2.93595580832599	8.53281273059571
N	9.60402682435692	0.06949055318703	3.22563937938794
N	7.12667236038233	1.71547602647548	2.60511031712014
O	8.31520005431772	-0.27394036840128	8.38721967959967
O	9.95174449890044	-1.66051888526493	6.77552978456971
O	8.84649415335909	1.93958872890217	4.67615170969833
O	6.27395954488083	1.24267943225662	4.87658479256076
O	7.96732009564280	-0.51535558774239	5.63921637728549
O	7.32458753871110	-3.11003949942256	6.16156441557453
O	7.25158726622653	-1.02756107755746	3.05755614190088
Re	8.02605334658802	-1.79171705007876	7.01942844389847
Re	7.74567077395072	0.34054711206863	3.97907901487908

List S24. O_2, BLYP

O	0.00000000000000	0.00000000000000	-0.11494588495732
O	0.00000000000000	0.00000000000000	1.11494588495732

List S25. $[Re(O)_2(ap)(isq^{\bullet})]$, BLYP

Re	7.25125385629240	4.65504090500151	16.01464005424813
O	8.33510302421455	3.66883072258562	14.51042714846749
O	6.20104473272757	2.83609759216929	15.97117787784923
O	8.53343011645083	5.81487852348572	16.19521951491138
O	5.94504733660359	5.40543713182891	16.88170123889116
N	6.20301979042765	4.99346650606864	14.26123129151392
N	8.30211885764385	3.37759536291092	17.25612105601101
C	4.92613114577016	5.63775474474768	14.26639766353443
C	3.75950915648313	4.87727227554220	14.09504485280526
H	3.84089123563152	3.80239780118314	13.96240471005858
C	7.78793249420689	3.54351665189031	13.32191090363678
C	2.51381643406360	5.50518579134892	14.12792827552586

H	1.60946091342310	4.91222410922804	14.00922968073323
C	6.76198595258991	1.77599942846728	16.50888948653455
C	7.93383072214681	2.04913426089378	17.28112715512052
C	5.94314657815998	4.30980733013971	11.88318874293534
H	5.05711289594620	4.91652424650687	11.75370426325743
C	8.11850908531634	-0.29131308963656	17.85029886868312
C	6.97821795941601	-0.54002112377243	17.03484180121929
H	6.64045475212315	-1.56557019373225	16.94265482300717
C	6.59492612026765	4.30828519792336	13.13076638481702
C	5.03957336029376	0.16875346259706	15.48621594830254
C	2.42669056390485	6.88846216500000	14.32678273926580
H	1.45440609527238	7.37526671973945	14.35318520696688
C	6.27468838205549	0.45027522743921	16.35756426802600
C	10.74891164581346	3.23432422233621	17.30269137090965
H	10.70266166610187	2.54867404668650	16.46170344954918
C	9.55725644646038	3.75445675868976	17.82937027909534
C	8.58655514479129	1.01184887642359	17.97343271946831
H	9.45231125577838	1.24629311194083	18.57825776658207
C	12.01423250114997	4.53295940922455	18.91146054720720
H	12.97016034875502	4.83878838852786	19.33063901659619
C	4.70439627428375	-1.33714689225221	15.43615733890908
H	5.52872670668224	-1.92393080475396	15.01211899994640
H	3.82453427584366	-1.49085089653303	14.80036773252658
H	4.47036007120736	-1.73730735839476	16.43029375468737
C	8.80325527040372	-1.47763838234640	18.54925490124618
C	9.59359017172094	4.67007481322684	18.88668265475197
H	8.66103010071509	5.07729913800369	19.26737275731584
C	3.59283611915844	7.64059696371096	14.50436846879668
H	3.53045330045808	8.71402946614937	14.66867216484706
C	5.75147150256737	3.43941382342447	9.47940840665398
C	3.81334741519383	0.91962375003062	16.07376299224340
H	3.60239099746216	0.57180286589800	17.09250941269228
H	2.92591076695447	0.72610611673247	15.45680638013144
H	3.98431347630891	1.99850455962987	16.10749082753119
C	6.43594714725308	3.51633348835995	10.85389227700636
C	10.82281136616353	5.05302062121749	19.42846146598567
H	10.84908806071359	5.76404138627889	20.25116528791713
C	8.30666893340283	2.72191212212342	12.28546611014104
C	4.84309573978048	7.01811776158857	14.48034876712304
H	5.75612235453136	7.58738221663005	14.63143073760768
C	11.97347866425239	3.62716260239008	17.84437634323058
H	12.89748993628377	3.23436159080674	17.42541285669359
C	7.60474070010523	2.73678523549177	11.08472878905148
H	7.96553376980821	2.11952955316188	10.27050464529285
C	5.29865933109382	0.64908017792075	14.03204808523633
H	5.50835001596471	1.71968191625465	13.99199305898147

H	4.41757377227977	0.44524583231075	13.41005325795397
H	6.15238389908241	0.11925138705672	13.59446496809331
C	9.31147032213352	-2.47371313887694	17.47238124842227
H	8.49140260349151	-2.85473744751874	16.85405779434956
H	9.80285902145515	-3.33185984387034	17.94877282175367
H	10.03570902278577	-1.98622960872475	16.80879098637735
C	10.76336054087723	2.81115279453697	12.85361624788450
H	10.95775042603057	3.50149105738598	12.02332104205422
H	11.66947697891169	2.21302081197779	13.01701326277809
H	10.56984227320310	3.40175484929926	13.75256205490136
C	9.56971204201451	1.87640748923223	12.51824883599382
C	10.00809793093666	-1.03616799480332	19.40674042896768
H	10.78240285730825	-0.55324129457153	18.79870412676952
H	10.45934255445064	-1.91274423218176	19.88654339645360
H	9.70847278277334	-0.33869173768770	20.19792847500340
C	9.33436294203096	0.88783103275564	13.69337581786570
H	9.09770250809307	1.41190813501274	14.62136620925629
H	10.23589340247298	0.28323868943806	13.85597026561034
H	8.50577110468911	0.20778745257117	13.46539987134722
C	4.49376833424215	4.33077244944712	9.40197768636962
H	3.74130580871057	4.03497410854545	10.14271984132928
H	4.03807821661949	4.23938067496242	8.40881887089130
H	4.73589317141365	5.38820857413804	9.56157131512440
C	7.78183929323140	-2.19215584963934	19.47347733674023
H	7.40548926137760	-1.50446689406779	20.24042725529581
H	8.25670679808398	-3.04372949111157	19.97761011123686
H	6.92201668993933	-2.57148640821649	18.91073021012089
C	6.74457997657867	3.90310853527826	8.38088918757687
H	7.06421033453292	4.93670782373884	8.55981088363696
H	6.26608757860348	3.85644174329180	7.39409997686268
H	7.64076357605396	3.27363562022272	8.34952608347161
C	9.94240842696292	1.04848993629588	11.26993461268991
H	9.14799921479153	0.34281157382080	10.99679446903369
H	10.84753557805530	0.46580295721089	11.47842996585844
H	10.15068065974583	1.68788087104628	10.40345475006237
C	5.32462148585782	1.97251585834773	9.20414170647853
H	6.18445689178987	1.29380781647539	9.19415040566739
H	4.82643845945295	1.90032662146164	8.22880710490599
H	4.62730422034079	1.62137484727360	9.97431779253306

List S26. $[Re(O)_2(ap)_2]^{-}$, BLYP

Re	7.25056524487459	4.66503215143727	16.01728785732043
O	8.35244417739518	3.67815649168323	14.53341879839497
O	6.18899568345380	2.85901395499031	15.95404198519918
O	8.54563925524770	5.81959818945501	16.22503550314102
O	5.92856038444437	5.42907954703878	16.86716123989967

N	6.25538594626702	5.06194783088314	14.25994316022726
N	8.24731904989009	3.41367063788427	17.30860872345347
C	4.95405695578991	5.64146488298948	14.26469040797457
C	3.81282043030595	4.83834445152827	14.10364220997699
H	3.93916366858850	3.76690968185621	13.97982825607533
C	7.81149692098924	3.56990529029526	13.32479760762324
C	2.54124242890804	5.41264328208371	14.13196658335221
H	1.66269700431202	4.77884105210957	14.02076373591743
C	6.73881275876640	1.79262524918630	16.52421298249070
C	7.88714466204985	2.06126055855857	17.30785766157494
C	5.98849356550438	4.33252558884127	11.88231883420890
H	5.10437340342511	4.94365682168847	11.75228717453081
C	8.08569994385430	-0.29203664158976	17.86024746255666
C	6.95753308959958	-0.53720844355391	17.05216685202625
H	6.61600998784413	-1.56151968677592	16.94630654526124
C	6.64096792345928	4.34091149648140	13.12358919681434
C	5.02912089977558	0.19316998330093	15.49064858745172
C	2.39222792255641	6.79304916079002	14.31984184301479
H	1.39934991166365	7.23887210629737	14.34472137749644
C	6.25837259505953	0.47186648311736	16.37463930679513
C	10.69648619732200	3.23018179581088	17.25379689334803
H	10.60511096349170	2.56333431594158	16.40160694168211
C	9.52842406696635	3.75577510109857	17.82986424750356
C	8.54224700073969	1.02485087264036	17.98853762070562
H	9.40673924205237	1.26206325273843	18.59582166713679
C	12.04944726957035	4.47209288521759	18.83881635153798
H	13.02680989722172	4.75315638595820	19.22732560966972
C	4.66061580630548	-1.30615179986901	15.46123200580601
H	5.47624621861726	-1.91616653752441	15.05275157072993
H	3.78130557061624	-1.45087201372093	14.82016120007144
H	4.41427779408861	-1.68609756171465	16.46108071639316
C	8.79176725266184	-1.47546282637686	18.55143975001545
C	9.63047864414683	4.64609237924854	18.90645356948965
H	8.71844543667206	5.06261934735353	19.32569998820695
C	3.52863044060812	7.59185485743712	14.48862250874952
H	3.42288307553273	8.66401788340418	14.64697232263603
C	5.77009160569253	3.44725214618140	9.47457407807755
C	3.80718344981592	0.97644803189501	16.04282122704817
H	3.56845278331272	0.64314396784746	17.06135408873833
H	2.92600995106215	0.80087977734637	15.40879952115536
H	4.01115156351222	2.04962172464787	16.07161392386465
C	6.47079363494011	3.52595154544199	10.84515629538310
C	10.88581449758017	5.00146234786340	19.40769964454495
H	10.95460493118241	5.69834696858052	20.24158521667897
C	8.32062831226802	2.74663483302619	12.29470970290200
C	4.80369112784742	7.02039066028074	14.46178298876262

H	5.69422022724168	7.62558924919768	14.60916288268360
C	11.94814724505634	3.58827795023125	17.75628177930846
H	12.84827803493648	3.18727912057048	17.29283415688328
C	7.62443772011263	2.75078952615562	11.07752689359208
H	7.98791219080464	2.12016705924174	10.27313687232227
C	5.31749609953559	0.64094739681664	14.03227992265953
H	5.58325277437161	1.69849186395367	13.98279890363906
H	4.43266977823107	0.47115284886056	13.40251576745295
H	6.14976161728127	0.06482667322712	13.61150315353830
C	9.31721793741414	-2.46067117470238	17.47366354024971
H	8.49950128147058	-2.84669181587753	16.85509944796180
H	9.82720708679097	-3.31606896981437	17.94034938883267
H	10.02713829900505	-1.95361890946273	16.80907428241062
C	10.76832739822152	2.80385076379282	12.91525209553774
H	10.99096142733514	3.49652855120572	12.09282458084947
H	11.66558970125041	2.19703595314811	13.10429996091219
H	10.54749923854012	3.39292712859329	13.80882965315763
C	9.57324609530403	1.88624115700344	12.54174341041279
C	9.99293700911417	-1.02239287344029	19.40936754937689
H	10.75507788284417	-0.52087906892658	18.80124820865972
H	10.46046061684704	-1.89520204062179	19.88415591937719
H	9.68354719599295	-0.33115074599181	20.20242940968300
C	9.30436128085086	0.88591160879874	13.69852981412894
H	9.00445825706763	1.40099240857214	14.61313363158095
H	10.20897616397358	0.29736675771262	13.90724395434963
H	8.50201887813159	0.19097692196764	13.42444942846729
C	4.51404742683048	4.34286083649698	9.40649607270192
H	3.77237018774092	4.05365444308392	10.16045564428743
H	4.04465431613638	4.25024248505496	8.41802050817497
H	4.76323081123293	5.39916075851112	9.56341900040261
C	7.79194701949764	-2.21672557988087	19.47726866788941
H	7.40932181969970	-1.53812126879893	20.24979133063406
H	8.27835554000575	-3.06871180186072	19.97425961023750
H	6.93321309855179	-2.59741928296690	18.91344303381328
C	6.74553914824113	3.90389520304803	8.35812001674292
H	7.06809363441439	4.93798963847339	8.53149930869188
H	6.26016107429148	3.85262133443592	7.37255398964200
H	7.64247512088069	3.27505578967861	8.32777923560972
C	9.97280820876455	1.06960426123872	11.29347053555059
H	9.18116150749583	0.37050958679530	10.99566616525805
H	10.87219337456414	0.48074937846565	11.51644526100281
H	10.19922652005243	1.71845463956094	10.43773817850005
C	5.32951255112699	1.98477737590903	9.20039932520349
H	6.18763488502664	1.30369691223100	9.19037142746165
H	4.82112546197388	1.90832053317966	8.22826587489653
H	4.64016030789454	1.64024495580142	9.98080366165440

List S27. [Re(O)(ap)(isq•)], BLYP

C	8.35215447156801	3.40063519117030	4.81228803756546
C	9.21414483347379	4.48291643344925	5.10261624400855
C	10.57210685497666	4.25612623921422	5.34331280142372
H	11.22611982075611	5.09841208129233	5.53906628140437
C	11.06931066373770	2.94737503822051	5.31567208053982
C	10.17765308203076	1.89172943653447	5.04376988016134
H	10.56311458551901	0.88196994127487	5.03289974271691
C	8.80840995747295	2.07442506651415	4.78095196311289
C	9.23754471699526	6.92871154052707	5.37179364796483
C	9.23391062920517	7.99068233406847	4.45830682785893
H	8.75175400055188	7.85671439402353	3.49331927781182
C	9.82797964384707	9.20792934991194	4.79997479258019
H	9.81426845781889	10.03018970868700	4.08811374526209
C	10.42922970334420	9.37244061425961	6.05173290926424
H	10.88607759190204	10.32271522020291	6.31830225921108
C	10.43082323533407	8.31124799734079	6.96457691633126
H	10.88116557129941	8.43712637020871	7.94687232220069
C	9.83450648934298	7.09420758724924	6.63145585021313
H	9.80698567267060	6.27601781541583	7.34582074959284
C	5.13108680263760	7.25309958385380	6.47239872446220
C	4.71394154152938	8.40887591823264	7.15064368249572
C	3.36818595904330	8.43287162017055	7.55513382808646
H	3.01382572462406	9.30644993403254	8.08445562484609
C	2.45937987067543	7.38671370394310	7.30253744717124
C	2.91260668521516	6.25608134945046	6.61216289459965
H	2.24326363599905	5.43305260104867	6.38598045081847
C	4.24668656727555	6.18098990575276	6.20555019338917
C	4.22978771079207	3.84210052793175	5.46300219678364
C	4.01106282690070	3.21338577986464	4.22920548741889
H	4.30310282420017	3.72772898591168	3.31744896580105
C	3.42915460279244	1.94492880844895	4.18294363526529
H	3.27100693776221	1.46178903694411	3.22135510702512
C	3.05026402499412	1.29928520957602	5.36398610446287
H	2.59693769606575	0.31141059502606	5.32633648017674
C	3.26531782143574	1.92772091985733	6.59687273197384
H	2.98543148331043	1.42582077308041	7.52067957219276
C	3.85568487327090	3.19079850149923	6.64994898244870
H	4.03881198168433	3.67838301095010	7.60390089977981
C	7.85432890467575	0.90146216694869	4.49622301595164
C	7.18747815337182	1.08907153090984	3.10732609848709
H	6.61125186410012	2.01528123341206	3.05849725285324
H	6.50756187974561	0.25128754650174	2.90507588411612
H	7.94783192221222	1.11021649051074	2.31658829046536
C	6.76917540446838	0.84963114995413	5.60336194480808
H	7.23190010400133	0.68422962066846	6.58438439883931

H	6.07177875561472	0.02558704027881	5.40954124107359
H	6.19195284069231	1.77456497643606	5.64559480578502
C	8.59213068381429	-0.45528296481334	4.48677429832986
H	9.37031343959914	-0.49204248108334	3.71466967507926
H	7.87186182564732	-1.25395517243696	4.27287356970014
H	9.05502509232360	-0.67701351525297	5.45606233375457
C	12.56963630509259	2.71183452303690	5.57642157425275
C	13.39799155268921	3.45015907381600	4.49168857318714
H	14.47170142087426	3.29545941302929	4.66059614807398
H	13.20588575924456	4.52856203165312	4.50204644403560
H	13.14914288266471	3.07332944240480	3.49218140001397
C	12.94181400908977	3.26941425277989	6.97544227733162
H	12.36739080385683	2.76151675449952	7.75984978288093
H	12.73766810191983	4.34329648363104	7.05043628334177
H	14.00996165984522	3.11463735375270	7.17576995533465
C	12.95047431606388	1.21625713120088	5.53787460195261
H	12.41172443906609	0.63966188504613	6.29949630890729
H	14.02390147171334	1.10663907753913	5.73404131903939
H	12.74423959048052	0.76988060951812	4.55778461669818
C	5.68713944177657	9.56968297922669	7.42114097401729
C	4.99823654206074	10.74125452861984	8.15391191942890
H	4.17171563175573	11.15893345421776	7.56621931416496
H	5.72916359050294	11.54201276327856	8.31788063648582
H	4.60862278381035	10.44048138000285	9.13416599594618
C	6.24037597707215	10.10894755143748	6.07448656574008
H	6.78821034615720	9.33977703924241	5.52674630220184
H	6.92525640564159	10.94613824177112	6.26118127877941
H	5.42121253913406	10.47146499237154	5.44103229039021
C	6.85793798298463	9.06807614591668	8.30815210701038
H	6.47913017618116	8.69539067380026	9.26829058298952
H	7.55392464746195	9.89230357068796	8.51192442066178
H	7.41679291560772	8.26760908946778	7.81909794998995
C	0.98542898815283	7.44985694541908	7.74783095699006
C	0.07240662656964	7.39382286273713	6.49410293604886
H	-0.98415796083623	7.44300963075160	6.78829944623377
H	0.22302073576734	6.46818067552968	5.92777190092709
H	0.28419742998001	8.23637079723911	5.82487310775396
C	0.65681017719672	8.74053046189277	8.52853733595914
H	1.26399054521690	8.82922717453071	9.43740091551640
H	-0.39736575420358	8.72759301352780	8.83055879307329
H	0.81662721127812	9.63740057079231	7.91817071240267
C	0.67336940336604	6.23848529860259	8.66543545772996
H	1.30540149899726	6.25806130567155	9.56193496516213
H	0.84737690503404	5.28662925511883	8.15180608471798
H	-0.37672097683477	6.26251884837873	8.98473306732906
N	8.55955357739024	5.71708182549112	5.04006205663410

N	4.84902276016428	5.12232804072557	5.49912487907893
O	6.23251647070298	6.39086813069769	3.13676461494630
O	7.06202963297400	3.77129793840612	4.54401061635168
O	6.42041581727389	7.05899471446917	6.03822216599327
Re	6.58137519674070	5.67197532089568	4.65831821662952

List S28. $[Re(O)(ap)_2]^-$, BLYP

C	8.35583910495621	3.35358438944937	4.94014956079264
C	9.22799645016995	4.44429231131453	5.15361106314934
C	10.60012504728706	4.22023986073763	5.28925856555948
H	11.26161736263432	5.07000000131887	5.42696069895970
C	11.11570336368418	2.91357856046203	5.23790623211351
C	10.22302530597498	1.84971176624049	5.04756156889422
H	10.61062096795822	0.84050143118215	5.01147609521410
C	8.83239129601190	2.03402896795053	4.89211633885640
C	9.22245583348785	6.83870133915951	5.64592684646419
C	9.16379946891719	8.03771500692160	4.91539880388393
H	8.62982717686049	8.03858088617856	3.96861198549304
C	9.75395792493972	9.20227909525225	5.40914508280366
H	9.68302625980059	10.12381240002525	4.83375134210342
C	10.42374929191917	9.19335997506424	6.63757649343225
H	10.87776749515706	10.10346087246326	7.02510848211955
C	10.48625112706244	8.00247424357746	7.37253118039097
H	10.98415669809560	7.98565483807876	8.34119492059403
C	9.89132435398773	6.83768234720933	6.88549869386296
H	9.92200938255507	5.92015843618041	7.46724249462462
C	5.07127178882926	7.34796051507628	6.30536944585259
C	4.65241708398912	8.49409839549963	6.99920498641944
C	3.32154223631174	8.50692620247743	7.46909916970283
H	2.97812205898747	9.38008253678830	8.00755135969234
C	2.43111035252284	7.44425743661895	7.26328370276111
C	2.88646233925524	6.31334174524956	6.56408108023793
H	2.22181602711513	5.47563593867212	6.37748954129181
C	4.20139975323578	6.25427596772605	6.09665870650626
C	4.18441350391445	3.93425676642153	5.32742500799598
C	4.06027373224766	3.26009941288547	4.10084662019449
H	4.42035178101975	3.75584938247179	3.20301515384347
C	3.51345093033872	1.97699981070471	4.04530910622446
H	3.44083003889926	1.46690854105986	3.08621992830307
C	3.07019413360295	1.34318218404990	5.21135845897864
H	2.64999259302902	0.34008437933976	5.16829301569669
C	3.19153344490303	2.00784374204253	6.43844014080329
H	2.87231682711205	1.51737688558176	7.35714713669881
C	3.74383157028615	3.28803399140572	6.49876375871847
H	3.85857207524650	3.79334797250645	7.45405876826848
C	7.86503290059799	0.85554997041125	4.67224127090399

C	7.14408171625801	1.02531428037813	3.30829647168137
H	6.58596434593151	1.96273522095264	3.27480707824703
H	6.44151796745723	0.19605694917795	3.14384563070257
H	7.87624668890806	1.02459791418270	2.49002825420145
C	6.82058957335765	0.81809066120278	5.81882368347041
H	7.32309704931296	0.66402544124897	6.78304806036058
H	6.11129463596587	-0.00631299342552	5.66399035260734
H	6.25335226479839	1.74874351148486	5.86874376514631
C	8.59539905154149	-0.50577117567140	4.65272346843922
H	9.33293607287402	-0.56201795793744	3.84202273507328
H	7.86138111538037	-1.30668183171759	4.49421963385875
H	9.11037391046669	-0.70628359886785	5.60085138586014
C	12.63655281328959	2.70572661509154	5.38411474896510
C	13.37340192944584	3.45658180798275	4.24344489877738
H	14.46173774259448	3.32459229864214	4.32993518327956
H	13.15740252398010	4.53010325455795	4.26785451238224
H	13.05448662316591	3.07397614162622	3.26597390182406
C	13.10924388720411	3.26707998471898	6.75085166501303
H	12.60665432962889	2.74370996685677	7.57387524839496
H	12.88082679063619	4.33432989809512	6.84506785623540
H	14.19485361615587	3.13834059136292	6.86957905466377
C	13.04092444348464	1.21723883886372	5.31313694190622
H	12.56993634334499	0.63335725037053	6.11325073585483
H	14.12962436786271	1.12297747024141	5.42213424352349
H	12.75811747735245	0.77007678652387	4.35256872065800
C	5.61708735259339	9.67298773108611	7.22655180541811
C	4.94878906789397	10.83668488160949	7.99186930301228
H	4.08721849685647	11.24137712122047	7.44577438015893
H	5.67679095710049	11.64825817947210	8.12091100757888
H	4.61023118897529	10.52958979998231	8.98956147550187
C	6.09838520565114	10.22235933469749	5.85710707619521
H	6.60540288376930	9.44547906668634	5.28224156381680
H	6.79806754019774	11.05648339539325	6.00837676510785
H	5.24410284910684	10.59006566138437	5.27342819464154
C	6.83709561513004	9.19465662830302	8.05746558466924
H	6.50610473457657	8.82163141605139	9.03583714905905
H	7.53531350725537	10.02627413088799	8.22454976019307
H	7.37736577358171	8.39587244836541	7.54647526543682
C	0.97732381426693	7.46701811285073	7.77680179357544
C	-0.00122816902602	7.33227293696149	6.58062532383406
H	-1.04427276036276	7.33884379173450	6.92907005473766
H	0.16645733072268	6.40077181672002	6.02912672442512
H	0.13413219760038	8.16437443467428	5.87846064303416
C	0.63462006832431	8.77088395171613	8.52964621636262
H	1.27549716830925	8.90602590429229	9.40926871123534
H	-0.40802447242562	8.73809283413200	8.87283209059207

H	0.74998615494559	9.65055686226835	7.88497468091216
C	0.75559238906439	6.28003698787667	8.75154610239824
H	1.42998600156929	6.36328384940755	9.61298983752458
H	0.95396801847182	5.31979421598214	8.26320472292987
H	-0.27987385163303	6.26812963252349	9.12177845472541
N	8.56599995015146	5.68783517810871	5.14810790667084
N	4.79608046164410	5.21042015761746	5.36115210534552
O	6.47343297515569	6.06928556504285	2.93307307368303
O	7.03650556310698	3.67862825154107	4.79304759190165
O	6.33042604181412	7.20067963547262	5.79464901888247
Re	6.62079958230979	5.62788025424006	4.58760457690698

List S29. [(ap)(isq•)Re(O)(μ-O)Re(O)(ap)(isq•)], BLYP

C	5.34636497574119	-2.02465176062581	7.34100391830292
C	5.14387253863091	-1.93991484524196	5.95545714670600
H	5.74157462405135	-1.25889844650579	5.36095081643411
C	4.16882548208765	-2.72980275399452	5.34658965154907
H	4.01640409637234	-2.64995118500928	4.27290540404937
C	3.40237764475111	-3.62016033448317	6.10608107809332
H	2.64853194000811	-4.24068968030218	5.62662936992633
C	3.61470862782163	-3.71362676172156	7.48619442126046
H	3.03184331372790	-4.41170366552985	8.08349458621823
C	4.57843155470222	-2.91650024476387	8.10628379370563
H	4.75389976457800	-2.99300504979708	9.17313014803289
C	7.08389338738966	0.47487091260059	9.38439324239234
C	5.99575424246528	-0.34691404870492	8.95658817464548
C	4.72128204740458	-0.19750762956814	9.54394421155362
H	3.89863238985486	-0.79507524409659	9.18020711082132
C	4.54107540757691	0.72044973437365	10.56394199786594
C	5.64314752969619	1.52709086554273	10.96248623579085
H	5.47236497489815	2.25710689878300	11.74482811310083
C	6.90845163629375	1.46070760139785	10.39232259262729
C	3.18957028415311	0.90368484549998	11.27445542178864
C	2.09263010252721	-0.00956190124784	10.68731993184429
H	2.35404794647288	-1.07034943776048	10.78162278202718
H	1.90947787813352	0.20565846464128	9.62789031008176
H	1.15217259328008	0.15188426256439	11.22737532480927
C	2.71865723794226	2.37505082203595	11.13755561040912
H	2.59270591079423	2.64469893750254	10.08286812388178
H	3.43549247503398	3.07601319343789	11.57861314778398
H	1.75574177362853	2.51521726161051	11.64515456902042
C	3.36420293838705	0.55371728621672	12.77635233107475
H	3.70163196338109	-0.48324586675479	12.89351424411789
H	2.41134390537355	0.67055938265609	13.30864952147665
H	4.10271478017566	1.20384348666591	13.25857382222652
C	8.04502368369656	2.42623710169028	10.76380756552990

C	7.61971721356223	3.41364629626301	11.87121747933693
H	7.34092800577719	2.89523146047232	12.79729073452830
H	6.77638906848271	4.04136106606912	11.55835969833443
H	8.45950095625727	4.07938107031037	12.10300559872662
C	9.28721420648139	1.64843984125911	11.27021192696703
H	9.67018280914238	0.96920397264647	10.50551620425287
H	9.04218220708372	1.06781746740761	12.16787333489567
H	10.08403882064796	2.35653620112228	11.53089041495921
C	8.43223996943884	3.24908615278554	9.50595188234903
H	8.77381812753959	2.60123107285769	8.69595584414345
H	9.24322874762722	3.94531537453099	9.75333942678871
H	7.57732626993690	3.83080615742751	9.14413625040088
C	8.30296168651832	-2.60407224137439	10.09794266357423
C	8.52862821185181	-1.88700409262420	11.28222869595434
H	9.31944914176986	-1.14613809130851	11.30266419549990
C	7.72469516241899	-2.11179535927105	12.40058523981597
H	7.89061543231500	-1.53490642142320	13.30788734363225
C	6.70106856082169	-3.06494140168521	12.35138851482122
H	6.07240607994796	-3.23674092781019	13.22222602611558
C	6.49100715383856	-3.79700155552474	11.17706244598699
H	5.70549320381310	-4.54913147173646	11.13731430368608
C	7.28510341363447	-3.56654003485505	10.05146396127022
H	7.12368537384652	-4.11433751499974	9.12753695818859
C	10.44285965278901	-2.54362726279638	8.94232749402808
C	11.16587345290057	-3.29247639278104	9.87533976671259
H	10.63262070634196	-3.79695331191610	10.67474160009396
C	12.55916790065618	-3.38750763383239	9.75709107192593
C	13.19306884027876	-2.71928124274094	8.69349717278923
H	14.26723071681648	-2.79885870427585	8.60164963020490
C	12.50507257361181	-1.94824086338410	7.73839658299712
C	11.11177556177873	-1.87882824014876	7.88922240588346
C	13.34315982622828	-4.21216991205598	10.79665673366546
C	13.12242311525123	-3.59647315252372	12.20399037956471
H	12.06236670893456	-3.59602381259846	12.48101719117221
H	13.47820320394585	-2.55920784731615	12.23109554271417
H	13.67105552465192	-4.16902220485891	12.96361614657819
C	12.83002926339086	-5.67583837453727	10.79084861590280
H	11.76424140283614	-5.73178946235026	11.03767978351529
H	13.37929661475806	-6.27605990470214	11.52815008251375
H	12.96987411493968	-6.13150377798371	9.80296448989816
C	14.86049921849935	-4.23589670423390	10.51421702807255
H	15.29613935896064	-3.22979327531566	10.54089217736991
H	15.08418698613785	-4.68120718886688	9.53731799196606
H	15.36601038395553	-4.83799284186536	11.27911190573915
C	13.21617474102242	-1.22153919161944	6.58449327837531
C	12.94593479956737	0.30238447419692	6.68610181941039

H	11.87899028711784	0.52188545611579	6.61165688755313
H	13.45732699440034	0.83491348167550	5.87688097370670
H	13.31320560334511	0.69560440416065	7.64251507129023
C	12.68066287186961	-1.75503269031329	5.22965562705905
H	12.84484103239843	-2.83622041977009	5.14568207661995
H	13.19981171781080	-1.26094650161983	4.40007998448259
H	11.61197695935195	-1.56101043228282	5.11992761282911
C	14.74350740507455	-1.44372877462628	6.61630858326901
H	15.00499260821004	-2.50416433642142	6.51524509385899
H	15.19303638453447	-1.06407514946603	7.54201516942008
H	15.20490277937325	-0.90713623316977	5.77919119797779
C	9.75980060977135	-0.91554073219673	2.63854145520095
C	9.43786059063265	-2.12084431665277	3.27994151236070
H	9.14688566080573	-2.11719312048141	4.32375088487421
C	9.49753233342124	-3.32167115710560	2.57294770290067
H	9.25207046788575	-4.24993636414453	3.08335636118338
C	9.85970051569693	-3.33084801384822	1.22178227792951
H	9.89739852320603	-4.26827110188516	0.67133274906378
C	10.16749695070624	-2.12616062981456	0.57922932004521
H	10.43900890184601	-2.12176386520917	-0.47436328411456
C	10.12422077184229	-0.92118717606243	1.28286130571131
H	10.35446439646926	0.01486583688013	0.78716216256429
C	10.80668164600537	1.09731976495132	3.47388209250192
C	12.05739351094185	0.89018168891615	2.85352274447565
H	12.20181022833894	0.01654511308744	2.23539097910225
C	13.08134955282260	1.79913957096372	3.05648158147989
C	12.85825418679429	2.89722856139111	3.93351581857083
H	13.68426731263039	3.57747914059816	4.10422027936669
C	11.66456857008342	3.13383108876269	4.60423909770386
C	10.60964836121175	2.22106085728357	4.33435900825284
C	14.45515041443898	1.65171063166924	2.38111822101040
C	14.51691612927129	0.42291015004383	1.44898330176283
H	15.50674550311715	0.36527287858196	0.98084649362409
H	13.77022651855743	0.48661806520418	0.64847134870640
H	14.35389840781578	-0.51259700606513	1.99714972408892
C	15.55418261945449	1.49543105685885	3.46445969265281
H	16.54326280239252	1.41690952606721	2.99519477431718
H	15.38388865864841	0.59020328839959	4.05785819892541
H	15.57327533722959	2.34823071393626	4.15163283376108
C	14.74125121400380	2.91922440197304	1.53317840223529
H	15.71158321567735	2.82710873341304	1.02841180283414
H	14.76901066007354	3.82315767101196	2.15150502982138
H	13.96605687591217	3.05731092333231	0.76960644429416
C	11.48993131263135	4.26683134315784	5.62836963511720
C	11.17294035532815	3.64466593902203	7.01410751250077
H	10.26421984872445	3.04007672088180	6.98028756947507

H	11.02794292321796	4.44045877042488	7.75540193214530
H	11.99764601883546	3.00718083061329	7.35053265654888
C	10.33124520782601	5.20852783842238	5.21075309308016
H	10.53205734805288	5.65797705669185	4.23086672698229
H	10.23041173706665	6.01999328422569	5.94292049698082
H	9.38156139886897	4.67118603347252	5.16248310920252
C	12.77025341379098	5.11806878779232	5.76541903884915
H	13.04417351383000	5.60067457459705	4.81883546553715
H	13.62289958071300	4.52013383857384	6.10940732395966
H	12.60021527837654	5.90943183582661	6.50497128819779
C	8.24212175347876	2.86180066338871	1.99797353847285
C	8.86024867509774	4.11659700895320	2.10430782237636
H	8.65557996122106	4.73001084624394	2.97446583408574
C	9.74465409590263	4.54742424566072	1.11451899346607
H	10.23754086681897	5.51203337701412	1.21538382599373
C	10.00777185463910	3.73719165794422	0.00406806233391
H	10.70047069982172	4.07188917442476	-0.76482380988315
C	9.37442990559882	2.49437428074000	-0.11286438447336
H	9.56483284070787	1.86511855333003	-0.98006011555717
C	8.49694117916464	2.05478627695687	0.88036510450062
H	8.00971745057876	1.08650363722102	0.81270362041352
C	6.23927834400408	3.08411428487293	3.36200436128927
C	5.61193256263123	4.07057005333916	2.58617652752962
H	6.02739639318103	4.31733085206511	1.61668291522492
C	4.46509654665173	4.70041590443838	3.07419187297167
C	3.97239529037219	4.32265459390860	4.34252702280461
H	3.07853704944883	4.81397747036347	4.70746085100737
C	4.57274801170855	3.35042994547404	5.15428548254489
C	5.72371523707092	2.73411053809813	4.62811611578267
C	4.01776592257336	2.95256009635659	6.53189950897645
C	3.64348895990255	1.44725182439598	6.51829031103842
H	2.90001564388049	1.23857526463752	5.73932425514223
H	3.21931134642735	1.15655338414229	7.48647082948394
H	4.51973721791426	0.82347165228391	6.33355443834404
C	5.08990082033061	3.21209790361729	7.62251705689428
H	5.98642988580488	2.61409486987670	7.44966837710085
H	4.69466873788508	2.95101346337115	8.61037736670053
H	5.37672855261666	4.27118662286907	7.63467100900385
C	2.75374083420101	3.75774152134200	6.90036376454060
H	1.94054681498129	3.59494563796748	6.18251773401940
H	2.95658479788354	4.83433890510910	6.95572732397746
H	2.39487811133131	3.43622879033254	7.88523547534332
C	3.72223330099403	5.78561338614755	2.27170718697644
C	2.27266579112798	5.31360650744711	1.98513555560728
H	1.71719551664642	5.13269364528863	2.91192919754147
H	2.27615520679930	4.38077761861206	1.40825477083725

H	1.72989729625299	6.07396478332270	1.40814341406636
C	3.68163162149045	7.09909461781300	3.09673292766229
H	3.16815216658849	6.95881267727306	4.05400944890180
H	3.15181016755319	7.88306381980052	2.53976513384503
H	4.69779230011735	7.45383102140472	3.30826223495774
C	4.40722772210380	6.08787497278364	0.92200383067961
H	5.43611273268672	6.44121236355939	1.06073400003198
H	3.85262027202722	6.87358681168296	0.39452720737247
H	4.43270081483570	5.20480134991273	0.27224178912677
N	6.35289525854740	-1.20218285397819	7.93910962952828
N	9.06699124367289	-2.33812957633128	8.92709453766627
N	9.68810963029973	0.30054767373204	3.38811774752646
N	7.38920973403006	2.37721058831068	3.02791112025294
O	8.21888326320129	0.24163668686384	8.76060733438826
O	10.29427839759750	-1.14703034187587	7.08548884450710
O	9.40026640115715	2.30660914430645	4.84734800649367
O	6.45591450138035	1.80051552804988	5.29118242216561
O	8.27838889063292	0.10614499213881	6.03238879499405
O	7.92103848147652	-2.62813770078216	6.36765616683006
O	7.14185576499968	-0.37970635737426	3.53888594968194
Re	8.34081674318760	-1.26313908706125	7.35045420484860
Re	7.95428224626564	0.92219019722486	4.34596025373659

List S30. O_2, PBE

O	0.00000000000000	0.00000000000000	-0.10949650494540
O	0.00000000000000	0.00000000000000	1.10949650494540

List S31. [Re(O)$_2$(ap)(isq$^{•}$)], PBE

Re	7.25387392635943	4.54755799803091	15.95621512396821
O	8.30640879586555	3.59920566681139	14.43207635910905
O	6.24518553965389	2.72753097421689	15.93876166523980
O	8.51888112104575	5.71089755536140	16.12705100954274
O	5.95976023162672	5.27902148215231	16.83547976335426
N	6.17693720629380	4.90611999561885	14.24023612717658
N	8.33542480992618	3.30866239026279	17.19242783421809
C	4.90494371705062	5.54382627027988	14.26850943913123
C	3.74444418661313	4.80091176040033	14.01162500356262
H	3.83090315967245	3.73582226439840	13.79710520208309
C	7.76741156079448	3.51931654361180	13.24074435045133
C	2.49905938142800	5.42263725991987	14.06171781398714
H	1.59745341520326	4.83764751079676	13.87631403622327
C	6.79222750245442	1.69023336700714	16.52187821364139
C	7.96535336054441	1.98733562722412	17.27831840225199
C	5.92959853500794	4.34763437808755	11.83267565486487
H	5.03767292845182	4.95841419192151	11.72671331462248
C	8.11369686024651	-0.31487032907062	17.97975273327014

C	6.97279849031661	-0.59123196168851	17.18035142495993
H	6.61259539464799	-1.61789197652363	17.15086998877894
C	6.57027791549799	4.27996972851459	13.08150749888371
C	5.04924315288606	0.05122584946119	15.60125038972790
C	2.40353028421053	6.78400295608963	14.36151586559700
H	1.42737591755345	7.26816511800382	14.40057245169947
C	6.28470644122077	0.36695151631130	16.44674418519305
C	10.77121277774278	3.11458902721790	17.30296634402573
H	10.72790960264082	2.35716360550005	16.52040236477500
C	9.58196346562482	3.70370495276630	17.75376042694303
C	8.60258033423372	0.98368431795837	18.02791302481460
H	9.47149165181327	1.23975068950763	18.62760217274162
C	12.03315626486897	4.50970172679654	18.82465532073130
H	12.98976905023053	4.82664236898891	19.24086584047843
C	4.68346637174971	-1.43756826667332	15.66329067967706
H	5.49335183338973	-2.07449044390724	15.27911437708073
H	3.79510570770989	-1.61813205591437	15.04235486883697
H	4.44474212225266	-1.75884406362220	16.68737529226378
C	8.76843390787086	-1.46181714621886	18.75264859874233
C	9.62229542169307	4.70224718455941	18.73173954610470
H	8.69045912301593	5.16143921924648	19.06020227187553
C	3.56121232781447	7.51918181809884	14.62311122848737
H	3.49275352583133	8.57995849752062	14.86607922769127
C	5.77968473199351	3.62825161456876	9.38557593149837
C	3.84506924392152	0.86577847654664	16.12039879666166
H	3.61657244862144	0.59872315120802	17.16190796109844
H	2.95649320752558	0.64724113129600	15.50939918437235
H	4.04034529586354	1.94312210582414	16.07655955890871
C	6.44082741063613	3.62178351913193	10.76554073453178
C	10.84688214246556	5.09797768276826	19.26725278606926
H	10.87327367765571	5.87535707536628	20.03142257761000
C	8.30123863990480	2.76135150267397	12.16595105162192
C	4.81092884527181	6.90295044694024	14.58294116665227
H	5.72091926809536	7.46277085980699	14.79659516168072
C	11.99132540158423	3.52148070153035	17.83780022445817
H	12.91548768598449	3.07125166600435	17.47354271091544
C	7.61037983397414	2.84047730928108	10.96303689627617
H	7.98835431064124	2.27318489799897	10.11456473864332
C	5.32380359997373	0.41305218388713	14.12562440937696
H	5.57686808687857	1.47265788887862	14.00736274810072
H	4.43201768788537	0.19956013376018	13.51766583820444
H	6.15648690129904	-0.18380336307638	13.72793635470399
C	9.25490250636314	-2.53225634493599	17.75395178525583
H	8.42838511720825	-2.92880052956452	17.14941355463234
H	9.71195085667837	-3.37384197517664	18.29501408085387
H	10.00495320027755	-2.11485006526614	17.06757590268318

C	10.74527198819791	2.83986629213679	12.73211824292118
H	10.94054633756349	3.56859828242795	11.93257029313230
H	11.65531609249721	2.23751467461080	12.87258502587789
H	10.54735021968909	3.39095114928844	13.65872210811904
C	9.56501880127743	1.91993180880451	12.35458806808504
C	9.96994963288486	-0.99161153209886	19.58238883364365
H	10.75788449024198	-0.55790382826911	18.95038096083949
H	10.40490641268396	-1.84675964096890	20.11812606415010
H	9.67725470423417	-0.24175849504109	20.33066066958537
C	9.33975525867620	0.87999192809454	13.47349338694091
H	9.10746316107735	1.35960506975245	14.43085199093277
H	10.24641282298817	0.27011224103023	13.60091725999802
H	8.50987527786422	0.20682952033885	13.21721937872618
C	4.52546731281568	4.51082505340259	9.34729500864285
H	3.76277385576109	4.16562251632638	10.05960143914922
H	4.08261379346878	4.47811051067009	8.34211256284813
H	4.75932360006923	5.56028643141719	9.57494322948977
C	7.73381017201710	-2.09048312404437	19.70926876592010
H	7.37151442129236	-1.34878437655021	20.43454376921226
H	8.18941888007860	-2.92345020108610	20.26460361563948
H	6.86430183655025	-2.48382182917781	19.16622639783821
C	6.78151637038190	4.16269674886630	8.34154430391757
H	7.08722771658573	5.18958870513912	8.58553187970139
H	6.32061763287071	4.16801329197071	7.34279434197773
H	7.68741797850956	3.54403771098287	8.29125668892567
C	9.93862743156164	1.16237939521813	11.07336442955715
H	9.14552876539659	0.46639647806433	10.76377228009124
H	10.84814837003919	0.57282334369095	11.25395780744606
H	10.14633087619762	1.84683692235924	10.23844598323321
C	5.37105810586645	2.18805993152085	9.01333275392699
H	6.23637147271799	1.51240462408981	8.98946496291884
H	4.90259345251981	2.17273616600354	8.01822363781823
H	4.65027933166536	1.78736459255255	9.73986926717451

List S32. $[Re(O)_2(ap)_2]^{-}$, PBE

Re	7.26504919642335	4.51631810118581	15.93678958543273
O	8.32670204362031	3.59630048123352	14.40984335570286
O	6.26604238834498	2.69785749678842	15.89415997810854
O	8.53408694429650	5.68471786629083	16.13007922265784
O	5.95768843408474	5.24332760318574	16.81717563375215
N	6.21656367880762	4.93969290794962	14.23085336273551
N	8.30772911369212	3.31011051194708	17.21791407562594
C	4.92596465873869	5.52397484411505	14.27093830087578
C	3.78085171103403	4.75409037196077	14.01593246129873
H	3.89889674916067	3.69376445019136	13.79331793122154
C	7.78792556920536	3.54749938784736	13.20238698239247

C	2.51608166034401	5.33570392440373	14.07327168095394
H	1.63284838832005	4.72181165204136	13.88707303667438
C	6.79078497247427	1.66673034051653	16.53571066611999
C	7.93656936257561	1.96920754436562	17.30659696495767
C	5.95468058582951	4.37396461632366	11.81271488478557
H	5.05701933018832	4.98052502157375	11.72180903097258
C	8.06930028778001	-0.33080589226875	18.05318843840989
C	6.94803154025981	-0.61401285681863	17.25314424112155
H	6.57514385515781	-1.63756804436656	17.23114895324951
C	6.60177161880282	4.30262151149734	13.05281320351440
C	5.06614491448255	0.02523660145211	15.61383130475334
C	2.37526179683813	6.69145372826257	14.38363514142586
H	1.38378836578955	7.14477642636150	14.43053525151505
C	6.28348676664043	0.34966974851427	16.48526228563136
C	10.74491223186212	3.05502154380514	17.27043349788031
H	10.66318240589361	2.28846391793987	16.49977631306351
C	9.57535098915881	3.67643575564123	17.73441936937392
C	8.55855024993944	0.97811181115619	18.07646548420781
H	9.42154546606186	1.24534566258648	18.68164175074213
C	12.08323072995919	4.43246322318687	18.74580119169798
H	13.05794648704189	4.72862943666658	19.13682326279817
C	4.66703063141714	-1.45396839848061	15.70603406097657
H	5.47526338270203	-2.11690653688632	15.36375884671506
H	3.79161814945380	-1.63406323501784	15.06460962178655
H	4.39671026272010	-1.74071628603710	16.73311679421445
C	8.72246613019840	-1.46076291095435	18.85949094209952
C	9.67452118631893	4.68394903521141	18.70182856260817
H	8.76148367657725	5.17379481172883	19.03898477990315
C	3.51324280620966	7.45714812219614	14.64620930591225
H	3.41416390616218	8.51398166359734	14.90054226781667
C	5.77701094232031	3.68261030730051	9.34787117081700
C	3.86022052955744	0.87422780283968	16.06834981345601
H	3.58771480812978	0.63275163360585	17.10645123522189
H	2.98863714364766	0.66682691343200	15.42692231566728
H	4.09044989673877	1.94431502546081	16.01326261367457
C	6.46020078687110	3.66267849430091	10.72144412647927
C	10.92098695839597	5.05752284665896	19.20287273825601
H	10.98410138238457	5.84682960625501	19.95415217756553
C	8.31592032119217	2.81399399820057	12.11707500201316
C	4.78112346643158	6.87922016827811	14.59129790855946
H	5.67636425274104	7.46127059491325	14.80859494476857
C	11.98821292906782	3.43252363539178	17.77350910600459
H	12.89119623609355	2.95095280663724	17.39355889528700
C	7.62582711178638	2.89675436092861	10.90160242171250
H	8.01048980037159	2.33564361127803	10.05051234930818
C	5.39037504987376	0.34020167987262	14.13831098568049

H	5.68512029529806	1.38778690466467	14.00812771040325
H	4.51024899655877	0.14246971366342	13.50593345947487
H	6.21675272863477	-0.29148934565462	13.78223876257600
C	9.22812369792705	-2.55514246986783	17.89790505345441
H	8.40713378828612	-2.96469421295695	17.29432064406928
H	9.68694456467912	-3.38413287742099	18.46054128465767
H	9.97872166804899	-2.14632674472343	17.20664776078453
C	10.75448199012503	2.88097413273490	12.71850860004508
H	10.96182276204559	3.62617565230779	11.93629688172898
H	11.66481506106100	2.27869519317055	12.86889086342389
H	10.52754248200891	3.41273028476551	13.64984896713108
C	9.58149382556918	1.97052697968073	12.29939998326121
C	9.91442993198110	-0.96849357087342	19.69129540835674
H	10.70366557068258	-0.54155631094275	19.05641160690772
H	10.34913318276319	-1.81027516955706	20.25104321734424
H	9.61006269380902	-0.20080016180583	20.41667238552702
C	9.34212063436996	0.90502672756572	13.38986419542215
H	9.06047720040897	1.36715525284711	14.34277680865734
H	10.25561389413009	0.30870457303207	13.54316365593237
H	8.53346229997885	0.22273971575769	13.09146665265225
C	4.51812202294314	4.56016946096341	9.33694238228139
H	3.77118135242541	4.20526509824559	10.06103108393933
H	4.05823047741110	4.53727276563330	8.33748146180583
H	4.75299920889979	5.60670980768380	9.57713981855983
C	7.68871356458635	-2.07740910370171	19.82394188682944
H	7.31975835002126	-1.31951888953537	20.52940483832869
H	8.13872906037843	-2.90144130156527	20.40059809377981
H	6.82255899578250	-2.47593005218548	19.27895579115684
C	6.75352074571595	4.23354907935816	8.28927551553059
H	7.05524995967745	5.25997389152068	8.54192222746267
H	6.28264376100035	4.24465476820704	7.29308004156883
H	7.66455212176783	3.62312814831754	8.22950737223151
C	9.98185214102016	1.24443327212520	11.00764297259741
H	9.19628545796031	0.55289663879428	10.66925372497310
H	10.89271838194724	0.65504747703333	11.19004195233387
H	10.19571403993610	1.95061312377751	10.19171844601765
C	5.36384688079482	2.24900152511161	8.95803973689873
H	6.23298784035565	1.57853778376757	8.92318472874635
H	4.88199099062759	2.23836539952134	7.96708173560005
H	4.65647713820997	1.83912539429206	9.69285745738920

List S33. [Re(O)(ap)(isq•)], PBE

C	8.34209195511519	3.40608460605688	4.82198964799143
C	9.20292814734326	4.48561551802729	5.11358148364244
C	10.55914196001796	4.26186429121316	5.35316751889312
H	11.21621584169814	5.10753258586091	5.54814103859725

C	11.05598498634406	2.95516997844790	5.32482232809867
C	10.16712462689286	1.90214192604379	5.05194605964912
H	10.55511686661299	0.88830940276136	5.03977409689966
C	8.79989930541277	2.08153466813484	4.78798596094381
C	9.22830598570338	6.91981424278915	5.36969217268600
C	9.21667430549888	7.98111284976330	4.45817326794298
H	8.71543611512087	7.84950517786452	3.49862124432951
C	9.82905539754250	9.19016283571586	4.78680367527514
H	9.81054776833543	10.01369520630027	4.07231333093905
C	10.45692099237438	9.34849511210479	6.02323620349201
H	10.93160447547273	10.29585490456971	6.27975589564788
C	10.46618582230728	8.28967861505536	6.93461464751611
H	10.94048072655476	8.41073762413225	7.90926945054153
C	9.85252359879907	7.08035232737454	6.61516158414337
H	9.83380908451946	6.25922174335258	7.33162893963499
C	5.14050050758965	7.24962415945635	6.46801902241678
C	4.72153065317978	8.40305445707355	7.14733951697799
C	3.37725725813155	8.42384084563343	7.54964681165068
H	3.02051192349988	9.30054266252328	8.08189592550919
C	2.47142605603245	7.37972098558912	7.29734701104320
C	2.92536615420292	6.25047400343538	6.60947115866332
H	2.25442523313215	5.42287482611980	6.38397641298936
C	4.25808204257335	6.17812522822028	6.20507488989280
C	4.23676181299311	3.85081903228790	5.46908085611378
C	4.01687486065528	3.22297778240903	4.23706439801969
H	4.31978504531655	3.73549813029059	3.32346639258831
C	3.41755622933434	1.96512765811910	4.18975041338862
H	3.25585843032181	1.48255626819008	3.22527327523106
C	3.02311685902861	1.32829775852153	5.36741156937594
H	2.55301647485662	0.34537292197891	5.32853880414042
C	3.23892488503250	1.95502813873535	6.59801268681530
H	2.94403220500292	1.45873595264651	7.52335341051482
C	3.84507606779784	3.20793224570260	6.65281674625448
H	4.02683666196421	3.69777845085904	7.60967658070307
C	7.86046340819965	0.90785861302015	4.49642953743845
C	7.20339274292310	1.09381778187621	3.11318670972183
H	6.62084128070460	2.02007686786793	3.06225426276538
H	6.52596056718281	0.25171614889608	2.90728784790707
H	7.96714068218325	1.11891932662226	2.32289127906496
C	6.77620126214069	0.83962392885238	5.59088913951178
H	7.23501030777063	0.68345836512765	6.57765353442424
H	6.09537697596497	-0.00074126676347	5.39234654710263
H	6.17632715899086	1.75528653787715	5.62866538876108
C	8.60675305834107	-0.43393688823065	4.48541541018635
H	9.39073345398940	-0.46168299512421	3.71526694911038
H	7.89234750825458	-1.23903920909487	4.26381920033951

H	9.06688894612517	-0.65728767393017	5.45854463565815
C	12.54871315007533	2.72090233024137	5.58544355463678
C	13.37299993716141	3.46012733523104	4.51182234514060
H	14.44881446394190	3.31060066345784	4.68679976816168
H	13.17508871569306	4.54013890236570	4.52252498148360
H	13.13155656854440	3.08341671319431	3.50802733125766
C	12.91570202825420	3.27123022218930	6.97833588030358
H	12.34401373448501	2.75675871854468	7.76354202161279
H	12.70550833814356	4.34641946283349	7.05789241477659
H	13.98715148280270	3.12151357271808	7.17756092753456
C	12.92619184319876	1.23424512580819	5.54193727814682
H	12.38645960434404	0.65346273462554	6.30335720269494
H	14.00187575103453	1.12338973038476	5.73834448118541
H	12.72011425450628	0.79000395377363	4.55797287761010
C	5.68214003950539	9.56365818010997	7.42294203018071
C	4.98855642252509	10.71733069554688	8.16066753973405
H	4.15750128152054	11.13599214294110	7.57538378661766
H	5.71632048650578	11.52304643752108	8.33035797438306
H	4.60108802867178	10.40628048780991	9.14141625427165
C	6.22455983693589	10.11348967659287	6.08703411910494
H	6.77761627758967	9.34995546451431	5.52943509879520
H	6.90732581935792	10.95436101789653	6.27976322645965
H	5.40116363253655	10.47841636959369	5.45670843794647
C	6.85240412047238	9.07038475842263	8.29914343187965
H	6.48095702210706	8.69104917296477	9.26196608116630
H	7.54380088018584	9.90184304419556	8.50270164374498
H	7.41916003523912	8.27375409331474	7.80487122961166
C	1.00496395290241	7.44046038879183	7.74158611417686
C	0.09601529924496	7.37362740458070	6.49721550694786
H	-0.96240192433762	7.41467296440773	6.79452601499599
H	0.25293371498660	6.44530318252465	5.93193447130899
H	0.29860235919611	8.21701548399636	5.82256706064528
C	0.67716438868012	8.72727283382938	8.51054148273621
H	1.28527709098741	8.82210254299374	9.42120364157720
H	-0.37920555441881	8.71438086422557	8.81356328431902
H	0.83547902338506	9.62336140982848	7.89401354328738
C	0.70057451287335	6.23983328229695	8.66007829330556
H	1.32924249724333	6.26868109053192	9.56138934357330
H	0.88417205340201	5.28374212649814	8.15176501441007
H	-0.35332191056697	6.25792064029099	8.97521059595333
N	8.54668901462935	5.71443623156718	5.04800958643554
N	4.86270659988835	5.12168658824708	5.50638806318116
O	6.23403064249850	6.37301284831193	3.15869312595283
O	7.06011166658623	3.78100347166354	4.55259457162855
O	6.42315062284203	7.05277842399978	6.03465506477046
Re	6.58254751955651	5.66665255426491	4.67374843120814

List S34. [Re(O)(ap)$_2$]$^-$, PBE

C	8.34759911204048	3.35487322940054	4.95588205774957
C	9.21866035619596	4.44377728142068	5.16992578490244
C	10.59024347711119	4.22351986813436	5.29690931902568
H	11.25360679798116	5.07764341959952	5.43265543703811
C	11.10743757439919	2.92063919805001	5.23963220473652
C	10.21750200548316	1.85780144596866	5.05119962072678
H	10.60861210793746	0.84512317453367	5.01036676390914
C	8.82827146643441	2.03771974393708	4.90205319759262
C	9.21711813021968	6.82633229063382	5.65310913257318
C	9.14000796924233	8.02470500824587	4.92698917144588
H	8.57761361029605	8.02400639569334	3.99236496508755
C	9.74769046845273	9.18531350393070	5.40184237588890
H	9.66389214466609	10.10869769033118	4.82586323767156
C	10.45283109537848	9.17461334517435	6.60733596664237
H	10.92431129615435	10.08485982637806	6.98068698853270
C	10.53339291288571	7.98557074987630	7.33836831074662
H	11.06270550488090	7.96624133850224	8.29319157729623
C	9.92304107066706	6.82399020225679	6.86995925917337
H	9.97082065239044	5.90244754797782	7.45059359673224
C	5.08138738418372	7.33790195066615	6.31813947728754
C	4.65888988747801	8.48474650124066	7.00743148681765
C	3.32847677996473	8.49783408187902	7.47040496571854
H	2.98168956958610	9.37626067369672	8.00756565197460
C	2.43979997925749	7.43734916124276	7.26428615470245
C	2.89679054163735	6.30609964580427	6.57207365330663
H	2.22950402299256	5.46534311144160	6.38335847097026
C	4.21228534725965	6.24558634438544	6.11216943447816
C	4.19028943511650	3.93913671077424	5.34449681711379
C	4.08331572128236	3.26277643766246	4.11972485433285
H	4.47132095750799	3.75479845526302	3.22689862456682
C	3.51709147892118	1.99109798413397	4.05547718905696
H	3.45544234365076	1.47951001767117	3.09326262874569
C	3.03760318454697	1.36943943323651	5.21060336459279
H	2.59851453739488	0.37197368787682	5.16029701447488
C	3.14239178116042	2.03481563741895	6.43574029419586
H	2.79213710902257	1.55267917391489	7.35066357534460
C	3.71236277818006	3.30423191212993	6.50523834623389
H	3.81179603367436	3.81356285805195	7.46401289903834
C	7.87430527847942	0.85813692407004	4.67955410763059
C	7.16191999545116	1.02288848889283	3.32117452458137
H	6.59859808615643	1.96141755896352	3.28474546042462
H	6.46042404130987	0.18962629963042	3.15606114019770
H	7.89673691390847	1.02354311986403	2.50247853429797

C	6.83138242388039	0.81002886092225	5.81463127696925
H	7.33071217751654	0.66960609124215	6.78489036193064
H	6.13660053994501	-0.03005864517300	5.65938311952307
H	6.24394201144823	1.73335967681749	5.85654775243820
C	8.61201592660046	-0.48868523325900	4.66151524295293
H	9.35395970679760	-0.53897560971575	3.85098950600365
H	7.88276864868209	-1.29639433711089	4.49951236721827
H	9.12668237509093	-0.68675608065109	5.61329583321843
C	12.62202893399680	2.71678878747708	5.37729317959044
C	13.34649754366905	3.46968448972193	4.24313128267720
H	14.43817678041390	3.34652468165779	4.32863696347770
H	13.12090197315480	4.54396465884517	4.26880521791530
H	13.02946444546881	3.08557761070053	3.26311351561232
C	13.09640235505726	3.27203411276040	6.73518459529634
H	12.60281612319554	2.74099070430896	7.56171395401438
H	12.85937728517226	4.33979464167127	6.83553566225561
H	14.18600645031207	3.15149480207327	6.84591450571448
C	13.02402142706315	1.23782400630121	5.29923144707526
H	12.55647652644581	0.64949381638385	6.10164473630642
H	14.11562712551019	1.14336307026699	5.40207508020826
H	12.73533883943270	0.79305906410012	4.33632711674129
C	5.61124931046722	9.66419218551955	7.23541173472332
C	4.93674385577916	10.81589477509137	7.99460775631598
H	4.07188826848782	11.21599456139953	7.44526666330675
H	5.66107971035446	11.63377195282435	8.12455265568690
H	4.59762392950330	10.50672409206871	8.99431389330193
C	6.08735702435682	10.21270826058233	5.87450365206073
H	6.59797890723662	9.43473943661378	5.29729436415173
H	6.78760957442366	11.04929023756587	6.02728213026132
H	5.23153460968101	10.58046492833873	5.28919336420913
C	6.82701702330425	9.19908440337439	8.06296258243865
H	6.49958679711093	8.82048629132925	9.04271725821664
H	7.51769025235044	10.04003694536455	8.23180857585181
H	7.37928042749922	8.40460231302030	7.54955227337187
C	0.98945658223971	7.46425232267113	7.76487537525100
C	0.02679251323276	7.33982120337889	6.56675799634233
H	-1.02126096046839	7.34374556723230	6.90705738339167
H	0.20048347330897	6.40985650303646	6.00922912575627
H	0.16840544131213	8.17794180871653	5.86959693163771
C	0.65183063972406	8.75880357608587	8.51664599750646
H	1.29218980016516	8.88823684653247	9.40075449932350
H	-0.39427526607726	8.72909826090949	8.85688248495048
H	0.77337411389015	9.64276501947923	7.87454455214782
C	0.75536531732296	6.28041121550429	8.72503767617233
H	1.41926906826825	6.35675089779717	9.59808859964818
H	0.95904894178957	5.31970777843701	8.23340771464955

H	-0.28718506048592	6.26810427792324	9.08167310402139
N	8.55380762243023	5.67968325721759	5.17087020136630
N	4.81037164373248	5.20483562124623	5.38551480889966
O	6.47475384637364	6.05524729238663	2.97686293670493
O	7.03495225464215	3.68005226049944	4.81507508972739
O	6.33398374845720	7.18660778143851	5.81161782346528
Re	6.62230602979337	5.61778752711737	4.62006044044509

List S35. [(ap)(isq•)Re(O)(μ-O)Re(O)(ap)(isq•)], PBE

C	5.28483045608891	-2.13469060546326	7.51988930998081
C	4.88521896493273	-2.07862391392442	6.18035997021892
H	5.37872267114402	-1.38658903842289	5.49948096542311
C	3.86156248686958	-2.91151244376982	5.73440418513999
H	3.54703580001514	-2.86018616105724	4.69182889795420
C	3.25055476620601	-3.81147133343074	6.61090680565049
H	2.45642149933875	-4.46797671677152	6.25397388328379
C	3.66464323647365	-3.87370838312385	7.94316573680708
H	3.20209107993971	-4.58365341334390	8.62988030485988
C	4.67621342139454	-3.03292486601280	8.40404236677384
H	5.01467697302992	-3.08025235856515	9.43805749976448
C	7.15097400803443	0.37878429866748	9.38488294081573
C	6.02313622489289	-0.39368131711951	8.99029746180913
C	4.77049688972771	-0.20156132909663	9.60205559244459
H	3.91433428568567	-0.76912394754839	9.25068604827110
C	4.65187762342375	0.71984283183660	10.62962605510872
C	5.80425282082795	1.44136974979320	11.03402411805969
H	5.69432719619574	2.15025702978824	11.85309281779286
C	7.05782943986820	1.31503231658829	10.44504577115898
C	3.32421196348782	0.97631125733219	11.34794384531512
C	2.17615932782726	0.15403719138720	10.74899134076350
H	2.36370385942340	-0.92623807603196	10.82761565011476
H	2.01160854491183	0.39829856546890	9.68983992062713
H	1.24509927231580	0.37111311160441	11.29077803131999
C	2.95737895586219	2.46971618573180	11.24071242037471
H	2.83404888553830	2.76622755355551	10.18967641710952
H	3.72990967767289	3.11298182536620	11.68266719328335
H	2.01177345961901	2.66921499215240	11.76594246540708
C	3.47800774377396	0.59133887607764	12.83361985478125
H	3.73844905157210	-0.47200792923662	12.93320961324755
H	2.53672983762456	0.76867537048602	13.37463245855658
H	4.26540040861068	1.17913066109923	13.32465672560641
C	8.27830344939510	2.12253621764808	10.88858735560504
C	7.94242338098588	3.07156060391704	12.04631801848212
H	7.59500794496268	2.52845361819285	12.93735056070383
H	7.17234950301807	3.80298882897849	11.76187328998321
H	8.84506261756344	3.63088240654301	12.32860650635575

C	9.39324581196993	1.16376928569145	11.35735447974885
H	9.70854096753440	0.48842131620042	10.55336330634949
H	9.05438562125597	0.56027049478415	12.21234649905910
H	10.27044907559532	1.74255175487314	11.68180293807431
C	8.78792606832957	2.96904194596481	9.70491501701511
H	9.06884862706873	2.33986404832708	8.85359174167004
H	9.67230024100399	3.54696819711031	10.01125790584645
H	8.01574053232859	3.67606000881642	9.37121925046668
C	8.12149363345162	-3.17019954992023	9.74900453560620
C	7.78794525056844	-2.51792123716401	10.94387041027219
H	8.19862052711338	-1.52957508402411	11.14326055468681
C	6.93224653881548	-3.13451759435539	11.85671025663278
H	6.66090054875892	-2.61368141365204	12.77596089999696
C	6.42640752032102	-4.41088893389600	11.59678888584994
H	5.76143111274464	-4.89227589010426	12.31462707268373
C	6.77439643563949	-5.06776527752503	10.41392027342707
H	6.38002092033696	-6.06260132023563	10.20354293595555
C	7.61144017752255	-4.44744171628771	9.48616408244306
H	7.86900488445980	-4.93318830055717	8.54516983886556
C	10.33251062721967	-2.60156174529525	8.94349808177526
C	11.04413264157582	-3.27968132849788	9.93357984450983
H	10.50027951511469	-3.86293351458497	10.67608039266259
C	12.44248489072072	-3.19470091968003	9.95284585144237
C	13.08704219537125	-2.42348593446246	8.97341407815547
H	14.17055596679116	-2.35585503200538	8.99560763875622
C	12.40582255851431	-1.72326090641533	7.96172164598077
C	11.01179264880181	-1.84163708452558	7.96970426767141
C	13.21595120122973	-3.94404436349795	11.04507349720042
C	12.74385690468447	-3.45576754517368	12.42981218440635
H	11.67021435176663	-3.63340014388456	12.57558507237322
H	12.92525980897440	-2.37786183988113	12.54463774896110
H	13.28597724164291	-3.98513181324544	13.22762942026227
C	12.93908743326028	-5.45536626875011	10.91784052570162
H	11.86827346760112	-5.67853536988096	11.01610846518503
H	13.47529230804632	-6.01054511791141	11.70211441147349
H	13.27173268147258	-5.83139262660731	9.94007456868555
C	14.73119463303821	-3.72298014216210	10.94998750180950
H	14.99574021152210	-2.66167516292131	11.05986906202372
H	15.13728941961764	-4.08155349583550	9.99350649895723
H	15.23459684362479	-4.27788496473570	11.75434048069912
C	13.12513065664750	-0.87774907017321	6.90838834333907
C	12.63229100610389	0.58200606109557	6.99419791780119
H	11.55154898939902	0.65440505431498	6.82732777668267
H	13.13657155354681	1.19634533506683	6.23429885032067
H	12.85754944776635	1.00466481479347	7.98410371483193
C	12.83047624020693	-1.44826043387493	5.50600120077908

H	13.17796491711139	-2.48841433063161	5.42878368163744
H	13.34866493713811	-0.85298735846950	4.74044192972844
H	11.75814952060028	-1.42529137119578	5.28069458959682
C	14.64598807316889	-0.87519854029314	7.11226002408064
H	15.07628001243383	-1.88357630119085	7.03073773942350
H	14.92582648227167	-0.45600256449201	8.08920886058396
H	15.11116800795106	-0.25035243914261	6.33659651035296
C	9.82900939694856	-0.87443736337639	2.53075312453200
C	9.69022793602460	-2.15207271403519	3.08350721870722
H	9.45353769584294	-2.25959532528003	4.14120697989147
C	9.85568183140772	-3.27246089213929	2.27272860147268
H	9.75441851544891	-4.26667318218523	2.70839698729423
C	10.13800561422072	-3.12471101408022	0.91255163395649
H	10.25660878906560	-4.00505965407912	0.28010862729031
C	10.25981751055245	-1.84663859523478	0.36337008132401
H	10.46563286851901	-1.72279541851808	-0.70052447225860
C	10.11312441650927	-0.71876748112121	1.16914809391113
H	10.19485111044335	0.28221642191268	0.74782739599350
C	10.74002207593400	1.12662587535130	3.48652841788088
C	12.00946540836378	0.98658011603470	2.89568132818095
H	12.22114056033148	0.10781652478352	2.29441050467676
C	12.96722068171249	1.96537157322641	3.10610701742104
C	12.63133025574988	3.08391583863090	3.91073279103223
H	13.39011372370481	3.85185875101581	4.05180482260854
C	11.40404940173821	3.25918279251053	4.54134343287762
C	10.44701548194458	2.23645788757338	4.32719200758063
C	14.36510755300841	1.88813728480159	2.48681804993265
C	14.58474982649784	0.57973576129926	1.71702719395112
H	15.60401333354238	0.56028903474609	1.30678211251701
H	13.88544420493652	0.47794445500371	0.87494112024749
H	14.46880961364728	-0.29871207974032	2.36757242801878
C	15.43247548499557	1.98292484406203	3.59528935640036
H	16.44005945967317	1.95238881110624	3.15542818394137
H	15.34169821766497	1.14462061012788	4.30045565984582
H	15.34402440012580	2.91529643759318	4.16829975922894
C	14.53975898219967	3.06695206099327	1.50704675691864
H	15.53785402011988	3.03708767149529	1.04543653887291
H	14.43141738503648	4.03450925245237	2.01584440946611
H	13.78674119409334	3.01893607027502	0.70776991929620
C	11.07382831956589	4.47426999197092	5.40934046175914
C	10.69693268505162	4.00785202438745	6.82961009847329
H	9.81594398004468	3.35775909882820	6.81639575669562
H	10.47167045943321	4.87949483580319	7.46146007345493
H	11.52647371326093	3.45470460684307	7.29165035423738
C	9.89015655823852	5.24451428044887	4.78619032283047
H	10.15171003373607	5.61243106991606	3.78299044439663

H	9.64021016831841	6.11619208013916	5.40889154632919
H	8.99490341484443	4.61625992503856	4.70853133387675
C	12.26458906407742	5.43520198222052	5.52081740900514
H	12.56526269401256	5.83526763450888	4.54146039220235
H	13.13812801881489	4.95234236949748	5.98250837683194
H	11.98254623395703	6.28762117225706	6.15423744169459
C	7.92715099146547	2.23511717742606	1.62443271422957
C	8.97147116818566	3.15326865891957	1.44813331205023
H	9.28526499989523	3.77085494905156	2.28811664056587
C	9.60036260159188	3.26048271921121	0.20786062628591
H	10.42439682155992	3.96409541222141	0.08195936317364
C	9.17811356215572	2.47323112449561	-0.86668708688947
H	9.66894519438657	2.56291270699625	-1.83635922924640
C	8.12598417061600	1.57060806857380	-0.69432122207496
H	7.79423368838655	0.95049134870041	-1.52787575477390
C	7.50677586275478	1.44307651154559	0.54926027567979
H	6.70331884071078	0.72412694539271	0.70826106941653
C	6.31890804920606	2.98654770781860	3.26983133009068
C	5.81717969849542	4.05683114937452	2.52007453148031
H	6.19479440367059	4.22243987034451	1.51360946963333
C	4.85020610268464	4.88933980499186	3.08783916623571
C	4.40580685826295	4.62073366218573	4.39662736157808
H	3.65467650488060	5.27819332592432	4.82952844509042
C	4.88138842197499	3.55939151882884	5.17854256834086
C	5.85341657540340	2.74727593408383	4.57533753516786
C	4.38713354916286	3.28407904975952	6.60056640994015
C	3.74537253879999	1.88224774863574	6.65378107546348
H	2.88664724469961	1.82147737460900	5.97014548374350
H	3.39262039146295	1.66809550303603	7.67309846794792
H	4.46363210076145	1.10281813098751	6.37518298882649
C	5.57534324239500	3.35033488223102	7.58209012477835
H	6.35607467010337	2.62973949031913	7.31531661052137
H	5.23614986753276	3.12292781025973	8.60305382282262
H	6.02010109304268	4.35612255906652	7.58143750113062
C	3.33751255491136	4.30762988841489	7.05275670246751
H	2.44242892211376	4.28912868489704	6.41466272333569
H	3.74051196335270	5.33046848495617	7.05627634163745
H	3.02034259075875	4.07159429624533	8.07852103165847
C	4.27088389805285	6.09173481384635	2.33298270451138
C	2.74279646001660	5.93254792862993	2.20360975154339
H	2.25630636987033	5.87918607953262	3.18664477350603
H	2.49134174836104	5.01491785793478	1.65352426022763
H	2.31439527028133	6.78851818196549	1.66083978709687
C	4.58449223251357	7.38240703114870	3.11667367738911
H	4.15106964265593	7.35770202394596	4.12528017370215
H	4.17378584774970	8.25810690964584	2.59199337114724

H	5.67003876461127	7.51958297548713	3.22039780460846
C	4.86033821323811	6.23131344267677	0.92320991029322
H	5.94889785226071	6.38160812794096	0.95066720148417
H	4.41553092210131	7.10282054186426	0.42238830449393
H	4.65059326356638	5.34678743605369	0.30533125623551
N	6.32529052159583	-1.27001582897805	7.97336852634537
N	8.96074209808194	-2.54331455449622	8.78780869062919
N	9.67102080847411	0.26673176725963	3.37275200083150
N	7.29344647861434	2.08458985231889	2.88840364746259
O	8.24733435278728	0.11335780784785	8.71548614774428
O	10.18098084847089	-1.23569918627016	7.08645390859133
O	9.23330507864964	2.23230562428790	4.82470250270532
O	6.45250508476578	1.68890582094633	5.17319911095748
O	8.19962113297917	-0.01663603333652	5.98404515026230
O	7.71391571700334	-2.67792735740541	6.29358863853998
O	7.24609886884791	-0.64386708894192	3.50857481969937
Re	8.24368847466275	-1.37927604030931	7.29406351235582
Re	7.91160289303185	0.74596801063994	4.27910421107376

List S36. O_2, TPSS

O	0.00000000000000	0.00000000000000	-0.10975264115326
O	0.00000000000000	0.00000000000000	1.10975264115326

List S37. [Re(O)$_2$(ap)(isq$^•$)], TPSS

Re	7.24167178029517	4.55213459017137	15.96391706477092
O	8.29802597550763	3.61046804794596	14.45399427183714
O	6.24141334244767	2.74251654787323	15.94159983841170
O	8.50461166562605	5.71535400294014	16.14614849731884
O	5.94288823803068	5.28324161772849	16.83561494061104
N	6.17266535204560	4.90846952983616	14.24845486980866
N	8.32466121908535	3.31612901131288	17.19656096477648
C	4.89962491505912	5.54864514866610	14.26939773827157
C	3.74489162781881	4.81456511622556	13.97565594521954
H	3.83210307418498	3.75984587113391	13.73825685601314
C	7.76239686247340	3.52038101677217	13.25962372159529
C	2.50156790416189	5.43747181422061	14.02064923823071
H	1.60569390579179	4.86220904229222	13.80709494610494
C	6.78976767888424	1.69937706914635	16.51794174732309
C	7.95986696415097	1.99217147087243	17.27273442706162
C	5.93021340425256	4.33409571464238	11.84393211249120
H	5.04236501869293	4.94059811887650	11.72945326126512
C	8.11394052325643	-0.31169840826981	17.94805217334064
C	6.97122894996131	-0.58310606371556	17.15167429618448
H	6.61530447939174	-1.60616154597608	17.11506906833721
C	6.56778083726308	4.27561303564774	13.09303972089869
C	5.03757690824000	0.07750538346339	15.59631982474781

C	2.40441506948972	6.79006529324747	14.35214339313182
H	1.43302606633573	7.27346115197412	14.38688504621422
C	6.28041704969262	0.38139645151758	16.43283382353713
C	10.75661837874337	3.09456952875809	17.31408118098222
H	10.70435755056558	2.32448185851838	16.55196427410740
C	9.57713653118252	3.70820784088627	17.75145038210291
C	8.60125179072176	0.98455618824228	18.01072298667637
H	9.46673510823623	1.23398517427648	18.60932584994266
C	12.03333453903454	4.50982668944810	18.80028532124159
H	12.98949709609815	4.82422651914237	19.20694154861044
C	4.66899241629391	-1.41290451835875	15.65107216951774
H	5.47313578122400	-2.04329631112919	15.25610203290869
H	3.77736443616013	-1.58498329911560	15.03977710951717
H	4.44306014192810	-1.73714136944685	16.67261370331936
C	8.77196121392403	-1.46619008943182	18.70450965732997
C	9.62713720401714	4.72850698395078	18.70324951865321
H	8.70497379041444	5.20404907494678	19.01841709411980
C	3.55801198839198	7.51525752496232	14.65060283460626
H	3.48782892865808	8.56527502577734	14.91716261616483
C	5.78857440843694	3.59032343019828	9.40738752979248
C	3.84021383645031	0.89392865673634	16.13498044725910
H	3.62291273040964	0.61734054488099	17.17187047086800
H	2.95003517977695	0.68485323156717	15.53049697464494
H	4.04382993222710	1.96527993206822	16.09799639609741
C	6.44629411845021	3.59980326815790	10.78760173789362
C	10.85524556687943	5.12201637195192	19.22832181389882
H	10.89112373292663	5.91359391647648	19.97037799470196
C	8.30358733252741	2.75826535597552	12.19617889197844
C	4.80560570880267	6.89794119999486	14.61575083189099
H	5.70926470909871	7.44676597197053	14.85695026012940
C	11.98016773593654	3.49964409186455	17.83829357628155
H	12.89509344559321	3.03279545200883	17.48607266877620
C	7.61679786909228	2.82379205266246	10.99238733830358
H	7.99572865778907	2.25459363797282	10.15156225886755
C	5.29731931423658	0.45318320696734	14.11945702012278
H	5.55611758432819	1.50776279226643	14.01552941453617
H	4.39831821685491	0.25433675544127	13.52484699729745
H	6.11682278098524	-0.14468589619819	13.70890431452843
C	9.25386724916694	-2.52657081504639	17.68924878134532
H	8.42444336091150	-2.90944559504250	17.08723560240827
H	9.70932298409271	-3.37199001721653	18.21693791136306
H	9.99826920042693	-2.10062346323574	17.00900589276802
C	10.74405127062118	2.86125076519736	12.77664982965035
H	10.93496156579787	3.58450552487791	11.97694995067960
H	11.65329178810125	2.26696290952163	12.92402083546345
H	10.53302874438915	3.41001457030880	13.69633637433118

C	9.57177243908803	1.92857198542247	12.39598923260976
C	9.97853553107515	-1.00227683907419	19.53427414952197
H	10.75705328242674	-0.56249372972565	18.90211870620306
H	10.41451163107308	-1.85989820987358	20.05662473677243
H	9.68693358401967	-0.26229418396304	20.28664441233336
C	9.35041409684638	0.89234632387040	13.52168282946767
H	9.09664765166697	1.37801645971515	14.46476741570518
H	10.26481399863019	0.30548538563788	13.66530480469008
H	8.54097154324274	0.20462745216167	13.25845989227732
C	4.52934733946345	4.46896189255256	9.36062448248440
H	3.77315802733113	4.12622891001006	10.07458496889212
H	4.09014282015015	4.42534811368851	8.35876734748594
H	4.76131039171380	5.51612295396900	9.58045728474479
C	7.73990148279119	-2.10842737811263	19.65844668444426
H	7.38345853306326	-1.37764176273498	20.39111760037864
H	8.19713858290019	-2.94579824645080	20.19728724338530
H	6.87306737349511	-2.49079298256765	19.11158985215587
C	6.79162984116281	4.11745825151946	8.35745034696912
H	7.08993999038549	5.14435451646197	8.59067714607124
H	6.33309943750600	4.10716985256005	7.36227501508930
H	7.69523571664288	3.50173518058713	8.32040517013420
C	9.95605454656746	1.16781076134648	11.11750283425018
H	9.16905397464482	0.47040466728525	10.81037203960168
H	10.86500752750219	0.58705414067782	11.30448301757532
H	10.15980466922592	1.85067439942419	10.28581194346008
C	5.38707133566944	2.14240429513904	9.04776836819228
H	6.25569823330047	1.47745058051359	9.03171537453516
H	4.92163245713865	2.11740925682941	8.05609698824117
H	4.67155322923259	1.74791225082958	9.77632692914398

List S38. $[Re(O)_2(ap)_2]^-$, TPSS

Re	7.24024484422715	4.56064358972296	15.97651278562432
O	8.32971823728430	3.63899862869833	14.47644174296761
O	6.22670006213189	2.75764700665748	15.91606401085623
O	8.51756910799731	5.70940312729842	16.21817094924452
O	5.92081170745091	5.31264702399425	16.81575180537075
N	6.24986116763022	4.99421317563277	14.25326816017091
N	8.23421764814744	3.34775892582650	17.26913902269195
C	5.05758385753005	5.76846410715258	14.18507955897974
C	3.86088612837019	5.28768004647912	14.72325563671074
H	3.86174906090612	4.32329366422699	15.21710114938935
C	7.78400886321418	3.54219403258467	13.27051484998362
C	2.70102074284914	6.05439383260236	14.63348452155030
H	1.77574822898190	5.67707513939935	15.06085711441870
C	6.77139150237450	1.70244556505790	16.50971952661303
C	7.90353486749385	1.99655319628569	17.29637590691462

C	5.90825928555705	4.28012170005909	11.89056614902645
H	5.00028783146301	4.86250880259313	11.79724291510154
C	8.16369901260699	-0.33902896991040	17.84350173470752
C	7.02716478895284	-0.60805693316499	17.06431355012062
H	6.69729486537328	-1.63821740156652	16.97774614326395
C	6.61187771341920	4.30689525507118	13.09833136997094
C	5.07425003864423	0.07100266167737	15.53039762467764
C	2.72238347073432	7.29860537712072	14.00133093157482
H	1.81576222899413	7.89393754860470	13.93432215557001
C	6.30695111575019	0.38037729472910	16.38333816099008
C	10.62603452106557	3.87718053876331	17.29403937976098
H	10.64430553336886	3.72680076091117	16.22077632195241
C	9.41313206867614	3.72743188302550	17.97173477635230
C	8.59990538248321	0.98226848968570	17.96007663904939
H	9.47576979640552	1.23936897785632	18.54253227960787
C	11.72542599567469	4.43423168936367	19.37728189429090
H	12.62346444067319	4.71298690162704	19.92196866156997
C	4.72207404771125	-1.42481695823186	15.54904572382014
H	5.53440241201314	-2.03443550779175	15.13784325085758
H	3.83052548677923	-1.59284690510268	14.93398824284164
H	4.50544419399691	-1.77763406271163	16.56387448568230
C	8.90185439137491	-1.50121731539966	18.51722079801886
C	9.36034451603945	3.93161919473078	19.35277349673662
H	8.40857290978738	3.81880620657502	19.86230194224845
C	3.91732642925109	7.77638184492702	13.46157508221640
H	3.94593682376891	8.74754766554291	12.97419527911301
C	5.63284395006264	3.38753497461110	9.50410016738042
C	3.86236577662845	0.85669139872976	16.07986041502281
H	3.63746875415109	0.54379220743766	17.10574910057927
H	2.97770102349486	0.66858997279315	15.45805143686203
H	4.07103392792978	1.92802008801058	16.08248103053992
C	6.37695496202186	3.48852029414675	10.84031061977809
C	10.51383420952784	4.28302017696938	20.05257667662128
H	10.46383589836399	4.44587548976289	21.12609491169490
C	8.28331675325063	2.74546380134796	12.22351290372724
C	5.08140561457034	7.01520624533582	13.55334123737542
H	6.02021552874759	7.37536194786149	13.14453223262530
C	11.77500685431075	4.23310979131959	17.99675269517467
H	12.71323551959925	4.35634880780905	17.46223468512138
C	7.55458211312807	2.74702083748391	11.02851024663516
H	7.91079794466918	2.13411431765795	10.20700410873738
C	5.33133568473323	0.48483601738645	14.06420064599845
H	5.58070165930439	1.54374527359815	13.98975744526456
H	4.43666018864858	0.29119947581534	13.45848559953926
H	6.16043508671509	-0.09353043976651	13.64445609154235
C	9.40201680155614	-2.48407193588446	17.43575783081082

H	8.56761451690529	-2.88160869927217	16.85005388021646
H	9.93394354459635	-3.32824007831557	17.89404432187436
H	10.08315207759212	-1.97505303732945	16.74628921419901
C	10.73081946307399	2.87464776518552	12.75337081070207
H	10.91390775751653	3.56476462427899	11.92186065756738
H	11.64683609713700	2.29634358989679	12.93028129645650
H	10.50598301321554	3.46179186891335	13.64599717778897
C	9.55995803001780	1.92398910498436	12.41759098736452
C	10.11594369496080	-1.02499816229832	19.33014979805069
H	10.84930545901023	-0.51960906986187	18.69335790470883
H	10.60750833529795	-1.88638883088112	19.79760500054222
H	9.81887553078929	-0.33064319870634	20.12297946097116
C	9.37070864542332	0.91591436309487	13.57320224038931
H	9.11059467878858	1.42423608729473	14.50196438489562
H	10.29754596374665	0.34894044740899	13.72872570974168
H	8.57147375748149	0.20734144530722	13.33458904624800
C	4.36795663093987	4.25970215043778	9.47772278187421
H	3.65887904043434	3.95856747169350	10.25564000429608
H	3.87016451246367	4.15785814329598	8.50605742843319
H	4.60845727239206	5.31741820547078	9.62748518407486
C	7.94377746086418	-2.24562571651307	19.47219487866426
H	7.58317772237055	-1.57043993850069	20.25535418542665
H	8.45386590001499	-3.09230123970068	19.95010287681077
H	7.07244445988051	-2.63102118474458	18.93428917363476
C	6.55789099099740	3.84510774700428	8.35568643910363
H	6.85923412492999	4.88786791776394	8.50011483467104
H	6.04413824289863	3.76147809335275	7.38897846694088
H	7.46553192270345	3.23533445451813	8.31429124954920
C	9.93346331961411	1.13399086776028	11.15336179381265
H	9.15073266462306	0.41682128410247	10.88197891506879
H	10.85597267496628	0.57164493102930	11.33869984715498
H	10.10641477656752	1.79670093684150	10.29782452357796
C	5.21227840378922	1.92188865968470	9.25849187263636
H	6.08433709540199	1.26177648213050	9.22581573016750
H	4.67540829784059	1.82977538668073	8.30519930473183
H	4.55722434218652	1.57268658496260	10.06312780601283

List S39. [Re(O)(ap)(isq•)], TPSS

C	8.34163819688764	3.41436428821184	4.81357656891030
C	9.20050035499739	4.49029762954543	5.10713430073905
C	10.55356265500999	4.26472621946378	5.35346123207096
H	11.21127539600832	5.10347440109296	5.54919326430078
C	11.04353749757274	2.95730248735844	5.33007385292766
C	10.15522012453509	1.90614163191357	5.05555709078194
H	10.53909974397484	0.89549593546541	5.04723739488191
C	8.79204820887836	2.09056688441499	4.78459105417184

C	9.22942817874665	6.92392136251477	5.37018683383114
C	9.20751391681082	7.99428041652852	4.47331339514435
H	8.70019845951944	7.87508373319968	3.52108135020654
C	9.82264683065201	9.19746209840485	4.81202122778595
H	9.79770797173097	10.02702503824218	4.11216883070719
C	10.46204705519660	9.33862971682880	6.04311959838499
H	10.93750358043356	10.27823157328507	6.30654939052088
C	10.47961662348798	8.26910178499819	6.93962774801411
H	10.96084038972116	8.37698022930070	7.90707790097501
C	9.86303324426782	7.06557556423393	6.61064226953027
H	9.84946816733025	6.23917690937966	7.31341396389409
C	5.14242575052797	7.25150300286316	6.45650217957984
C	4.72479609916534	8.40198974157012	7.13485359639887
C	3.38463933553643	8.41868633239579	7.54507890677901
H	3.02831869003594	9.29053775018945	8.07634430847672
C	2.48457258689449	7.37036250251890	7.29914026339422
C	2.93712463420454	6.24317689975017	6.61061619762287
H	2.27030102809063	5.41684197121685	6.39159548757265
C	4.26638621260118	6.17833548962604	6.19978156840414
C	4.24729871358752	3.85041700095641	5.46436085611343
C	4.03515679146709	3.21948206396667	4.23530031148594
H	4.34395252359763	3.72483416935169	3.32572698667615
C	3.43389290283804	1.96410517965897	4.19127508147458
H	3.27705512339050	1.47872763833887	3.23286011549654
C	3.03253978741847	1.33495705312321	5.36916920808817
H	2.56254141279709	0.35713095811790	5.33293680688868
C	3.24327297613381	1.96612485583821	6.59678993948940
H	2.94385174779699	1.47680651308317	7.51877366597320
C	3.85093033856606	3.21691618431893	6.64872587352609
H	4.02821850118225	3.70963160874770	7.59927675614719
C	7.84916758998847	0.92245321327653	4.48962912715659
C	7.20693201186073	1.10690178028784	3.09678393832881
H	6.63859940509782	2.03634692839328	3.03997735257508
H	6.52629854834887	0.27277463187825	2.89154394882948
H	7.97770248279278	1.12054519110104	2.31901592424170
C	6.74992539429785	0.86838457491334	5.57270320907040
H	7.19588572508376	0.71280274121474	6.56072365127580
H	6.06744687887196	0.03678649793576	5.36769044705346
H	6.16547020411791	1.78886713520369	5.59564724925295
C	8.59006093512727	-0.42443617606783	4.49496742202930
H	9.37865940728227	-0.45775732493738	3.73576111429634
H	7.87552119999501	-1.22360794220519	4.27272587928190
H	9.03779740349640	-0.63823086535064	5.47133028935166
C	12.53290181783504	2.71912868336670	5.59926585726629
C	13.36693533826140	3.46130576101279	4.53186882095295
H	14.43678613094835	3.31208318503908	4.71695061048965

H	13.16556260953706	4.53644788292460	4.54648252197124
H	13.13313729413853	3.08646677435471	3.53033507145381
C	12.89235831420185	3.26929319480665	6.99682437203982
H	12.31726770023265	2.75526862192077	7.77375403772147
H	12.68011307561935	4.34026908776681	7.07065068317913
H	13.95895167355211	3.12006806561385	7.19945087113985
C	12.90621487960250	1.22940154290863	5.55540071916931
H	12.36047821030157	0.65431640581270	6.31079640641264
H	13.97667812459023	1.11685472674377	5.75621448951141
H	12.70150137429600	0.79177244453363	4.57287414275090
C	5.68170995467732	9.56579974397493	7.40120216279744
C	4.98600054874096	10.71745911089710	8.14409730029613
H	4.15145983836939	11.12642578183816	7.56477888644022
H	5.70872079773987	11.52345961621591	8.30719418721285
H	4.60847191211998	10.40211565152224	9.12269008587014
C	6.21216436989455	10.11558293093465	6.05813902600169
H	6.75584864899063	9.35070552726106	5.50245511541922
H	6.89374562785125	10.95305810863402	6.24551889684843
H	5.38438708704236	10.47716177601034	5.43915869287443
C	6.86375581497244	9.07834709032656	8.26835167420165
H	6.50190183730166	8.70460886573746	9.23222373143948
H	7.55152374485096	9.91033071324337	8.45847978542359
H	7.42037460805216	8.28323255317891	7.77063260838178
C	1.02230739284025	7.42307547801355	7.75409428240363
C	0.10225819501478	7.34098958547576	6.51610188700948
H	-0.94939761898808	7.36947974018077	6.82330374104129
H	0.26826824710322	6.41494909858791	5.95757876322628
H	0.28932223717235	8.18168784540380	5.84063832561639
C	0.69246867098359	8.71341813894754	8.52014532087643
H	1.30626239627935	8.81298826916897	9.42141155812615
H	-0.35799997074089	8.69516378650959	8.82845666561014
H	0.84392601869483	9.60188568768825	7.89834743167883
C	0.73430617858091	6.22151485908234	8.68065488265108
H	1.36818942640275	6.26056712225717	9.57258123433633
H	0.92340192589258	5.27156470750122	8.17178283180310
H	-0.31381730899333	6.23297082849667	9.00072557627067
N	8.54535235651440	5.72066951552985	5.03665123901000
N	4.87345865221244	5.12360069874212	5.49780968430569
O	6.22913663155116	6.38074286567762	3.14537299256252
O	7.05975597675261	3.79270278468950	4.53615574501614
O	6.42656751954403	7.05947689320341	6.01513609138713
Re	6.58359280150768	5.67440304260581	4.65780605969265

List S40. $[Re(O)(ap)_2]^{-}$, TPSS

C	8.34505199048515	3.36320357496728	4.95151980980246
C	9.21380592937239	4.44887995256139	5.16602147233300

C	10.58245164410685	4.22786912995651	5.30128452178342
H	11.24634793305160	5.07548620053892	5.43758023027164
C	11.09410631875502	2.92423129459217	5.25019956286508
C	10.20555918782747	1.86297221034871	5.06021637161930
H	10.59317722014086	0.85330590175698	5.02435416549744
C	8.81948577411917	2.04757572864487	4.90333973877364
C	9.20949030805356	6.83307344282479	5.64550162918308
C	9.11208351102016	8.03398810745898	4.93062465712692
H	8.54039547510507	8.03615203183693	4.00749785214183
C	9.71594463437955	9.19382632367383	5.40751726687931
H	9.61857097388384	10.11768012358029	4.84351020070219
C	10.43607429926381	9.17771302524794	6.60256010742176
H	10.90362583226038	10.08447613050296	6.97603451270738
C	10.53505388959592	7.98462553911047	7.32179668212324
H	11.07468455455516	7.96152928565672	8.26541616286044
C	9.92805641018309	6.82346435741345	6.85204288712496
H	9.98874969578369	5.90158227245431	7.42159490366780
C	5.08178746053814	7.33311138693637	6.30977843300578
C	4.66631958665067	8.47622883076021	7.00184095858369
C	3.34044567415513	8.49020425848432	7.47249590898186
H	2.99816375342481	9.36362189130323	8.01204586660998
C	2.45300425968232	7.43105294779670	7.26705625457767
C	2.90304584680432	6.30244628106347	6.56915035031638
H	2.23498739040349	5.46789492550780	6.38263750910736
C	4.21464398814094	6.24421118866968	6.10380576737091
C	4.19553967264337	3.93785697477491	5.33292108592148
C	4.11773675151295	3.25021830392536	4.11477899533202
H	4.52109189761346	3.73187411253957	3.22923771889685
C	3.55824258240134	1.97726543430156	4.05334419226410
H	3.51875205396633	1.45638042190861	3.10037821329031
C	3.05680214116239	1.36803080872876	5.20421723526161
H	2.62338849845859	0.37294648876135	5.15618332685308
C	3.13367449786415	2.04641383612658	6.42254650022204
H	2.76689596582935	1.57523070617890	7.33118686037296
C	3.69834143645323	3.31681273805119	6.49022773677359
H	3.77743575384637	3.83435767739499	7.44084364261498
C	7.86219058962844	0.87401860276115	4.67508891711778
C	7.16753197128903	1.04008229791831	3.30533363769246
H	6.62540406486529	1.98528620720027	3.26015984253803
H	6.45674189968519	0.22024686763698	3.14038970136184
H	7.91066822331565	1.02232704077548	2.49996123383489
C	6.80112409760135	0.83706451530822	5.79653359376041
H	7.28589510647797	0.70310478190368	6.77041210781108
H	6.11145238560406	-0.00042072100022	5.63528600435195
H	6.22144539895586	1.76016751614669	5.81863481737771
C	8.59549128767695	-0.47741727694058	4.67271391137478

H	9.34414812887434	-0.53192408984855	3.87431939350436
H	7.86672621102629	-1.27940583287044	4.50725171768827
H	9.09608025611742	-0.66802636444861	5.62877211962736
C	12.60615051737934	2.71808612531081	5.39708144764083
C	13.33990903104440	3.47223317196419	4.26663400816350
H	14.42672161934970	3.34981352357467	4.36255055517453
H	13.11161685662400	4.54166319636446	4.29406950251143
H	13.03118640158402	3.08785071482238	3.28889455377852
C	13.07350882153530	3.27569258776397	6.75898502706332
H	12.57706698583414	2.74624596428326	7.57912273225156
H	12.83462299442586	4.33936801939879	6.85227267796008
H	14.15878618814357	3.15601764746810	6.87322373680432
C	13.00519533452832	1.23616799733269	5.32170118538297
H	12.53180420770068	0.65384997618795	6.11936203768914
H	14.09216824071977	1.14089599757702	5.43021133469252
H	12.71979146769095	0.79582147585637	4.36055117432724
C	5.62093036960998	9.65294432343984	7.22257933204900
C	4.95204169310946	10.80316924675739	7.99271654348766
H	4.08452737259156	11.20026650110786	7.45370032779692
H	5.67513118687582	11.61754375343380	8.11743005278404
H	4.62425262066603	10.48813726856595	8.98985298209354
C	6.08382274983946	10.20288772914122	5.85544784587174
H	6.57925236022547	9.42296684419393	5.27652691606426
H	6.78793610897290	11.03176681390243	6.00259756308391
H	5.22472696914597	10.57440674673517	5.28525785642531
C	6.84801861155010	9.18538143665669	8.03553236137986
H	6.53091309321008	8.80459828988489	9.01321490381383
H	7.53486239143082	10.02515021038522	8.19791299105640
H	7.38917906465948	8.39673640767671	7.51175940691131
C	1.00794511586586	7.45426707165581	7.77887798833987
C	0.03361627898121	7.32111577543270	6.58850097196652
H	-1.00642409422040	7.31578716003192	6.94017059898000
H	0.21150950341676	6.39477603782060	6.03438494696578
H	0.16112860270463	8.15761774914889	5.89337752421676
C	0.67318070098137	8.75158580511567	8.53110253523026
H	1.31818983570349	8.88114816980616	9.40657980742952
H	-0.36707870653749	8.72000562188713	8.87625596929335
H	0.79063484976585	9.62902013556411	7.88643704140432
C	0.78698787674045	6.26847300577466	8.74357980361211
H	1.45608948030032	6.35020940240264	9.60665989240913
H	0.99130799074179	5.31456220652493	8.24836837960786
H	-0.24918880582310	6.25287144861500	9.10615434935809
N	8.54866981190823	5.68556652370497	5.15885752913076
N	4.81153211378007	5.20662895660208	5.36985824405512
O	6.47340317654903	6.06057114022425	2.95944958616490
O	7.03110507373318	3.69012754640466	4.80294262116682

O	6.33488789206612	7.18335710677667	5.79741302692697
Re	6.62040563028306	5.62279977583761	4.60187383820120

List S41. [(ap)(isq•)Re(O)(μ-O)Re(O)(ap)(isq•)], TPSS

C	5.19064616310746	-2.17404749464017	7.50954485243125
C	4.77144498246706	-2.12384690076529	6.17804210198266
H	5.27931903429311	-1.46794210316942	5.48116042696007
C	3.70504216726711	-2.91724832976078	5.76754977792478
H	3.37352861540155	-2.87182576861797	4.73503653694042
C	3.07247890702066	-3.77184372910554	6.67226204288681
H	2.24745945396116	-4.39653751179371	6.34390016276028
C	3.50698427836795	-3.82731553241832	7.99681332155783
H	3.02889553364175	-4.50059694916006	8.70191477880482
C	4.56175692298642	-3.02451235762545	8.42232654286695
H	4.91718227855190	-3.06594249965655	9.44516038055856
C	7.22099786172043	0.23527516254990	9.33371274197533
C	6.05180518434849	-0.48007770825826	8.97243941294935
C	4.83497079592498	-0.25106830473189	9.63614279835889
H	3.94448923631402	-0.76805841588511	9.30796130535793
C	4.80126079553750	0.64644378558435	10.68894584602276
C	5.99877969724501	1.30205192766427	11.06675462733246
H	5.95385626415578	1.98667570487438	11.90620307218046
C	7.21859623750891	1.13476440502340	10.42299992136695
C	3.51688622800801	0.94671071727161	11.46381521712509
C	2.31372371350431	0.17202575158144	10.90570511047619
H	2.46780289654513	-0.91031281369051	10.96903800017800
H	2.11929814214968	0.43128717455488	9.85987015204064
H	1.41854998095105	0.41855487375928	11.48567126275101
C	3.20602643100049	2.45692383057584	11.38073651566518
H	3.05294446551040	2.76233104833084	10.34103099853512
H	4.02157242480129	3.05850785275543	11.79323092637135
H	2.29580630594832	2.68784580376707	11.94544203114398
C	3.71448960849981	0.54524778233597	12.94204350106276
H	3.92909385535649	-0.52532844565527	13.02369317732767
H	2.80799784795042	0.76238053786988	13.51827674912660
H	4.54477800737273	1.09338583149926	13.39754439619632
C	8.49888398535462	1.85213007000913	10.84733218370696
C	8.25777162770525	2.78508969182713	12.04342762411253
H	7.90501360119884	2.23602906978203	12.92349409122130
H	7.52817543036366	3.56683707766710	11.80563254294773
H	9.19949126772507	3.27536360590283	12.30995723864501
C	9.56604719652199	0.80987748952839	11.25508023979822
H	9.79681678495144	0.12771084334295	10.43413794011617
H	9.22281476136882	0.22350359715369	12.11472150302924
H	10.49076585966051	1.32175660152226	11.54415887496881
C	9.02783482566925	2.69781917195114	9.66977649967401

H	9.22943040141117	2.07344140970878	8.79913903448722
H	9.95751930527966	3.19894574277157	9.96119034445377
H	8.29766243020041	3.46244635558522	9.38741817521939
C	8.01317936864572	-3.31631949383031	9.63806156428969
C	7.74719784927333	-2.69063172731444	10.86220244598855
H	8.21618653154924	-1.73962622033787	11.08424207411709
C	6.87957075838925	-3.28884274074667	11.77256100657207
H	6.66190854126649	-2.78996237702719	12.71236588598920
C	6.29189941720124	-4.52121599438955	11.47901305531083
H	5.62019202892283	-4.98805176198735	12.19299452102181
C	6.56774757312333	-5.14834757812911	10.26352171322397
H	6.10874995965538	-6.10335232795886	10.02635356943769
C	7.41719537493254	-4.54433334289034	9.33784866881549
H	7.61880984648906	-5.00496274576448	8.37684965504948
C	10.22923696571747	-2.64835273173255	8.91852045673038
C	10.93010430465277	-3.22757796734197	9.97408400420386
H	10.39441305781658	-3.81086948514522	10.71529675857908
C	12.31390459463335	-3.03764174936298	10.05937476182022
C	12.95770597808560	-2.26950765225847	9.07934758445246
H	14.02601718180398	-2.12088804774659	9.15648363213617
C	12.28557325384796	-1.67277327567900	7.99975048152333
C	10.90908073721571	-1.89283270387004	7.94653925927390
C	13.07389376424966	-3.67075418736784	11.22960917405842
C	12.51056692675830	-3.11993187999992	12.55788747824446
H	11.44337698861952	-3.33893357606663	12.65869381189924
H	12.63906190822048	-2.03383921463222	12.60958219531855
H	13.03379794665643	-3.57223427680132	13.40836430568315
C	12.88189019446375	-5.20263883973252	11.19345086382021
H	11.82418223952143	-5.47352421175641	11.26274862409286
H	13.40919935779663	-5.67140996778818	12.03238536820640
H	13.27596491069712	-5.61880842330064	10.26070720264778
C	14.57989538871844	-3.37187036582624	11.18292084886977
H	14.77918848515872	-2.29655516169505	11.23858408673854
H	15.03945094017442	-3.76108648381095	10.26838377849209
H	15.07288046997985	-3.84912329481955	12.03649055462589
C	12.99558454739610	-0.82033451593866	6.94744016501234
C	12.39505507641192	0.60371445149711	6.94475264359341
H	11.33117397723390	0.58254358549331	6.70520343111227
H	12.90338474609587	1.22233058627595	6.19774625441108
H	12.51775962345310	1.07417233158071	7.92620498884424
C	12.81021673231265	-1.46260068967672	5.55493091566124
H	13.23459205711819	-2.47183614440015	5.53813785750736
H	13.31988063729827	-0.85898050152139	4.79636843943535
H	11.75427434486499	-1.52582512294170	5.28784422463369
C	14.50239356177535	-0.70657898090272	7.22454276674777
H	14.99385001633166	-1.68509189897692	7.20185995436909

H	14.70323583210174	-0.24044140256343	8.19495711498352
H	14.96110265657502	-0.08215987663875	6.45094022625460
C	9.86901786873161	-0.92240107923162	2.45897837248202
C	9.73125556667859	-2.20797232210448	2.98758036964708
H	9.45238923956189	-2.33392543941244	4.02700450647405
C	9.95816532461461	-3.31068211136335	2.17076757714264
H	9.85988630999369	-4.30923383494810	2.58460517928476
C	10.29975248348821	-3.13593516743497	0.82842712925882
H	10.46564027050552	-4.00015627747002	0.19253847062757
C	10.42008439884815	-1.84889597852807	0.30388435413856
H	10.67078242133887	-1.70536705562689	-0.74282179964255
C	10.21242427900330	-0.73818961891184	1.11719397838214
H	10.29123376410800	0.26654268458481	0.71877864039105
C	10.66869250650380	1.11855782780267	3.43561245058382
C	11.95150830197423	1.03882405512926	2.86903049862418
H	12.22362768123796	0.16881582447330	2.28873984962879
C	12.84789957495806	2.07108364599398	3.08442151436365
C	12.43788531649334	3.17966794203222	3.86440190364836
H	13.14735121687423	3.98743320567130	4.00447121534349
C	11.19253080653609	3.29164515526948	4.47088474657368
C	10.30627125081222	2.21206954131974	4.26145106484568
C	14.25944228909994	2.06134699972469	2.49516610992159
C	14.54975683068560	0.76892092095249	1.71771516908305
H	15.57113622705852	0.79896700185966	1.32479994393410
H	13.86697054604974	0.64818905107586	0.87015741002561
H	14.46140558510177	-0.11394565370760	2.35928552192102
C	15.29602389895602	2.19248502828854	3.63180215941284
H	16.31014873220611	2.21124347256137	3.21735494082956
H	15.22210076049840	1.34726984474586	4.32322133048661
H	15.14785560690054	3.11238903476752	4.20508380619802
C	14.40690837861435	3.25815796644435	1.52951654730740
H	15.41418788522358	3.27638858060168	1.09834149870848
H	14.24144243146382	4.20905883711386	2.04527239039424
H	13.68064138741680	3.18305748995675	0.71371711855995
C	10.77011606599634	4.50197035491138	5.30265466739457
C	10.38665235735777	4.04499334697149	6.72547606984435
H	9.57272850676447	3.32059125634732	6.69646669437458
H	10.06203736813445	4.90802368751785	7.31724198827391
H	11.24327883095806	3.58526589019662	7.22785993120149
C	9.55090328188747	5.18087534708773	4.63659542830798
H	9.81383202182412	5.55218047717528	3.63969740445264
H	9.22701858605829	6.03568855606131	5.24087939617119
H	8.71008252604403	4.49038463560365	4.54551586451438
C	11.89859266268424	5.53791787016494	5.41461991746529
H	12.19381551618357	5.92677772538642	4.43376644625105
H	12.78449552340794	5.11878504775048	5.90398064423441

H	11.54946169403771	6.38214276115740	6.01758549813416
C	7.88804719378245	2.07279058154774	1.51589953368087
C	8.87970212072650	3.04203968830891	1.32120424384741
H	9.14101271427879	3.70439094684917	2.13807653298658
C	9.52286210889971	3.13785550901998	0.08967042462926
H	10.30323748909323	3.88005476898450	-0.05050951706465
C	9.16932632976416	2.28415741605386	-0.95752966398659
H	9.66875887525241	2.36513452120171	-1.91821241468310
C	8.17598187778838	1.32392317176986	-0.76302527715788
H	7.90191510577796	0.65181764120996	-1.57056277181568
C	7.54273631016249	1.20851281323970	0.47326065049977
H	6.78907401720903	0.44873242235691	0.64931084033842
C	6.35624212319735	2.91072460269611	3.20013284674216
C	5.93034081456144	4.03759696096062	2.49109930577444
H	6.29959922371677	4.20318021530974	1.48691431106401
C	5.04809476967131	4.92704125782328	3.10404834187327
C	4.60461680792405	4.65923797291391	4.41192138764892
H	3.92320476132874	5.36267808602785	4.87573431296976
C	5.00298893964490	3.53894183870220	5.15017689324509
C	5.89159844299595	2.67394229472932	4.50292890295665
C	4.53108845361140	3.26662218271057	6.57897270994332
C	3.81107662640482	1.90128886464529	6.63387129912068
H	2.93332460157524	1.90084846249629	5.97912163508147
H	3.47982768598646	1.69731027984668	7.65766289177856
H	4.47626893990569	1.09429228944243	6.32355581362320
C	5.75193833386335	3.24704560477839	7.52646033850259
H	6.46949663924051	2.48047613462608	7.23108160891614
H	5.42788834709789	3.03738670763002	8.55151305369396
H	6.25878031743142	4.21782607819921	7.51455821743296
C	3.55522835549910	4.34442498566999	7.07494104234987
H	2.65614561492317	4.39563482085942	6.45160739276306
H	4.02176625215223	5.33511631187567	7.09340322344421
H	3.24344631443919	4.10233290128228	8.09629905040705
C	4.56184699907103	6.19718536505471	2.39877662126901
C	3.02225051040626	6.16879206862701	2.28903030178318
H	2.55318176216216	6.11254676828897	3.27589494640129
H	2.69268666077735	5.30160668492341	1.70787735074515
H	2.66154067246896	7.07674029150589	1.79191758236092
C	4.99052632994908	7.43100296868793	3.22312383554118
H	4.56911846864963	7.40127712672722	4.23213941792129
H	4.64576492261671	8.35096322544363	2.73677311664263
H	6.08091254933722	7.47370008904336	3.31281051675830
C	5.14586736144276	6.33608885067045	0.98467599317782
H	6.23915230257605	6.39500493354962	1.00568986398691
H	4.76845990271344	7.25449922116898	0.52291239080126
H	4.85826840246285	5.49487420056845	0.34543537998062

N	6.27841642216952	-1.34861577618435	7.92654586542400
N	8.86841038460678	-2.70265978142055	8.68057697189849
N	9.64594814048262	0.20282151291285	3.30904588703360
N	7.24061465412581	1.93735368604983	2.77557990671512
O	8.27465150001528	-0.04522656664973	8.59917609196313
O	10.08786187437535	-1.39339336618218	6.98576638497201
O	9.09159635236228	2.13396699957061	4.75956388177277
O	6.40628046316340	1.54658209632588	5.05982673408120
O	8.12086881178503	-0.18293704109309	5.86638630974516
O	7.55862021316344	-2.81827735753246	6.21956930910651
O	7.25561699569216	-0.79005492854172	3.36075625978566
Re	8.16016027744969	-1.52785070574493	7.18737656863051
Re	7.85604802715425	0.60332450650846	4.17436807629631

List S42. O_2, B3LYP

O	0.00000000000000	0.00000000000000	-0.10236670874370
O	0.00000000000000	0.00000000000000	1.10236670874370

List S43. [Re(O)$_2$(ap)(isq$^\bullet$)], B3LYP

Re	7.22676128768965	4.62257093992793	15.98460636596698
O	8.28835297568304	3.66791605858520	14.52379175889536
O	6.19635640669355	2.78193808599293	15.95462162044911
O	8.45976203415548	5.79107418857187	16.21040327693912
O	5.91976936298428	5.30773445401999	16.85375659223172
N	6.19603370592564	4.95861777159745	14.27705734695629
N	8.29215152732874	3.34119251412697	17.22343856492468
C	4.94644372653018	5.64003095763338	14.26845732739585
C	3.76441323832991	4.91324249914084	14.15019796206531
H	3.81286991429161	3.83660415781335	14.07153279925624
C	7.75837346696586	3.53144045685331	13.32935835327175
C	2.54617985503347	5.57551509141959	14.15832236003508
H	1.62813184343394	5.00878434433189	14.07761033601620
C	6.75479060840687	1.75290892668239	16.49446595897581
C	7.93290360501820	2.03455506307468	17.25528466339108
C	5.93686901403266	4.29363693436461	11.90129348862885
H	5.05372972009524	4.89531742303994	11.77360870227727
C	8.13192856333286	-0.28617571609665	17.83369120915864
C	6.98348174373980	-0.54454794264314	17.03376051533589
H	6.65383325681728	-1.56765377898568	16.95557801889450
C	6.58368901602766	4.28531854153186	13.13924797180682
C	5.04152301440096	0.12994643352000	15.50951345894443
C	2.50307793346122	6.96043372177077	14.28154254595577
H	1.55149361088929	7.47468759143171	14.28948933815046
C	6.27401594679371	0.42393738911335	16.36315059727175
C	10.72526037235420	3.25623127221341	17.25458901698626
H	10.69075750572684	2.60264704125710	16.39473718335680

C	9.53404569535077	3.72846254391835	17.79952140550154
C	8.59457774900958	1.00400589211550	17.94275307990675
H	9.46185846158691	1.24398949610053	18.53272765678357
C	11.96061980947767	4.51372392063582	18.89496066056697
H	12.90587281688714	4.82245307036682	19.32046315293101
C	4.70679432422339	-1.36594350055438	15.49594285989378
H	5.52072933815774	-1.96036005039746	15.07772439243163
H	3.82544313384410	-1.53091487891892	14.87558127322861
H	4.48231314844331	-1.74235869322829	16.49525680925936
C	8.81813030065624	-1.46175055604337	18.52568234951887
C	9.55340379975621	4.60126991500225	18.88011826870800
H	8.61917230296570	4.97522384668817	19.27438679671373
C	3.68516982508434	7.68002208187454	14.40488590634014
H	3.65736604187461	8.75632371724643	14.50920396821580
C	5.75794735515078	3.44355681658721	9.50348584537876
C	3.82680835358159	0.88854338290643	16.08081420100034
H	3.61898890894669	0.56602768884593	17.10236823740170
H	2.94293555121486	0.68052408338277	15.47445508308128
H	3.99386235085223	1.96247297182424	16.08899513463474
C	6.43321925930749	3.51138513373750	10.87416376545116
C	10.76817327925204	4.98749285044021	19.42920653590108
H	10.78216624579462	5.66582935785412	20.27156630500927
C	8.28455095406711	2.72537537996188	12.30366914655389
C	4.90800910861312	7.02205850339756	14.40150298408970
H	5.83506104695797	7.56777789887489	14.50734738808268
C	11.93576344391423	3.65177640928833	17.80397708712579
H	12.86122594009132	3.29408994509381	17.37282601042390
C	7.59338347450451	2.74280782136829	11.10357164320190
H	7.96190604344898	2.13625345971285	10.29226388426955
C	5.29598061383675	0.57283492274185	14.05431397573303
H	5.51262327587266	1.63478527060010	13.98468144027789
H	4.41465291390128	0.36173541352967	13.44570134932002
H	6.13751518219701	0.02743063922960	13.62646809007937
C	9.31157384549015	-2.45379712429679	17.45420806785837
H	8.49080323305889	-2.83955829252526	16.85018959059833
H	9.80703435284504	-3.30354031093180	17.92748219369111
H	10.02412216490393	-1.97218134230849	16.78345735322781
C	10.72301042230236	2.83973219155152	12.86598787342987
H	10.91088499968688	3.52423664491755	12.03719571986522
H	11.63136969686534	2.25705858089700	13.03475813858082
H	10.52134957124592	3.43125297616512	13.75577816521096
C	9.54877886374574	1.89776030049192	12.53495284166764
C	10.02241610998266	-1.01917256612468	19.36364149810266
H	10.78723702703653	-0.54121488813168	18.75035949951276
H	10.47565435917599	-1.88936466777490	19.83951853709844
H	9.73178520831302	-0.32329907302203	20.15148388555807

C	9.32815652953821	0.91566086536521	13.70278151800568
H	9.08834429535402	1.43712676190155	14.62498747400912
H	10.23177934740634	0.32467858863409	13.86596227751288
H	8.51164605433588	0.22867588339527	13.47908604827819
C	4.50686502719433	4.32621545854339	9.43014786847950
H	3.75631946287976	4.02394560538845	10.16160619540823
H	4.05732259764193	4.24281512068850	8.43983462530427
H	4.74415658846759	5.37729212668266	9.59875024907322
C	7.81251041618808	-2.16700466237768	19.45587459918517
H	7.44578447136031	-1.48103559273314	20.22060488973516
H	8.29141397423147	-3.01111043439090	19.95544367853882
H	6.95163951092337	-2.54915028489055	18.90829318665442
C	6.74505084840336	3.91709881739245	8.42018986234223
H	7.05954823837569	4.94450632722800	8.60823297876752
H	6.27340476264813	3.87827100760091	7.43615444657586
H	7.63893786271625	3.29465027531449	8.38667959482273
C	9.92751040972824	1.07797513414099	11.29630268456487
H	9.14280558253385	0.37062722129957	11.02314130725864
H	10.83186865827297	0.50502265411425	11.50516266353850
H	10.13163962148114	1.71522126366750	10.43485023798618
C	5.33839165471356	1.98984492235657	9.21388532679002
H	6.19496292623554	1.31639683914472	9.20303784463969
H	4.84855374023617	1.92472274165829	8.24022315489147
H	4.64090825951539	1.63156975649531	9.97236490268000

List S44. $[Re(O)_2(ap)_2]^-$, B3LYP

Re	7.24860127182932	4.61521331662167	15.99210124630053
O	8.32852566275536	3.65340752691577	14.51792313477303
O	6.21006523006816	2.82979466560138	15.94320643067883
O	8.51888206757248	5.76027255911619	16.19173391566979
O	5.95015491435371	5.36597100893538	16.83762220736419
N	6.25316321730739	5.01501433828094	14.26324486650374
N	8.24654449146727	3.38934939110151	17.27042006118080
C	4.96374508110390	5.60011845748480	14.26943285289579
C	3.82512443066146	4.80640286803415	14.13104790011721
H	3.94456675941138	3.73766859483371	14.02717786949566
C	7.79961407467289	3.55461102427795	13.31423431715386
C	2.56504577949598	5.38467079564319	14.15170810651528
H	1.68740535130782	4.75769091225115	14.05607089811512
C	6.74765239169374	1.77303959403944	16.51963194761299
C	7.88872779976998	2.04483307253381	17.29031643415836
C	5.98880376801447	4.32152767824207	11.88959761030199
H	5.10570762906409	4.92569841979471	11.76745616766214
C	8.08031412379250	-0.28526202092631	17.86942626024057
C	6.95522132950035	-0.53418284840833	17.07582664285268
H	6.60879182418756	-1.55228657302492	16.98722001622066

C	6.63654162075217	4.31639788454611	13.12233290901943
C	5.03699505986870	0.17945963300438	15.52120001224169
C	2.42578993668784	6.76024943671208	14.31035260080546
H	1.44120349580436	7.20981934887011	14.32962370624687
C	6.26326891258404	0.46289584601463	16.39112301964837
C	10.68046944401067	3.23016523184659	17.21507010900986
H	10.59487063617476	2.57724873061626	16.35847852602418
C	9.51630183755667	3.73608353900506	17.79289431847179
C	8.54034229781246	1.02295540878485	17.97624933876526
H	9.40434233418705	1.26357147912963	18.57270883557892
C	12.01503695092578	4.44610769335849	18.81126757953790
H	12.98459969874255	4.72469316574236	19.20399062299358
C	4.65933887764124	-1.30683635812428	15.52151631377537
H	5.46320502441012	-1.92795714844674	15.12246105522327
H	3.78045614585885	-1.45555591156182	14.89100475517063
H	4.41588906655157	-1.66397823754986	16.52396442680646
C	8.77949065783783	-1.45471872799912	18.57021055346367
C	9.61103877485118	4.60308398002440	18.87633440806027
H	8.70082980549443	5.00408570478087	19.29968768150374
C	3.55968749587926	7.55032780282941	14.45636268513238
H	3.46169156380222	8.62017060813882	14.59142002158581
C	5.78488370261484	3.47967191424235	9.48197082440205
C	3.83086490468573	0.97622487812983	16.05611519572218
H	3.58907330122933	0.66329705176228	17.07407029416026
H	2.95340431907391	0.79884776537614	15.42778663634654
H	4.04203234915357	2.04232096165358	16.06860852026793
C	6.47573579310007	3.53910029571634	10.84797783666877
C	10.85532188660601	4.95614970150404	19.38239217619651
H	10.91800149764534	5.63599535629911	20.22278706686927
C	8.31289712747939	2.75498202786809	12.28230264928464
C	4.82308376002869	6.97395710956271	14.43598947576541
H	5.71298872107110	7.57403268283660	14.56425644126325
C	11.92128683845650	3.58438769122108	17.72236823237535
H	12.81932025108456	3.19639902979880	17.25820350604090
C	7.62516138250632	2.77298417053645	11.07042505051499
H	7.99374620770311	2.16062405962814	10.26208655767227
C	5.32521760421633	0.59315545528900	14.06476229221218
H	5.59996572319292	1.64147492628714	13.99291726807409
H	4.44256256698617	0.42058901776510	13.44292568267700
H	6.14645529738218	0.00323652846063	13.65618815970541
C	9.29487279418989	-2.44985975350976	17.51332493991555
H	8.47889923861223	-2.84243840896929	16.90698396629028
H	9.80181634574416	-3.29417641419082	17.98925240196229
H	9.99985869111849	-1.95836272102763	16.84145194168427
C	10.74381139290079	2.82088559134296	12.89846136183828
H	10.96379526710352	3.51690141195597	12.08607080812922

H	11.63930121488742	2.22119719348416	13.08430836088604
H	10.51957867347818	3.39981257556115	13.79083708160275
C	9.56367866589836	1.90583541827396	12.51821888960714
C	9.97599808280039	-0.99649945041812	19.41245349535944
H	10.73490182090544	-0.50919836785074	18.79924001967219
H	10.43897993795316	-1.85954917799784	19.89575950423252
H	9.67284987999420	-0.29707524008484	20.19256466113746
C	9.30392409194025	0.89823402697406	13.65528188180164
H	9.00722461510400	1.39821488872331	14.57267097092480
H	10.20649240832077	0.31351365288450	13.85266102842660
H	8.50726221516833	0.20703999247267	13.37779016263476
C	4.53595138595126	4.36730759683162	9.42382359861831
H	3.79529919015239	4.06628075789346	10.16559046050056
H	4.07362293882011	4.28964898320395	8.43734977215294
H	4.78049757404154	5.41595272613177	9.59741629651620
C	7.78785397350218	-2.17446179461041	19.50286218361468
H	7.41160313535425	-1.48846144806513	20.26347925178526
H	8.27165590176837	-3.01596575456799	20.00688299385331
H	6.93066561371197	-2.55947106818587	18.95118723252846
C	6.75607176800778	3.95258950795633	8.38445623735152
H	7.07465299876899	4.97885794852938	8.57360396997550
H	6.27625525520696	3.91609684472236	7.40224258669347
H	7.64950503856014	3.32950472530640	8.34664636949783
C	9.96535134042793	1.11168277660551	11.26931173750706
H	9.18220268502795	0.41533093147926	10.96441251607595
H	10.86298477989891	0.52851603890622	11.48493640670756
H	10.18805548865685	1.76801883388270	10.42598367089321
C	5.35203303894820	2.03151969625392	9.18528266916836
H	6.20660016224320	1.35552187280822	9.16962456749768
H	4.85231121160708	1.96948427197781	8.21440631996782
H	4.66229511354394	1.67505682030867	9.95168845189041

List S45. [Re(O)(ap)(isq•)], B3LYP

C	8.33638727028902	3.41844860378250	4.81485449404973
C	9.19089177457446	4.48912925257540	5.11653324331742
C	10.53709720137137	4.26234398911197	5.37050986714546
H	11.18632849544515	5.09815651611912	5.57749125089831
C	11.03010333904098	2.96225551358822	5.34068603152330
C	10.14721358491163	1.91672594318516	5.05114687652645
H	10.53112449806013	0.91266554722122	5.03494126177125
C	8.79022965539373	2.10093941068223	4.77639722864314
C	9.22141842318674	6.92154227798440	5.36802419095728
C	9.24233459725733	7.95880905325908	4.44140597328389
H	8.77183096304268	7.81536734820408	3.47839451531702
C	9.84801329552204	9.16584749752574	4.76362581525470
H	9.85407238220422	9.96979967803670	4.04005127204297

C	10.43738396851908	9.34305880913219	6.00874857160767
H	10.90509000142607	10.28536522009562	6.25984766560707
C	10.41483675016164	8.30597105742216	6.93491100334875
H	10.85794413520929	8.44163780666269	7.91248109799883
C	9.80592023824618	7.10042868742783	6.62098087700780
H	9.76174278007199	6.30007539879523	7.34598457504830
C	5.14182294758417	7.24625149484004	6.45833297510642
C	4.72559182724301	8.39259669402084	7.13303082628751
C	3.38998021904626	8.41147224162431	7.54139104750628
H	3.03604836801220	9.27978146208474	8.06785865947495
C	2.49226102760060	7.36757384359070	7.29804818452562
C	2.94609263599428	6.24448759458453	6.61347070356734
H	2.28421283500385	5.42062390916315	6.39573957850875
C	4.26953425624646	6.17789028518113	6.20296716157027
C	4.25547408132867	3.85088929878666	5.47296841631791
C	4.00671779230493	3.23501940207254	4.24968959993159
H	4.27983883327204	3.74833752692187	3.33820311407663
C	3.41756751042646	1.97894798057655	4.20901841803116
H	3.23560710414380	1.50551536437677	3.25361691797559
C	3.06086279640639	1.33428224818193	5.38632151774075
H	2.60058349454576	0.35610883770629	5.35325736603313
C	3.30550761923711	1.95039393543281	6.60933021099123
H	3.04124892629741	1.44959221536021	7.53120586792639
C	3.90384654706813	3.20020701468660	6.65566387397552
H	4.10768364890618	3.67923054294251	7.60319063533838
C	7.84810090586470	0.93610350948306	4.46838955448686
C	7.19525285746731	1.14006181005713	3.08784141488000
H	6.61893665429861	2.05984863372283	3.04728900357039
H	6.52291340940051	0.30767884707710	2.87050227842079
H	7.95607091819099	1.17422559888731	2.30617598912187
C	6.76288455930704	0.86146070910535	5.55806115824807
H	7.21572697778019	0.68449147391929	6.53514182940728
H	6.07635265753992	0.04016743193406	5.34708361186650
H	6.18135334961150	1.77656199919039	5.61042785271581
C	8.58668607188765	-0.40798086629404	4.44316537654658
H	9.36649764157609	-0.42978390705455	3.68058130331603
H	7.87484578923438	-1.20141005632582	4.21243398309494
H	9.04016036542515	-0.64276262915247	5.40711303539256
C	12.51752612187444	2.72463401792560	5.61800640222891
C	13.35599005858947	3.46395082675320	4.55835528907149
H	14.42111271571935	3.30702429476495	4.74050185936640
H	13.16746666293055	4.53700583939565	4.57342322728103
H	13.12267522528995	3.09742792814063	3.55771860133874
C	12.86905958493239	3.26690901034972	7.01588189441975
H	12.28635033945210	2.75786744072047	7.78508403472467
H	12.66825348700193	4.33506898170989	7.09596872840524

H	13.92821109796756	3.10888295221298	7.22879964115119
C	12.89357257111199	1.23909954847114	5.57393484880255
H	12.34738231597191	0.66085934065490	6.32043257588507
H	13.95837963039827	1.12719816520735	5.78236319064408
H	12.70121618437787	0.80190967742471	4.59343546464121
C	5.68899544574734	9.55046201553328	7.40156608391913
C	4.99988802404241	10.71121457270819	8.12917222083342
H	4.17633679383123	11.12404536337757	7.54487715699429
H	5.72391769033902	11.51040071704620	8.29229785658167
H	4.61395600467745	10.41090035453854	9.10437934614007
C	6.23836202156106	10.08949310281025	6.06638562256122
H	6.79032521614225	9.32869814235338	5.52217858949913
H	6.91372864503111	10.92655098994761	6.25424075992236
H	5.42377674022782	10.44555199621462	5.43346577845504
C	6.85198105469330	9.05709012223626	8.28346378557655
H	6.47665571577229	8.68902980182581	9.24007356837706
H	7.54336477302828	9.87845323544219	8.48311656734156
H	7.40911924546243	8.25986327760263	7.79977798014342
C	1.03039760072460	7.42068460240776	7.75214722701435
C	0.11151578127680	7.33864133384796	6.51878892180434
H	-0.93649107756489	7.37874400506931	6.82280831872867
H	0.26422425470561	6.41311597145240	5.96420480512274
H	0.30329957821129	8.17045908732629	5.83968156055572
C	0.69821756135977	8.70983188025993	8.51253305137120
H	1.30519165915507	8.81461341789027	9.41270905291951
H	-0.34870867023453	8.69272595861257	8.81828451267111
H	0.84714843637895	9.59522112962459	7.89321728945583
C	0.74247012867836	6.22807522293403	8.68318193008106
H	1.37929903583266	6.26708197761876	9.56836139336454
H	0.92075427930690	5.27568818139704	8.18455043780697
H	-0.29897278131400	6.24510567694559	9.01095995916246
N	8.54063174054737	5.71646754573437	5.05238935981661
N	4.87487824923134	5.12284718102503	5.50578999725809
O	6.23862603704174	6.37664600317977	3.16726959676154
O	7.06266077206440	3.79627998144621	4.54202104419729
O	6.41679907318513	7.05349484723026	6.01660932084163
Re	6.58416699305369	5.67130222313441	4.66924386945813

List S46. $[Re(O)(ap)_2]^-$, B3LYP

C	8.34220816173814	3.36543036241677	4.94681297628082
C	9.20713319990046	4.44814443268642	5.17033699943486
C	10.56959739608205	4.22703278479755	5.30691630477013
H	11.22575563659732	5.07188851803939	5.45343388096320
C	11.08472455621106	2.93008921479714	5.24755947291763
C	10.20109824339349	1.87437161423226	5.04567224926531
H	10.58854518242078	0.87165987273742	5.00222961603277

C	8.81937678920603	2.05725004303408	4.88790287778856
C	9.20256365551026	6.82468042228213	5.66694430130831
C	9.15527042709750	8.01127543338412	4.93432723014859
H	8.62560533757575	8.01197977703597	3.99177948634431
C	9.75305330528291	9.16640134872241	5.41653837397406
H	9.69061964672184	10.07935372585396	4.83801991353104
C	10.42036323518457	9.15914263936314	6.63541242052597
H	10.88253481760242	10.06180487546557	7.01341840168495
C	10.47205618541796	7.98030119208093	7.37265153225190
H	10.97052830212076	7.96398283163903	8.33403061887396
C	9.86798953158029	6.82618212389690	6.89729286968700
H	9.89163343806946	5.91677086517046	7.48123104445053
C	5.08563299345101	7.33162765272219	6.30517715656843
C	4.66594611962310	8.47336793351501	6.98536615723050
C	3.34433810913679	8.48626056483791	7.45513511660812
H	3.00104874841112	9.35809957889155	7.98399169550323
C	2.46349401972044	7.42672706703045	7.26172275891376
C	2.91909861418682	6.29873547700331	6.57580818672227
H	2.26071748197214	5.46164027662419	6.39876689347398
C	4.22414187330910	6.24062101625964	6.10976289722083
C	4.20661856368721	3.93267169426300	5.35724746684441
C	4.06935374860029	3.26689552783357	4.13879700242232
H	4.42477344164028	3.75785124993002	3.24348477054198
C	3.51278051944846	1.99745053020585	4.08338618272274
H	3.42989502597544	1.49388533975451	3.12860604418873
C	3.07157681992754	1.36992185297438	5.24212863131900
H	2.64169589128960	0.37762796718084	5.19900684934684
C	3.20555083114909	2.02636008428448	6.46151923281348
H	2.88569664297217	1.54162892460613	7.37568347070272
C	3.76913434773177	3.29184201599919	6.52097986395324
H	3.89186757544486	3.79138459934725	7.47170321863255
C	7.86197804945833	0.88545397544885	4.65431995410673
C	7.14800997722776	1.06562162649309	3.30098209728570
H	6.59212062829459	1.99788858794382	3.27446083524386
H	6.44980184756942	0.24185717297953	3.12981267227568
H	7.87626430980447	1.07198129369848	2.48717077161927
C	6.82290396122500	0.83060124356475	5.78987831313429
H	7.32076397752189	0.67066480950475	6.74883856873027
H	6.12383151917610	0.00704666256429	5.62571700333408
H	6.25168818376269	1.75172280487881	5.84813592270509
C	8.59250728790073	-0.46329690529890	4.62100866845617
H	9.32921429691231	-0.50688089911994	3.81685299001112
H	7.86587264421607	-1.26029238981566	4.45054534278286
H	9.10159253787112	-0.67324823637703	5.56340310026449
C	12.59558995816314	2.72526603354664	5.39889139107986
C	13.33205659152262	3.47888208171058	4.27553644169739

H	14.41407449250870	3.34889767787863	4.36767838272992
H	13.11604568514101	4.54630414359801	4.30432982363192
H	13.02236310763960	3.10414303068399	3.29867591707156
C	13.05750036556493	3.27401041087650	6.76168972484990
H	12.55384796891726	2.74888036105424	7.57496911851799
H	12.83052782736167	4.33508740111642	6.86105729546274
H	14.13652613262312	3.14515432013865	6.88407192296010
C	12.99877230197239	1.24809886715823	5.32077359034442
H	12.52683719983619	0.66130278789685	6.11004277367181
H	14.08101837431990	1.15496267332249	5.43487610598812
H	12.72431473511636	0.80905666319545	4.36075940817174
C	5.62026368462636	9.65108505796462	7.19958623000661
C	4.95309215221138	10.80884376736926	7.95319097133323
H	4.09277011408313	11.20385175863312	7.40998431908193
H	5.67361150415571	11.62043474630206	8.07336267975766
H	4.62118809082965	10.50932705022715	8.94889289059255
C	6.09100688545281	10.18947697671623	5.83496823967711
H	6.59437202414849	9.41513813609009	5.26433136832584
H	6.78618587986095	11.02107516958222	5.97800208810382
H	5.23875177498897	10.55115984092229	5.25567119240964
C	6.83638205775919	9.18898903469135	8.02431930030910
H	6.51325529820711	8.82587534228884	9.00246387410539
H	7.52751756258313	10.02095972947434	8.17999182815524
H	7.37576171950048	8.39198355848967	7.52182090995418
C	1.01832059777232	7.45034893113116	7.77038746924255
C	0.04908143393249	7.31901234611221	6.58080261538760
H	-0.98884568134136	7.32737497756016	6.92544147000800
H	0.21508185142102	6.39240243372029	6.03220325045712
H	0.18634849972528	8.14647376503043	5.88283656042906
C	0.67953964435815	8.74541609372595	8.51793002831823
H	1.31840089536229	8.88077374191566	9.39168191760896
H	-0.35675139632272	8.71252662318741	8.86127560380380
H	0.79200939917709	9.62044158462629	7.87646101291131
C	0.79347969847667	6.27231151095865	8.73690186142256
H	1.46247195445169	6.35134268854460	9.59543489607329
H	0.98720897106893	5.31611216494034	8.25186931970552
H	-0.23704231215233	6.26413576413963	9.10301830549764
N	8.54624399330097	5.68076383299366	5.17918437931617
N	4.81934887010109	5.19797682012512	5.39244360258184
O	6.47273418758751	6.05440076891113	2.97754337735707
O	7.03776315763471	3.68951670123395	4.80357254054614
O	6.32951777666258	7.18218093818000	5.79779473204147
Re	6.62082733335820	5.61983051467121	4.60954886338878

List S47. [(ap)(isq•)Re(O)(μ-O)Re(O)(ap)(isq•)], B3LYP

C	5.31463128451800	-2.06651298732976	7.46007620566351

C	4.92270350789390	-1.96562926723184	6.12959287661328
H	5.41313982415444	-1.25849408270768	5.47799095229216
C	3.90822686000352	-2.78228095895578	5.65408236455725
H	3.60033417619728	-2.69879345962674	4.62083042954339
C	3.30010937379343	-3.70853026464384	6.49417672879946
H	2.51564566716541	-4.35005451450495	6.11580930506837
C	3.70754237120514	-3.81328625602604	7.81902238609820
H	3.24729964514745	-4.53996384606782	8.47506582765159
C	4.71062438647387	-2.98908033808347	8.30810727309719
H	5.03955435597530	-3.06732955518288	9.33286719102526
C	7.15851203537488	0.41492617471322	9.36160012542298
C	6.04085170386458	-0.35065432522058	8.95404810453759
C	4.78788782700085	-0.16727973231739	9.55968795146490
H	3.94491095064682	-0.73166437456932	9.20411009004872
C	4.66118208218362	0.74051773412071	10.58330751281249
C	5.80529514809279	1.46838127637194	10.99184624623787
H	5.68517761752765	2.17090416420068	11.80088580813769
C	7.05305851723220	1.34991818221806	10.41471442938770
C	3.33255803137975	0.99045266103056	11.29689474241973
C	2.19455715394005	0.15057766460267	10.70700629366726
H	2.39470666018778	-0.91815513345204	10.79325212587443
H	2.02548342584745	0.38245186810564	9.65482487209602
H	1.26921308695014	0.35901081102301	11.24515314722811
C	2.95046526772766	2.47642108974826	11.17084602865129
H	2.83342223384536	2.75657210351198	10.12384362444188
H	3.70613433331348	3.12838568683078	11.60806593591576
H	2.00576416803003	2.66977943936942	11.68246516823204
C	3.48450886194088	0.62344778190222	12.78506468729088
H	3.75509417745044	-0.42766014839750	12.89542981309956
H	2.54557221017943	0.79277689787206	13.31574121571432
H	4.25547671121376	1.22096132093920	13.27134676332954
C	8.26705358446504	2.16365855119518	10.85680798241869
C	7.92180373222368	3.12380012440631	12.00021165755438
H	7.57577464407476	2.59347629764239	12.88911081659989
H	7.15502444406139	3.84223060878836	11.70625616737518
H	8.81327485559984	3.68681747389522	12.27819168609916
C	9.37975999407122	1.21454486488526	11.34320476211607
H	9.70203631703873	0.53923958637303	10.55452614084879
H	9.03688017762220	0.62165956910666	12.19343282657430
H	10.24526694745729	1.79453777065771	11.66878119279999
C	8.78143270534868	2.99663767186275	9.66842215791658
H	9.07511659468121	2.36328631057596	8.83671529482091
H	9.65016387553043	3.58289472718557	9.97342143091909
H	8.01253957199287	3.68590784600094	9.31823505547723
C	8.13294343789381	-3.10365872578973	9.74259443212023
C	7.79489877250606	-2.43662337251858	10.91844088284584

H	8.20717197627078	-1.45772237035823	11.10674069001994
C	6.93134411128875	-3.03151314112693	11.82824430725468
H	6.65773014431204	-2.50081445278741	12.73042285105192
C	6.42166897127720	-4.30196197354758	11.58286490104222
H	5.75214456065840	-4.76495134057122	12.29534913379669
C	6.77567998324624	-4.97495109865031	10.41865980202531
H	6.38034894083227	-5.96229219910027	10.22054028588648
C	7.62100376408928	-4.37480909846164	9.49535564715592
H	7.88161968569394	-4.87117575384382	8.57123978838333
C	10.34538770208306	-2.56326963977839	8.92691871521998
C	11.05275085605728	-3.24243415168380	9.90850322533925
H	10.51303160128076	-3.81315990653846	10.64916275904721
C	12.44539667708361	-3.17304287491350	9.91974656903240
C	13.08905410119779	-2.41722581054443	8.93768425898101
H	14.16300225146302	-2.36493395840915	8.95002187166726
C	12.40894966510431	-1.71600401924829	7.93504050398896
C	11.02245696936236	-1.81411735421561	7.95724474094375
C	13.21578091634513	-3.92710404329175	11.00818758332646
C	12.75972237500641	-3.43127463556187	12.39339914880877
H	11.69451233738297	-3.59894264012195	12.54853404617808
H	12.94887576032760	-2.36226826138921	12.50128288057554
H	13.30090586158632	-3.95710661513172	13.18285660677144
C	12.92420404539578	-5.43423833908603	10.88674471531561
H	11.86119932079370	-5.64664417127849	10.99776665479952
H	13.46119052410159	-5.98977891276590	11.65868350323383
H	13.23944352319856	-5.81035589934051	9.91236351918602
C	14.73149773381361	-3.72336567432356	10.90502794254375
H	15.00547832560319	-2.67258752306703	11.00856009645871
H	15.12573012339389	-4.08719032830713	9.95530211215556
H	15.22902903602294	-4.27630075867917	11.70311907240890
C	13.12372256492850	-0.89130170742356	6.86482371943381
C	12.65441782025282	0.57433257899431	6.93650852511772
H	11.58353507722488	0.66097590758168	6.77417531007351
H	13.16150907453049	1.16771443004319	6.17480806913028
H	12.88790191836436	1.00089642383392	7.91364618975515
C	12.80228645396844	-1.47245113276543	5.47494716799759
H	13.13136209610275	-2.51063008335985	5.40659537685537
H	13.31407026068015	-0.89886482933494	4.70163310760328
H	11.73629822545992	-1.43710644669595	5.26672958900981
C	14.64572727023920	-0.90883347064256	7.04743057823944
H	15.05521237559292	-1.91705696672294	6.97140981173731
H	14.94250952349600	-0.48840611578331	8.00928825937275
H	15.10797038950366	-0.30419329268621	6.26631830686039
C	9.81024706214708	-0.86822705027508	2.56826029470860
C	9.68297812587917	-2.13096365544501	3.13670428669816
H	9.46971136335191	-2.22632438774096	4.19054350453386

C	9.82597306089399	-3.25555498675969	2.33856418517881
H	9.73256694657402	-4.23690895391706	2.78308919044167
C	10.07516964061743	-3.12347364746778	0.97698941330812
H	10.17615108042688	-4.00380073247084	0.35656043155209
C	10.18674841355775	-1.85768255399960	0.41372387400823
H	10.36837261147956	-1.74710100991469	-0.64697835324738
C	10.06130718846445	-0.72668329251851	1.20740364474915
H	10.13878329166580	0.26038778149431	0.77810926693419
C	10.74944018550417	1.11486569603403	3.51229552619714
C	12.01212535292124	0.95526875345709	2.91983124568640
H	12.20420214327922	0.08369208733006	2.32052085340735
C	12.97988034296274	1.90762253417232	3.13097762408230
C	12.66903929002791	3.02314609337014	3.94619134217424
H	13.43977809924823	3.76255772310854	4.09341010255692
C	11.45569198528568	3.21791015805191	4.57354112738824
C	10.47803331635668	2.22506056906476	4.34611200525625
C	14.37403110199398	1.81071606685980	2.51195880782779
C	14.56390646484447	0.51505841750967	1.71613867873456
H	15.57280738969746	0.48486393640849	1.30312597543804
H	13.86354217801336	0.44362796173912	0.88291732046773
H	14.43661909058390	-0.36681805087650	2.34501643801600
C	15.43990737391962	1.85293726153170	3.62236787672001
H	16.43963965097419	1.79728572880604	3.18776635210900
H	15.31983585765038	1.01239945938351	4.30629177388387
H	15.38155353670715	2.77034341695148	4.20706754909706
C	14.58146249503677	3.00164864549471	1.55715136006812
H	15.57167910127671	2.95382836048709	1.09979240914672
H	14.49983223946317	3.95529883491307	2.07871306175843
H	13.83533515747612	2.98958892040132	0.76136936780459
C	11.15037966098991	4.42059145743136	5.46349139744848
C	10.76122855226178	3.93235908188197	6.87093706368634
H	9.87937730884370	3.29982934304420	6.84163080183537
H	10.54657431155876	4.78769178473621	7.51399016065703
H	11.57459305442800	3.36351088556379	7.32243178558930
C	9.98703476887934	5.23001387369099	4.85750005932359
H	10.25036459498103	5.59764432884819	3.86370539083245
H	9.76923764144962	6.09522177298585	5.48646000605709
H	9.08145557763346	4.63308040770582	4.78037673434331
C	12.36151383511860	5.35001523153979	5.59733005837606
H	12.66799691706098	5.76391882748025	4.63509565380016
H	13.21696217074330	4.83810236325753	6.04098063161285
H	12.10320413437623	6.18608737329034	6.24794234200493
C	7.95589601112432	2.30349720563570	1.68403014147047
C	9.00675503648083	3.20949192481957	1.55405342503870
H	9.30336026886868	3.79909206363917	2.40720173312497
C	9.66200497199736	3.34017296097490	0.33723257934163

H	10.48861405084188	4.03237037669932	0.24741614750235
C	9.25800922070406	2.58779414317077	-0.76029635677621
H	9.76766372767577	2.69447085133549	-1.70852769067326
C	8.19629575116015	1.69869478474817	-0.63477585561803
H	7.87878684068521	1.10870398965798	-1.48415732242129
C	7.55212425871420	1.54880545953562	0.58580521859654
H	6.74492329456567	0.84036088871541	0.70750343688001
C	6.29675550081329	2.98670270023744	3.30689437343818
C	5.76323781244224	4.03329095312656	2.55882936698164
H	6.13447410600668	4.20795898514220	1.56236751340344
C	4.77077928074975	4.82860366588215	3.11867994613174
C	4.33692815054979	4.55016386684553	4.42193007359377
H	3.56845911224021	5.17510993874885	4.84636461624587
C	4.85030128231081	3.51468342566801	5.20190945170743
C	5.84295578906141	2.73913099101164	4.60393176308168
C	4.37273772015090	3.22311443796012	6.62421765165630
C	3.78354212815941	1.80108723335435	6.68344724679197
H	2.94183162756608	1.70506080057148	5.99571580027756
H	3.42960873472852	1.58477925129804	7.69196145216045
H	4.52558423595272	1.05155219519501	6.42194682049174
C	5.55697146955784	3.33559008780829	7.60288615724509
H	6.35413006831425	2.64355977103450	7.34647501890528
H	5.22742985977355	3.11093632678497	8.61792228674136
H	5.96624324930359	4.34741039057396	7.59200493775921
C	3.28703806744968	4.20596556552932	7.07664331912793
H	2.39815958316298	4.14811193386492	6.44681577114879
H	3.64567338854355	5.23619240772148	7.07213082666417
H	2.98554755461866	3.96607621047763	8.09687968210222
C	4.14982828085171	6.00505774543396	2.35979071681645
C	2.63005716595287	5.79382837441557	2.23110174344161
H	2.14752205125534	5.73238681843142	3.20607814631789
H	2.41300305988520	4.87046891431369	1.69220201889119
H	2.17639865473210	6.62355349687861	1.68467769753193
C	4.42043847734408	7.30921793859840	3.13311545423273
H	3.98919718579692	7.27961529518911	4.13330574417114
H	3.98801908625339	8.16171641570997	2.60484015090671
H	5.49311157354215	7.47862977308108	3.23697551510714
C	4.73112346066683	6.15774722968679	0.94945730214390
H	5.80588121594650	6.34216004074940	0.97389150140619
H	4.26077212810634	7.00553914290827	0.44925937837168
H	4.55053736160865	5.27072654326047	0.34093574171661
N	6.35172811723172	-1.21676660051136	7.94394737007130
N	8.97522643483692	-2.49524879163958	8.77800471635385
N	9.67175251325400	0.28236149974597	3.39844323147910
N	7.30249080004094	2.12087606866982	2.92923577615692
O	8.25496099919003	0.14556621261440	8.71658658061473

O	10.19627096371720	-1.19412862548167	7.08647271618844
O	9.26920055722385	2.25275542481911	4.82380756790779
O	6.47786363604337	1.71155892108750	5.20763240612002
O	8.24736356936448	0.06157167490085	6.01781744618160
O	7.75513403811959	-2.59963382514967	6.27247280429334
O	7.24779958219351	-0.59642787822504	3.57351481756102
Re	8.26852165254618	-1.33136797502702	7.29014970937096
Re	7.92919340777232	0.78565029570664	4.30494493557746

List S48. O_2, PBE0

O	0.00000000000000	0.00000000000000	-0.09646954430361
O	0.00000000000000	0.00000000000000	1.09646954430361

List S49. [Re(O)$_2$(ap)(isq$^\bullet$)], PBE0

Re	7.21072045947458	4.55264376307207	15.93467407600598
O	8.24929921872807	3.62671888924849	14.48915655348858
O	6.20976406914653	2.69326641292189	15.92196955439684
O	8.40590229078176	5.73726659440493	16.17717208968634
O	5.90016126952375	5.17771603677283	16.81653996867967
N	6.18082084215817	4.90989688260750	14.26509560265159
N	8.29820527789143	3.29522773918771	17.17916887377028
C	4.93500325348916	5.58417215258891	14.26977082461766
C	3.76181152395605	4.87349826990102	14.03980025446318
H	3.81678352269673	3.80702199902756	13.86163359170642
C	7.73495293476689	3.51577444761327	13.28203085322108
C	2.54431594154413	5.53046719023086	14.05898913708229
H	1.63163686134518	4.97178828106334	13.89034157605843
C	6.76024116944433	1.69504658976831	16.49900337794433
C	7.94419627471629	1.99900090450076	17.24747035351994
C	5.94038124131587	4.32814112797847	11.86474696529758
H	5.06015751981756	4.94061701724588	11.74445699775651
C	8.13376282461221	-0.29276447061554	17.91293822853957
C	6.97852520091535	-0.57622190482580	17.13119317585555
H	6.64012063552352	-1.60207263149616	17.09959981225037
C	6.57077802817790	4.27123692536084	13.10430016712676
C	5.03820138562878	0.03893580311603	15.59970499801114
C	2.49018621584704	6.89552553855372	14.30448369947652
H	1.53584109919565	7.40727021535722	14.32030022960560
C	6.27172571142366	0.36257138117797	16.42704960800318
C	10.72290746612027	3.18494814860383	17.20212253342201
H	10.67689372199595	2.49589737309860	16.36812945520416
C	9.54153081593366	3.69241165332253	17.73010071306874
C	8.60641394406972	0.99221581898734	17.96754917031364
H	9.47864672902420	1.25070237436928	18.54734736456125
C	11.97728650848232	4.48678505529691	18.78451954996383
H	12.92890892530046	4.80002049249438	19.19564332575625

C	4.68976583800976	-1.44353203040643	15.67268614468867
H	5.49361691076015	-2.07083521551555	15.28048965409674
H	3.80058148700837	-1.63274874726917	15.06875114113175
H	4.46851915851471	-1.76090665721312	16.69442113514592
C	8.80534443523195	-1.43835578628724	18.65064338576183
C	9.57611158416993	4.60581014244812	18.77323872004414
H	8.64698838526284	5.00766956752395	19.15643076647931
C	3.66113975514375	7.59931092943797	14.53862475599595
H	3.62558099399084	8.66332274251678	14.73775593395549
C	5.79783540390229	3.57601596548251	9.43923642076982
C	3.84029260244861	0.83840471063785	16.12627753172094
H	3.63108486742672	0.58053616119421	17.16681019488843
H	2.95265997607804	0.60141765606763	15.53463895361822
H	4.01639074418319	1.91076094631508	16.06788579857922
C	6.44991834358378	3.58432541643217	10.81603450836358
C	10.79523164208543	4.99565269910157	19.30168556424008
H	10.82080073369583	5.70678783395162	20.11804026663600
C	8.27328546580764	2.74830409032647	12.24120627136409
C	4.88337205545395	6.94682397100149	14.52344479611292
H	5.80464365431135	7.48337873841649	14.71206317338244
C	11.93789488514908	3.58530347900209	17.73084685489966
H	12.85806338486324	3.19801799952117	17.31072626518458
C	7.59886421494643	2.80910525644401	11.03238807062902
H	7.98173677846975	2.23021110952881	10.20366777321050
C	5.29549304657431	0.39466209544004	14.12995088259504
H	5.54082916645646	1.44719954089489	14.00150599332913
H	4.40394172242484	0.17501175766180	13.53752791107135
H	6.12001403649988	-0.19758227752068	13.72847995036498
C	9.27693879129404	-2.47949006937839	17.62928183542197
H	8.44843048181120	-2.87757995391410	17.04163190984013
H	9.75469978237635	-3.31700615209897	18.14295013014454
H	10.00064414930452	-2.04435441075821	16.93733689753343
C	10.69631737591880	2.84752992238501	12.81425656320124
H	10.89280062207136	3.55167503194994	12.00293345886310
H	11.60384691163187	2.26027157548564	12.97920060355244
H	10.48568199650295	3.42033317919439	13.71637366201879
C	9.53171604959004	1.91914110258754	12.45242292806815
C	10.01119450874140	-0.97319239609263	19.45848297196637
H	10.77869474662000	-0.52824758322857	18.82186823676476
H	10.45908966723642	-1.82724748647065	19.96975191257842
H	9.72935887278204	-0.24161886391294	20.21837738460038
C	9.30788299064945	0.90926458620896	13.58381247303429
H	9.06478720703966	1.40691951714352	14.52094300631198
H	10.21169857593058	0.31281650944938	13.73378773267000
H	8.49020519960488	0.22897149367655	13.33802830891787
C	4.55807431147611	4.46197512295629	9.38633335759344

H	3.79544536466126	4.13286527699751	10.09539138462892
H	4.12125773906131	4.42134746563610	8.38639414303283
H	4.79757341132974	5.50533978931479	9.60150069201857
C	7.79902656255395	-2.08309540297669	19.61016649786197
H	7.44672994814753	-1.35944422838951	20.34775125081648
H	8.27006584726329	-2.91255021101350	20.14271678474119
H	6.92841104444222	-2.47736230575531	19.08439727634006
C	6.79757976941590	4.08735918723388	8.39763722595080
H	7.11125170258125	5.10676927755301	8.63100086648760
H	6.34241575274209	4.08775781217650	7.40383822059228
H	7.69207272452961	3.46402927070747	8.35577261286895
C	9.91419123632331	1.14088750343020	11.19814607144151
H	9.12897908533682	0.44296410892401	10.89869634609076
H	10.81747028424623	0.55995004510452	11.39569856892959
H	10.12538473006572	1.80451415019296	10.35703412520715
C	5.38054194910878	2.14566274094055	9.08421772203086
H	6.23538970210806	1.46798877592550	9.06947434160588
H	4.91511664361640	2.12083945384351	8.09550172891930
H	4.66171410638352	1.76095599890199	9.81073426758711

List S50. $[Re(O)_2(ap)_2]^-$, PBE0

Re	7.25683840472079	4.53392989395292	15.94687240825506
O	8.31473907520443	3.60995608922959	14.45891133743892
O	6.24978606156847	2.75319564394966	15.91280272835152
O	8.50938843737765	5.67851746581558	16.14070901888783
O	5.97222334686860	5.26676467831453	16.80075486001608
N	6.23865424105855	4.94378630153554	14.25129859492587
N	8.27471618051236	3.33591068320596	17.21358022368645
C	4.95114520185989	5.51703852845258	14.27625389983550
C	3.82149602192443	4.72628342913856	14.08203347637505
H	3.95159913867434	3.66339999327454	13.92197688727030
C	7.79281827251889	3.54232556847323	13.25609968408362
C	2.55944597845172	5.29279594200987	14.11718630805994
H	1.68705544170688	4.66451483349513	13.97758095805516
C	6.77019944058472	1.71425168271814	16.52347312783975
C	7.91136329748651	1.99990618765382	17.28254604712997
C	5.97935363605187	4.34143288915809	11.85850485748323
H	5.08776844161738	4.94204182155458	11.75673062480501
C	8.06419116490518	-0.29883826444643	17.96476917578455
C	6.94018670804226	-0.56450765530441	17.18130784100653
H	6.57376651925803	-1.58170289856945	17.13936001940695
C	6.62243253495183	4.29266840805877	13.08947180475376
C	5.04241499334866	0.10649545046578	15.59885442890196
C	2.40812801728534	6.65393250901295	14.34550810155419
H	1.41898159182910	7.09578852847767	14.37570915537906
C	6.26698314164720	0.40956061780291	16.45160955830540

C	10.69739654472862	3.12131589572281	17.19679768238500
H	10.60578441453642	2.41277899306712	16.38326555687631
C	9.54031107299710	3.68920076459773	17.72365345015706
C	8.54540263505169	1.00241988217161	18.01361344902748
H	9.41151590946335	1.25693705575237	18.60634673765582
C	12.04091991404038	4.40378369095623	18.72431722931575
H	13.01351976663545	4.68429132993268	19.11147640922960
C	4.64729423757692	-1.36500701743313	15.66677033063692
H	5.44325880055543	-2.01532073607786	15.29600779042565
H	3.76508910689092	-1.53010764239482	15.04320236027102
H	4.39909042684895	-1.67207028659197	16.68577945057284
C	8.73307555311390	-1.43938698190124	18.72471261278756
C	9.64559787114835	4.62106637628021	18.74827640679586
H	8.73962521575025	5.06996334854473	19.13499519145265
C	3.53194207064650	7.44089674713066	14.54667498559722
H	3.42431740025244	8.50282173353471	14.73601011619612
C	5.80357958777159	3.60614648360551	9.42337870895525
C	3.85427002044422	0.94018564569333	16.09147226312792
H	3.60337861249389	0.67769090771431	17.12228194573881
H	2.97594834437739	0.74836508030332	15.46695743476083
H	4.08244987125770	2.00408908718794	16.05631450663574
C	6.48172347899567	3.61272084591333	10.78925283749865
C	10.88972678722886	4.97572635682093	19.24448349076698
H	10.95914932587314	5.70756065835265	20.04115681695205
C	8.31943581145517	2.79496493426815	12.19525931530428
C	4.79721112196177	6.87707438976038	14.51217179837487
H	5.68238289485412	7.47681026494157	14.68146598173881
C	11.93828497077925	3.47768857376501	17.69498542778835
H	12.83248569152073	3.03800067936902	17.26793895389445
C	7.63750122805204	2.85510686109865	10.98442651755047
H	8.02053279383803	2.28170844167394	10.15068513402910
C	5.33758291757381	0.44937050953936	14.13434967542520
H	5.62698305062798	1.49238806610311	14.01843322688159
H	4.45243709760659	0.26260507420017	13.51836125621570
H	6.15243691673193	-0.16979100196537	13.75323117309691
C	9.22796229473116	-2.49508735431320	17.73132098353561
H	8.40582921405911	-2.89624940417764	17.13669202506311
H	9.70511357612300	-3.32763169163968	18.25806309182890
H	9.95452191685327	-2.06082800744338	17.04143707050010
C	10.73809868495353	2.87247281537121	12.80352853134201
H	10.95058397732537	3.59705416868402	12.01337369477077
H	11.64143640942531	2.27783477921930	12.97254285534303
H	10.50468418469327	3.42031291483262	13.71514219723075
C	9.57663861953455	1.95965830509530	12.39557717369603
C	9.92780917980383	-0.96276512646140	19.54393363355853
H	10.69860343748790	-0.51849374562925	18.91080406407581

H	10.37390963240255	-1.80951390344004	20.07217997403100
H	9.63246344101005	-0.21999204255524	20.28783890536285
C	9.33431159695959	0.91614335655915	13.49168785894812
H	9.04185986376715	1.38512817300385	14.42932746154059
H	10.24368846166597	0.33130280440658	13.66121945200725
H	8.53862553779682	0.22860533391034	13.19716626931591
C	4.55353326425717	4.47917790068031	9.39726380925853
H	3.81109047794431	4.13937926700516	10.12227769372736
H	4.09694238510796	4.43966950362643	8.40477066411942
H	4.78789274384622	5.52261529748802	9.61774734009209
C	7.72720092340420	-2.08438623246588	19.68305569261154
H	7.36491021068941	-1.35238534553521	20.40798082969507
H	8.19065663779347	-2.91204959296250	20.22922913213717
H	6.86160924147277	-2.47590294248030	19.14673092002086
C	6.77338143962717	4.13266190967306	8.36137225763152
H	7.07744594110939	5.15525829395987	8.59467436345575
H	6.30298454017975	4.12898362801499	7.37295423456610
H	7.67608531929474	3.52205192721484	8.30892750725616
C	9.98125332263435	1.22065397618265	11.12417260148059
H	9.20388199184701	0.52691653961236	10.79498616136130
H	10.88647202429000	0.63953281614809	11.31765782091766
H	10.19594694917511	1.91100244070494	10.30470748305999
C	5.39105534415835	2.17570091895380	9.06331127750390
H	6.25374612417189	1.50844061342509	9.03393551431323
H	4.90654063986061	2.14907463075830	8.08209476052995
H	4.69267769740709	1.78094773550589	9.80420834239897

List S51. [Re(O)(ap)(isq•)], PBE0

C	8.32924996787469	3.42515392354999	4.82287665812179
C	9.18151977572996	4.49124698342756	5.12137836655313
C	10.52384954811531	4.26788293389623	5.37561491738689
H	11.17550138674838	5.10597494477045	5.57981988661210
C	11.01436295396054	2.96827643514438	5.34948157035122
C	10.13442083211502	1.92645384849834	5.06259450801097
H	10.51829462953333	0.91911696572798	5.04787712378499
C	8.77943757897265	2.11024120454740	4.78655932232420
C	9.20805485520791	6.91272222202051	5.37343385110710
C	9.22505115436118	7.95454392096353	4.45576566171388
H	8.74433866852736	7.81856059851664	3.49448437357739
C	9.84198335245371	9.15260746325746	4.77845464040992
H	9.84556028024246	9.96235090795878	4.05926998114567
C	10.44830240686224	9.31569721632110	6.01391304334279
H	10.92853932154683	10.25317872930342	6.26520550008289
C	10.43055605839356	8.27424067360712	6.93089879202690
H	10.88977444209862	8.39920123310833	7.90399229494813
C	9.80911090399920	7.07822136746144	6.61783745962101

H	9.77040155010600	6.27019466464613	7.33748539688020
C	5.14766137175520	7.24643330415761	6.44110686188402
C	4.73363094567561	8.38823708600915	7.11786010768087
C	3.40225607578650	8.40149469140262	7.53378313900589
H	3.04800402811988	9.27114289020868	8.06399442701302
C	2.51009144050694	7.35781826002790	7.29735275753657
C	2.96258405265294	6.23717783573049	6.61174730886857
H	2.30135812114566	5.40822278136587	6.39851722817231
C	4.28063893999024	6.17860736513949	6.19501906857983
C	4.26544958732115	3.86564340057035	5.45944724902253
C	4.01526683771238	3.25546934636100	4.23663716321134
H	4.29451384632642	3.77035435509562	3.32540817092465
C	3.41461270974911	2.00784327562645	4.19225227083359
H	3.22943409698381	1.53793816432323	3.23414695258822
C	3.04779413408486	1.36694531385827	5.36492941935534
H	2.57576514544514	0.39300095729696	5.32872593427837
C	3.29392456715816	1.97739054436457	6.58720598639320
H	3.01999416614213	1.47796218952279	7.50857588548254
C	3.90356731362039	3.21842986582012	6.63785756311026
H	4.10771938937615	3.69648406807302	7.58786625342223
C	7.84605517425397	0.94808169891059	4.47683680965011
C	7.20612069509786	1.14749262781588	3.09901217505220
H	6.62862976420805	2.06862191799281	3.05213737073306
H	6.53383694208281	0.31404657956541	2.87999862258805
H	7.97162415174648	1.17983459524452	2.32062326739647
C	6.75910859614801	0.87200206404669	5.55289754707389
H	7.20497334942346	0.70734804529302	6.53631171793864
H	6.08275875858161	0.04047161640973	5.34190573857522
H	6.16467584580997	1.78247231805213	5.59308856950541
C	8.58601828490374	-0.38590658535655	4.45964458628265
H	9.37162267583419	-0.40708037834664	3.70127238694716
H	7.87713875110237	-1.18252268672462	4.22543221174602
H	9.03375416314722	-0.61761004368172	5.42841191897738
C	12.49414440045986	2.73168955695391	5.62900394197475
C	13.33060967199282	3.46896928310720	4.57878020761247
H	14.39627787164299	3.31587512696487	4.76725414195159
H	13.13818589051277	4.54268821919658	4.59225028787165
H	13.10411844867987	3.10132452470549	3.57593089833565
C	12.83896797103120	3.27008578007480	7.02079801394139
H	12.25630323226658	2.75811306683144	7.78947253982045
H	12.63402504151023	4.33884966245179	7.10151681805643
H	13.89941012499889	3.11573188693448	7.23549520732193
C	12.86583883157164	1.25366437686398	5.58391896098817
H	12.31751158414722	0.67467544023737	6.33007771611311
H	13.93134931270515	1.14009527214088	5.79388493081381
H	12.67430465662770	0.81757567923688	4.60135608248992

C	5.68562114313843	9.54711045989592	7.38219115261458
C	4.99534144004348	10.69212871119215	8.11639354966416
H	4.16364284596004	11.10184389284510	7.53926578209061
H	5.71519048772101	11.49701171239144	8.27785263523680
H	4.61906287472284	10.38392143799549	9.09430012460183
C	6.21590514358843	10.09259090638075	6.05182231487069
H	6.76998324772549	9.33665097329306	5.49898361703921
H	6.88826085148353	10.93392442239432	6.23819235009661
H	5.39355655732096	10.44657143602751	5.42624984815883
C	6.85370570782450	9.06262936470510	8.24720219565748
H	6.49118242573205	8.68878510447110	9.20751530504003
H	7.54115672687842	9.88999502066986	8.44178340921203
H	7.41431747409665	8.26984478146750	7.75567844254561
C	1.05857420700735	7.40325321380361	7.76135440703088
C	0.13413291384728	7.30251262316335	6.54440818148368
H	-0.91211998396561	7.33026150206259	6.85942679780169
H	0.29320671694836	6.37453980146782	5.99314623249517
H	0.30708622665731	8.13298611564686	5.85701982007686
C	0.72734761729921	8.69011844927808	8.50907540412767
H	1.34045323140498	8.80527804568752	9.40533865459194
H	-0.31842997697247	8.67040978326486	8.82255125524923
H	0.86790805884610	9.57190417606884	7.88047904707514
C	0.79172673765901	6.22224868713281	8.69960385653463
H	1.43378269125549	6.27584473134362	9.58139894150661
H	0.97837624122402	5.26665156253938	8.20704006172818
H	-0.24900544663900	6.22999987090122	9.03356858979205
N	8.52828911845510	5.71509411278458	5.05410230204503
N	4.88798310059988	5.13006869711294	5.49649995317897
O	6.25790245765408	6.34210443242599	3.17290912266291
O	7.06113748333278	3.81082376618217	4.55073373942466
O	6.41416647094625	7.05233839977635	5.98824400926055
Re	6.59055458131520	5.66455222902580	4.67715913198299

List S52. $[Re(O)(ap)_2]^{-}$, PBE0

C	8.33026112837140	3.37329585192487	4.95409153157725
C	9.19444463300049	4.45317824975196	5.17691550803989
C	10.55501129731042	4.23511714125855	5.30948584581595
H	11.21243703192471	5.08278834715084	5.45360002619735
C	11.06961592441693	2.94124673025863	5.24979359756532
C	10.18844753612894	1.88730246327251	5.05106360672865
H	10.57763520500920	0.88204098239856	5.00760147449097
C	8.80923166883643	2.06773207189777	4.89556532524028
C	9.19262368267617	6.81697393639930	5.66814595528893
C	9.13355989328069	8.00382234991804	4.94184840342537
H	8.58470181231659	8.00624380079738	4.00778491008793
C	9.74442914110384	9.15338603085954	5.41318059816619

H	9.67419881197096	10.06858332976493	4.83645081936467
C	10.43659388815541	9.14074226523613	6.61465975175909
H	10.91224820034370	10.04139230687530	6.98436675557914
C	10.49974076205780	7.96222799416038	7.34576622508980
H	11.02048124418748	7.94081164868066	8.29667129850171
C	9.88342867426550	6.81332809125615	6.88157355475174
H	9.91781311641511	5.89988571755279	7.46222140558733
C	5.09786779229514	7.32120387587434	6.30721271236909
C	4.67675293893383	8.46012622612384	6.98755965050399
C	3.35690035700611	8.47261898820035	7.45301449286686
H	3.01220563708714	9.34645226307082	7.98379774662268
C	2.47749409550648	7.41688947448022	7.25629234160389
C	2.93261308346861	6.29136308821540	6.57210954543203
H	2.27227927000359	5.45317059859547	6.39113259468715
C	4.23681529512073	6.23345034948350	6.11181763152189
C	4.21725253442243	3.93848559314865	5.36343704436337
C	4.09258309940177	3.26768023061232	4.14937569481588
H	4.46654303432869	3.75309004767970	3.25595346718216
C	3.52319749298913	2.00694727990927	4.09236334503941
H	3.44800482265949	1.49922920270620	3.13753297883986
C	3.05655134315885	1.39277841481706	5.24473100085611
H	2.61340174723295	0.40499161121702	5.19989218929930
C	3.17843074910830	2.05348546478662	6.45962345086171
H	2.83656999661381	1.57816538689589	7.37233215528661
C	3.75424996857515	3.31033422189595	6.52128714967000
H	3.86631175118450	3.81626715765026	7.47213492306536
C	7.86186125859429	0.89667150132505	4.66304752505395
C	7.15751864657658	1.06937166834053	3.31333311426576
H	6.59965751099023	2.00276630518026	3.28171581730182
H	6.45971320381502	0.24353325316137	3.14366916126229
H	7.88879369077848	1.07348211199078	2.50107529147428
C	6.82267215690366	0.83972849866989	5.78713513747819
H	7.31592393322317	0.69496876571719	6.75178296707343
H	6.13545713770451	0.00428842388593	5.62488854647297
H	6.23685981522426	1.75524914230298	5.83274982100856
C	8.59643471082415	-0.44066381775686	4.63751801657936
H	9.33667839786140	-0.48348034382719	3.83490754099723
H	7.87312463182132	-1.24217998188781	4.46739466468798
H	9.10341040405395	-0.64443260417503	5.58383158872708
C	12.57376292672975	2.73880900655423	5.39800426802604
C	13.30285997310467	3.49034479450098	4.28005151841251
H	14.38635774247730	3.36618309390652	4.37379916164759
H	13.08028443982489	4.55781623484460	4.30771418496633
H	12.99560382036646	3.11288024439896	3.30238932659044
C	13.03375446049210	3.28530002852689	6.75283267175720
H	12.53454654639994	2.75685377798128	7.56801089354752

H	12.80109190728123	4.34651453663291	6.85351752786600
H	14.11475013677235	3.16183049995315	6.87183743050760
C	12.97318493895729	1.26933559240581	5.31879625405084
H	12.50156978821874	0.68232335803643	6.10984104856162
H	14.05661706346188	1.17542774943497	5.43064042464389
H	12.69564372620441	0.83090873785003	4.35793480858038
C	5.62196440791572	9.63541523193611	7.20558372069189
C	4.95249831684696	10.77932837384809	7.96150382436463
H	4.08947482145359	11.17401564391320	7.41988847539786
H	5.67080950249570	11.59372781774655	8.08535434090132
H	4.62253541961694	10.47235399715336	8.95695245837656
C	6.08552321424226	10.17852205767057	5.84999238602714
H	6.59026385055830	9.40563956997928	5.27464231907545
H	6.78081772831486	11.01089876838788	5.99617351300299
H	5.23125042750590	10.54153992199470	5.27290190902506
C	6.83536434450498	9.17868803891814	8.02179355430427
H	6.51665414327327	8.80466662938725	8.99819306484020
H	7.51991147475208	10.01660827562468	8.18293634086156
H	7.38253882507457	8.38906920016443	7.51108151444459
C	1.03781860309007	7.44115673887384	7.75875669185656
C	0.07758134285146	7.30978101911648	6.57285417563373
H	-0.96193406501124	7.31283038573191	6.91575363392626
H	0.24930916080513	6.38394939213451	6.02207622042417
H	0.21310378235818	8.13970574802044	5.87596106569159
C	0.70198083809176	8.73045216800645	8.50049467365440
H	1.34027837701005	8.86529054701188	9.37630823211551
H	-0.33605535999366	8.69933384705633	8.84198627081114
H	0.81749741262008	9.60541203307638	7.85730768252476
C	0.81226088962768	6.26912475849166	8.71895055081322
H	1.47747001483896	6.34895517206203	9.58156057768362
H	1.01109213783477	5.31261282634669	8.23335281255167
H	-0.22111520625542	6.25863367425495	9.07974517219968
N	8.53224846234998	5.67871034964743	5.18867157634182
N	4.83390169766536	5.19561577954012	5.39986786475211
O	6.47423535315207	6.04723849769013	3.00587753440629
O	7.03344897506307	3.69823959767172	4.81340600682236
O	6.33451760719379	7.16912816318254	5.80269819087213
Re	6.62163187062172	5.61574641262886	4.62474822591925

List S53. [(ap)(isq•)Re(O)(μ-O)Re(O)(ap)(isq•)], PBE0

C	5.30916470404374	-2.07778014602998	7.50783070158019
C	4.88263107147452	-1.98772519075444	6.19018329285777
H	5.35582906751695	-1.28174021799313	5.52084162419713
C	3.85847526609411	-2.80728653417831	5.74962214234946
H	3.52141194158874	-2.73161354320775	4.72345714992552
C	3.27586994065415	-3.72537249324275	6.61133786149372

H	2.48093051264414	-4.37102017959908	6.25887825233375
C	3.71793465679772	-3.81956261050331	7.92246374001180
H	3.27598226252084	-4.54261636969305	8.59708977538141
C	4.73029198681160	-2.99210784392776	8.37782320421136
H	5.08801039815333	-3.06010904407534	9.39665492244336
C	7.16609112055758	0.39928997623012	9.36500159017567
C	6.04557074711757	-0.36038013621043	8.97337860704483
C	4.80086279561382	-0.17791151517327	9.58792394269080
H	3.95039079138278	-0.74159399322441	9.23957596573710
C	4.68898034123896	0.72882972035037	10.61256976479516
C	5.83783396113862	1.44576512607968	11.01264498112852
H	5.73099663927171	2.14501432007951	11.83046102290249
C	7.07874587667397	1.32395632279401	10.42478310288070
C	3.37402189641854	0.98177367082286	11.33557528559604
C	2.23570021975325	0.15004133295012	10.75620259728348
H	2.43303758832973	-0.92064693690290	10.84169692980427
H	2.06067404765418	0.38275973308141	9.70365422696707
H	1.31352476574497	0.36192068609458	11.30065825008891
C	2.99911991525516	2.46143606615850	11.21559720774358
H	2.86429506630385	2.74240551917568	10.16933730613877
H	3.76634082128744	3.11075311082928	11.63989631346352
H	2.06348691435722	2.65962268763430	11.74407703281715
C	3.53789264076636	0.61468430498324	12.81375410821260
H	3.80457852215127	-0.43882508232056	12.92163727411759
H	2.60404718430856	0.78891079309008	13.35391273619394
H	4.31720433521906	1.20880678926335	13.29373626101754
C	8.29826342132070	2.11360956742221	10.87042559380129
C	7.96764060420236	3.05997033889821	12.01874728806972
H	7.61729077286376	2.52350359979095	12.90374556632429
H	7.20837176670124	3.79162075249073	11.73331177032452
H	8.86745886733598	3.60968979426927	12.30086811055376
C	9.39041359517026	1.14687135005407	11.34295288752186
H	9.69823450288604	0.46860429841609	10.54832299094158
H	9.04228699129024	0.55508300919062	12.19320606558716
H	10.26896115918235	1.71040599781906	11.66608257816581
C	8.82174125939030	2.94771349504740	9.69768009204512
H	9.09872420679918	2.31893871814277	8.85430500693204
H	9.70523487350306	3.51082356171939	10.00810171095948
H	8.06576623794947	3.65954207958980	9.36046975634776
C	8.09914374053947	-3.15557781200424	9.69603233782614
C	7.72683121211941	-2.51931913897286	10.87621237114368
H	8.10392099983128	-1.52717888292695	11.08325685256057
C	6.88098655511864	-3.15821977537002	11.76880494746556
H	6.58217636708729	-2.65069713172331	12.67810594685662
C	6.42289535192542	-4.44084728744724	11.50252660939207
H	5.76627466990121	-4.93982790461311	12.20472265258722

C	6.80945113354524	-5.08193294474785	10.33423687664834
H	6.45319309475302	-6.08203335451241	10.11923149502030
C	7.63765312116895	-4.43935637897971	9.42817960902243
H	7.92729485508522	-4.91457813930285	8.49960149491641
C	10.29952090713763	-2.57470429230329	8.92077709487273
C	10.99880477289002	-3.25895967426532	9.90039087707993
H	10.45500507412364	-3.84174741519537	10.63235152045580
C	12.38796804471183	-3.17904589465158	9.92429775289317
C	13.03314554709027	-2.40683265556282	8.96134498369738
H	14.10926655192856	-2.34304655049535	8.98690967466044
C	12.35973655053424	-1.70085068750844	7.96141201731919
C	10.97653559601355	-1.81403645490315	7.96545598597755
C	13.15070452713096	-3.93496011447519	11.00669441280052
C	12.68759316907103	-3.45077913256960	12.38429843357279
H	11.61950942733278	-3.61773279313029	12.52979865396733
H	12.87786363765160	-2.38202258009899	12.50204520824877
H	13.22215628306488	-3.98432095852180	13.17455453041810
C	12.86362995021219	-5.43339531264925	10.87514275804141
H	11.79852941194976	-5.64749935962172	10.97506075948345
H	13.39412668976678	-5.99225428180323	11.65059155879574
H	13.18857693733319	-5.80548425441129	9.90130500252393
C	14.65798632837945	-3.72720155395166	10.91107068513279
H	14.92935460249463	-2.67560452083860	11.02550621182826
H	15.05654637055989	-4.08214271332961	9.95833113074882
H	15.15441882978382	-4.28668813644740	11.70677171738166
C	13.07962345262835	-0.85446363992163	6.92219904849129
C	12.59383480361543	0.59606428160669	7.01405999627421
H	11.52047848912956	0.67088817242554	6.84887540511342
H	13.09617404147726	1.20812723721119	6.26120333208524
H	12.81857966409224	1.01175456302950	7.99909926909294
C	12.78955421273280	-1.41076331601447	5.52484703399463
H	13.12630894145590	-2.44656919483051	5.44356405756728
H	13.31305302574961	-0.81922042843224	4.77039552263152
H	11.72559490197997	-1.37708534760271	5.29685020187376
C	14.59010314855030	-0.85864950211274	7.12987599948642
H	15.01378740859065	-1.86124995370949	7.04026388396117
H	14.86615912241161	-0.45284575071586	8.10543227700395
H	15.05704141993947	-0.23211068392148	6.36713882565325
C	9.83400502954936	-0.84475935506231	2.57279890035218
C	9.73245839469657	-2.11493809975704	3.12313299949943
H	9.52077476524432	-2.22690642455689	4.17811662025452
C	9.90152486964863	-3.22336149166894	2.31263375349232
H	9.82899315809617	-4.21350632877305	2.74470961664510
C	10.15100291540430	-3.06819844278564	0.95670301719128
H	10.27307887676430	-3.93904253684147	0.32444532036780
C	10.23669787203945	-1.79601227696221	0.41144652784715

H	10.41867987530676	-1.66725384280301	-0.64852923643378
C	10.08591552800659	-0.68017360511041	1.21723265495270
H	10.14460621539816	0.31675916872627	0.80071719267236
C	10.74015963814690	1.13736887784040	3.52066076278101
C	11.99934927736375	0.99830340618884	2.92488530586955
H	12.20825493476897	0.12594766138309	2.32677908823900
C	12.94639510077072	1.97111878703333	3.13042132397721
C	12.61207218269505	3.08365325360220	3.93245717966797
H	13.36497834087971	3.84777655163815	4.06801647080343
C	11.39796598051779	3.25585710064964	4.56258753186689
C	10.44924355491903	2.23574534772276	4.35398052125598
C	14.33574094581902	1.89798743316195	2.51430151300383
C	14.54906207774132	0.60355964321661	1.73847159475187
H	15.56037057200195	0.58592598325397	1.32768500032457
H	13.85133287040358	0.51043334701738	0.90345690693792
H	14.43500444986358	-0.27385514377158	2.37857762195085
C	15.39444147341576	1.97684572702403	3.61794522205514
H	16.39609984046017	1.94270755233786	3.18282098435425
H	15.29675170147934	1.13923031482049	4.31146746247694
H	15.31265007099131	2.89932620481227	4.19428855330860
C	14.51537016451451	3.07615213694513	1.55162259773692
H	15.50844243967798	3.04727415700953	1.09636646941917
H	14.40944523301298	4.03425289174976	2.06331284494305
H	13.77061813007890	3.03732413791700	0.75392858750494
C	11.06360249127832	4.46584975837476	5.41938191576142
C	10.68872499543919	4.00783893810177	6.83159594616971
H	9.81776400533494	3.35650670391346	6.81990076157488
H	10.45765998935496	4.87589479767684	7.45369322972402
H	11.51525021821377	3.46630857428014	7.29591160430637
C	9.88596182262245	5.22227668643168	4.79340306900504
H	10.14632233992597	5.58442930700526	3.79562725550154
H	9.63218426851928	6.08986708834162	5.40740308646851
H	8.99913500227558	4.59452654383605	4.71659599671425
C	12.24373761206284	5.42474461042638	5.52594919599126
H	12.54067036479351	5.81904086547015	4.55120772545410
H	13.11276435183004	4.94948484105038	5.98696987346800
H	11.96056600025232	6.27328378679886	6.15150890675332
C	7.93425662831103	2.20004368511232	1.65706129041236
C	8.99593506736690	3.07906854583296	1.46618356911275
H	9.34061401880137	3.68061492990083	2.29620641756215
C	9.59996142547324	3.17367349520729	0.22268718645761
H	10.43602511726789	3.84880917676821	0.08506540195613
C	9.13515560665658	2.41257605855664	-0.84060040961581
H	9.60590535871639	2.49289256810239	-1.81279218107382
C	8.06504547559665	1.54942135638318	-0.65406176261157
H	7.69911472499639	0.95048688493587	-1.47909157164818

C	7.47088081772457	1.43559239256174	0.59249983763342
H	6.65158319741369	0.74839504243969	0.76130441520089
C	6.34608799163593	2.95973274401820	3.29464301498139
C	5.83500979692962	4.00797606523276	2.53901932488744
H	6.20321598481453	4.16423073338570	1.53554409509970
C	4.86923282166108	4.83123630976186	3.09841348297787
C	4.44016639617629	4.57760594977070	4.40435760783152
H	3.69061419917337	5.22864882880457	4.83079119005490
C	4.92950782974384	3.53816717075561	5.18957962291217
C	5.89603837607860	2.73316716645755	4.59295968972962
C	4.45417136986980	3.27898125398926	6.61129484761130
C	3.82306615910741	1.88521017352705	6.68861374897525
H	2.96897301894618	1.80944119315257	6.01219913575307
H	3.47579450930446	1.68866982217237	7.70554200002867
H	4.53982726970767	1.10961887810973	6.42418532749844
C	5.64292095189233	3.36074431094716	7.57424109288004
H	6.41676317400936	2.64140221430216	7.31360146111622
H	5.31436197624009	3.15001807334956	8.59470186714615
H	6.08418045725891	4.36008455025080	7.55470127346432
C	3.41145760256258	4.29761079705587	7.05769529587337
H	2.51492918064356	4.26423428964901	6.43482344206313
H	3.80634334893643	5.31572386743011	7.04081915554087
H	3.11026232743427	4.07706558424506	8.08386340068748
C	4.27346003224818	6.00896680531236	2.33578704091000
C	2.75754096170988	5.82808602010476	2.21569958760371
H	2.27855778202209	5.78080430820730	3.19470717222731
H	2.51854262110741	4.90588927915107	1.68216402926539
H	2.31771851773811	6.66520188548349	1.66718192178182
C	4.57100543140766	7.30598921036997	3.09432079176910
H	4.14461218659369	7.29064292242076	4.09826392627661
H	4.14973582878826	8.16294526482931	2.56215827315267
H	5.64798073186103	7.45772255300636	3.19122070843481
C	4.85125153561200	6.13550346715498	0.93044671447688
H	5.93088811781602	6.29819322720167	0.95080873103743
H	4.39649413796360	6.98901773680365	0.42327357326542
H	4.65055113899774	5.24596319390466	0.32971915795599
N	6.34897108603292	-1.22283540558801	7.96049046633202
N	8.93551573281782	-2.50956218020642	8.75833293190510
N	9.67633630254992	0.28811642222410	3.41469874007634
N	7.32301472774590	2.06608545008721	2.92400013401972
O	8.24511323095566	0.12970880199970	8.69868190122312
O	10.15144857583668	-1.20631463816493	7.09484610168930
O	9.24887688759675	2.22395222444958	4.84436828823582
O	6.50391190117477	1.69252051442318	5.18801122805919
O	8.22803352345100	0.03604463927336	6.01571261728944
O	7.69908681561795	-2.58802557129350	6.28896229575927

O	7.30343953476642	-0.62298656472407	3.57412763037334
Re	8.23737014350685	-1.33790626796339	7.29274626819420
Re	7.94484119201949	0.75913471658987	4.30881441349454

References

(1) Dolomanov, O. V.; Bourhis, L. J.; Gildea, R. J.; Howard, J. A. K.; Puschmann, H., OLEX2: a complete structure solution, refinement, and analysis program. *J. Appl. Cryst.* **2009**, *42*, 339-341.
(2) Sheldrick, G. M., SHELXT-Integrated space-group and crystal-structure determination. *Acta Crystallogr., Sect. A* **2015**, *71*, 3-8.
(3) Sheldrick, G. M., Crystal structure refinement with SHELXL. *Acta Crystallogr., Sect. C* **2015**, *71*, 3-8.
